# Supplementary material for: Evaluating the reactivation of herpesviruses and inflammation as cardiovascular and cerebrovascular risk factors in antiretroviral therapy initiators in an African HIV-infected population (RHICCA): a protocol for a longitudinal cohort study
Source: BMJ Open. 2019 Sep 12;9(9):e025576. doi: 10.1136/bmjopen-2018-025576 (PMC6747662; doi:10.1136/bmjopen-2018-025576)
Supplement: Supplementary data [file bmjopen-2018-025576supp001.pdf]

RHICCA – Clinical endpoint diagnostic criteria vs 3.5 Apr 2018

RHICCA
Reactivation of herpesviruses and inflammation as cardiovascular and cerebrovascular risk factors in ART initiators

	CONFIRMED	PROBABLE
**INFECTIONS**		
Aspergillosis, invasive pulmonary	**Confirmed: 1 + 2 + 3:**<br>1. Compatible clinical course **(Appendix 13)**,<br>2. CXR abnormality<br>compatible with aspergillosis;<br>3. invasive mycelia consistent with Aspergillus on lung biopsy or positive culture of lung tissue or **positive culture of sputum** collected by any method	**Probable: 1 + 2 + 3:**<br>1. Compatible clinical course **(Appendix 13)**,<br>2. CXR abnormality<br>compatible with aspergillosis;<br>3. invasive mycelia consistent with Aspergillus on tissue biopsy or positive culture at a normally sterile site (e.g., blood) apart from the lungs.
Aspergillosis, other invasive	**Confirmed: 1 + 2 + 3:**<br>1.compatible clinical course **(Appendix 11)**,<br>2. invasive mycelia consistent with Aspergillus on tissue biopsy or clinical evidence of infection,<br>3. positive culture from the affected tissue	**Probable: 1 + 2:**<br>1.clinical evidence of invasive infection **(Appendix 11)**, 2.invasive mycelia consistent with Aspergillus on tissue biopsy or positive culture at a normally sterile site (e.g., blood) apart from the involved tissue
Bartonellosis	**Confirmed 1+ 2:**<br>1.Clinical or histologic evidence of bacillary angiomatosis or bacillary peliosis,<br>2. a positive culture or PCR for *B. quintana* or *B. henselae*	**Probable 1 + 2:**<br>1.Clinical evidence of bacillary angiomatosis or bacillary peliosis **(Appendix 12)**,<br>2. positive silver stain for bacilli from a skin lesion or an affected organ
Candidiasis,oral	**Confirmed:1 + 2 + 3:**<br>1. Macroscopic appearance on examination of the mouth<br>2. microscopic evidence of yeasts or pseudo hyphae<br>3. no evidence of oesophageal involvement -	**Probable: 1 + 2 + 3:**<br>1. a clinical diagnosis of oral candidiasis and/or microscopic evidence of yeasts or pseudo hyphae<br>2. clinical response to treatment<br>3. no evidence of oesophageal involvement
Candidiasis of bronchi, trachea, or lungs	**Confirmed:1 + 2:**<br><br>Macroscopic appearance at bronchoscopy or autopsy<br>microscopic evidence of yeasts or pseudo hyphae	**None**
Candidiasis, esophageal	**Confirmed: 1 + 2:**<br>1. Macroscopic appearance at esophagoscopy or autopsy.<br>2. microscopic evidence of yeasts or pseudo hyphae	**Probable: 1 + 2 + 3:**<br><br>1. Recent onset of retrosternal pain or difficulty on swallowing.<br>2. a clinical diagnosis of oral candidiasis, endoscopic visualization of candidiasis and/or microscopic evidence of yeasts or pseudo hyphae from oropharyngeal mucosa<br>3. clinical response to treatment

Peterson I, *et al. BMJ Open* 2019; 9:e025576. doi: 10.1136/bmjopen-2018-025576

RHICCA – Clinical endpoint diagnostic criteria vs 3.5 Apr 2018

RHICCA
Reactivation of herpesviruses and inflammation as cardiovascular and cerebrovascular risk factors in ART initiators

	CONFIRMED	PROBABLE
**INFECTIONS (CONTINUED)**		
Cryptococcosis, extrapulmonary (not meningitis)	**Confirmed: 1 or 2 or 3:**<br><br>From tissue other than lung or hilum:<br>1. microscopic demonstration of narrow based budding yeast<br>2. positive culture,<br>3. antigen detention	**None**
Cryptococcosis meningitis	**Confirmed: 1 or 2 or 3 or 4:**<br>1. Brain histopathology microscopic demonstration of narrow based budding yeast<br>2. CSF evidence of India ink test<br>3. CSF evidence of positive culture<br>4. CSF evidence of positive antigen detection	**None**
Cryptosporidiosis	**Confirmed: 1 + 2**<br>1. Diarrhea for > 1 month<br>2. positive microscopy	**None**
CMV retinitis	Autopsy demonstration	**Probable 1 + 2:**<br>1. Typical appearance on fundoscopy of discrete patches of retinal whitening, spreading along blood vessels.<br>2. Associated vasculitis, hemorrhage and necrosis, confirmed by ophthalmologist

	CONFIRMED	PROBABLE
**INFECTIONS (CONTINUED)**		
HZV single dermatome	**Confirmed 1+2:**<br>1.multiple ulcerated lesions affecting at least 1 dermatome, and/or 1 or more contiguous dematomes;<br><br>2. positive culture, PCR, or antigen assay from affected tissue	**Probable 1+ 2:**<br>1.multiple typical ulcerated lesions affecting at Least 1 dermatome, and/or 1 or more contiguous dermatomes;<br><br>2. response to an antiviral active against HZV unless resistance is demonstrated
HZV, disseminated	**Confirmed 1+2:**<br>1. multiple ulcerated lesions affecting at least 2 non-contiguous dermatomes, or with generalized cutaneous dissemination HZV involvement of the lung, liver, brain, or other internal organs<br>2. positive culture, PCR, or antigen assay from affected tissue	**Probable 1+2:**<br>1. multiple typical ulcerated lesions affecting at least 2 non-contiguous dermatomes, or with generalized cutaneous dissemination<br>2. response to an antiviral active against HZV unless resistance is demonstrated
HSV mucocutaneous ulceration	**Confirmed 1 +2:**<br>1.Ulceration for > 1 Month<br>2. Histology, culture, PCR, or detection of antigen from affected tissue	**Probable 1 + 2:**<br>1. Typical HSV ulceration for > 1 month,<br>2. response to an antiviral active against HZV unless resistance is demonstrated
Histoplasmosis, disseminated or extrapulmonary	**Confirmed 1+2:**<br>1.Compatible symptoms,<br>2. histology or culture or elevated blood or urine antigen levels	None
Isosporiasis	**Confirmed 1 + 2:**<br>1.Diarrhea for > 1 month<br>2.microscopic identification of *Isospora belli*	None

RHICCA – Clinical endpoint diagnostic criteria vs 3.5 Apr 2018

Microsporidiosis	**Confirmed 1 + 2:**<br>1.Diarrhea for > 1 month<br>2.Microscopic identification of Microsporidia	None
MAC and other mycobacterial disseminated diseas	**Confirmed 1 + 2:**<br>1. Fever, fatigue, anemia or diarrhea<br>2.positive culture from blood, body fluids or tissue other than pulmonary, hilar or stool	**Probable 1+2+3:**<br>1. Fever, fatigue, anemia or diarrhea<br>2. **AFB** or positive direct MAC PCR in blood, body fluids or tissue other than pulmonary, hilar or stool<br>3. no concurrent non-pulmonary TB

	CONFIRMED	PROBABLE	POSSIBLE
M. tuberculosis disease, pulmonary	**Confirmed 1+2:**<br>1.Compatible symptoms of fever, dyspnea, cough, weight loss or fatigue<br>2. culture or PCR from **sputum** or bronchial lavage or lung tissue	**Probable 1+2+3+4:**<br>1.Symptoms of fever, dyspnea, cough, weight loss or fatigue<br>2.abnormal chest X-ray,<br>3. AFBs seen in sputum or lavage or lung tissue but not grown in culture,<br>4.responds to treatment	**Possible 1+2+3+4:**<br>1.Symptoms of fever, dyspnea , cough, weight loss or fatigue<br>2.abnormal chest X-ray compatible with pulmonary TB (such as upper lobe cavitation, pleural exudate)<br>3. No other etiology for pulmonary symptoms and signs identified,<br>4.Responds to anti tuberculosis treatment
M. tuberculosis disease, Extrapulmonary (not meningitis)	**Confirmed 1+2:**<br>1. Compatible symptoms<br>2. culture or PCR or MTB Xpert from blood or affected tissue (i.e. pericardial, ascites, and lymph glands)	**Probable 1+2+3**:<br>1.Compatible symptoms<br>2. AFBs seen from affected tissue or blood<br>3.concurrent diagnosis of pulmonary TB or responds to treatment	**Possible 1+2+3:**<br>1.Compatible symptoms<br>2. No other etiology for symptoms and signs identified<br>3.concurrent diagnosis of pulmonary TB or responds to treatment
M. tuberculosis disease, meningitis	**Confirmed 1+2:**<br>**1.** Clinical symptoms of meningism **(Appendix 7)**<br>**2.** Tissue/CSF culture, or PCR, or AFB or MTB Xpert	**Probable 1+ a score ≥12 (Appendix: Table 2):**<br>1. Clinical symptoms of meningism **(Appendix 7)**<br>2. A score ≥12, based on clinical, CSF, cerebral brain imaging criteria or evidence of TB elsewhere	
Nocardiosis	**Confirmed 1+2:**<br>1. Clinical evidence of invasive Infection **(Appendix 8)**<br>2. a positive culture from the affected tissue or blood	**Probable 1+2:**<br>1. Clinical evidence of invasive Infection **(Appendix 8)**<br>2. microscopic evidence of bronchial weakly acid fast organisms from the affected tissue	

RHICCA – Clinical endpoint diagnostic criteria vs 3.5 Apr 2018

RHICCA
Reactivation of herpesviruses and inflammation as cardiovascular and cerebrovascular risk factors in ART initiators

Pneumocystis jirovecii pulmonary	**Confirmed 1+2:**<br>1. compatible clinical syndrome **(Appendix 9)**<br>2. microscopic or histological demonstration of *P. jirovecii* cysts in a pulmonary specimen	Probable 1+2+3+4+5<br>1. dyspnea or cough, or fever progressive over > 1 week<br>2. **diffuse chest x-ray abnormality** or, if on inhalational pentamidine, diffuse upper lung field abnormality<br>3. evidence of hypoxia<br>4. not suggestive of bacterial pneumonia (i.e., not purulent sputum or hemoptysis, no bacterial pathogen identified in blood or bronchial wash)<br>5. response to PcJ treatment
Pneumocystis jirovecii, extrapulmonary	**Confirmed 1+2:**<br>1. compatible clinical syndrome<br>2. microscopic or histological demonstration of *P. jirovecii* cysts in a tissue other than pulmonary specimen	None

Peterson I, *et al. BMJ Open* 2019; 9:e025576. doi: 10.1136/bmjopen-2018-025576

RHICCA – Clinical endpoint diagnostic criteria vs 3.5 Apr 2018

RHICCA
Reactivation of herpesviruses and inflammation as cardiovascular and cerebrovascular risk factors in ART initiators

	CONFIRMED	PROBABLE
**INFECTIONS (CONTINUED)**		
Pneumonia, **SINGLE EPISODE (isolated)**<br>bacterial, excludes: (a) post-obstructive pneumonias, including in those with intrapulmonary/bronchial lesions, (b) aspiration pneumonia, including in those with decreased consciousness levels, (c) nosocomial ventilator-associated pneumonias	**Confirmed 1+2+3:**<br>pneumonia episodes must occur after enrollment;<br>1. Signs and symptoms suggestive of bacterial pneumonia (**appendix 10**)<br>2. Focal CXR abnormality compatible with bacterial pneumonia,<br>3. identification of a bacterial pathogen by positive blood culture, positive bronchoscopic sputum culture, detection of Legionella or pneumococcal antigen in urine or blood or diagnostic serologic findings	**Probable 1+2:**<br>pneumonia episodes must occur after enrollment;<br>1.Signs and symptoms suggestive of bacterial pneumonia (**Appendix 10**)<br>2. Focal CXR abnormality compatible with Bacterial pneumonia
Pneumonia, **recurrent**<br>bacterial, excludes: (a) post-obstructive pneumonias, including in those with intrapulmonary/bronchial lesions, (b) aspiration pneumonia, including in those with decreased consciousness levels, (c) nosocomial ventilator-associated pneumonias	**Confirmed 1+2+3+4+5**<br>Second pneumonia episodes must occur after enrollment and after an isolated pneumonia, within 365 days of the first episode and with strong clinical evidence that the first episode was cured;<br>1. Signs and symptoms of second event suggestive of bacterial pneumonia (**Appendix 10**)<br>2. Focal CXR abnormality compatible with bacterial pneumonia,<br>3. identification of a bacterial pathogen by positive blood culture, positive bronchoscopic sputum culture, detection of Legionella or pneumococcal antigen in urine or blood or diagnostic serologic findings<br>4. the second pneumonia had onset of symptoms < 365 days after the first episode<br>5. there is strong evidence that the first episode was cured such as an intervening clear CXR or absence of symptoms after > 1 month off antibacterial effective against pathogens commonly producing pneumonia	**Probable 1+2+3+4:**<br>Second pneumonia episodes must occur after enrollment and after an isolated pneumonia, within 365 days of the first episode and with strong clinical evidence that the first episode was cured;<br>1. Signs and symptoms suggestive of bacterial pneumonia (**Appendix 10**)<br>2. focal CXR abnormality compatible with bacterial pneumonia<br>3. the second pneumonia had onset of symptoms < 365 days after the first episode<br>4. there is strong evidence that the first episode was cured such as an intervening clear CXR or absence of symptoms after > 1 month off antibacterials effective against pathogens commonly producing pneumonia
PML (progressive multifocal leukoencephalopathy)	**Confirmed 1 or 2:**<br>1. positive histology,<br>2. compatible clinical (**Appendix 11**) and radiologic course and positive CSF PCR for JK virus	**Probable 1+2+3:**<br>1. Consistent symptoms (**Appendix 11**),<br>2. brain image consistent with PML,<br>3. no response to toxo treatment or toxoplasma [illegible]
Salmonella blood stream infection or bacteraemia, isolated	**Confirmed 1:**<br>A septic episode must occur after enrollment;<br>1. Positive blood or tissue culture	None
Salmonella blood stream infection or bacteraemia, recurrent	**Confirmed 1:**<br>A second septic episode must occur after enrollment and after an isolated episode;<br>1. Has met the criteria of isolated Salmonella septicemia<br>2. Positive blood or tissue culture on the second episode<br>3. the second septicemia had onset of symptoms < 365 days after the first episode<br>4. the second septicemia must be due to a different Salmonella serotype or there must be strong evidence that the first episode was cured such as a negative blood culture off effective antibacterials for > 1 week or absence of symptoms off antibacterials for > 1 month	None

RHICCA – Clinical endpoint diagnostic criteria vs 3.5 Apr 2018

RHICCA
Reactivation of herpesviruses and inflammation as cardiovascular and cerebrovascular risk factors in ART initiators

Toxoplasmosis of brain	**Confirmed 1+2+3:**<br>1. Compatible clinical findings **(Appendix 12)**<br>2. Compatible radiological findings<br>3. Detection of T gondii in the **CSF** or brain tissue (i.e. microscopy or PCR)	**Probable 1+2+3:**<br>1. Symptoms of focal intracranial abnormality or decreased consciousness<br>2. brain image consistent with lesion(s) enhanced by contrast<br>3. positive toxoplasma serology or responds to treatment clinically or by scan

	CONFIRMED	PROBABLE
**NEOPLASMS**		
Cervical carcinoma, invasive	**Confirmed 1:**<br>1. Histology (NOT carcinoma-in-situ)	**None**
Kaposi sarcoma, (mucocutaneous or visceral)	**Confirmed 1:**<br>1. Histology	1. Highly typical appearance<br>2. persistence for > 1 month
Lymphoma, primary, of brain	**Confirmed 1:**<br>1. Histology of brain tissue	**Probable 1+2+3:**<br>1. Symptoms consistent with lymphoma<br>2. at least one CNS lesion with mass effect<br>3. lack of clinical or radiographic response at least 2 weeks of treatment for toxoplasmosis
Lymphoma, Hodgkin's	1. Histology	None
Lymphoma, non-Hodgkin's, all cell types	**Confirmed 1:**<br>1. Histology	None
**NEUROLOGICAL**		
HIV-related encephalopathy (including AIDS Dementia Complex)	None	**Probable 1+2+3+4:**<br>1. Cognitive or motor dysfunction interfering with usual activity, progressive over weeks or months<br>2. no other condition to explain the findings<br>3. brain image obtained and suggests no other causes<br>4. grade 2 or worse impairment in at least 2 domains by NARS (**appendix – table 1**) excluding abnormal domains at trial entry. (For persons with abnormal domains at entry worsening by at least two grades meets criteria.)

Peterson I, *et al. BMJ Open* 2019; 9:e025576. doi: 10.1136/bmjopen-2018-025576

RHICCA – Clinical endpoint diagnostic criteria vs 3.5 Apr 2018

RHICCA
Reactivation of herpesviruses and inflammation as cardiovascular and cerebrovascular risk factors in ART initiators

CARDIOVASCULAR DISEASES		
Acute Myocardial Infarction	**Confirmed: One of the following 5 criteria (adapted from 2007 Universal Definition of Myocardial Infarction): [1 + (2 or 3 or 6)] or 4 or 5**<br>1. Rise and/or fall of cardiac biomarkers (preferably troponin), with at least one value above 99th percentile of upper reference limit (URL);<br>2. Occurrence of a compatible clinical syndrome, including symptoms (such as chest pain **– see Appendix 1**) consistent with myocardial ischemia;<br>3. ECG changes indicative of new ischemia (new ST-changes or new left bundle branch block [LBBB]), or development of pathological Q waves on the ECG;<br>4. Sudden unexpected cardiac death involving cardiac arrest before biomarkers are obtained or before a time when biomarkers appear, along with (a) new ST-changes or new LBB, or (b) evidence of fresh thrombus on coronary angiography or at autopsy<br>5. Pathologic findings of acute myocardial infarction (including acute MI demonstrated as the cause of death on autopsy)<br>6. Development of a) evolving new Q waves, or b) evolving ST elevation, preferably based on at least 2 ECGs taken during the same hospital admission	**Probable 1 and 2:**<br>1. Occurrence of a compatible clinical syndrome (**Appendix 1**), including symptoms (such as chest pain) consistent with myocardial ischemia)<br>2. Development of a) evolving new Q waves, or b) evolving ST elevation, preferably based on at least; ECGs taken during the same hospital admission.
Peripheral vascular disease	**Confirmed (1+2) or (1+3):**<br>1. Compatible clinical signs and symptoms (**see Appendix 3**)<br>2. Positive results on diagnostic imaging studies (e.g., Doppler ultrasound, contrast arteriography, MRI arteriography); 3. Ankle Brachial Pressure Index < 0.90 in non-diabetics	**Probable 1:**<br>1. Compatible clinical signs and symptoms (**see Appendix 3**)
Stroke	**Confirmed (1+2) or 3 or 4 or 5:**<br>1. Acute onset with a clinically compatible course, including unequivocal objective findings of a localizing neurologic deficit **(appendix 4)**;<br>2. CT or MRI compatible with diagnosis of stroke and current neurologic signs and symptoms<br>3. Stroke diagnosed as cause of death at autopsy<br>4. Compatible clinical history and positive lumbar puncture compatible with subarachnoid hemorrhage<br>5. Death certificate or death note from medical record listing stroke as cause of death	**Probable (1+2) or (1+3):**<br>1. Acute onset with a clinically compatible course, including unequivocal objective findings of a localizing neurologic deficit **(appendix 4)**;<br>2. Positive lumbar puncture compatible with subarachnoid hemorrhage<br>3. Death certificate or death note from medical record listing stroke as cause of death
Congestive heart failure	**Confirmed (1+2) or (1+3) or (1+4):**<br>1. Clinical signs and symptoms compatible with left or right sided heart failure [**Appendix 5]** without an alternative explanation<br>2. Hemodynamic measurements, radionucleotide ventriculography, echocardiogram, cardiac catheterization, or multiple gated acquisition scan showing a decreased ejection fraction of < 45%<br>3. Echocardiogram, cardiac catheterization or other studies showing evidence of increased left atrial pressure or right heart failure;<br>4. Elevated levels of Brain Natriuretic Peptide (BNP) or NT-proBNP	**Probable 1+2+3:**<br>1. Clinical signs and symptoms compatible with left or right sided heart failure [**Appendix 5]** without an alternative explanation<br>2. Chest x-ray or other imaging study showing evidence of congestive heart failure, including cardiac enlargement;<br>3. Documentation of treatment for congestive heart failure

Peterson I, *et al. BMJ Open* 2019; 9:e025576. doi: 10.1136/bmjopen-2018-025576

RHICCA – Clinical endpoint diagnostic criteria vs 3.5 Apr 2018

RHICCA
Reactivation of herpesviruses and inflammation as cardiovascular and cerebrovascular risk factors in ART initiators

Coronary artery disease requiring drug treatment	**Confirmed (1 or 2) + 3:**<br><br>1. Evidence of myocardial ischemia based on either diagnostic imaging (such as a stress echocardiogram or thallium scan) or diagnostic changes on an electrocardiogram (such as during stress testing or an episode of chest pain)<br>2. Evidence of coronary artery disease based on coronary angiography or other diagnostic imaging<br>3. Use of medication given to treat or prevent angina (e.g., nitrates, beta blockers, calcium channel blockers)	**Probable 1+2:**<br><br>1. Other evidence of myocardial ischemia and/or coronary artery disease (including that based primarily upon symptoms and clinical presentation, such as chest pain with exertion)<br>2. Use of medication given to treat or prevent angina (e.g., nitrates, beta blockers, calcium channel blockers)
Deep vein thrombosis	**Confirmed 1:**<br>1. Diagnosis of deep vein thrombosis (DVT) by contrast venography, or **ultrasonography** other comparable imaging techniques;	**Probable (1)+2+3:**<br>1. An elevated **D-dimer test**;<br>2. A score on the Wells Clinical Prediction Rule for DVT of ≥ 3 points;<br>3. Absence of alternative diagnosis as likely or greater than that of deep venous thrombosis.<br>**Wells Clinical Prediction Rule for DVT (Appendix 6)**
**SYSTEMIC DISEASES**		
Anaemia<br>NOT FOR ERC<br>TO BE DETERMINED RETROSPECTIVELY	**Confirmed 1**<br>Classified according to both WHO and DAIDS thresholds for severe/grade 3-4 anaemia	
Chronic Kidney disease	**Confirmed: 1 or 2**<br><br>1. Kidney damage for >3 months, as defined by structural or functional abnormalities of the kidney with or without decreased GRF, manifest by either;<br>- Pathological abnormalities; or<br>- Markers of kidney damage, including abnormalities in the composition of the blood or urine or abnormalities in imaging results<br>2. GFR <60mL/min/1.73m2 for >3months, with or without kidney disease (estimated by CKD-EPI)	**Confirmed: 1 or 2**<br><br>1. Isolated Kidney damage, as defined by structural or functional abnormalities of the kidney with or without decreased GRF, manifest by either;<br>- Pathological abnormalities; or<br>- Markers of kidney damage, including abnormalities in the composition of the blood or urine or abnormalities in imaging results<br>2. Isolated GFR <60mL/min/1.73m2, with or without kidney disease (estimated by CKD-EPI)
End-stage renal disease	**Confirmed: 1**<br>1.Hemodialysis or peritoneal dialysis documented in a clinical note for a period of at least three months;	**Probable: 1**<br>1. Hemodialysis or peritoneal dialysis documented in a clinical note for a period of at least one month and up to the time of death in a patient who dies within three months after dialysis begins
Diabetes Mellitus<br><br>NOT FOR ERC<br>TO BE DETERMINED RETROSPECTIVELY	**Confirmed: 1 or 2 or 3 or 4**<br><br>1. Symptoms of diabetes plus casual plasma glucose concentration ≥ 200 mg/dL (11.1 *mmol/L*). (Casual is defined as any time of day without regard to time since last meal. The classic symptoms of diabetes include polyuria and polydipsia.)<br><br>2. Fasting plasma glucose ≥ 126 mg/dL (7.0 *mmol/L*). (Fasting is defined as no caloric intake for at least 8 hours.)<br><br>3. 2-hour post-load glucose ≥ 200 mg/dL (11.1 *mmol/L*) during an oral glucose tolerance test. (The test should be performed as described by WHO, using glucose load containing the equivalent of 75 g anhydrous glucose dissolved in water.)<br><br>4.An **HbA1c** of 48mmol/mol (6.5%) or above.	**None**

Peterson I, *et al. BMJ Open* 2019; 9:e025576. doi: 10.1136/bmjopen-2018-025576

RHICCA – Clinical endpoint diagnostic criteria vs 3.5 Apr 2018

RHICCA
Reactivation of herpesviruses and inflammation as cardiovascular and cerebrovascular risk factors in ART initiators

Decompensate Liver disease	**Confirmed: 1+2**<br>1. Histologic, radiographic, or ultrasound evidence of cirrhosis, as documented by one of the following:<br>a. Histologic evidence of cirrhosis obtained by liver biopsy or autopsy<br>b. MRI or CT consistent with cirrhosis<br>c. A positive result on ultrasound imaging consistent with cirrhosis<br><br>2. Clinical evidence of decompensation, as documented by one of the following, and without an alternative explanation:<br>a. Ascites<br>b. Hepatic encephalopathy<br>c. Bleeding from gastric or esophageal varices<br>d. Spontaneous bacterial peritonitis	**Probable: 1**<br><br>1. Clinical evidence of decompensation, as documented by one of the following, and without an alternative explanation:<br>a. Ascites<br>b. Hepatic encephalopathy<br>c. Bleeding from gastric or esophageal varices<br>d. Spontaneous bacterial peritonitis
Hypertension	**Confirmed:** 1 or 2<br>1. An average of three blood pressure (BP) readings of systolic of 140 mm Hg or above and/or diastolic of 90 mm Hg or above on the same day and persist 5-7 days after the initial reading.<br>2. An isolated reading of 140mg systolic or 90mg diastolic and presence of the following end-organ disease:<br>a. Cardiac (i.e. left ventricular hypertrophy meeting the ECG criteria [**Appendix 2**] on evidence on cardiac echocardiogram)<br>b. Renal (i.e. microalbuminuria [urinary albumin excretion of 30-300mg/dl], elevated creatinine, reduced estimated GFR (60-90ml/min)<br>c. Retinal(i.e. hypertensive retinal changes)<br>d. Vascular disease (i.e. stroke [persisting on day 7], peripheral vascular disease, myocardial infarction, coronary artery disease requiring drug treatment, congestive cardiac failure)	**Probable: 1**<br><br>1. An average of three BP readings of systolic of 140 mm Hg or above and/or diastolic of 90 mm Hg or above on the same day.
Hyperlipidemia<br><br>NOT FOR ERC<br>TO BE DETERMINED RETROSPECTIVELY	**Confirmed: 1 or 2**<br>1. Fasting total cholesterol >200mg/dl (>5.2 mmol/L) or LDL cholesterol >130mg/dl (>3.4mmol/l) or Triglycerides >150 mg/dl (1.7 mmol/L)<br>2. Non-fasting total cholesterol >240mg/dl (>6.2 mmol/L) or LDL cholesterol >160mg/dl (>4.1 mmol/L) or Triglycerides >200 mg/dl (2.3mmol/L)	**None**
HIV wasting syndrome	None	**Probable:1 + 2 + 3**<br>1. unexplained, involuntary weight loss >10% from baseline,<br>2. persistent diarrhea with > 2 liquid stools/d for > 1 month or weakness for > 1 month or fever for > 1 month,<br>3. tests for alternate causes of weight loss, such as cancer, TB, MAC, cryptosporidiosis or other specific causes of weight loss, if obtained, should be negative

Peterson I, *et al. BMJ Open* 2019; 9:e025576. doi: 10.1136/bmjopen-2018-025576

RHICCA – Clinical endpoint diagnostic criteria vs 3.5 Apr 2018

RHICCA

Reactivation of herpesviruses and inflammation as cardiovascular and cerebrovascular risk factors in ART initiators

Appendix

1. Clinical syndrome of Myocardial infarction (a+ b +d) or (c+d)
    a. Chest pain (with associated clamminess, pallor)
    b. Radiation to the upper extremity and jaw
    c. Epigastric discomfort with exertion or at rest
    d. Severe discomfort lasting for more than 20 minutes

2. ECG criteria for LVH

Any two of the following 3 criteria's should be met:

Sokolow Lyon Criteria<br><br>• S in V_1 or V_2 + R in V_5 or V_6 (whichever is larger) ≥ 35 mm (≥ 7 large squares)<br>• R in aVL ≥ 11 mm<br><br>**Meets all of Sokolow Lyon criteria to be diagnostic**	
Cornell voltage criteria<br><br>ECG diagnosis of LVH involve measurement of the sum of the R wave in lead aVL and the S wave in lead V_3. The Cornell criteria for LVH are:<br><br>• S in V_3 + R in aVL > 28 mm (men)<br>• S in V_3 + R in aVL > 20 mm (women)<br><br>**Meets all of Cornell voltage criteria to be diagnostic**	
Romhilt-Estes point score system ECG Criteria<br>Voltage Criteria (any of):<br>1. R or S in limb leads ≥20 mm<br>2. S in V_1 or V_2 ≥30 mm<br>3. R in V_5 or V_6 ≥30 mm<br>--<br>ST-T Abnormalities:<br><br>1. ST-T vector opposite to QRS without digitalis<br>2. ST-T vector opposite to QRS with digitalis<br>3. Negative terminal P mode in V_1 1 mm in depth and 0.04 sec in duration (indicates left atrial enlargement)<br>4. Left axis deviation (QRS of -30° or more)<br>5. QRS duration ≥0.09 sec<br>6. Delayed R wave peak time (intrinsicoid deflection) in V_5 or V_6 (>0.05 sec)<br><br>**Romhilt-Estes point score >4 is diagnostic**	Points<br><br>3<br><br><br><br><br><br><br>3<br>1<br>3<br><br>2<br>1<br>1

3. Clinical syndrome of Peripheral vascular disease (a+ (b or c or d)
    a. Painful cramping in the hip, thigh or calf muscles after certain activities, such as walking or climbing stairs (claudication)
    b. femoral bruit
    c. decreased peripheral pulses
    d. change in color or temperature of limb suggesting peripheral arterial disease

Peterson I, *et al. BMJ Open* 2019; 9:e025576. doi: 10.1136/bmjopen-2018-025576

RHICCA – Clinical endpoint diagnostic criteria vs 3.5 Apr 2018

RHICCA
Reactivation of herpesviruses and inflammation as cardiovascular and cerebrovascular risk factors in ART initiators

4. Clinical syndrome of stroke; should meet the 3 criteria's;

1. **Sudden onset**	
2. **Focal deficit** (or global disturbance but not seizures)	**Large artery disease (anterior circulation syndrome)**<br>Hemi-paresis + Hemi-sensory loss + higher cortical dysfunction (gaze paresis, language impairment [expression + comprehension], visual field defect, hemi-neglect)<br><br>**Large artery disease (posterior circulation syndrome)**<br>Vertigo, visual field defect, gaze paresis, double vision, swallowing difficultly, crossed signs [contralateral limb weakness and ipsilateral cranial nerves abnormality], ataxic limb and gait, drowsy/loss of consciousness<br><br>**Small vessel disease (lacunar syndrome)**<br>Pure hemi-sensory loss<br>Pure hemiparesis<br>Pure sensorimotor<br>Pure ataxic hemiparesis (including dysarthria-clumsy hand syndrome)<br><br>Thunderclap headache*
3. **Lasting > 24 hours** (<24 hours is a TIA)	
*seen in those with a suspicion of subarachnoid or venous stroke. In this case criteria 1 and 3 does not necessarily have to be met	

5. Clinical syndrome of congestive heart failure:

Using the Framingham criteria relies on clinical signs and symptoms; 1 or more major and two or more minor criteria are clinically suggestive of heart failure:

Major criteria

A. Acute pulmonary edema
B. Cardiomegaly
C. Hepatojugular reflex
D. Neck vein distention
E. Paroxysmal nocturnal Dyspnea or Orthopnea
F. Pulmonary crackles
G. Third Heart Sound (S3 Gallup Rhythm)

Minor Criteria

A. Ankle edema
B. Dyspnea on exertion
C. Hepatomegaly
D. Nocturnal cough
E. Pleural Effusion
F. Tachycardia (Heart Rate >120 beats per minute)

Peterson I, *et al. BMJ Open* 2019; 9:e025576. doi: 10.1136/bmjopen-2018-025576

RHICCA – Clinical endpoint diagnostic criteria vs 3.5 Apr 2018

6. Wells Clinical Prediction Rule for DVT (Adapted from: Wells PS et al. Lancet 1997;350:1796).

One point for each of the following:

- Active cancer (treatment ongoing or within previous 6 months, or palliative)
- Paralysis, paresis, or plaster immobilization of lower extremities
- Recently bedridden for more than 3 days, or major surgery, within 4 weeks
- Localized tenderness along distribution of the deep venous system
- Entire leg swollen
- Calf swelling by more than 3 cm when compared with the asymptomatic leg (measured 10 cm below tibial tuberosity)
- Pitting edema (greater in the symptomatic leg)
- Collateral superficial veins (non-varicose)

7. Clinical symptoms of meningism
   Meninigism is the triad of nuchal **rigidity** (neck stiffness), **photophobia** (intolerance of bright light) and **headache**.

8. Clinical symptoms of norcardia

   Symptoms vary and depend on the organs involved.

   If in the lungs, symptoms may include:
   - Chest pain when breathing (may occur suddenly or slowly)
   - Coughing up blood
   - Fevers
   - Night sweats
   - Weight loss

   If in the brain, symptoms may include:
   - Fever
   - Headache
   - Seizures
   - If the skin is affected, symptoms may include:
   - Skin breakdown
   - Skin breakdown and abnormal passage or draining tract (fistula)
   - Ulcers or nodules with infection sometimes spreading along lymph nodes

   Some people with nocardia infection have no symptoms.

9. Symptoms of Pneumocystis Pneumonia
   - Fever.
   - Mild and dry cough or wheezing.
   - Shortness of breath, especially with activity.
   - Rapid breathing.
   - Fatigue.
   - Major weight loss.
   - Chest pain when you breathe.

10. Clinical syndrome of bacterial pneumonia
    - cough with thick yellow, green, or blood-tinged mucus.
    - chest pain that worsens when coughing or breathing.
    - sudden onset of chills.
    - fever of 102°F or above (fever lower than 102°F in older persons)
    - headache.
    - muscle pain.
    - breathlessness or rapid breathing.

Peterson I, *et al. BMJ Open* 2019; 9:e025576. doi: 10.1136/bmjopen-2018-025576

RHICCA – Clinical endpoint diagnostic criteria vs 3.5 Apr 2018

11. Clinical finding of Central Nervous System PML
    - Deficits in motor function, especially **weakness** and **clumsiness**, are common
    - associated altered mental state or behaviour and fever

12. Clinical finding of CNS toxoplasmosis
    - Headaches
    - Seizures
    - Focal neurological deficit of a subacute onset
    - confusion and coma
    - A lung infection, causing cough, fever, and shortness of breath may co-exist.
    -

13. Clinical symptoms suggest of Aspergillosis;

- Fever and chills.
- Cough that brings up blood-streaked sputum (hemoptysis)
- Severe bleeding from the lungs.
- Shortness of breath.
- Chest or joint pain.
- Headaches or eye symptoms.
- Nosebleed
- Facial swelling on one side

Table 1: Abbreviated NARS (Neuropsychiatric AIDS Rating Scale) Grading for HIV Encephalopathy
Adapted from: Price RW, Brew BJ. The AIDS dementia complex. J Infect Dis 1988; 158 (5): 1079-83, and Hughes CP, Berg L, Danziger WL. A new clinical scale for the staging of dementia. Brit J Psych 1982; 140: 566-92.

NARS stage	Cognitive-Behavioral Domains					
	**Orientation**	**Memory**	**Motor**	**Behavior**	**Problem solving**	**Activities of daily living**
**0.5**	fully oriented	complains of memory problems	fully ambulatory slightly slowed movements	normal	has slight mental slowing	slight impairment in business dealings
**1**	fully oriented, may have brief periods of “spaciness"	mild memory problems	balance, co-ordination and handwriting difficulties	more irritable, labile or apathetic, withdrawn	difficulty planning and completing work	can do simple daily tasks, may need prompting
**2**	some disorientation	memory moderately impaired, new learning impaired	ambulatory but may require walking aid	some impulsivity or agitated behavior	severe impairment, poor social judgement, gets lost easily	needs assistance with ADLs
**3**	frequent disorientation	severe memory loss, only fragments of memory remain	ambulatory with assistance	may have organic psychosis	judgement very poor	cannot live independently
**4**	confused and disoriented	virtually no memory	bedridden	mute and unresponsive	no problem solving ability	nearly vegetative

Peterson I, *et al. BMJ Open* 2019; 9:e025576. doi: 10.1136/bmjopen-2018-025576

RHICCA – Clinical endpoint diagnostic criteria vs 3.5 Apr 2018

Peterson I, *et al. BMJ Open* 2019; 9:e025576. doi: 10.1136/bmjopen-2018-025576

RHICCA – Clinical endpoint diagnostic criteria vs 3.5 Apr 2018

Table 2: Diagnostic criteria for classification of definite, probable, possible, and not tuberculosis meningitis (Marais S, et al. Lancet Infect Dis 2010)

**Clinical criteria**	Diagnostic score (Maximum category score=6)
Symptom duration of more than 5 days	4
Systemic symptoms suggestive of tuberculosis (one or more of the following): weight loss (or poor weight gain in children), night sweats, or persistent cough for more than 2 weeks	2
History of recent (within past year) close contact with an individual with pulmonary tuberculosis or a positive TST or IGRA (only in children <10 years of age)	2
Focal neurological deficit (excluding cranial nerve palsies)	1
Cranial nerve palsy	1
Altered consciousness	1
**CSF criteria**	(Maximum category score=4)
Clear appearance	1
Cells: 10–500 per µl	1
Lymphocytic predominance (>50%)	1
Protein concentration greater than 1 g/L	1
CSF to plasma glucose ratio of less than 50% or an absolute CSF glucose concentration less than 2.2mmol/L	1
**Cerebral imaging criteria**	(Maximum category score=6)
Hydrocephalus	1
Basal meningeal enhancement	2
Tuberculoma	2
Infarct	1
Pre-contrast basal hyperdensity	2
Evidence of tuberculosis elsewhere	(Maximum category score=4)
**Chest radiograph** suggestive of active tuberculosis: signs of tuberculosis=2; miliary tuberculosis=4	2/4
**CT/ MRI/** ultrasound evidence for tuberculosis outside the CNS 2	
**AFB** identifi ed or *Mycobacterium tuberculosis* cultured from another source—ie, sputum, lymph node, gastric washing, urine, blood culture	2<br>4
Positive commercial *M tuberculosis* NAAT from extra-neural specimen	4

Exclusion of alternative diagnoses

An alternative diagnosis must be confirmed microbiologically (by stain, culture, or NAAT when appropriate), serologically (eg, syphilis), or histopathologically (eg, lymphoma). The list of alternative diagnoses that should be considered, dependent upon age, immune status, and geographical region, include: pyogenic bacterial meningitis, cryptococcal meningitis, syphilitic meningitis, viral meningo-encephalitis, cerebral malaria, parasitic or eosinophilic meningitis (*Angiostrongylus cantonesis*, *Gnathostoma spinigerum*, toxocariasis, cysticercosis), cerebral toxoplasmosis and bacterial brain abscess (space-occupying Lesion on cerebral imaging)and malignancy (eg, lymphoma)

TST=tuberculin skin test. IGRA=interferon-gamma release assay. NAAT=nucleic acid amplification test. AFB=acid-fast bacilli. The individual points for each criterion (one, two, or four points) were determined by consensus and by considering their quantified diagnostic value as defined in studies.

Key:
Bold text: of the options available likely to be the only tool available in a Malawi setting
Greyed out text: ideal investigation but not available in a Malawi setting

Peterson I, *et al. BMJ Open* 2019; 9:e025576. doi: 10.1136/bmjopen-2018-025576
