# Supplementary material for: Evaluating the reactivation of herpesviruses and inflammation as cardiovascular and cerebrovascular risk factors in antiretroviral therapy initiators in an African HIV-infected population (RHICCA): a protocol for a longitudinal cohort study
Source: BMJ Open. 2019 Sep 12;9(9):e025576. doi: 10.1136/bmjopen-2018-025576 (PMC6747662; doi:10.1136/bmjopen-2018-025576)
Supplement: Author's manuscript [file bmjopen-2018-025576.draft_revisions.pdf]

BMJ Open is committed to open peer review. As part of this commitment we make the peer review history of every article we publish publicly available.

When an article is published we post the peer reviewers' comments and the authors' responses online. We also post the versions of the paper that were used during peer review. These are the versions that the peer review comments apply to.

The versions of the paper that follow are the versions that were submitted during the peer review process. They are not the versions of record or the final published versions. They should not be cited or distributed as the published version of this manuscript.

BMJ Open is an open access journal and the full, final, typeset and author-corrected version of record of the manuscript is available on our site with no access controls, subscription charges or pay-per-view fees (http://bmjopen.bmj.com).

If you have any questions on BMJ Open's open peer review process please email info.bmjopen@bmj.com

BMJ Open

Protocol for a longitudinal cohort study to evaluate the reactivation of herpesviruses and inflammation as cardiovascular and cerebrovascular risk factors in antiretroviral therapy initiators, in an African HIV-infected population (RHICCA)

Journal:	*BMJ Open*
Manuscript ID	bmjopen-2018-025576
Article Type:	Protocol
Date Submitted by the Author:	22-Jul-2018

Complete List of Authors:	Peterson, Ingrid; Liverpool School of Tropical Medicine; Malawi Liverpool Wellcome Trust Clinical Research Programme<br>Ntsui, Ntobeko; University of Cape Town<br>Jambo, Kondwani; Liverpool School of Tropical Medicine; Malawi Liverpool Wellcome Trust Clinical Research Programme<br>Kelly, Christine; Malawi Liverpool Wellcome Trust Clinical Research Programme; University College Dublin<br>Huwa, Jacqueline; Malawi Liverpool Wellcome Trust Clinical Research Programme<br>Afran, Louise; Malawi Liverpool Wellcome Trust Clinical Research Programme<br>Tatuene, Joseph; University of liverpool, Institute of Infection and Global Health; Malawi-Liverpool-Wellcome Trust Clinical Research Programme,<br>Pett, Sarah; University College London, Institute of Infection and Global Health; University of New South Wales, Kirby Institute<br>Henrion, Marc; Malawi Liverpool Wellcome Trust Clinical Research Programme; Liverpool School of Tropical Medicine<br>Van Ososterhout, Joep; University of Malawi College of Medicine; Dignitas International<br>Heyderman, Robert; University College London, Division of Infection and Immunity; University of Malawi College of Medicine, Malawi Liverpool Wellcome Trust Clinical Research Programme<br>Mwandumba, Henry; Liverpool School of Tropical Medicine; Malawi Liverpool Wellcome Trust Clinical Research Programme<br>Benjamin, Laura; University of Liverpool Institute of Infection and Global Health, ; University College London Institute of Neurology,

Keywords:	Ischaemic heart disease < CARDIOLOGY, EPIDEMIOLOGY, HIV & AIDS < INFECTIOUS DISEASES, Stroke medicine < INTERNAL MEDICINE

SCHOLARONE™
Manuscripts

For peer review only - http://bmjopen.bmj.com/site/about/guidelines.xhtml

BMJ OPEN

Title: Protocol for a longitudinal cohort study to evaluate the reactivation of herpesviruses and inflammation as cardiovascular and cerebrovascular risk factors in antiretroviral therapy initiators, in an African HIV-infected population (RHICCA)

Authors:

Ingrid Peterson[1,2], Ntobeko Ntusi[3], Kondwani C Jambo[1,2], Christine Kelly[1,4], Jacqueline Huwa[1], Louise Afran[1], Joseph Kamtchum-Tatuene[5], Sarah Pett[6,7,8], Marc Henrion[1,2], Joep J van Oosterhout[9,10], Robert Heyderman[11], Henry C Mwandumba[1,2], Laura Benjamin[4,12**] and for the Investigators of the RHICCA study*

Affiliations:

1. Malawi-Liverpool-Wellcome Trust Clinical Research Programme, University of Malawi College of Medicine
2. Department of Clinical Sciences, Liverpool School of Tropical Medicine
3. Department of Medicine, University of Cape Town
4. HIV Molecular Research Group, University College Dublin
5. Institute of Infection and Global Health, University of Liverpool
6. Institute of Global Health, University College London
7. MRC CTU at UCL, Institute of Medicine, Clinical Trials and Methodology, University College London
8. Kirby Institute, University of New South Wales, Australia
9. Dignitas International, PO Box 071, Zomba, Malawi
10. College of Medicine, University of Malawi
11. Department of Infection and Immunity, University College London
12. Department of Brain Repair and Rehabilitation, Institute of Neurology, UCL

For peer review only - http://bmjopen.bmj.com/site/about/guidelines.xhtml

*The Investigators of the RHICCA study

Agbor Ako - Research and Development, GlaxoSmithKline

Brian Angus - Oxford Centre for Clinical Tropical Medicine, University of Oxford

Myles Connor - University of Edinburgh

Reena Dwivedi - Greater Manchester Neurosciences Centre, Salford Royal Foundation Trust

Lewis Haddow - Institute for Global Health, University College London

Terttu Heikinheimo-Connell - Hyvinkää Hospital, Department of Neurology, University of Helsinki

Elizabeth Joekes - Liverpool School of Tropical Medicine

Vanessa Kandoole - Department of Medicine, University of Malawi College of Medicine, Blantyre

Moffat Nyrienda - MRC Research Unit, Uganda

Kennedy Malisita- Department of Medicine, Queen Elizabeth Central Hospital

Jane Mallewa- Department of Medicine, University of Malawi College of Medicine, Blantyre

Elsayed Z. Soliman - School of Medicine, Wake Forest School of Medicine

Tom Solomon - Institute of Infection and Global Health, University of Liverpool

**Corresponding author

Laura Benjamin

Institute of Infection and Global Health,

Ronald Ross building,

The University of Liverpool,

L69 7BE, Liverpool,

For peer review only - http://bmjopen.bmj.com/site/about/guidelines.xhtml

BMJ OPEN

United Kingdom

l.benjamin@liverpool.ac.uk

Key words:

Cardiovascular, cerebrovascular, HIV, herpesvirus, Immune dysregulation,

Journal Guidance:

Abstract word count: 300/300

Article word count: 3995 /4000

Figure/Table: 5/5

For peer review only

ABSTRACT

Introduction: In Sub-Saharan Africa, rising rates of cerebrovascular and cardiovascular disease (CBD/CVD) are intersecting with an aging HIV-infected population. The widespread use of antiretroviral therapy (ART) may confer an additive risk and may not completely suppress the risk associated with HIV infection. High-quality prospective studies are needed to determine if HIV-infected patients in Africa are at increased risk of CBD/CVD and to identify factors associated with this risk. This study will test the hypothesis that immune activation and dysfunction, driven by HIV and reactivation of latent herpesvirus infections lead to increased CBD/CVD risk in Malawian adults aged $\geq$ 35 years.

Methods and Analysis: We will conduct a single-center 36-month prospective cohort study in 800 HIV-infected patients initiating antiretroviral therapy (ART) and 190 HIV-uninfected controls in Blantyre, Malawi. Patients and controls will be recruited from government ART clinics and the community, respectively and will be frequency-matched by 5-year age band and sex. At baseline and follow-up visits, we will measure carotid intima thickness (CIMT), pulse wave velocity (PWV) as surrogate markers of vasculopathy, and thus CBD/CVD risk. Our primary exposures of interest will be prospectively measured; these include cytomegalovirus and varicella zoster reactivation, changes in HIV plasma viral load, and markers of systemic inflammation and endothelial function. Multivariable regression models will be developed to assess the study's primary hypothesis. The occurrence of clinical CBD/CVD will be assessed as secondary study endpoints. ISRCTN registry https://doi.org/10.1186/ISRCTN42862937.

Ethics and dissemination: This was approved by the University of Malawi College of Medicine and the Liverpool School of Tropical Medicine research ethics committees. Our goal is to gain insight into the pathogenesis of cardiovascular and cerebrovascular disease among HIV cohorts on ART, in sub-Saharan Africa, and provide data to inform future interventional clinical trials. This study started in May 2017 and will continue until August 2020.

For peer review only

For peer review only - http://bmjopen.bmj.com/site/about/guidelines.xhtml

STRENGTHS AND LIMITATIONS

- This is one of the first large-scale studies in Sub-Saharan Africa to explore the relationship between HIV infection, latent herpesviruses, inflammation and cardiovascular and cerebrovascular diseases, immediately after starting antiretroviral therapy (ART).
- Clinical events and death will be comprehensively reviewed through an end-point review committee, using strict diagnostic criteria for events based on those used in the INSIGHT network, or validated verbal autopsy for death with limited data.
- Because of the recent roll-out of ART in asymptomatic patients, there will be an absence of ART-naïve population, limiting our ability to explore the impact of ART.
- Approximately one-third of strokes will be asymptomatic. We anticipate not capturing some of these. However, multiple cerebral infarcts without a focal neurological deficit will manifest as cognitive impairment, which we will screen for, and corroborate with MRI imaging in a small number of cases.
- Two-thirds of myocardial infarction will be silent and could potentially be missed. In a nested group, we will use a digital electrocardiogram to evaluate this further.

For peer review only - http://bmjopen.bmj.com/site/about/guidelines.xhtml

INTRODUCTION

The growing epidemic of cerebrovascular disease (CBD e.g. Stroke) and cardiovascular disease (CVD e.g. myocardial infarction) now intersects with the HIV epidemic[1]. Countries like Malawi, have an adult HIV prevalence of approximately 10%[2]. There is an increased life expectancy among people living with HIV, largely because of the successful scale-up of ART[3]. In Europe and the US, HIV is associated with a 50% increased risk of CVD compared to HIV-uninfected populations[4], attributable to long-term antiretroviral therapy (ART) use and HIV *per se*[4 5]. HIV infection is also associated with a 1.8 fold increased risk of all-cause heart failure in US veterans[6]. Our recent case-control study of stroke in Malawian adults is one of several examples that demonstrates a high risk of HIV infection associated with stroke and heart disease, pointing to a considerable and unappreciated CBD/CVD risk among HIV patients, in this setting[7-10].

There are reports of geographical differences in the distribution of CVD risk factors, supporting the argument that evidence derived from high-income countries cannot be applied to Sub-Saharan (SSA)[11]. Addressing this knowledge gap is essential to the development of clinical drug trials for primary prevention of CBD/CVD among individuals living with HIV. Vasculopathy due to accelerated atherosclerosis, arterial stiffening and vasculitis are the major mechanisms believed to underlie the CBD/CVD burden[12 13]. It is hypothesized that despite viral suppression, low-grade HIV virus replication and the associated host systemic inflammation are important drivers of this vasculopathy (Figure 1). In patients receiving ART, HIV antigenemia, partly resulting from HIV persistence in sanctuary sites, incomplete virologic suppression and virologic resurgence, drives the chronic immune activation observed in about 20% of ART patients in SSA[14]. This immune state is characterized by ongoing activation and senescence of cell-mediated immunity[15 16], increased monocyte/macrophage activation, stimulation of the interleukin-6 (IL-6) pathway and production of acute phase proteins[17-19]. Activation of the IL-6 pathway is established with atherosclerosis[20 21], and may also contribute to non-atherosclerotic vasculopathy. Inflammation alone confers a 2-fold increased risk of clinical CBD/CVD events[22]. The current push to introduce more effective ART regimens, and to start treatment soon after HIV diagnosis is made, may reduce inflammation and in turn, CBD/CVD risk[23]. However, there is

For peer review only - http://bmjopen.bmj.com/site/about/guidelines.xhtml

growing evidence of chronic inflammation in HIV despite achieving the goal of therapy, which is long-term suppression (<50 copies/mL) of plasma viral load, suggesting adjunctive therapy may be required.[24-26]

In addition to HIV, there is compelling evidence that reactivation of latent herpesviruses may be an important cause of vasculopathy. In HIV-uninfected elderly populations from high-income settings, latent cytomegalovirus (CMV) infection drives dysregulation of cell-mediated immunity[15 27-29], not dissimilar to what's described in HIV-associated immune activation[29]. CMV and other viral proteins have been found in atherosclerotic plaques[20]. Varicella-zoster virus (VZV) can directly infect the vascular endothelium to cause vasculitis and subsequent stroke and was found to be the commonest opportunistic infection (prevalence 15%) in a study of HIV-infected stroke patients in Malawi [12]. The seroprevalence of herpesviruses is high in SSA[30], particularly in HIV-infected populations[16].

The involvement of herpesviruses in the mechanistic pathway for CBD/CVD is compelling and may offer additional therapeutic avenues, especially for CMV and VZV. However, our understanding is incomplete, and its population impact is yet to be defined. It is important to determine if, in addition to ART, there is a role for other pharmacological interventions targeting latent viral infections or downstream inflammatory pathways to reduce vasculopathy in HIV-infected patients on ART. Previous work from North America supports the potential of treating reactivated herpesviruses[31]. Furthermore, there are opportunities for intervention using the recently licensed Letermovir; a treatment for CMV. By focusing on HIV and Herpes viral antigenemia and immune dysregulation as mechanisms of vasculopathy, this study will identify subgroups of HIV-infected patients on ART at high risk of CBD/CVD, the timing of CBD/CVD risk in such patients, as well as potential targets for intervention.

For peer review only - http://bmjopen.bmj.com/site/about/guidelines.xhtml

STUDY OBJECTIVES

This study will test the hypothesis that immune activation and dysfunction, driven by HIV and reactivation of latent CMV/VZV herpesvirus infections lead to increased CBD/CVD risk in adults aged ≥ 35 years in SSA. We will address this through the following objectives;

1) To determine if progression of the surrogate marker of CBD/CVD or occurrence of new-onset vasculopathy is higher in adults aged ≥ 35 years with HIV infection on ART compared to those without HIV.
2) To determine if progression of surrogate markers of CBD/CVD or occurrence of new-onset vasculopathy is higher in adults aged ≥ 35 years with HIV/herpes viral antigenemia or chronic immune activation compared to those without HIV/herpes viral antigenemia or chronic immune activation. Specifically, we will determine if progression of surrogate markers or new-onset vasculopathy is higher:
    a. in ART patients with reactivated latent herpes viral infection, compared to those without reactivated latent herpes viral infection.
    b. in ART patients with the highest 25% of markers for immune activation, inflammation or endothelial activation compared to the bottom 25%
    c. in ART patients with incomplete virologic suppression or virologic resurgence of HIV, compared to those with suppressed HIV plasma viral load.

The secondary study objectives are to determine if viral antigenemia or chronic immune activation increase occurrence of the following clinical events: 1) stroke, 2) myocardial infarction (MI), 3) angina (excluding MI), 4) peripheral vascular disease (PVD), 5) all-cause death/vascular-related death and 6) immune reconstitution inflammatory vasculopathy.

METHODS AND ANALYSIS

<u>Study design</u>

To address objective 1, we will conduct a single-center 36-month prospective cohort study in 800 HIV-infected patients initiating ART and 190 HIV-uninfected adults aged ≥ 35 years. HIV-infected and HIV-uninfected participants will be frequency matched by 5-year age band and sex. On a 6-monthly basis, we will measure markers of viral infection, inflammation and endothelial function along with surrogate markers for CBD/CVD (Figure 2).

For peer review only - http://bmjopen.bmj.com/site/about/guidelines.xhtml

Study Setting

This study will recruit consecutive ART patients from the ART clinic of Queen Elizabeth Central Hospital (QECH), and ART clinics in several Blantyre City Community Health Centres (CHCs). These clinics collectively initiate over 100 HIV-infected patients aged ≥ 35 years onto ART each month. HIV-uninfected adults will be selected from pre-ART counseling sessions, and from randomly selected households in the community by two-stage random sampling (of households and individuals within households) from a previously enumerated sampling frame in the CHC catchment areas[32]. All study procedures will be conducted at QECH, which is located adjacent to the Malawi-Liverpool-Wellcome Trust Clinical Research Programme (MLW). QECH also hosts a 0.35T MRI imaging facility, which will contribute to characterizing our secondary endpoints.

Study Participants

Study inclusion criteria will be: a) age ≥ 35 years and b) resident in Blantyre. HIV-infected patients must further be: c) ART-naïve or initiated ART <10 days prior to enrolment and d) initiating standard first-line ART (in Malawi this is: Tenofovir [TDF]/Lamivudine [3TC]/Efavirenz [EFV]). Adult controls must further be: e) HIV-uninfected. Study exclusion criteria are: f) clinical history of CBD/CVD, g) pregnancy, h) critical illness or symptomatic anemia at baseline and i) enrollment in an intervention study.

Justification of study inclusion and exclusion criteria is as follows; in many populations, CBD/CVD risk rises sharply from 35-years of age[33], thus individuals aged 35 and older will be eligible (recruitment of participants aged 35 -39 will be limited to 15% of the study sample to avoid overrepresentation). Restricting recruitment by age will enable this study to have greater statistical power. For clarity of etiologic inference, the study will assess the risk of new-onset vasculopathy not associated with pregnancy and thus exclude patients who are pregnant or with a history of CBD/CVD. To eliminate confounding by ART regimen, patients must initiate on standard first-line ART (> 90% of ART patients in Blantyre do this). Critically ill patients are excluded primarily for ethical reasons.

For peer review only - http://bmjopen.bmj.com/site/about/guidelines.xhtml

Laboratory methods

Surface immunophenotyping of peripheral blood mononuclear cells

Immunophenotyping will be used to characterize peripheral blood mononuclear cells (PBMC) isolated from blood samples of HIV-uninfected and HIV-infected ART initiators. PBMCs will be harvested by density centrifugation using lymphoprep (Axis Shield, UK). PBMCs ($2x10^6$) will be stained with anti-CD45 PerCP CY5.5, anti-CD3 AF700, anti-CD4 BV421, anti-CD8 PE Dazzle, anti-CD38 BV605, anti-HLA-DR APC CY7, anti-CD57 APC, anti-PD1 PE CY7, anti-CTLA4 PE, and anti-CD223 FITC (all from eBiosciences, UK) to determine the expression of these markers on the surface of T-cells. In addition, ($2x10^6$) PBMCs stained with anti-CD16 BV421, anti-CD14 PE, anti-HLA-DR PerCP CY5.5, anti-CD45 AF700, anti-CCR2 BV605, anti-CD11b APC, anti-CX3CR1 PE Dazzle and anti-CD38 FITC (all from eBiosciences, UK) will be used for monocytes. Dead cells, $CD3^+$ T-cells, and $CD56^+$ NK cells will be excluded using: LIVE/DEAD™ Fixable Aqua Dead Cell Stain (Thermofisher, UK), anti-CD3 BV503 and anti-CD56 BV503 (eBiosciences, UK), respectively. Stained cells will be acquired on a BD LSR Fortessa flow cytometer (Becton Dickinson, USA) and data will be analyzed using FlowJo software version 10.0 (Tree Star, San Carlos, CA). For each stained sample analyzed, the median fluorescence intensity (MFI) for each parameter will be normalized to its respective unstained control.

Measurement of soluble markers of immune activation using multiplex bead array

A custom made multiplex assay will be used to assess soluble markers of monocyte activation (CD163), systemic inflammation (Interleukin-6) and endothelial activation (Intracellular adhesion molecule 1) in plasma, isolated from blood samples of HIV-uninfected and HIV-infected ART initiators. Following isolation, plasma will be aliquoted and stored at -80°C until further use.

Assessment of exposure to human cytomegalovirus and varicella zoster virus by ELISA

Quantitative VIDAS CMV IgG and IgM (BioMerieux, USA) and VZV glycoprotein IgG Low-Level Enzyme Immunoassay Kit [VaccZyme™EIA], will be used to determine exposure to these

For peer review only - http://bmjopen.bmj.com/site/about/guidelines.xhtml

viruses using a commercial enzyme-linked immunosorbent assay (ELISA) platform. These kits will detect VZV antigen to a sensitivity and specificity of 97.8% and 96.8% respectively and for CMV, 97.2% and 100% for IgG and 100% and 97.4% for IgM respectively[34 35]. Plasma samples from HIV-uninfected and HIV-infected ART initiators stored at -80°C following collection will be used for these assessments

HIV

HIV infection will be diagnosed using two rapid tests in parallel, EIA rapid tests (Determine HIV-1/2 [Abbott Laboratories, USA] and Uni-Gold HIV [Trinity Biotech PLC, Ireland]), will be used as a tiebreak). HIV-1 RNA levels in plasma will be measured using the Abbott Real-Time HIV-1 assay with a lower limit of detection of 150 copies/mL (Abbott Molecular, Germany), according to the manufacturer's instructions. CD4+ T -cell count measurements will be performed using BD FACS Count machine (Partec platform).

<u>Procedures</u>

Carotid-femoral pulse wave velocity (PWV)[36] and carotid intima-media thickness (CIMT)[37] measurement will be performed in accordance with expert consensus guidelines, using a standardized study protocol on the Vicorder system (SMART Medical, UK) and Philips CX50 machine (Philips healthcare, UK) respectively. CIMT measurements will be performed by three trained operators. The intra-class correlation coefficient will be used to assess the performance of the operators against that of a certified neurosonologist prior to study commencement.

<u>Outcomes</u>

Primary outcomes

Primary outcomes are the progression of surrogate markers of CBD/CVD, namely PWV and CIMT as well as the occurrence of new-onset vasculopathy defined by threshold values outlined in Table 1.

For peer review only - http://bmjopen.bmj.com/site/about/guidelines.xhtml

BMJ OPEN

For peer review only

For peer review only - http://bmjopen.bmj.com/site/about/guidelines.xhtml

Secondary outcomes

Secondary outcomes are the following clinical events: 1) stroke, 2) myocardial infarction (MI), 3) unstable angina, 4) peripheral vascular disease (PVD), 5) all-cause death/ vascular death and 6) immune reconstitution inflammatory syndrome (IRIS) vasculopathy (Table 1). Changes in PWV or endothelial activation at 6 months post ART initiation will be interpreted as a subclinical vascular IRIS event. These outcomes will be assessed through active surveillance in QECH inpatient wards for admissions of study participants. To improve capture of clinical outcomes, we will conduct brief telephone interviews with study participants about CBD/CVD symptoms and hospitalizations between study visits and facilitate unsolicited participant self-report. Clinical events and deaths in study participants will be reviewed by an independent endpoint review committee (ERC), comprising of clinicians experienced in Endpoint review. Each event will be reviewed and adjudicated by the ERC Chair and 2 ERC reviewers, using a standard set of diagnostic criteria (Table 1 and Supplement – S1). The format of reporting will be based on modifications of the INSIGHT network clinical diagnostic criteria. Deaths will be reviewed by the ERC using the CoDe approach[23]. For death with limited clinical data, a validated verbal autopsy will be performed to ascertain the cause[38].

Exposures

The exposure for Primary Objective 1 will be HIV status. Yearly HIV rapid tests in HIV-uninfected adults will be performed to exclude those with new HIV infections (Figure 2).

Potential confounding and mediating factors will be recorded in study participants. This will include demographic factors, lifestyle and behavioral factors (e.g. cigarette smoking and alcohol consumption), chronic co-morbidities (i.e. hypertension, diabetes), cardiometabolic, renal and hematological factors (i.e. full blood count, creatinine in urine and serum, body-mass-index, waist-to-hip ratio, random glucose, HbA1c, and lipid profile). Blood pressure will be measured at all study visits. Although vascular immune reconstitution inflammatory syndrome (IRIS) (Table 1) will be considered as a primary endpoint, non-vascular IRIS will be defined as a risk factor. Where feasible, we will conduct PCR tests for common causes of IRIS in blood or cerebrospinal fluid (CSF) samples. Adherence to ART and change of ART regimen

For peer review only - http://bmjopen.bmj.com/site/about/guidelines.xhtml

will be assessed at all study visits through extraction of data from 'ART master cards'; this is a government-supported monitoring tool used by all patients on ART, in Malawi.

For Objective 2a-2c, markers of herpes and HIV viral antigenemia and immune inflammation will be measured according to the outline in Table 2. For primary objective 2a, reactivated latent herpes viral infections will be assessed by quantification of VZV, and CMV antibodies. We will estimate the risk of atherosclerosis and arterial stiffening associated with current herpesviruses reactivation at baseline, and sustained reactivation (i.e. those that continue to have a high titer from measurement at baseline to 6 months after ART initiation). Hyperactivation of B cells may result in an expansion of polyclonal antibodies and thus an overestimation of virus-specific antibody titers. To address this issue and make appropriate adjustments for hypergammaglobulinemia we will 1) measure more than one herpesviruses and 2) measure total IgG.

For primary objective 2b, markers of immune activation, inflammation, and endothelial activation will be measured (Figures 1 & 3). Quantitative cell surface immunophenotyping will be performed for CD4+ and CD8+ T-cell activation (e.g. HLA-DR) and senescence (e.g. CD57) in a subset of participants. In all study participants, at baseline, 6, 12, months, we will measure soluble markers associated with systemic inflammation and endothelial activation.

For primary objective 2c, incomplete viral response and viral rebound of HIV will be measured by quantitative PCR in patients on ART.[39] HIV viral load will be measured in patients on ART at 0, 6 and 12 months.

<u>Data Collection Between May 2017 and August 2020</u>

The two-stage screening will be conducted to find and recruit potential study participants. A trained field worker will first screen to assess eligibility for criteria (a)-(c) in pre-ART counseling sessions, and in individuals from randomly selected households in the community. Eligible participants will then be referred to QECH to complete screening for criteria (d)-(i) and if eligible, consented to participate in the study. At study visits, a tablet-

For peer review only - http://bmjopen.bmj.com/site/about/guidelines.xhtml

based, standardized Open Data Kit (ODK) case report form (CRF) will be administered in one-on-one interviews by a study nurse to capture demographic and clinical data. Study data will be collected as outlined in Table 2. Daily upload of electronic data will occur with oversight from the data manager at MLW. We will collect up to 30ml of whole blood. An ACR dipstick test will be used to test for creatinine, proteinuria, and glucosuria. In a subset of participants, an electrocardiogram supported by a digital platform and echocardiogram will be performed at baseline, 6 and 24 months, as well as in any participant experiencing a clinical event suggestive of a cardiac etiology. To facilitate the retention and clinical referrals of participants, contact will be made every 3 months to assess the occurrence of clinical events. Participants who miss a scheduled study visit will be contacted by phone and/or visited at home to assess their willingness to maintain their participation and to record intervening clinical events. Recording and definitions of other clinical events, including HIV associated events will be evaluated by the ERC chair. SMS messages will be used for appointment reminders. Technical appendix, statistical code, and dataset will be made available from a data repository, after publication of our work.

Sample Size and Statistical analysis

The required sample size for the study's primary objectives is 800 HIV-infected patients and 190 HIV-uninfected adults using standard, normal distribution approximation sample size formulas for comparing proportions in two groups of unequal size and based on the following assumptions: **a)** 18.4% of study participants have abnormal PWV at baseline. In our ongoing studies of vasculopathy in HIV-infected patients, 18.4% aged $\geq$35 years have a PWV (>12 m/s), **b)** 20% of both HIV-infected patients and HIV-uninfected adults will be lost to follow-up, including by death and HIV sero-conversion[40 41]. **c)** The minimum relative risk (RR) of interest is 2 for Objective 1 and 1.8 for Objective 2. **d)** Cumulative risk of clinically significant vasculopathy over study follow-up is 18.4%. This is based on study data cited in (a). **e)** For objectives 2a)-c)**,** the exposure prevalence for each risk factor is 20%. **f)** Statistical tests will have 80% power based on a 2-sided test with; α=0.05. Testing of hypotheses for the secondary outcome will be exploratory. However, we estimate 26 strokes (4 mimics), an unknown number of MIs and 80 deaths occurring during the study[7 42].

For peer review only - http://bmjopen.bmj.com/site/about/guidelines.xhtml

The reporting of this study will be prepared in accordance with the STROBE guidelines[43]. Summary and descriptive statistics will be tabulated for all primary and secondary outcome variables, as well as for exposure variables and potential confounding or mediating factors. Time plots for all outcome variables will be inspected. Quantitative data analysis will be conducted to assess the primary outcomes. The statistical analyses will slightly differ for the interim and the final analysis. While we will develop models that adjust for potentially confounding or mediating variables, we will also perform unadjusted analyses. At the interim analysis, the latter will be performed using t-tests or Wilcoxon signed rank tests (depending on whether the data are normally distributed or not) for PWV and Fisher's exact test for new-onset vasculopathy.

For primary objective 1, we will develop a total of 2 regression models at the interim analysis. A generalized linear regression model (GLM) will compare mean progression of arterial damage from baseline in HIV-infected patients and HIV-uninfected adults. This model will regress change from baseline in PWV on HIV status. A log link function may be used if required to satisfy model assumptions, otherwise, an identity link function will be used. We will develop a second model to estimate the risk ratio (RR) and population attributable fraction of new-onset vasculopathy in HIV-infected patients compared to HIV-uninfected adults. This will be a logistic model which regresses a binary factor for new-onset vasculopathy on HIV status in all participants without new-onset vasculopathy at baseline. In the final analysis, we will repeat the analysis done at the interim stage but with the outcome at 24 months and develop another GLM, using CIMT as the response. Furthermore, we will extend the two GLM to linear mixed model (LMM) to account for the correlation in the data due to the repeated measurements for everyone. If a log link is necessary for the GLMs to satisfy model assumptions, we will develop marginal models using generalized estimating equations (GEEs) instead of the LMMs. In addition to the logistic regression models, differences in risk of new-onset vasculopathy between HIV-infected and HIV-uninfected adults will be assessed using time-to-event models adjusting for time-varying covariates and interval-censored outcomes as appropriate.

For primary objective 2a, we will develop regression models to assess if progression of PWV and CIMT or occurrence of new-onset vasculopathy is higher in HIV-infected patients with a

For peer review only - http://bmjopen.bmj.com/site/about/guidelines.xhtml

high viral burden. At the interim analysis, aGLM will be developed to compare mean progression of PWV in HIV-infected patients with and without reactivated latent herpes viral infection. This model will regress change from baseline in PWV on three log-transformed variables for antibody titer of CMV, and VZV. A logistic model will be used to estimate the RR and attributable fraction of new-onset vasculopathy in HIV-infected patients (without new-onset vasculopathy at baseline) with reactivated latent herpes viral infection compared to those without reactivated latent herpes infection. This model will regress a binary factor for new-onset vasculopathy on the two binary variables for herpesvirus reactivation at 0 and 6 months. For the final analysis, the GLMs will be extended to LMMs (especially GEEs, if a log link is required) and risk of new-onset vasculopathy in the two groups will be assessed using time-to-event models.

To assess if progression of PWV and CIMT is higher in HIV-infected patients with immune activation (primary objective 2b) we will develop, at the interim analysis, a GLM comparing mean progression of PWV in HIV-infected patients and levels of immune and inflammation markers. Initially, one model will be run for each marker, by regressing change in PWV on marker quantile in all HIV-infected patients. We will then work to develop a comprehensive model with multiple markers that are not highly correlated with one another. At final analysis, this will be repeated for both PWV and CIMT, with outcomes at 24 months. We will also again use LMMs or GEEs to make full use of the longitudinal nature of the data at final analysis.

Modeling for primary objective 2c will be conducted in a similar fashion as described for primary objective 2a and 2b, by regressing factors for progression of vasculopathy from baseline and new onset vasculopathy on binary factors for incomplete viral response and viral resurgence. All models for primary objectives will include potential confounding variables and time of follow-up since baseline. Other modeling approaches will be used to examine important questions answerable by study data. For example, the time-to-event data models that will also be used to identify the time point of greatest vasculopathy risk in ART patients.

For peer review only - http://bmjopen.bmj.com/site/about/guidelines.xhtml

For secondary study objectives, we will use univariate methods to assess the frequency of clinical events within exposure strata. If there is a sufficient number of clinical events we will develop logistic regression models for each clinical event type like primary objectives 1 and 2. We will also analyze a binary variable for the occurrence of any of the study's secondary outcomes, versus no such occurrence. As part of exploratory analyses, we will also aim to identify risk groups that are potentially incompletely captured with the measured exposure variables. We will perform unsupervised group-based multi-trajectory modeling of multivariate longitudinal patient trajectories to confirm any associations we have found using more traditional approaches[44].

ETHICS AND DISSEMINATION

Written informed consent will be obtained from all study participants, either written or witnessed verbal consent with thumbprint if the participant is non-literate. Study data will be maintained in an encrypted and password protected database to which only study staff will have access. Study participants who develop a clinical event will be managed, using the hospital guidelines, by our study clinician alongside the hospital doctor. Clinical data will be anonymized using unique identifying code. Study data will be kept for 10 years and then destroyed with a record, as recommended by good clinical practice guidelines. This protocol was approved by the ethics committees at University of Malawi College of Medicine (Protocol P02/16/1874) and the Liverpool School of Tropical Medicine (Protocol 16-014).

DISCUSSION

African regions continue to bear the brunt of HIV infection, in 2013, an estimated 8.5 million adults were receiving ART[45]. As the landscape evolves, this population will live longer with stable HIV infection but likely remain at an increased risk of CBD/CVD compared to HIV-uninfected individuals of a similar age and sex. This study will be the first to determine the extent to which HIV reactivation of herpesvirus infection and inflammation contribute to CBD/CVD risk in an adult African population starting ART. The results of this work could

For peer review only - http://bmjopen.bmj.com/site/about/guidelines.xhtml

potentially open avenues for novel anti-inflammatory and anti-viral interventions for the primary prevention of CBD/CVD in HIV populations in Africa.

Acknowledgments

The authors would like to thank the NCD Africa Open Lab of GlaxoSmithKline review committee for providing valuable advice for this protocol and the INSIGHT network for sharing their clinical endpoint criteria. LB is supported by an NIHR Clinical Lecturer Fellowship. SLP is supported by an MRC (UK) core funding MC_UU_12023/23.

For peer review only

AUTHORS' CONTRIBUTIONS

LB and IP developed the first draft. HM, NT, KJ, CK had major input for the revision of the second draft. All other authors subsequently contributed to the review of the manuscript.

FUNDING STATEMENT

Funding for this study was provided by the GlaxoSmithKline Africa Non-Communicable Disease Open Lab Grant (Project Number: 7964)

COMPETING INTERESTS

SLP has academic grants from Sysmex Corporation, Gilead Sciences, and ViiV Healthcare. All other authors have no competing interest.

For peer review only - http://bmjopen.bmj.com/site/about/guidelines.xhtml

REFERENCES

1. Roth GA, Johnson C, Abajobir A, et al. Global, Regional, and National Burden of Cardiovascular Diseases for 10 Causes, 1990 to 2015. *J Am Coll Cardiol* 2017;70(1):1-25. doi: 10.1016/j.jacc.2017.04.052
2. Organization WH. Global Update on HIV Treatment 2013: Results, Impact and Opportunities. WHO Report. Kuala Lumpure, Malaysia, 2013.
3. Macro NSONaI. Malawi Demographic and Health Survey 2010. Zomba,Malawi, and Calverton, Maryland, USA: NSO and ICF Macro, 2010.
4. Freiberg MS, Chang CC, Kuller LH, et al. HIV infection and the risk of acute myocardial infarction. *JAMA Intern Med* 2013;173(8):614-22. doi: 10.1001/jamainternmed.2013.3728

1659742 [pii] [published Online First: 2013/03/06]
5. Currier JS, Lundgren JD, Carr A, et al. Epidemiological evidence for cardiovascular disease in HIV-infected patients and relationship to highly active antiretroviral therapy. *Circulation* 2008;118(2):e29-35. doi: 10.1161/CIRCULATIONAHA.107.189624 [published Online First: 2008/06/21]
6. Butt AA, Chang CC, Kuller L, et al. Risk of heart failure with human immunodeficiency virus in the absence of prior diagnosis of coronary heart disease. *Archives of internal medicine* 2011;171(8):737-43. doi: 10.1001/archinternmed.2011.151 [published Online First: 2011/04/27]
7. Benjamin LA, Corbett EL, Connor MD, et al. HIV, antiretroviral treatment, hypertension, and stroke in Malawian adults: A case-control study. *Neurology* 2016;86(4):324-33. doi: 10.1212/WNL.0000000000002278
8. Allain TJ, Kinley L, Tsidya B, et al. The spectrum of heart disease in adults in Malawi: A review of the literature with reference to the importance of echocardiography as a diagnostic modality. *Malawi Med J* 2016;28(2):61-65. [published Online First: 2016/11/30]
9. Soliman EZ, Juma H. Cardiac disease patterns in northern Malawi: epidemiologic transition perspective. *J Epidemiol* 2008;18(5):204-8. [published Online First: 2008/08/30]
10. Syed FF, Sani MU. Recent advances in HIV-associated cardiovascular diseases in Africa. *Heart* 2013;99(16):1146-53. doi: 10.1136/heartjnl-2012-303177 [published Online First: 2013/05/18]
11. Soliman EZ, Sharma S, Arasteh K, et al. Baseline cardiovascular risk in the INSIGHT Strategic Timing of AntiRetroviral Treatment (START) trial. *HIV Med* 2015;16 Suppl 1:46-54. doi: 10.1111/hiv.12233 [published Online First: 2015/02/26]
12. Benjamin LA, Allain TJ, Mzinganjira H, et al. The Role of Human Immunodeficiency Virus-Associated Vasculopathy in the Etiology of Stroke. *J Infect Dis* 2017;216(5):545-53. doi: 10.1093/infdis/jix340 [published Online First: 2017/09/22]
13. Benjamin LA, Bryer A, Lucas S, et al. Arterial ischemic stroke in HIV: Defining and classifying etiology for research studies. *Neurol Neuroimmunol Neuroinflamm* 2016;3(4):e254. doi: 10.1212/NXI.0000000000000254
14. Nakanjako D, Kiragga A, Ibrahim F, et al. Sub-optimal CD4 reconstitution despite viral suppression in an urban cohort on antiretroviral therapy (ART) in sub-Saharan Africa: frequency and clinical significance. *AIDS Res Ther* 2008;5:23. doi: 10.1186/1742-6405-5-23 [published Online First: 2008/10/30]
15. Pawelec G, Derhovanessian E. Role of CMV in immune senescence. *Virus Res* 2011;157(2):175-9. doi: 10.1016/j.virusres.2010.09.010 [published Online First: 2010/09/28]
16. Appay V, Sauce D. Immune activation and inflammation in HIV-1 infection: causes and consequences. *J Pathol* 2008;214(2):231-41. doi: 10.1002/path.2276 [published Online First: 2007/12/29]
17. Shaw AC, Joshi S, Greenwood H, et al. Aging of the innate immune system. *Curr Opin Immunol* 2010;22(4):507-13. doi: 10.1016/j.coi.2010.05.003 [published Online First: 2010/07/30]

For peer review only - http://bmjopen.bmj.com/site/about/guidelines.xhtml

18. Hearps AC, Angelovich TA, Jaworowski A, et al. HIV infection and aging of the innate immune system. *Sex Health* 2011;8(4):453-64. doi: 10.1071/SH11028 [published Online First: 2011/12/01]
19. Kovacs EJ, Palmer JL, Fortin CF, et al. Aging and innate immunity in the mouse: impact of intrinsic and extrinsic factors. *Trends Immunol* 2009;30(7):319-24. doi: 10.1016/j.it.2009.03.012 [published Online First: 2009/06/23]
20. Hansson GK. Inflammation, atherosclerosis, and coronary artery disease. *The New England journal of medicine* 2005;352(16):1685-95. doi: 10.1056/NEJMra043430 [published Online First: 2005/04/22]
21. Boekholdt SM, Stroes ES. The interleukin-6 pathway and atherosclerosis. *Lancet* 2012;379(9822):1176-8. doi: 10.1016/S0140-6736(12)60361-4 [published Online First: 2012/03/17]
22. Duprez DA, Neuhaus J, Kuller LH, et al. Inflammation, coagulation and cardiovascular disease in HIV-infected individuals. *PloS one* 2012;7(9):e44454. doi: 10.1371/journal.pone.0044454 [published Online First: 2012/09/13]
23. Lundgren JD, Babiker AG, Gordin F, et al. Initiation of Antiretroviral Therapy in Early Asymptomatic HIV Infection. *N Engl J Med* 2015;373(9):795-807. doi: 10.1056/NEJMoa1506816 [published Online First: 2015/07/21]
24. Eggena MP, Barugahare B, Okello M, et al. T cell activation in HIV-seropositive Ugandans: differential associations with viral load, CD4+ T cell depletion, and coinfection. *The Journal of infectious diseases* 2005;191(5):694-701. doi: 10.1086/427516 [published Online First: 2005/02/03]
25. Mussini CL, P.; Cozzi-Lepri,A.; Lapadula,G.; Marchetti,G.; Nicastri,E.; Cingolani,A.; Lichtner,M.;Antinori,A.; Gori,A.; Monforte, A. CD4/CD8 ratio normalisation and non-AIDS-related events in individuals with HIV who achieve viral load suppression with antiretroviral therapy: an observational cohort study. *Lancet HIV* 2015;2:e98–e106.
26. Sereti I, Krebs SJ, Phanuphak N, et al. Persistent, Albeit Reduced, Chronic Inflammation in Persons Starting Antiretroviral Therapy in Acute HIV Infection. *Clin Infect Dis* 2017;64(2):124-31. doi: 10.1093/cid/ciw683 [published Online First: 2016/10/16]
27. Brunner S, Herndler-Brandstetter D, Weinberger B, et al. Persistent viral infections and immune aging. *Ageing Res Rev* 2011;10(3):362-9. doi: 10.1016/j.arr.2010.08.003 [published Online First: 2010/08/24]
28. Moss P. The emerging role of cytomegalovirus in driving immune senescence: a novel therapeutic opportunity for improving health in the elderly. *Curr Opin Immunol* 2010;22(4):529-34. doi: 10.1016/j.coi.2010.07.001 [published Online First: 2010/08/06]
29. Appay V, Rowland-Jones SL. Premature ageing of the immune system: the cause of AIDS? *Trends Immunol* 2002;23(12):580-5. [published Online First: 2002/12/05]
30. Schaftenaar E, Verjans GM, Getu S, et al. High seroprevalence of human herpesviruses in HIV-infected individuals attending primary healthcare facilities in rural South Africa. *PloS one* 2014;9(6):e99243. doi: 10.1371/journal.pone.0099243 [published Online First: 2014/06/11]
31. Hunt PW, Martin JN, Sinclair E, et al. Valganciclovir reduces T cell activation in HIV-infected individuals with incomplete CD4+ T cell recovery on antiretroviral therapy. *J Infect Dis* 2011;203(10):1474-83. doi: 10.1093/infdis/jir060 [published Online First: 2011/04/20]
32. Corbett EL. Intensified HIV/TB prevention linking home-based HIV testing, including the option of selftesting, with HIV care. ISRCTN02004005. London: ISRCTN, 2012.
33. Roth GA, Huffman MD, Moran AE, et al. Global and regional patterns in cardiovascular mortality from 1990 to 2013. *Circulation* 2015;132(17):1667-78. doi: 10.1161/CIRCULATIONAHA.114.008720
34. Maple PA, Breuer J, Quinlivan M, et al. Comparison of a commercial Varicella Zoster glycoprotein IgG enzyme immunoassay with a reference time resolved fluorescence immunoassay (VZV TRFIA) for measuring VZV IgG in sera from pregnant women, sera sent for confirmatory

For peer review only - http://bmjopen.bmj.com/site/about/guidelines.xhtml

testing and pre and post vOka vaccination sera from healthcare workers. *J Clin Virol* 2012;53(3):201-7. doi: 10.1016/j.jcv.2011.12.010 [published Online First: 2012/01/21]
35. Carlier P, Harika N, Bailly R, et al. Laboratory evaluation of the new Access (R) cytomegalovirus immunoglobulin IgM and IgG assays. *J Clin Virol* 2010;49(3):192-7. doi: 10.1016/j.jcv.2010.07.024 [published Online First: 2010/08/31]
36. Laurent S, Cockcroft J, Van Bortel L, et al. Expert consensus document on arterial stiffness: methodological issues and clinical applications. *Eur Heart J* 2006;27(21):2588-605. doi: 10.1093/eurheartj/ehl254 [published Online First: 2006/09/27]
37. Touboul PJ, Hennerici MG, Meairs S, et al. Mannheim carotid intima-media thickness and plaque consensus (2004-2006-2011). An update on behalf of the advisory board of the 3rd, 4th and 5th watching the risk symposia, at the 13th, 15th and 20th European Stroke Conferences, Mannheim, Germany, 2004, Brussels, Belgium, 2006, and Hamburg, Germany, 2011. *Cerebrovasc Dis* 2012;34(4):290-6. doi: 10.1159/000343145

000343145 [pii] [published Online First: 2012/11/07]
38. Serina P, Riley I, Stewart A, et al. Improving performance of the Tariff Method for assigning causes of death to verbal autopsies. *BMC Med* 2015;13:291. doi: 10.1186/s12916-015-0527-9 [published Online First: 2015/12/09]
39. Organization WH. Consolidated ARV guidelines 2013 [Available from: http://www.who.int/hiv/pub/guidelines/arv2013/art/artmonitoring/en/index4.html accessed 15 Oct 2015.
40. Misiri HE, Edriss A, Aalen OO, et al. Estimation of HIV incidence in Malawi from cross-sectional population-based sero-prevalence data. *Journal of the International AIDS Society* 2012;15(1):14. doi: 10.1186/1758-2652-15-14 [published Online First: 2012/03/16]
41. MacPherson P, Houben RM, Glynn JR, et al. Pre-treatment loss to follow-up in tuberculosis patients in low- and lower-middle-income countries and high-burden countries: a systematic review and meta-analysis. *Bull World Health Organ* 2014;92(2):126-38. doi: 10.2471/BLT.13.124800 [published Online First: 2014/03/14]
42. Walker R, Whiting D, Unwin N, et al. Stroke incidence in rural and urban Tanzania: a prospective, community-based study. *Lancet Neurol* 2010;9(8):786-92. doi: S1474-4422(10)70144-7 [pii]

10.1016/S1474-4422(10)70144-7 [published Online First: 2010/07/09]
43. von Elm E, Altman DG, Egger M, et al. The Strengthening the Reporting of Observational Studies in Epidemiology (STROBE) Statement: guidelines for reporting observational studies. *International journal of surgery* 2014;12(12):1495-9. doi: 10.1016/j.ijsu.2014.07.013 [published Online First: 2014/07/22]
44. Nagin DS, Jones BL, Passos VL, et al. Group-based multi-trajectory modeling. *Stat Methods Med Res* 2018;27(7):2015-23. doi: 10.1177/0962280216673085 [published Online First: 2018/05/31]
45. Organization WH. Global Update on the Health Sector Response to HIV 2014. Geneva: World Health Organization, 2014.

For peer review only - http://bmjopen.bmj.com/site/about/guidelines.xhtml

BMJ OPEN

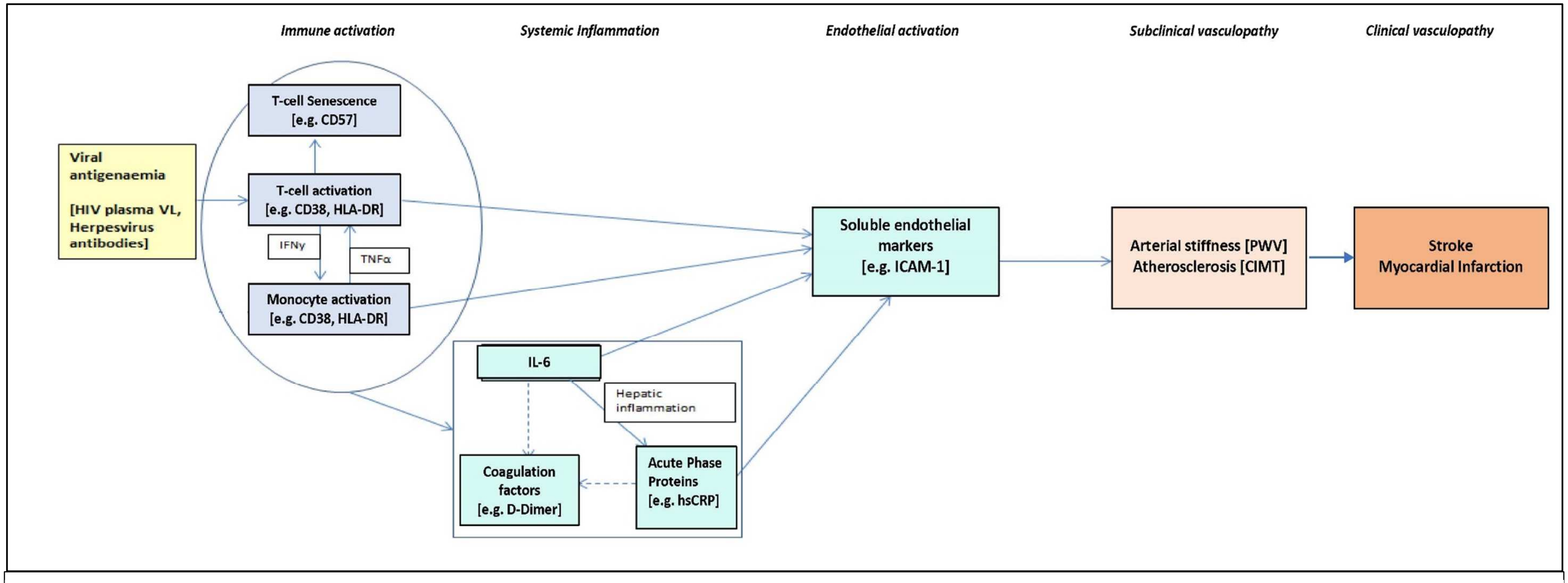

Figure 1. Hypothetical pathway of the interplay between chronic viruses, immune activation, systemic inflammation, endothelial activation, and vasculopathy.

KEY: VL- viral load, IL-6 – Interleukin 6, hsCRP – highly sensitive CRP, ICAM-1 – intracellular cell adhesion molecule 1, PWV – pulse wave velocity, CIMT – Carotid intimal medial thickness.

For peer review only - http://bmjopen.bmj.com/site/about/guidelines.xhtml

BMJ OPEN

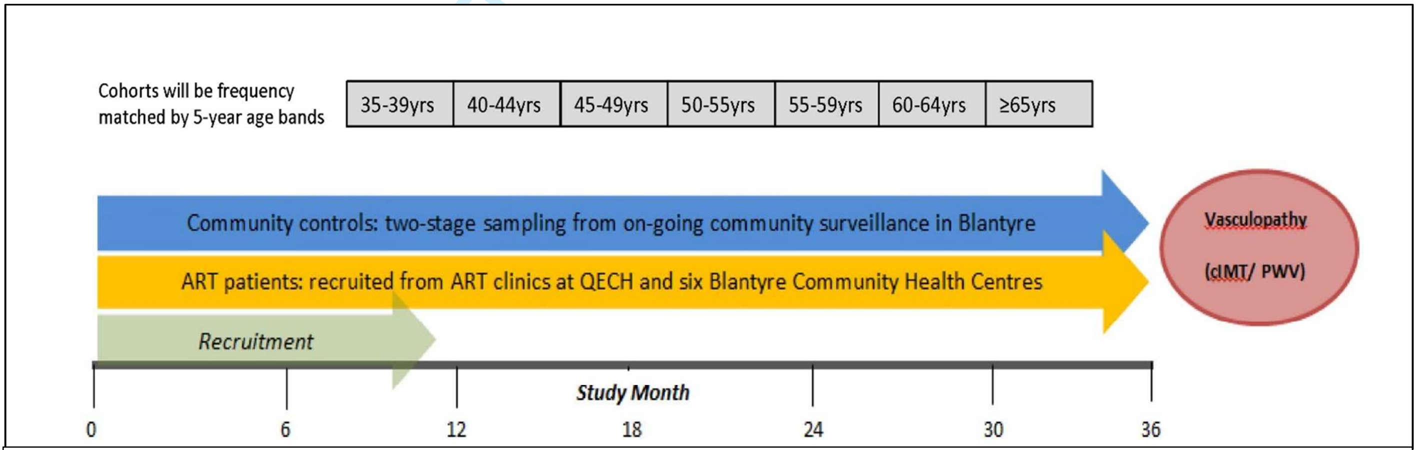

Figure 2. Outline of study design for a 36-month cohort study

KEY: yrs-years, CIMT-carotid intimal medial thickness, PWV – pulse wave velocity

For peer review only - http://bmjopen.bmj.com/site/about/guidelines.xhtml

BMJ OPEN

Table 1: Case definitions of primary and secondary endpoints for the study

	Type	Definitions
**Primary Endpoint**	Carotid intimal medial thickness (CIMT)	The occurrence of new-onset vasculopathy [CIMT – a measure of atherosclerosis]: CIMT >0.9 mm or >75th percentile of age/sex references values or presence of plaque on the carotid scan<br><br>Progression: total change in CIMT at 24 months from baseline
	Pulse wave velocity (PWV)	Occurrence of new onset vasculopathy [PWV – a measure of arterial stiffness]: PWV >12[m/s]<br><br>Progression: total change in PWV at 24 months from baseline
**Secondary endpoint**	Stroke	**Confirmed (1+2) or 3 or 4 or 5:**<br>1. Acute onset with a clinically compatible course, including unequivocal objective findings of a localizing neurologic deficit<br>2. CT or MRI compatible with a diagnosis of stroke and current neurologic signs and symptoms<br>3. Stroke diagnosed as the cause of death at autopsy<br>4. Compatible clinical history and positive lumbar puncture compatible with subarachnoid hemorrhage<br>5. Death certificate or death note from medical record listing stroke as the cause of death
	Myocardial Infarction [MI]	**Confirmed: One of the following 5 criteria (adapted from 2007 Universal Definition of Myocardial Infarction): [1 + (2 or 3 or 6)] or 4 or 5**<br><br>1. Rise and/or fall of cardiac biomarkers (preferably troponin), with at least one value above the 99th percentile of upper reference limit (URL);<br>2. The occurrence of a compatible clinical syndrome, including symptoms consistent with myocardial ischemia;<br>3. ECG changes indicative of new ischemia (new ST-changes or new left bundle branch block [LBBB]), or development of pathological Q waves on the ECG;<br>4. Sudden unexpected cardiac death involving cardiac arrest before biomarkers are obtained or before a time when biomarkers appear, along with (a) new ST-changes or new LBB, or (b) evidence of fresh thrombus on coronary angiography or at autopsy<br>5. Pathologic findings of acute myocardial infarction (including acute MI demonstrated as the cause of death on autopsy)<br>6. Development of a) evolving new Q waves, or b) evolving ST elevation, preferably based on at least 2 ECGs taken during the same hospital admission
	Coronary artery disease requiring drug treatment	**Confirmed (1 or 2) + 3:**<br><br>1. Evidence of myocardial ischemia based on either diagnostic imaging (such as a stress echocardiogram or thallium scan) or diagnostic changes on an electrocardiogram (such as during stress testing or an episode of chest pain)<br>2. Evidence of coronary artery disease based on coronary angiography or other diagnostic imaging<br>3. Use of medication given to treat or prevent angina (e.g., nitrates, beta blockers, calcium channel blockers)
	Peripheral vascular disease [PVD]	**Confirmed (1+2) or (1+3):**<br>1. Compatible clinical signs and symptoms<br>2. Positive results on diagnostic imaging studies (e.g., Doppler ultrasound, contrast arteriography, MRI arteriography); 3. Ankle Brachial Pressure Index < 0.90 in non-diabetics
	Vascular Immune reconstitution syndrome (IRIS)	A new onset vasculopathy within 6 months of starting ART
	All-cause death and vascular-related deaths	Death (of any or vascular cause) that occurs after recruitment into the study

For peer review only - http://bmjopen.bmj.com/site/about/guidelines.xhtml

BMJ OPEN

Table 2. Laboratory tests and clinical procedures in ART patients and HIV-uninfected adults

	Study Time Points						
	Baseline	6 months	12 months	18 months	24 months	30 months	36 months
**Clinical Procedures**							
PWV	X	X	X	X	X	X	X
CIMT	X				X		
ABPI	X	X	X	X	X	X	X
Cardiac Echo *(participant sub-set)*	X				X		
ECG *(participant sub-set)*	X				X		
**Cardiometabolic markers**							
Creatinine	X		X		X		X
Cholesterol (LDL, HDL, Triglycerides)	X		X		X		X
Serum glucose/HBA1C	X		X		X		X
**HIV Infection and Progression**							
HIV viral load *(HIV patients)*	X	X	X				
CD4 count *(HIV patients)*	X	X	X				
HIV rapid test *(controls)*	X		X		X		X
**Immune dysregulation**							
Soluble markers of systemic inflammation	X	X	X				
Soluble markers of endothelial activation	X	X	X				
CD8 and CD4 T-cell activation and senescence *(participant subset)*	X	X	X		X		X
Monocyte/ Macrophage activation and senescence *(participant subset)*	X	X	X		X		X
Herpesviruses infection							
CMV IgG	X	X					
VZV IgG	X	X					

For peer review only

For peer review only - http://bmjopen.bmj.com/site/about/guidelines.xhtml

BMJ OPEN

For peer review only

For peer review only - http://bmjopen.bmj.com/site/about/guidelines.xhtml

RHICCA – Clinical endpoint diagnostic criteria vs 3.5 Apr 2018

RHICCA
Reactivation of herpesviruses and inflammation as cardiovascular and cerebrovascular risk factors in ART initiators

	CONFIRMED	PROBABLE
**INFECTIONS**		
Aspergillosis, invasive pulmonary	**Confirmed: 1 + 2 + 3:**<br>1. Compatible clinical course **(Appendix 13)**,<br>2. CXR abnormality compatible with aspergillosis;<br>3. invasive mycelia consistent with Aspergillus on lung biopsy or positive culture of lung tissue or **positive culture of sputum** collected by any method	**Probable: 1 + 2 + 3:**<br>1. Compatible clinical course **(Appendix 13)**,<br>2. CXR abnormality compatible with aspergillosis;<br>3. invasive mycelia consistent with Aspergillus on tissue biopsy or positive culture at a normally sterile site (e.g., blood) apart from the lungs.
Aspergillosis, other invasive	**Confirmed: 1 + 2 + 3:**<br>1.compatible clinical course **(Appendix 11)**,<br>2. invasive mycelia consistent with Aspergillus on tissue biopsy or clinical evidence of infection,<br>3. positive culture from the affected tissue	**Probable: 1 + 2:**<br>1.clinical evidence of invasive infection **(Appendix 11)**, 2.invasive mycelia consistent with Aspergillus on tissue biopsy or positive culture at a normally sterile site (e.g., blood) apart from the involved tissue
Bartonellosis	**Confirmed 1+ 2:**<br>1.Clinical or histologic evidence of bacillary angiomatosis or bacillary peliosis,<br>2. a positive culture or PCR for *B. quintana* or *B. henselae*	**Probable 1 + 2:**<br>1.Clinical evidence of bacillary angiomatosis or bacillary peliosis **(Appendix 12)**,<br>2. positive silver stain for bacilli from a skin lesion or an affected organ
Candidiasis,oral	**Confirmed:1 + 2 + 3:**<br>1. Macroscopic appearance on examination of the mouth<br>2. microscopic evidence of yeasts or pseudo hyphae<br>3. no evidence of oesophageal involvement -	**Probable: 1 + 2 + 3:**<br>1. a clinical diagnosis of oral candidiasis and/or microscopic evidence of yeasts or pseudo hyphae<br>2. clinical response to treatment<br>3. no evidence of oesophageal involvement
Candidiasis of bronchi, trachea, or lungs	**Confirmed:1 + 2:**<br><br>Macroscopic appearance at bronchoscopy or autopsy<br>microscopic evidence of yeasts or pseudo hyphae	**None**
Candidiasis, esophageal	**Confirmed: 1 + 2:**<br>1. Macroscopic appearance at esophagoscopy or autopsy.<br>2. microscopic evidence of yeasts or pseudo hyphae	**Probable: 1 + 2 + 3:**<br>1. Recent onset of retrosternal pain or difficulty on swallowing.<br>2. a clinical diagnosis of oral candidiasis, endoscopic visualization of candidiasis and/or microscopic evidence of yeasts or pseudo hyphae from oropharyngeal mucosa<br>3. clinical response to treatment

For peer review only

RHICCA – Clinical endpoint diagnostic criteria vs 3.5 Apr 2018

RHICCA
Reactivation of herpesviruses and inflammation as cardiovascular and cerebrovascular risk factors in ART initiators

	CONFIRMED	PROBABLE
**INFECTIONS (CONTINUED)**		
Cryptococcosis, extrapulmonary (not meningitis)	**Confirmed: 1 or 2 or 3:**<br><br>From tissue other than lung or hilum:<br>1. microscopic demonstration of narrow based budding yeast<br>2. positive culture,<br>3. antigen detention	**None**
Cryptococcosis meningitis	**Confirmed: 1 or 2 or 3 or 4:**<br>1. Brain histopathology microscopic demonstration of narrow based budding yeast<br>2. CSF evidence of India ink test<br>3. CSF evidence of positive culture<br>4. CSF evidence of positive antigen detection	**None**
Cryptosporidiosis	**Confirmed: 1 + 2**<br>1. Diarrhea for > 1 month<br>2. positive microscopy	**None**
CMV retinitis	Autopsy demonstration	**Probable 1 + 2:**<br>1. Typical appearance on fundoscopy of discrete patches of retinal whitening, spreading along blood vessels.<br>2. Associated vasculitis, hemorrhage and necrosis, confirmed by ophthalmologist

	CONFIRMED	PROBABLE
**INFECTIONS (CONTINUED)**		
HZV single dermatome	**Confirmed 1+2:**<br>1.multiple ulcerated lesions affecting at least 1 dermatome, and/or 1 or more contiguous dematomes;<br><br>2. positive culture, PCR, or antigen assay from affected tissue	**Probable 1+ 2:**<br>1.multiple typical ulcerated lesions affecting at Least 1 dermatome, and/or 1 or more contiguous dermatomes;<br><br>2. response to an antiviral active against HZV unless resistance is demonstrated
HZV, disseminated	**Confirmed 1+2:**<br>1. multiple ulcerated lesions affecting at least 2 non-contiguous dermatomes, or with generalized cutaneous dissemination HZV involvement of the lung, liver, brain, or other internal organs<br>2. positive culture, PCR, or antigen assay from affected tissue	**Probable 1+2:**<br>1. multiple typical ulcerated lesions affecting at least 2 non-contiguous dermatomes, or with generalized cutaneous dissemination<br>2. response to an antiviral active against HZV unless resistance is demonstrated
HSV mucocutaneous ulceration	**Confirmed 1 +2:**<br>1.Ulceration for > 1 Month<br>2. Histology, culture, PCR, or detection of antigen from affected tissue	**Probable 1 + 2:**<br>1. Typical HSV ulceration for > 1 month,<br>2. response to an antiviral active against HZV unless resistance is demonstrated
Histoplasmosis, disseminated or extrapulmonary	**Confirmed 1+2:**<br>1.Compatible symptoms,<br>2. histology or culture or elevated blood or urine antigen levels	None
Isosporiasis	**Confirmed 1 + 2:**<br>1.Diarrhea for > 1 month<br>2.microscopic identification of *Isospora belli*	None

For peer review only - http://bmjopen.bmj.com/site/about/guidelines.xhtml

RHICCA – Clinical endpoint diagnostic criteria vs 3.5 Apr 2018

RHICCA
Reactivation of herpesviruses and inflammation as cardiovascular and cerebrovascular risk factors in ART initiators

Microsporidiosis	**Confirmed 1 + 2:**<br>1.Diarrhea for > 1 month<br>2.Microscopic identification of Microsporidia	None
MAC and other mycobacterial disseminated diseas	**Confirmed 1 + 2:**<br>1. Fever, fatigue, anemia or diarrhea<br>2.positive culture from blood, body fluids or tissue other than pulmonary, hilar or stool	**Probable 1+2+3:**<br>1. Fever, fatigue, anemia or diarrhea<br>2. **AFB** or positive direct MAC PCR in blood, body fluids or tissue other than pulmonary, hilar or stool<br>3. no concurrent non-pulmonary TB

-

	CONFIRMED	PROBABLE	POSSIBLE
M. tuberculosis disease, pulmonary	**Confirmed 1+2:**<br>1.Compatible symptoms of fever, dyspnea, cough, weight loss or fatigue<br>2. culture or PCR from **sputum** or bronchial lavage or lung tissue	**Probable 1+2+3+4:**<br>1.Symptoms of fever, dyspnea, cough, weight loss or fatigue<br>2.abnormal chest X-ray,<br>3. AFBs seen in sputum or lavage or lung tissue but not grown in culture,<br>4.responds to treatment	**Possible 1+2+3+4:**<br>1.Symptoms of fever, dyspnea , cough, weight loss or fatigue<br>2.abnormal chest X-ray compatible with pulmonary TB (such as upper lobe cavitation, pleural exudate)<br>3. No other etiology for pulmonary symptoms and signs identified,<br>4.Responds to anti tuberculosis treatment
M. tuberculosis disease, Extrapulmonary (not meningitis)	**Confirmed 1+2:**<br>1. Compatible symptoms<br>2. culture or PCR or MTB Xpert from blood or affected tissue (i.e. pericardial, ascites, and lymph glands)	**Probable 1+2+3**:<br>1.Compatible symptoms<br>2. AFBs seen from affected tissue or blood<br>3.concurrent diagnosis of pulmonary TB or responds to treatment	**Possible 1+2+3:**<br>1.Compatible symptoms<br>2. No other etiology for symptoms and signs identified<br>3.concurrent diagnosis of pulmonary TB or responds to treatment
M. tuberculosis disease, meningitis	**Confirmed 1+2:**<br>**1.** Clinical symptoms of meningism **(Appendix 7)**<br>**2.** Tissue/CSF culture, or PCR, or AFB or MTB Xpert	**Probable 1+ a score ≥12 (Appendix: Table 2):**<br>1. Clinical symptoms of meningism **(Appendix 7)**<br>2. A score ≥12, based on clinical, CSF, cerebral brain imaging criteria or evidence of TB elsewhere	
Nocardiosis	**Confirmed 1+2:**<br>1. Clinical evidence of invasive Infection **(Appendix 8)**<br>2. a positive culture from the affected tissue or blood	**Probable 1+2:**<br>1. Clinical evidence of invasive Infection **(Appendix 8)**<br>2. microscopic evidence of bronchial weakly acid fast organisms from the affected tissue	

For peer review only

RHICCA – Clinical endpoint diagnostic criteria vs 3.5 Apr 2018

RHICCA

Reactivation of herpesviruses and inflammation as cardiovascular and cerebrovascular risk factors in ART initiators

Pneumocystis jirovecii pulmonary	**Confirmed 1+2:**<br>1. compatible clinical syndrome **(Appendix 9)**<br>2. microscopic or histological demonstration of *P. jirovecii* cysts in a pulmonary specimen	Probable 1+2+3+4+5<br>1. dyspnea or cough, or fever progressive over > 1 week<br>2. **diffuse chest x-ray abnormality** or, if on inhalational pentamidine, diffuse upper lung field abnormality<br>3. evidence of hypoxia<br>4. not suggestive of bacterial pneumonia (i.e., not purulent sputum or hemoptysis, no bacterial pathogen identified in blood or bronchial wash)<br>5. response to PcJ treatment
Pneumocystis jirovecii, extrapulmonary	**Confirmed 1+2:**<br>1. compatible clinical syndrome<br>2. microscopic or histological demonstration of *P. jirovecii* cysts in a tissue other than pulmonary specimen	None

For peer review only

RHICCA – Clinical endpoint diagnostic criteria vs 3.5 Apr 2018

RHICCA

Reactivation of herpesviruses and inflammation as cardiovascular and cerebrovascular risk factors in ART initiators

	CONFIRMED	PROBABLE
**INFECTIONS (CONTINUED)**		
Pneumonia, **SINGLE EPISODE (isolated)**<br>bacterial, excludes: (a) post-obstructive pneumonias, including in those with intrapulmonary/bronchial lesions, (b) aspiration pneumonia, including in those with decreased consciousness levels, (c) nosocomial ventilator-associated pneumonias	**Confirmed 1+2+3:**<br>pneumonia episodes must occur after enrollment;<br>1. Signs and symptoms suggestive of bacterial pneumonia (**appendix 10**)<br>2. Focal CXR abnormality compatible with bacterial pneumonia,<br>3. identification of a bacterial pathogen by positive blood culture, positive bronchoscopic sputum culture, detection of Legionella or pneumococcal antigen in urine or blood or diagnostic serologic findings	**Probable 1+2:**<br>pneumonia episodes must occur after enrollment;<br>1.Signs and symptoms suggestive of bacterial pneumonia (**Appendix 10**)<br>2. Focal CXR abnormality compatible with Bacterial pneumonia
Pneumonia, **recurrent**<br>bacterial, excludes: (a) post-obstructive pneumonias, including in those with intrapulmonary/bronchial lesions, (b) aspiration pneumonia, including in those with decreased consciousness levels, (c) nosocomial ventilator-associated pneumonias	**Confirmed 1+2+3+4+5**<br>Second pneumonia episodes must occur after enrollment and after an isolated pneumonia, within 365 days of the first episode and with strong clinical evidence that the first episode was cured;<br>1. Signs and symptoms of second event suggestive of bacterial pneumonia (**Appendix 10**)<br>2. Focal CXR abnormality compatible with bacterial pneumonia,<br>3. identification of a bacterial pathogen by positive blood culture, positive bronchoscopic sputum culture, detection of Legionella or pneumococcal antigen in urine or blood or diagnostic serologic findings<br>4. the second pneumonia had onset of symptoms < 365 days after the first episode<br>5. there is strong evidence that the first episode was cured such as an intervening clear CXR or absence of symptoms after > 1 month off antibacterial effective against pathogens commonly producing pneumonia	**Probable 1+2+3+4:**<br>Second pneumonia episodes must occur after enrollment and after an isolated pneumonia, within 365 days of the first episode and with strong clinical evidence that the first episode was cured;<br>1. Signs and symptoms suggestive of bacterial pneumonia (**Appendix 10**)<br>2. focal CXR abnormality compatible with bacterial pneumonia<br>3. the second pneumonia had onset of symptoms < 365 days after the first episode<br>4. there is strong evidence that the first episode was cured such as an intervening clear CXR or absence of symptoms after > 1 month off antibacterials effective against pathogens commonly producing pneumonia
PML (progressive multifocal leukoencephalopathy)	**Confirmed 1 or 2:**<br>1. positive histology,<br>2. compatible clinical (**Appendix 11**) and radiologic course and positive CSF PCR for JK virus	**Probable 1+2+3:**<br>1. Consistent symptoms (**Appendix 11)**,<br>2. brain image consistent with PML,<br>3. no response to toxo treatment or toxoplasma [illegible]
Salmonella blood stream infection or bacteraemia, isolated	**Confirmed 1:**<br>A septic episode must occur after enrollment;<br>1. Positive blood or tissue culture	None
Salmonella blood stream infection or bacteraemia, recurrent	**Confirmed 1:**<br>A second septic episode must occur after enrollment and after an isolated episode;<br>1. Has met the criteria of isolated Salmonella septicemia<br>2. Positive blood or tissue culture on the second episode<br>3. the second septicemia had onset of symptoms < 365 days after the first episode<br>4. the second septicemia must be due to a different Salmonella serotype or there must be strong evidence that the first episode was cured such as a negative blood culture off effective antibacterials for > 1 week or absence of symptoms off antibacterials for > 1 month	None

For peer review only

For peer review only - http://bmjopen.bmj.com/site/about/guidelines.xhtml

RHICCA – Clinical endpoint diagnostic criteria vs 3.5 Apr 2018

RHICCA
Reactivation of herpesviruses and inflammation as cardiovascular and cerebrovascular risk factors in ART initiators

Toxoplasmosis of brain	**Confirmed 1+2+3:**<br>1. Compatible clinical findings **(Appendix 12)**<br>2. Compatible radiological findings<br>3. Detection of T gondii in the **CSF** or brain tissue (i.e. microscopy or PCR)	**Probable 1+2+3:**<br>1. Symptoms of focal intracranial abnormality or decreased consciousness<br>2. brain image consistent with lesion(s) enhanced by contrast<br>3. positive toxoplasma serology or responds to treatment clinically or by scan

	CONFIRMED	PROBABLE
**NEOPLASMS**		
Cervical carcinoma, invasive	**Confirmed 1:**<br>1. Histology (NOT carcinoma-in-situ)	**None**
Kaposi sarcoma, (mucocutaneous or visceral)	**Confirmed 1:**<br>1. Histology	1. Highly typical appearance<br>2. persistence for > 1 month
Lymphoma, primary, of brain	**Confirmed 1:**<br>1. Histology of brain tissue	**Probable 1+2+3:**<br>1. Symptoms consistent with lymphoma<br>2. at least one CNS lesion with mass effect<br>3. lack of clinical or radiographic response at least 2 weeks of treatment for toxoplasmosis
Lymphoma, Hodgkin's	1. Histology	None
Lymphoma, non-Hodgkin's, all cell types	**Confirmed 1:**<br>1. Histology	None
**NEUROLOGICAL**		
HIV-related encephalopathy (including AIDS Dementia Complex)	None	**Probable 1+2+3+4:**<br>1. Cognitive or motor dysfunction interfering with usual activity, progressive over weeks or months<br>2. no other condition to explain the findings<br>3. brain image obtained and suggests no other causes<br>4. grade 2 or worse impairment in at least 2 domains by NARS (**appendix – table 1**) excluding abnormal domains at trial entry. (For persons with abnormal domains at entry worsening by at least two grades meets criteria.)

For peer review only - http://bmjopen.bmj.com/site/about/guidelines.xhtml

RHICCA – Clinical endpoint diagnostic criteria vs 3.5 Apr 2018

RHICCA
Reactivation of herpesviruses and inflammation as cardiovascular and cerebrovascular risk factors in ART initiators

CARDIOVASCULAR DISEASES		
Acute Myocardial Infarction	**Confirmed: One of the following 5 criteria (adapted from 2007 Universal Definition of Myocardial Infarction): [1 + (2 or 3 or 6)] or 4 or 5**<br><br>1. Rise and/or fall of cardiac biomarkers (preferably troponin), with at least one value above 99th percentile of upper reference limit (URL);<br>2. Occurrence of a compatible clinical syndrome, including symptoms (such as chest pain **– see Appendix 1**) consistent with myocardial ischemia;<br>3. ECG changes indicative of new ischemia (new ST-changes or new left bundle branch block [LBBB]), or development of pathological Q waves on the ECG;<br>4. Sudden unexpected cardiac death involving cardiac arrest before biomarkers are obtained or before a time when biomarkers appear, along with (a) new ST-changes or new LBB, or (b) evidence of fresh thrombus on coronary angiography or at autopsy<br>5. Pathologic findings of acute myocardial infarction (including acute MI demonstrated as the cause of death on autopsy)<br>6. Development of a) evolving new Q waves, or b) evolving ST elevation, preferably based on at least 2 ECGs taken during the same hospital admission	**Probable 1 and 2:**<br>1. Occurrence of a compatible clinical syndrome (**Appendix 1**), including symptoms (such as chest pain) consistent with myocardial ischemia)<br>2. Development of a) evolving new Q waves, or b) evolving ST elevation, preferably based on at least; ECGs taken during the same hospital admission.
Peripheral vascular disease	**Confirmed (1+2) or (1+3):**<br>1. Compatible clinical signs and symptoms (**see Appendix 3**)<br>2. Positive results on diagnostic imaging studies (e.g., Doppler ultrasound, contrast arteriography, MRI arteriography); 3. Ankle Brachial Pressure Index < 0.90 in non-diabetics	**Probable 1:**<br>1. Compatible clinical signs and symptoms (**see Appendix 3**)
Stroke	**Confirmed (1+2) or 3 or 4 or 5:**<br>1. Acute onset with a clinically compatible course, including unequivocal objective findings of a localizing neurologic deficit **(appendix 4)**;<br>2. CT or MRI compatible with diagnosis of stroke and current neurologic signs and symptoms<br>3. Stroke diagnosed as cause of death at autopsy<br>4. Compatible clinical history and positive lumbar puncture compatible with subarachnoid hemorrhage<br>5. Death certificate or death note from medical record listing stroke as cause of death	**Probable (1+2) or (1+3):**<br><br>1. Acute onset with a clinically compatible course, including unequivocal objective findings of a localizing neurologic deficit **(appendix 4)**;<br>2. Positive lumbar puncture compatible with subarachnoid hemorrhage<br>3. Death certificate or death note from medical record listing stroke as cause of death
Congestive heart failure	**Confirmed (1+2) or (1+3) or (1+4):**<br>1. Clinical signs and symptoms compatible with left or right sided heart failure [**Appendix 5]** without an alternative explanation<br>2. Hemodynamic measurements, radionucleotide ventriculography, echocardiogram, cardiac catheterization, or multiple gated acquisition scan showing a decreased ejection fraction of < 45%<br>3. Echocardiogram, cardiac catheterization or other studies showing evidence of increased left atrial pressure or right heart failure;<br>4. Elevated levels of Brain Natriuretic Peptide (BNP) or NT-proBNP	**Probable 1+2+3:**<br>1. Clinical signs and symptoms compatible with left or right sided heart failure [**Appendix 5]** without an alternative explanation<br>2. Chest x-ray or other imaging study showing evidence of congestive heart failure, including cardiac enlargement;<br>3. Documentation of treatment for congestive heart failure

For peer review only

For peer review only - http://bmjopen.bmj.com/site/about/guidelines.xhtml

Coronary artery disease requiring drug treatment	**Confirmed (1 or 2) + 3:**<br><br>1. Evidence of myocardial ischemia based on either diagnostic imaging (such as a stress echocardiogram or thallium scan) or diagnostic changes on an electrocardiogram (such as during stress testing or an episode of chest pain)<br>2. Evidence of coronary artery disease based on coronary angiography or other diagnostic imaging<br>3. Use of medication given to treat or prevent angina (e.g., nitrates, beta blockers, calcium channel blockers)	**Probable 1+2:**<br><br>1. Other evidence of myocardial ischemia and/or coronary artery disease (including that based primarily upon symptoms and clinical presentation, such as chest pain with exertion)<br>2. Use of medication given to treat or prevent angina (e.g., nitrates, beta blockers, calcium channel blockers)
Deep vein thrombosis	**Confirmed 1:**<br>1. Diagnosis of deep vein thrombosis (DVT) by contrast venography, or **ultrasonography** other comparable imaging techniques;	**Probable (1)+2+3:**<br>1. An elevated **D-dimer test**;<br>2. A score on the Wells Clinical Prediction Rule for DVT of ≥ 3 points;<br>3. Absence of alternative diagnosis as likely or greater than that of deep venous thrombosis.<br>**Wells Clinical Prediction Rule for DVT (Appendix 6)**
**SYSTEMIC DISEASES**		
Anaemia<br>NOT FOR ERC<br>TO BE DETERMINED RETROSPECTIVELY	**Confirmed 1**<br>Classified according to both WHO and DAIDS thresholds for severe/grade 3-4 anaemia	
Chronic Kidney disease	**Confirmed: 1 or 2**<br><br>1. Kidney damage for >3 months, as defined by structural or functional abnormalities of the kidney with or without decreased GRF, manifest by either;<br>- Pathological abnormalities; or<br>- Markers of kidney damage, including abnormalities in the composition of the blood or urine or abnormalities in imaging results<br>2. GFR <60mL/min/1.73m2 for >3months, with or without kidney disease (estimated by CKD-EPI)	**Confirmed: 1 or 2**<br><br>1. Isolated Kidney damage, as defined by structural or functional abnormalities of the kidney with or without decreased GRF, manifest by either;<br>- Pathological abnormalities; or<br>- Markers of kidney damage, including abnormalities in the composition of the blood or urine or abnormalities in imaging results<br>2. Isolated GFR <60mL/min/1.73m2, with or without kidney disease (estimated by CKD-EPI)
End-stage renal disease	**Confirmed: 1**<br>1.Hemodialysis or peritoneal dialysis documented in a clinical note for a period of at least three months;	**Probable: 1**<br>1. Hemodialysis or peritoneal dialysis documented in a clinical note for a period of at least one month and up to the time of death in a patient who dies within three months after dialysis begins
Diabetes Mellitus<br><br>NOT FOR ERC<br>TO BE DETERMINED RETROSPECTIVELY	**Confirmed: 1 or 2 or 3 or 4**<br><br>1. Symptoms of diabetes plus casual plasma glucose concentration ≥ 200 mg/dL (11.1 *mmol/L*). (Casual is defined as any time of day without regard to time since last meal. The classic symptoms of diabetes include polyuria and polydipsia.)<br><br>2. Fasting plasma glucose ≥ 126 mg/dL (7.0 *mmol/L*). (Fasting is defined as no caloric intake for at least 8 hours.)<br><br>3. 2-hour post-load glucose ≥ 200 mg/dL (11.1 *mmol/L*) during an oral glucose tolerance test. (The test should be performed as described by WHO, using glucose load containing the equivalent of 75 g anhydrous glucose dissolved in water.)<br><br>4.An **HbA1c** of 48mmol/mol (6.5%) or above.	**None**

For peer review only

For peer review only - http://bmjopen.bmj.com/site/about/guidelines.xhtml

RHICCA – Clinical endpoint diagnostic criteria vs 3.5 Apr 2018

RHICCA
Reactivation of herpesviruses and inflammation as cardiovascular and cerebrovascular risk factors in ART initiators

Decompensate Liver disease	**Confirmed: 1+2**<br>1. Histologic, radiographic, or ultrasound evidence of cirrhosis, as documented by one of the following:<br>a. Histologic evidence of cirrhosis obtained by liver biopsy or autopsy<br>b. MRI or CT consistent with cirrhosis<br>c. A positive result on ultrasound imaging consistent with cirrhosis<br><br>2. Clinical evidence of decompensation, as documented by one of the following, and without an alternative explanation:<br>a. Ascites<br>b. Hepatic encephalopathy<br>c. Bleeding from gastric or esophageal varices<br>d. Spontaneous bacterial peritonitis	**Probable: 1**<br><br>1. Clinical evidence of decompensation, as documented by one of the following, and without an alternative explanation:<br>a. Ascites<br>b. Hepatic encephalopathy<br>c. Bleeding from gastric or esophageal varices<br>d. Spontaneous bacterial peritonitis
Hypertension	**Confirmed:** 1 or 2<br>1. An average of three blood pressure (BP) readings of systolic of 140 mm Hg or above and/or diastolic of 90 mm Hg or above on the same day and persist 5-7 days after the initial reading.<br>2. An isolated reading of 140mg systolic or 90mg diastolic and presence of the following end-organ disease:<br>a. Cardiac (i.e. left ventricular hypertrophy meeting the ECG criteria [**Appendix 2**] on evidence on cardiac echocardiogram)<br>b. Renal (i.e. microalbuminuria [urinary albumin excretion of 30-300mg/dl], elevated creatinine, reduced estimated GFR (60-90ml/min)<br>c. Retinal(i.e. hypertensive retinal changes)<br>d. Vascular disease (i.e. stroke [persisting on day 7], peripheral vascular disease, myocardial infarction, coronary artery disease requiring drug treatment, congestive cardiac failure)	**Probable: 1**<br><br>1. An average of three BP readings of systolic of 140 mm Hg or above and/or diastolic of 90 mm Hg or above on the same day.
Hyperlipidemia<br><br><br>NOT FOR ERC<br>TO BE DETERMINED RETROSPECTIVELY	**Confirmed: 1 or 2**<br>1. Fasting total cholesterol >200mg/dl (>5.2 mmol/L) or LDL cholesterol >130mg/dl (>3.4mmol/l) or Triglycerides >150 mg/dl (1.7 mmol/L)<br>2. Non-fasting total cholesterol >240mg/dl (>6.2 mmol/L) or LDL cholesterol >160mg/dl (>4.1 mmol/L) or Triglycerides >200 mg/dl (2.3mmol/L)	**None**
HIV wasting syndrome	None	**Probable:1 + 2 + 3**<br>1. unexplained, involuntary weight loss >10% from baseline,<br>2. persistent diarrhea with > 2 liquid stools/d for > 1 month or weakness for > 1 month or fever for > 1 month,<br>3. tests for alternate causes of weight loss, such as cancer, TB, MAC, cryptosporidiosis or other specific causes of weight loss, if obtained, should be negative

For peer review only

RHICCA – Clinical endpoint diagnostic criteria vs 3.5 Apr 2018

Appendix

1. Clinical syndrome of Myocardial infarction (a+ b +d) or (c+d)
   a. Chest pain (with associated clamminess, pallor)
   b. Radiation to the upper extremity and jaw
   c. Epigastric discomfort with exertion or at rest
   d. Severe discomfort lasting for more than 20 minutes

2. ECG criteria for LVH

Any two of the following 3 criteria's should be met:

Sokolow Lyon Criteria<br><br>• S in V_1 or V_2 + R in V_5 or V_6 (whichever is larger) ≥ 35 mm (≥ 7 large squares)<br>• R in aVL ≥ 11 mm<br><br>**Meets all of Sokolow Lyon criteria to be diagnostic**	
Cornell voltage criteria<br><br>ECG diagnosis of LVH involve measurement of the sum of the R wave in lead aVL and the S wave in lead V_3. The Cornell criteria for LVH are:<br><br>• S in V_3 + R in aVL > 28 mm (men)<br>• S in V_3 + R in aVL > 20 mm (women)<br><br>**Meets all of Cornell voltage criteria to be diagnostic**	
Romhilt-Estes point score system ECG Criteria<br>Voltage Criteria (any of):<br>1. R or S in limb leads ≥20 mm<br>2. S in V_1 or V_2 ≥30 mm<br>3. R in V_5 or V_6 ≥30 mm<br>--<br>ST-T Abnormalities:<br><br>1. ST-T vector opposite to QRS without digitalis<br>2. ST-T vector opposite to QRS with digitalis<br>3. Negative terminal P mode in V_1 1 mm in depth and 0.04 sec in duration (indicates left atrial enlargement)<br>4. Left axis deviation (QRS of -30° or more)<br>5. QRS duration ≥0.09 sec<br>6. Delayed R wave peak time (intrinsicoid deflection) in V_5 or V_6 (>0.05 sec)<br><br>**Romhilt-Estes point score >4 is diagnostic**	Points<br><br>3<br><br><br><br><br><br><br>3<br>1<br>3<br><br>2<br>1<br>1

3. Clinical syndrome of Peripheral vascular disease (a+ (b or c or d)
   a. Painful cramping in the hip, thigh or calf muscles after certain activities, such as walking or climbing stairs (claudication)
   b. femoral bruit
   c. decreased peripheral pulses
   d. change in color or temperature of limb suggesting peripheral arterial disease

For peer review only

For peer review only - http://bmjopen.bmj.com/site/about/guidelines.xhtml

RHICCA – Clinical endpoint diagnostic criteria vs 3.5 Apr 2018

RHICCA
Reactivation of herpesviruses and inflammation as cardiovascular and cerebrovascular risk factors in ART initiators

4. Clinical syndrome of stroke; should meet the 3 criteria's;

**1. Sudden onset**	
2. **Focal deficit** (or global disturbance but not seizures)	**Large artery disease (anterior circulation syndrome)**<br>Hemi-paresis + Hemi-sensory loss + higher cortical dysfunction (gaze paresis, language impairment [expression + comprehension], visual field defect, hemi-neglect)<br><br>**Large artery disease (posterior circulation syndrome)**<br>Vertigo, visual field defect, gaze paresis, double vision, swallowing difficulty, crossed signs [contralateral limb weakness and ipsilateral cranial nerves abnormality], ataxic limb and gait, drowsy/loss of consciousness<br><br>**Small vessel disease (lacunar syndrome)**<br>Pure hemi-sensory loss<br>Pure hemiparesis<br>Pure sensorimotor<br>Pure ataxic hemiparesis (including dysarthria-clumsy hand syndrome)<br><br>Thunderclap headache*
3. **Lasting > 24 hours** (<24 hours is a TIA)	
*seen in those with a suspicion of subarachnoid or venous stroke. In this case criteria 1 and 3 does not necessarily have to be met	

5. Clinical syndrome of congestive heart failure:

Using the Framingham criteria relies on clinical signs and symptoms; 1 or more major and two or more minor criteria are clinically suggestive of heart failure:

Major criteria

A. Acute pulmonary edema
B. Cardiomegaly
C. Hepatojugular reflex
D. Neck vein distention
E. Paroxysmal nocturnal Dyspnea or Orthopnea
F. Pulmonary crackles
G. Third Heart Sound (S3 Gallup Rhythm)

Minor Criteria

A. Ankle edema
B. Dyspnea on exertion
C. Hepatomegaly
D. Nocturnal cough
E. Pleural Effusion
F. Tachycardia (Heart Rate >120 beats per minute)

For peer review only

RHICCA – Clinical endpoint diagnostic criteria vs 3.5 Apr 2018

6. Wells Clinical Prediction Rule for DVT (Adapted from: Wells PS et al. Lancet 1997;350:1796).

One point for each of the following:

- Active cancer (treatment ongoing or within previous 6 months, or palliative)
- Paralysis, paresis, or plaster immobilization of lower extremities
- Recently bedridden for more than 3 days, or major surgery, within 4 weeks
- Localized tenderness along distribution of the deep venous system
- Entire leg swollen
- Calf swelling by more than 3 cm when compared with the asymptomatic leg (measured 10 cm below tibial tuberosity)
- Pitting edema (greater in the symptomatic leg)
- Collateral superficial veins (non-varicose)

7. Clinical symptoms of meningism
   Meninigism is the triad of nuchal **rigidity** (neck stiffness), **photophobia** (intolerance of bright light) and **headache**.

8. Clinical symptoms of norcardia

   Symptoms vary and depend on the organs involved.

   If in the lungs, symptoms may include:
   - Chest pain when breathing (may occur suddenly or slowly)
   - Coughing up blood
   - Fevers
   - Night sweats
   - Weight loss

   If in the brain, symptoms may include:
   - Fever
   - Headache
   - Seizures
   - If the skin is affected, symptoms may include:
   - Skin breakdown
   - Skin breakdown and abnormal passage or draining tract (fistula)
   - Ulcers or nodules with infection sometimes spreading along lymph nodes

   Some people with nocardia infection have no symptoms.

9. Symptoms of Pneumocystis Pneumonia
   - Fever.
   - Mild and dry cough or wheezing.
   - Shortness of breath, especially with activity.
   - Rapid breathing.
   - Fatigue.
   - Major weight loss.
   - Chest pain when you breathe.

10. Clinical syndrome of bacterial pneumonia
    - cough with thick yellow, green, or blood-tinged mucus.
    - chest pain that worsens when coughing or breathing.
    - sudden onset of chills.
    - fever of 102°F or above (fever lower than 102°F in older persons)
    - headache.
    - muscle pain.
    - breathlessness or rapid breathing.

For peer review only

For peer review only - http://bmjopen.bmj.com/site/about/guidelines.xhtml

RHICCA – Clinical endpoint diagnostic criteria vs 3.5 Apr 2018

11. Clinical finding of Central Nervous System PML
    - Deficits in motor function, especially **weakness** and **clumsiness**, are common
    - associated altered mental state or behaviour and fever

12. Clinical finding of CNS toxoplasmosis
    - Headaches
    - Seizures
    - Focal neurological deficit of a subacute onset
    - confusion and coma
    - A lung infection, causing cough, fever, and shortness of breath may co-exist.
    -

13. Clinical symptoms suggest of Aspergillosis;

- Fever and chills.
- Cough that brings up blood-streaked sputum (hemoptysis)
- Severe bleeding from the lungs.
- Shortness of breath.
- Chest or joint pain.
- Headaches or eye symptoms.
- Nosebleed
- Facial swelling on one side

Table 1: Abbreviated NARS (Neuropsychiatric AIDS Rating Scale) Grading for HIV Encephalopathy
Adapted from: Price RW, Brew BJ. The AIDS dementia complex. J Infect Dis 1988; 158 (5): 1079-83, and Hughes CP, Berg L, Danziger WL. A new clinical scale for the staging of dementia. Brit J Psych 1982; 140: 566-92.

NARS stage	Cognitive-Behavioral Domains					
	**Orientation**	**Memory**	**Motor**	**Behavior**	**Problem solving**	**Activities of daily living**
**0.5**	fully oriented	complains of memory problems	fully ambulatory slightly slowed movements	normal	has slight mental slowing	slight impairment in business dealings
**1**	fully oriented, may have brief periods of "spaciness"	mild memory problems	balance, co-ordination and handwriting difficulties	more irritable, labile or apathetic, withdrawn	difficulty planning and completing work	can do simple daily tasks, may need prompting
**2**	some disorientation	memory moderately impaired, new learning impaired	ambulatory but may require walking aid	some impulsivity or agitated behavior	severe impairment, poor social judgement, gets lost easily	needs assistance with ADLs
**3**	frequent disorientation	severe memory loss, only fragments of memory remain	ambulatory with assistance	may have organic psychosis	judgement very poor	cannot live independently
**4**	confused and disoriented	virtually no memory	bedridden	mute and unresponsive	no problem solving ability	nearly vegetative

For peer review only

RHICCA – Clinical endpoint diagnostic criteria vs 3.5 Apr 2018

RHICCA
Reactivation of herpesviruses and inflammation as cardiovascular and cerebrovascular risk factors in ART initiators

For peer review only

RHICCA – Clinical endpoint diagnostic criteria vs 3.5 Apr 2018

Table 2: Diagnostic criteria for classification of definite, probable, possible, and not tuberculosis meningitis (Marais S, et al. Lancet Infect Dis 2010)

	Diagnostic score
**Clinical criteria**	(Maximum category score=6)
Symptom duration of more than 5 days	4
Systemic symptoms suggestive of tuberculosis (one or more of the following): weight loss (or poor weight gain in children), night sweats, or persistent cough for more than 2 weeks	2
History of recent (within past year) close contact with an individual with pulmonary tuberculosis or a positive TST or IGRA (only in children <10 years of age)	2
Focal neurological deficit (excluding cranial nerve palsies)	1
Cranial nerve palsy	1
Altered consciousness	1
**CSF criteria**	(Maximum category score=4)
Clear appearance	1
Cells: 10–500 per µl	1
Lymphocytic predominance (>50%)	1
Protein concentration greater than 1 g/L	1
CSF to plasma glucose ratio of less than 50% or an absolute CSF glucose concentration less than 2.2mmol/L	1
**Cerebral imaging criteria**	(Maximum category score=6)
Hydrocephalus	1
Basal meningeal enhancement	2
Tuberculoma	2
Infarct	1
Pre-contrast basal hyperdensity	2
Evidence of tuberculosis elsewhere	(Maximum category score=4)
**Chest radiograph** suggestive of active tuberculosis: signs of tuberculosis=2; miliary tuberculosis=4	2/4
**CT/ MRI/** ultrasound evidence for tuberculosis outside the CNS 2	
**AFB** identifi ed or *Mycobacterium tuberculosis* cultured from another source—ie, sputum, lymph node, gastric	2
washing, urine, blood culture	4
Positive commercial *M tuberculosis* NAAT from extra-neural specimen	
	4
Exclusion of alternative diagnoses	

An alternative diagnosis must be confirmed microbiologically (by stain, culture, or NAAT when appropriate), serologically (eg, syphilis), or histopathologically (eg, lymphoma). The list of alternative diagnoses that should be considered, dependent upon age, immune status, and geographical region, include: pyogenic bacterial meningitis, cryptococcal meningitis, syphilitic meningitis, viral meningo-encephalitis, cerebral malaria, parasitic or eosinophilic meningitis (*Angiostrongylus cantonesis*, *Gnathostoma spinigerum*, toxocariasis, cysticercosis), cerebral toxoplasmosis and bacterial brain abscess (space-occupying Lesion on cerebral imaging)and malignancy (eg, lymphoma)

TST=tuberculin skin test. IGRA=interferon-gamma release assay. NAAT=nucleic acid amplification test. AFB=acid-fast bacilli. The individual points for each criterion (one, two, or four points) were determined by consensus and by considering their quantified diagnostic value as defined in studies.

Key:
Bold text: of the options available likely to be the only tool available in a Malawi setting
Greyed out text: ideal investigation but not available in a Malawi setting

For peer review only

For peer review only - http://bmjopen.bmj.com/site/about/guidelines.xhtml

STROBE Statement—checklist of items that should be included in reports of observational studies

	Item No	Recommendation
**Title and abstract**	**1**	(*a*) Indicate the study's design with a commonly used term in the title or the abstract
		(*b*) Provide in the abstract an informative and balanced summary of what was done and what was found
**Introduction**		
Background/rationale	**2**	Explain the scientific background and rationale for the investigation being reported
Objectives	**3**	State specific objectives, including any prespecified hypotheses
**Methods**		
Study design	**4**	Present key elements of study design early in the paper
Setting	**5**	Describe the setting, locations, and relevant dates, including periods of recruitment, exposure, follow-up, and data collection
Participants	**6**	(*a*) *Cohort study*—Give the eligibility criteria, and the sources and methods of selection of participants. Describe methods of follow-up<br>*Case-control study*—Give the eligibility criteria, and the sources and methods of case ascertainment and control selection. Give the rationale for the choice of cases and controls<br>*Cross-sectional study*—Give the eligibility criteria, and the sources and methods of selection of participants
		(*b*) *Cohort study*—For matched studies, give matching criteria and number of exposed and unexposed<br>*Case-control study*—For matched studies, give matching criteria and the number of controls per case
Variables	**7**	Clearly define all outcomes, exposures, predictors, potential confounders, and effect modifiers. Give diagnostic criteria, if applicable
Data sources/ measurement	**8***	For each variable of interest, give sources of data and details of methods of assessment (measurement). Describe comparability of assessment methods if there is more than one group
Bias	**9**	Describe any efforts to address potential sources of bias
Study size	**10**	Explain how the study size was arrived at
Quantitative variables	**11**	Explain how quantitative variables were handled in the analyses. If applicable, describe which groupings were chosen and why
Statistical methods	**12**	(*a*) Describe all statistical methods, including those used to control for confounding
		(*b*) Describe any methods used to examine subgroups and interactions
		(*c*) Explain how missing data were addressed
		(*d*) *Cohort study*—If applicable, explain how loss to follow-up was addressed<br>*Case-control study*—If applicable, explain how matching of cases and controls was addressed<br>*Cross-sectional study*—If applicable, describe analytical methods taking account of sampling strategy
		(*e*) Describe any sensitivity analyses

Continued on next page

For peer review only - http://bmjopen.bmj.com/site/about/guidelines.xhtml

For peer review only

**Results**		
Participants	13*	(a) Report numbers of individuals at each stage of study—eg numbers potentially eligible, examined for eligibility, confirmed eligible, included in the study, completing follow-up, and analysed
		(b) Give reasons for non-participation at each stage
		(c) Consider use of a flow diagram
Descriptive data	14*	(a) Give characteristics of study participants (eg demographic, clinical, social) and information on exposures and potential confounders
		(b) Indicate number of participants with missing data for each variable of interest
		(c) *Cohort study*—Summarise follow-up time (eg, average and total amount)
Outcome data	15*	*Cohort study*—Report numbers of outcome events or summary measures over time
		Case-control study—Report numbers in each exposure category, or summary measures of exposure
		Cross-sectional study—Report numbers of outcome events or summary measures
Main results	16	(*a*) Give unadjusted estimates and, if applicable, confounder-adjusted estimates and their precision (eg, 95% confidence interval). Make clear which confounders were adjusted for and why they were included
		(*b*) Report category boundaries when continuous variables were categorized
		(*c*) If relevant, consider translating estimates of relative risk into absolute risk for a meaningful time period
Other analyses	17	Report other analyses done—eg analyses of subgroups and interactions, and sensitivity analyses
**Discussion**		
Key results	18	Summarise key results with reference to study objectives
Limitations	19	Discuss limitations of the study, taking into account sources of potential bias or imprecision. Discuss both direction and magnitude of any potential bias
Interpretation	20	Give a cautious overall interpretation of results considering objectives, limitations, multiplicity of analyses, results from similar studies, and other relevant evidence
Generalisability	21	Discuss the generalisability (external validity) of the study results
**Other information**		
Funding	22	Give the source of funding and the role of the funders for the present study and, if applicable, for the original study on which the present article is based

*Give information separately for cases and controls in case-control studies and, if applicable, for exposed and unexposed groups in cohort and cross-sectional studies.

Note: An Explanation and Elaboration article discusses each checklist item and gives methodological background and published examples of transparent reporting. The STROBE checklist is best used in conjunction with this article (freely available on the Web sites of PLoS Medicine at http://www.plosmedicine.org/, Annals of Internal Medicine at http://www.annals.org/, and Epidemiology at http://www.epidem.com/). Information on the STROBE Initiative is available at www.strobe-statement.org.

For peer review only

BMJ Open

Protocol for a longitudinal cohort study to evaluate the reactivation of herpesviruses and inflammation as cardiovascular and cerebrovascular risk factors in antiretroviral therapy initiators, in an African HIV-infected population (RHICCA)

Journal:	*BMJ Open*
Manuscript ID	bmjopen-2018-025576.R1
Article Type:	Protocol
Date Submitted by the Author:	08-Jan-2019
Complete List of Authors:	Peterson, Ingrid; Liverpool School of Tropical Medicine; Malawi Liverpool Wellcome Trust Clinical Research Programme<br>Ntsui, Ntobeko; University of Cape Town<br>Jambo, Kondwani; Liverpool School of Tropical Medicine; Malawi Liverpool Wellcome Trust Clinical Research Programme<br>Kelly, Christine; Malawi Liverpool Wellcome Trust Clinical Research Programme; University College Dublin<br>Huwa, Jacqueline; Malawi Liverpool Wellcome Trust Clinical Research Programme<br>Afran, Louise; Malawi Liverpool Wellcome Trust Clinical Research Programme<br>Tatuene, Joseph; University of liverpool, Institute of Infection and Global Health; Malawi-Liverpool-Wellcome Trust Clinical Research Programme,<br>Pett, Sarah; University College London, Institute of Infection and Global Health; University of New South Wales, Kirby Institute<br>Henrion, Marc; Malawi Liverpool Wellcome Trust Clinical Research Programme; Liverpool School of Tropical Medicine<br>Van Oosterhout, Joep; University of Malawi College of Medicine; Dignitas International<br>Heyderman, Robert; University College London, Division of Infection and Immunity; University of Malawi College of Medicine, Malawi Liverpool Wellcome Trust Clinical Research Programme<br>Mwandumba, Henry; Liverpool School of Tropical Medicine; Malawi Liverpool Wellcome Trust Clinical Research Programme<br>Benjamin, Laura; University of Liverpool Institute of Infection and Global Health, ; University College London Institute of Neurology,
**Primary Subject Heading**:	Cardiovascular medicine
Secondary Subject Heading:	HIV/AIDS, Immunology (including allergy), Infectious diseases, Global health
Keywords:	Ischaemic heart disease < CARDIOLOGY, EPIDEMIOLOGY, HIV & AIDS < INFECTIOUS DISEASES, Stroke medicine < INTERNAL MEDICINE, Cardiovascular

SCHOLARONE™
Manuscripts

BMJ OPEN

Title: Protocol for a longitudinal cohort study to evaluate the reactivation of herpesviruses and inflammation as cardiovascular and cerebrovascular risk factors in antiretroviral therapy initiators, in an African HIV-infected population (RHICCA)

Authors:

Ingrid Peterson[1,2], Ntobeko Ntsui[3], Kondwani C Jambo[1,2], Christine Kelly[1,4], Jacqueline Huwa[1], Louise Afran[1], Joseph Kamtchum-Tatuene[5], Sarah Pett[6,7,8], Marc Henrion[1,2], Joep Van Oosterhout[9,10], Robert Heyderman[11], Henry C Mwandumba[1,2], Laura A Benjamin[4,12**] and for the Investigators of the RHICCA study*

Affiliations:

1. Malawi-Liverpool-Wellcome Trust Clinical Research Programme, University of Malawi College of Medicine
2. Department of Clinical Sciences, Liverpool School of Tropical Medicine
3. Department of Medicine, University of Cape Town
4. HIV Molecular Research Group, University College Dublin
5. Institute of Infection and Global Health, University of Liverpool
6. Institute of Global Health, University College London
7. MRC CTU at UCL, Institute of Medicine, Clinical Trials and Methodology, University College London
8. Kirby Institute, University of New South Wales, Australia
9. Dignitas International, PO Box 071, Zomba, Malawi
10. College of Medicine, University of Malawi
11. Department of Infection and Immunity, University College London
12. Department of Brain Repair and Rehabilitation, Institute of Neurology, UCL

For peer review only - http://bmjopen.bmj.com/site/about/guidelines.xhtml

BMJ OPEN

*The Investigators of the RHICCA study

Brian Angus - Oxford Centre for Clinical Tropical Medicine, University of Oxford

Myles Connor - University of Edinburgh

Reena Dwivedi - Greater Manchester Neurosciences Centre, Salford Royal Foundation Trust

Lewis Haddow - Institute for Global Health, University College London

Terttu Heikinheimo-Connell - Hyvinkää Hospital, Department of Neurology, University of Helsinki

Elizabeth Joekes - Liverpool School of Tropical Medicine

Vanessa Kandoole - Department of Medicine, University of Malawi College of Medicine, Blantyre

Moffat Nyrienda - MRC Research Unit, Uganda

Kennedy Malisita- Department of Medicine, Queen Elizabeth Central Hospital

Jane Mallewa- Department of Medicine, University of Malawi College of Medicine, Blantyre

Elsayed Z. Soliman - School of Medicine, Wake Forest School of Medicine

Tom Solomon - Institute of Infection and Global Health, University of Liverpool

**Corresponding author

Laura Benjamin

Institute of Infection and Global Health,

Ronald Ross building,

The University of Liverpool,

L69 7BE, Liverpool,

United Kingdom

For peer review only - http://bmjopen.bmj.com/site/about/guidelines.xhtml

BMJ OPEN

l.benjamin@liverpool.ac.uk

Key words:

Cardiovascular, cerebrovascular, HIV, herpesvirus, Immune dysregulation,

Journal Guidance:

Abstract word count: 300/300

Article word count: 4095 /4000

Figure/Table: 5/5

For peer review only

BMJ OPEN

ABSTRACT

Introduction: In Sub-Saharan Africa, rising rates of cerebrovascular and cardiovascular disease (CBD/CVD) are intersecting with an aging HIV-infected population. The widespread use of antiretroviral therapy (ART) may confer an additive risk and may not completely suppress the risk associated with HIV infection. High-quality prospective studies are needed to determine if HIV-infected patients in Africa are at increased risk of CBD/CVD and to identify factors associated with this risk. This study will test the hypothesis that immune activation and dysfunction, driven by HIV and reactivation of latent herpesvirus infections lead to increased CBD/CVD risk in Malawian adults aged $\geq$ 35 years.

Methods and Analysis: We will conduct a single-centre 36-month prospective cohort study in 800 HIV-infected patients initiating antiretroviral therapy (ART) and 190 HIV-uninfected controls in Blantyre, Malawi. Patients and controls will be recruited from government ART clinics and the community, respectively and will be frequency-matched by 5-year age band and sex. At baseline and follow-up visits, we will measure carotid intima thickness (CIMT), pulse wave velocity (PWV) as surrogate markers of vasculopathy, and thus CBD/CVD risk. Our primary exposures of interest will be prospectively measured; these include cytomegalovirus and varicella zoster reactivation, changes in HIV plasma viral load, and markers of systemic inflammation and endothelial function. Multivariable regression models will be developed to assess the study's primary hypothesis. The occurrence of clinical CBD/CVD will be assessed as secondary study endpoints. ISRCTN registry https://doi.org/10.1186/ISRCTN42862937.

Ethics and dissemination: This was approved by the University of Malawi College of Medicine and the Liverpool School of Tropical Medicine research ethics committees. Our goal is to gain insight into the pathogenesis of cardiovascular and cerebrovascular disease among HIV cohorts on ART, in sub-Saharan Africa, and provide data to inform future interventional clinical trials. This study started in May 2017 and will continue until August 2020.

For peer review only - http://bmjopen.bmj.com/site/about/guidelines.xhtml

STRENGTHS AND LIMITATIONS

- This is one of the first large-scale studies in Sub-Saharan Africa to explore the relationship between HIV infection, latent herpesviruses, inflammation and cardiovascular and cerebrovascular diseases, immediately after starting antiretroviral therapy (ART).
- Clinical events and death will be comprehensively reviewed through an end-point review committee, using strict diagnostic criteria for events based on those used in the INSIGHT network, or validated verbal autopsy for death with limited data.
- Because of the recent roll-out of ART in asymptomatic patients, there will be an absence of ART-naïve population, limiting our ability to explore the impact of ART.
- Approximately one-third of strokes will be asymptomatic. We anticipate not capturing some of these. However, multiple cerebral infarcts without a focal neurological deficit will manifest as cognitive impairment, which we will screen for, and corroborate with MRI imaging in a small number of symptomatic cases.
- Two-thirds of myocardial infarction will be silent and could potentially be missed. In a nested group, we will use a digital electrocardiogram to evaluate this further.

For peer review only - http://bmjopen.bmj.com/site/about/guidelines.xhtml

INTRODUCTION

The growing epidemic of cerebrovascular disease (CBD e.g. Stroke) and cardiovascular disease (CVD e.g. myocardial infarction) now intersects with the HIV epidemic[1]. Countries like Malawi, have an adult HIV prevalence of approximately 10%[2]. There is an increased life expectancy among people living with HIV, largely because of the successful scale-up of ART[3]. In Europe and the US, HIV is associated with a 50% increased risk of CVD compared to HIV-uninfected populations[4], attributable to long-term antiretroviral therapy (ART) use and HIV *per se*[4 5]. HIV infection is also associated with a 1.8 fold increased risk of all-cause heart failure in US veterans[6]. Our recent case-control study of stroke in Malawian adults is one of several examples that demonstrates a high risk of HIV infection associated with stroke and heart disease, pointing to a considerable and unappreciated CBD/CVD risk among HIV patients, in this setting[7-10].

There are reports of geographical differences in the distribution of CVD risk factors, supporting the argument that evidence derived from high-income countries cannot be applied to Sub-Saharan (SSA)[11]. Addressing this knowledge gap is essential to the development of clinical drug trials for primary prevention of CBD/CVD among individuals living with HIV. Vasculopathy due to accelerated atherosclerosis, arterial stiffening and vasculitis are the major mechanisms believed to underlie the CBD/CVD burden[12 13]. It is hypothesized that despite viral suppression, low-grade HIV virus replication and the associated host systemic inflammation are important drivers of this vasculopathy (Figure 1). In patients receiving ART, HIV antigenemia, partly resulting from HIV persistence in sanctuary sites, incomplete virologic suppression and virologic resurgence, drives the chronic immune activation observed in about 20% of ART patients in SSA[14]. This immune state is characterized by ongoing activation and senescence of cell-mediated immunity[15 16], increased monocyte/macrophage activation, stimulation of the interleukin-6 (IL-6) pathway and production of acute phase proteins[17-19]. Activation of the IL-6 pathway is established with atherosclerosis[20 21], and may also contribute to non-atherosclerotic vasculopathy. Inflammation alone confers a 2-fold increased risk of clinical CBD/CVD events[22]. The current push to introduce more effective ART regimens, and to start treatment soon after HIV diagnosis is made, may reduce inflammation and in turn, CBD/CVD risk[23]. However, there is

For peer review only - http://bmjopen.bmj.com/site/about/guidelines.xhtml

growing evidence of chronic inflammation in HIV despite achieving the goal of therapy, which is long-term suppression (<50 copies/mL) of plasma viral load, suggesting adjunctive therapy may be required.[24-26]

In addition to HIV, there is compelling evidence that reactivation of latent herpesviruses may be an important cause of vasculopathy. In HIV-uninfected elderly populations from high-income settings, latent cytomegalovirus (CMV) infection drives dysregulation of cell-mediated immunity[15 27-29], not dissimilar to what's described in HIV-associated immune activation[29]. CMV and other viral proteins have been found in atherosclerotic plaques[20]. Varicella-zoster virus (VZV) can directly infect the vascular endothelium to cause vasculitis and subsequent stroke and was found to be the commonest opportunistic infection (prevalence 15%) in a study of HIV-infected stroke patients in Malawi [12]. The seroprevalence of herpesviruses is high in SSA[30], particularly in HIV-infected populations[16].

The involvement of herpesviruses in the mechanistic pathway for CBD/CVD is compelling and may offer additional therapeutic avenues, especially for CMV and VZV. However, our understanding is incomplete, and its population impact is yet to be defined. It is important to determine if, in addition to ART, there is a role for other pharmacological interventions targeting latent viral infections or downstream inflammatory pathways to reduce vasculopathy in HIV-infected patients on ART. Previous work from North America supports the potential of treating reactivated herpesviruses[31]. Furthermore, there are opportunities for intervention using the recently licensed Letermovir; a treatment for CMV. By focusing on HIV and Herpes viral antigenemia and immune dysregulation as mechanisms of vasculopathy, this study will identify subgroups of HIV-infected patients on ART at high risk of CBD/CVD, the timing of CBD/CVD risk in such patients, as well as potential targets for intervention.

For peer review only - http://bmjopen.bmj.com/site/about/guidelines.xhtml

STUDY OBJECTIVES

This study will test the hypothesis that immune activation and dysfunction, driven by HIV and reactivation of latent CMV/VZV herpesvirus infections lead to increased CBD/CVD risk in adults aged $\geq$ 35 years in SSA. We will address this through the following objectives;

1) To determine if progression of the surrogate marker of CBD/CVD or occurrence of new-onset vasculopathy is higher in adults aged $\geq$ 35 years with HIV infection on ART compared to those without HIV.
2) To determine if progression of surrogate markers of CBD/CVD or occurrence of new-onset vasculopathy is higher in adults aged $\geq$ 35 years with HIV/herpes viral antigenemia or chronic immune activation compared to those without HIV/herpes viral antigenemia or chronic immune activation. Specifically, we will determine if progression of surrogate markers or new-onset vasculopathy is higher:
    a. in ART patients with reactivated latent herpes viral infection, compared to those without reactivated latent herpes viral infection.
    b. in ART patients with the highest 25% of markers for immune activation, inflammation or endothelial activation compared to the bottom 25%
    c. in ART patients with incomplete virologic suppression or virologic resurgence of HIV, compared to those with suppressed HIV plasma viral load.

The secondary study objectives are to determine if viral antigenemia or chronic immune activation increase occurrence of the following clinical events: 1) stroke, 2) myocardial infarction (MI), 3) angina (excluding MI), 4) peripheral vascular disease (PVD), 5) all-cause death/vascular-related death and 6) immune reconstitution inflammatory vasculopathy.

METHODS AND ANALYSIS

Study design

To address objective 1, we will conduct a single-center 36-month prospective cohort study in 800 HIV-infected patients initiating ART and 190 HIV-uninfected adults aged $\geq$ 35 years. HIV-infected and HIV-uninfected participants will be frequency matched by 5-year age band and sex. On a 6-monthly basis, we will measure markers of viral infection, inflammation and endothelial function along with surrogate markers for CBD/CVD (Table 1).

For peer review only - http://bmjopen.bmj.com/site/about/guidelines.xhtml

Study Setting

This study will recruit consecutive ART patients from the ART clinic of Queen Elizabeth Central Hospital (QECH), and ART clinics in several Blantyre City Community Health Centres (CHCs). These clinics collectively initiate over 100 HIV-infected patients aged ≥ 35 years onto ART each month. HIV-uninfected adults will be selected from pre-ART counseling sessions, and from randomly selected households in the community by two-stage random sampling (of households and individuals within households) from a previously enumerated sampling frame in the CHC catchment areas[32]. All study procedures will be conducted at QECH, which is located adjacent to the Malawi-Liverpool-Wellcome Trust Clinical Research Programme (MLW). QECH also hosts a 0.35T MRI imaging facility, which will contribute to characterizing our secondary endpoints.

Study Participants

Study inclusion criteria will be: a) age ≥ 35 years and b) resident in Blantyre. HIV-infected patients must further be: c) ART-naïve or initiated ART <10 days prior to enrolment and d) initiating standard first-line ART (in Malawi this is: Tenofovir [TDF]/Lamivudine [3TC]/Efavirenz [EFV]). Adult controls must further be: e) HIV-uninfected. Study exclusion criteria are: f) clinical history of CBD/CVD, g) pregnancy, h) critical illness or symptomatic anemia at baseline and i) enrollment in an intervention study. At the analysis stage abnormal PWV at baseline (as defined in Table 2) will be excluded for new-onset vasculopathy analysis but not for progression of vasculopathy. The same approach will be applied for baseline CIMT measurements. If the study participant becomes pregnant after recruitment, they will be withdrawn.

Justification of study inclusion and exclusion criteria is as follows; in many populations, CBD/CVD risk rises sharply from 35-years of age[33], thus individuals aged 35 and older will be eligible (recruitment of participants aged 35 -39 will be limited to 15% of the study sample to avoid overrepresentation). Restricting recruitment by age will enable this study to have greater statistical power. For clarity of etiologic inference, the study will assess the risk of new-onset vasculopathy not associated with pregnancy and thus exclude patients who are pregnant or with a history of CBD/CVD. To eliminate confounding by ART regimen, patients

For peer review only - http://bmjopen.bmj.com/site/about/guidelines.xhtml

must initiate on standard first-line ART (> 90% of ART patients in Blantyre do this). Critically ill patients are excluded primarily for ethical reasons.

Laboratory methods

Surface immunophenotyping of peripheral blood mononuclear cells

Immunophenotyping will be used to characterize peripheral blood mononuclear cells (PBMC) isolated from blood samples of HIV-uninfected and HIV-infected ART initiators. PBMCs will be harvested by density centrifugation using lymphoprep (Axis Shield, UK). PBMCs ($2x10^6$) will be stained with anti-CD45 PerCP CY5.5, anti-CD3 AF700, anti-CD4 BV421, anti-CD8 PE Dazzle, anti-CD38 BV605, anti-HLA-DR APC CY7, anti-CD57 APC, anti-PD1 PE CY7, anti-CTLA4 PE, and anti-CD223 FITC (all from eBiosciences, UK) to determine the expression of these markers on the surface of T-cells. In addition, ($2x10^6$) PBMCs stained with anti-CD16 BV421, anti-CD14 PE, anti-HLA-DR PerCP CY5.5, anti-CD45 AF700, anti-CCR2 BV605, anti-CD11b APC, anti-CX3CR1 PE Dazzle and anti-CD38 FITC (all from eBiosciences, UK) will be used for monocytes. Dead cells, $CD3^+$ T-cells, and $CD56^+$ NK cells will be excluded using: LIVE/DEAD™ Fixable Aqua Dead Cell Stain (Thermofisher, UK), anti-CD3 BV503 and anti-CD56 BV503 (eBiosciences, UK), respectively. Stained cells will be acquired on a BD LSR Fortessa flow cytometer (Becton Dickinson, USA) and data will be analyzed using FlowJo software version 10.0 (Tree Star, San Carlos, CA). For each stained sample analyzed, the median fluorescence intensity (MFI) for each parameter will be normalized to its respective unstained control.

Measurement of soluble markers of immune activation using multiplex bead array

A custom-made multiplex assay will be used to assess soluble markers of monocyte activation (CD163), systemic inflammation (Interleukin-6) and endothelial activation (Intracellular adhesion molecule 1) in plasma, isolated from blood samples of HIV-uninfected and HIV-infected ART initiators. Following isolation, plasma will be aliquoted and stored at -80°C until further use.

For peer review only - http://bmjopen.bmj.com/site/about/guidelines.xhtml

Assessment of exposure to human cytomegalovirus and varicella zoster virus by ELISA

Quantitative VIDAS CMV IgG and IgM (BioMerieux, USA) and VZV glycoprotein IgG Low-Level Enzyme Immunoassay Kit [VaccZyme™EIA], will be used to determine exposure to these viruses using a commercial enzyme-linked immunosorbent assay (ELISA) platform. These kits will detect VZV antigen to a sensitivity and specificity of 97.8% and 96.8% respectively and for CMV, 97.2% and 100% for IgG and 100% and 97.4% for IgM respectively[34 35]. Plasma samples from HIV-uninfected and HIV-infected ART initiators stored at -80°C following collection will be used for these assessments

HIV

HIV infection will be diagnosed using two rapid tests in parallel, EIA rapid tests (Determine HIV-1/2 [Abbott Laboratories, USA] and Uni-Gold HIV [Trinity Biotech PLC, Ireland]), will be used as a tiebreak). HIV-1 RNA levels in plasma will be measured using the Abbott Real-Time HIV-1 assay with a lower limit of detection of 150 copies/mL (Abbott Molecular, Germany), according to the manufacturer's instructions. CD4+ T -cell count measurements will be performed using BD FACS Count machine (Partec platform).

<u>Procedures</u>

Carotid-femoral pulse wave velocity (PWV)[36] and carotid intima-media thickness (CIMT)[37] measurement will be performed in accordance with expert consensus guidelines, using a standardized study protocol on the Vicorder system (SMART Medical, UK) and Philips CX50 machine (Philips healthcare, UK) respectively. CIMT measurements will be performed by three trained operators. The intra-class correlation coefficient will be used to assess the performance of the operators against that of a certified neurosonologist prior to study commencement.

For peer review only - http://bmjopen.bmj.com/site/about/guidelines.xhtml

Outcomes

Primary outcomes

Primary outcomes are the progression of surrogate markers of CBD/CVD, namely PWV and CIMT as well as the occurrence of new-onset vasculopathy defined by threshold values outlined in Table 2.

Secondary outcomes

Secondary outcomes are the following clinical events: 1) stroke, 2) myocardial infarction (MI), 3) unstable angina, 4) peripheral vascular disease (PVD), 5) all-cause death/ vascular death and 6) immune reconstitution inflammatory syndrome (IRIS) vasculopathy (Table 2). Changes in PWV or endothelial activation at 6 months post ART initiation will be interpreted as a subclinical vascular IRIS event. These outcomes will be assessed through active surveillance in QECH inpatient wards for admissions of study participants. To improve capture of clinical outcomes, we will conduct brief telephone interviews with study participants about CBD/CVD symptoms and hospitalizations between study visits and facilitate unsolicited participant self-report. Clinical events and deaths in study participants will be reviewed by an independent endpoint review committee (ERC), comprising of clinicians experienced in Endpoint review. Each event will be reviewed and adjudicated by the ERC Chair and 2 ERC reviewers, using a standard set of diagnostic criteria (Table 2 and Supplement – S1). The format of reporting will be based on modifications of the INSIGHT network clinical diagnostic criteria. Deaths will be reviewed by the ERC using the CoDe approach[23]. For death with limited clinical data, a validated verbal autopsy will be performed to ascertain the cause[38].

Exposures

The exposure for Primary Objective 1 will be HIV status. Yearly HIV rapid tests in HIV-uninfected adults will be performed to exclude those with new HIV infections (Figure 2).

Potential confounding and mediating factors will be recorded in study participants. This will include demographic factors, lifestyle and behavioral factors (e.g. cigarette smoking and alcohol consumption), chronic co-morbidities (i.e. hypertension, diabetes), cardiometabolic,

For peer review only - http://bmjopen.bmj.com/site/about/guidelines.xhtml

renal and hematological factors (i.e. full blood count, creatinine in urine and serum, body-mass-index, waist-to-hip ratio, random glucose, HbA1c, and lipid profile). Blood pressure will be measured at all study visits. Although vascular immune reconstitution inflammatory syndrome (IRIS) (Table 2) will be considered as a primary endpoint, non-vascular IRIS will be defined as a risk factor. Where feasible, we will conduct PCR tests for common causes of IRIS in blood or cerebrospinal fluid (CSF) samples. Adherence to ART and change of ART regimen will be assessed at all study visits through extraction of data from 'ART master cards'; this is a government-supported monitoring tool used by all patients on ART, in Malawi.

For Objective 2a-2c, markers of herpes and HIV viral antigenemia and immune inflammation will be measured according to the outline in Table 1. For primary objective 2a, reactivated latent herpes viral infections will be assessed by quantification of VZV, and CMV antibodies. We will estimate the risk of atherosclerosis and arterial stiffening associated with current herpesviruses reactivation at baseline, and sustained reactivation (i.e. those that continue to have a high titer from measurement at baseline to 6 months after ART initiation). Hyperactivation of B cells may result in an expansion of polyclonal antibodies and thus an overestimation of virus-specific antibody titers. To address this issue and make appropriate adjustments for hypergammaglobulinemia we will 1) measure more than one herpesviruses and 2) measure total IgG.

For primary objective 2b, markers of immune activation, inflammation, and endothelial activation will be measured (Figures 1 & Table 1). Quantitative cell surface immunophenotyping will be performed for CD4+ and CD8+ T-cell activation (e.g. HLA-DR) and senescence (e.g. CD57) in a subset of participants. In all study participants, at baseline, 6, 12, months, we will measure soluble markers associated with systemic inflammation and endothelial activation.

For primary objective 2c, incomplete viral response and viral rebound of HIV will be measured by quantitative PCR in patients on ART.[39] HIV viral load will be measured in patients on ART at 0, 6 and 12 months.

For peer review only - http://bmjopen.bmj.com/site/about/guidelines.xhtml

Data Collection Between May 2017 and August 2020

The two-stage screening will be conducted to find and recruit potential study participants. A trained field worker will first screen to assess eligibility for criteria (a)-(c) in pre-ART counseling sessions, and in individuals from randomly selected households in the community. Eligible participants will then be referred to QECH to complete screening for criteria (d)-(i) and if eligible, consented to participate in the study. At study visits, a tablet-based, standardized Open Data Kit (ODK) case report form (CRF) will be administered in one-on-one interviews by a study nurse to capture demographic and clinical data. Study data will be collected as outlined in Table 1. Daily upload of electronic data will occur with oversight from the data manager at MLW. We will collect up to 30ml of whole blood. An ACR dipstick test will be used to test for creatinine, proteinuria, and glucosuria. In a subset of participants, an electrocardiogram supported by a digital platform and echocardiogram will be performed at baseline, 6 and 24 months, as well as in any participant experiencing a clinical event suggestive of a cardiac etiology. To facilitate the retention and clinical referrals of participants, contact will be made every 3 months to assess the occurrence of clinical events. Participants who miss a scheduled study visit will be contacted by phone and/or visited at home to assess their willingness to maintain their participation and to record intervening clinical events. Recording and definitions of other clinical events, including HIV associated events will be evaluated by the ERC chair. SMS messages will be used for appointment reminders. Technical appendix, statistical code, and dataset will be made available from a data repository, after publication of our work.

Sample Size and Statistical Analysis

The required sample size for the study's primary objectives is 800 HIV-infected patients and 190 HIV-uninfected adults using standard, normal distribution approximation sample size formulas for comparing proportions in two groups of unequal size and based on the following assumptions: **a)** 18.4% of HIV positive study participants have abnormal PWV at baseline. In our ongoing studies of vasculopathy in HIV-infected patients, 18.4% aged $\geq$35 years have a PWV (>12 m/s), **b)** 20% of both HIV-infected patients and HIV-uninfected adults will be lost to follow-up, including by death and HIV sero-conversion[40 41]. **c)** The minimum relative risk (RR) of interest is 2 for Objective 1 and 1.8 for Objective 2. **d)** Cumulative risk of

For peer review only - http://bmjopen.bmj.com/site/about/guidelines.xhtml

clinically significant vasculopathy over study follow-up is 18.4%. This is based on study data cited in (a). **e)** For objectives 2a)-c)**,** the exposure prevalence for each risk factor is 20%. **f)** Statistical tests will have 80% power based on a 2-sided test with; α=0.05. Testing of hypotheses for the secondary outcome will be exploratory. However, we estimate 26 strokes (4 mimics), an unknown number of MIs and 80 deaths occurring during the study[7 42].

The reporting of this study will be prepared in accordance with the STROBE guidelines[43]. Summary and descriptive statistics will be tabulated for all primary and secondary outcome variables, as well as for exposure variables and potential confounding or mediating factors. Time plots for all outcome variables will be inspected. Quantitative data analysis will be conducted to assess the primary outcomes.

There will be 3 analysis time points: 1) after recruitment has finished and baseline data is available for all participants (baseline analysis), 2) once every participant has completed 6 months in the study (6-month analysis) and 3) at 36 months, when each participant has completed 24 months in the study (final analysis).

The baseline analysis will largely consist of descriptive statistics on participant characteristics and data recorded at baseline. Simple regression models will also be used to investigate relationships between exposure and outcome variables measured at baseline. Unadjusted analyses will consist of paired t-tests or Wilcoxon signed rank tests (depending whether the data are normally distributed or not) for continuously measured variables and Chi-Squared or Fisher's exact tests (depending on contingency table cell counts) for binary and categorical variables. Adjusted analyses will be conducted using generalised linear models (GLMs).

The 6-month analysis will be limited in score and serves 2 purposes: 1) characterise new onset vasculopathy in HIV-infected participants that have initiated ART treatment at baseline (vascular IRIS) and 2) define vasculopathy outcomes for the final analysis. The main analysis of the study data happens at the final analysis time point.

For peer review only - http://bmjopen.bmj.com/site/about/guidelines.xhtml

For objective 1 we will develop three regression models. Two GLMs will be developed to compare mean progression of arterial damage from baseline in HIV-infected ART patients and HIV-uninfected adults. These models will regress change from baseline in PWV, respectively cIMT, on HIV status. We will develop a third model to estimate the RR and population attributable fraction of new-onset arterial damage in HIV-infected patients compared to HIV-uninfected adults.

For objective 2a, a set of GLMs will be developed to compare mean progression of vasculopathy in HIV-infected ART patients with and without reactivated latent herpes viral infection. These models will regress change from baseline in PWV, respectively cIMT, on two log-transformed variables for antibody titres of CMV and VZV, respectively.

For objective 2b, we will again fit a set GLMs, with change from baseline in PWV as response variable, this time to investigate if, in HIV-infected ART patients, there is an association between progression of vasculopathy and immune activation and inflammation biomarkers (IL-6, ICAM, CD163). Specifically, for each marker, we will regress PWV on marker quantiles. After having built models for each marker, we will then develop comprehensive multiple regression models for PWV and cIMT with multiple independent markers as predictor variables.

For objective 2c, we will proceed as for objective 2a, but comparing HIV-infected ART patients with incomplete virological suppression or virological resurgence of HIV to those with suppressed HIV plasma viral load.

In addition to these analyses, given the repeated measurements for PWV, immune activation, inflammation markers, we will extent the GLMs for PWV to linear mixed models taking full account of the longitudinal nature of the data. Mixed models will also handle deviations from the visit schedule in a principled fashion and use all available data for drop-

For peer review only - http://bmjopen.bmj.com/site/about/guidelines.xhtml

outs. In the case a log link function is required for PWV in the GLMs, we will fit marginalised models using GEE instead of the LMMs.

For secondary study objectives, we will use univariate methods to assess the frequency of clinical events within exposure strata. If there are sufficient numbers of clinical events we will develop Poisson or negative binomial regression models (depending on model fit) for each clinical event type to compare exposure-defined participants.

We will also use time-to-event models, specifically Cox proportional hazard models, to investigate associations between all-cause mortality and exposures.

As part of exploratory analyses, we will aim to identify risk groups that are potentially incompletely captured with the measured exposure variables. We will perform unsupervised group-based multi-trajectory modeling of multivariate longitudinal patient trajectories to confirm any associations we have found using more traditional approaches[44].

All efforts will be made to collect complete data on all study participants. However, there will inevitably be missing data due to drop-outs and a variety of other reasons. All primary analyses will be performed using multiple imputation. For sensitivity analyses, we will use all-available-cases (AAC), direct likelihood and fully Bayesian models and, for GEE models, weighted GEE. If the number of missingness patterns is sufficiently small, we will also use pattern mixture models which can be used under the general missing-not-at-random setting but make additional identification assumptions.

PATIENT PUBLIC INVOLVEMENT

The global burden of HIV associated CBD and CVD has tripled over the last two decades with the greatest impact in sub-Saharan Africa. CBD and CVD are a priority for patients in Malawi as HIV infection is endemic and the population are living for longer. Knowledge of this, informed our research question with the aim of understanding the mechanisms and thus

For peer review only - http://bmjopen.bmj.com/site/about/guidelines.xhtml

direct targeted novel therapies to reduce this burden. Patients will be involved in the recruitment of the study, but not in the design. Patients and their advisors will be thanked for contributing to the study.

ETHICS AND DISSEMINATION

Written informed consent will be obtained from all study participants, either written or witnessed verbal consent with thumbprint if the participant is non-literate. Study data will be maintained in an encrypted and password protected database to which only study staff will have access. Study participants who develop a clinical event will be managed, using the hospital guidelines, by our study clinician alongside the hospital doctor. Clinical data will be anonymized using unique identifying code. Study data will be kept for 10 years and then destroyed with a record, as recommended by good clinical practice guidelines. This protocol was approved by the ethics committees at University of Malawi College of Medicine (Protocol P02/16/1874) and the Liverpool School of Tropical Medicine (Protocol 16-014).

DISCUSSION

African regions continue to bear the brunt of HIV infection, in 2013, an estimated 8.5 million adults were receiving ART[45]. As the landscape evolves, this population will live longer with stable HIV infection but likely remain at an increased risk of CBD/CVD compared to HIV-uninfected individuals of a similar age and sex. This study will be the first to determine the extent to which HIV reactivation of herpesvirus infection and inflammation contribute to CBD/CVD risk in an adult African population starting ART. The results of this work could potentially open avenues for novel anti-inflammatory and anti-viral interventions for the primary prevention of CBD/CVD in HIV populations in Africa.

ACKNOWLEDGMENTS

The authors would like to thank Agbor Ako and Maria Davy from Research and Development, GlaxoSmithKline and the NCD Africa Open Lab of GlaxoSmithKline review committee for providing valuable advice for this protocol. The authors would like to thank

For peer review only - http://bmjopen.bmj.com/site/about/guidelines.xhtml

For peer review only

BA, MC, LH, THC, JVO, NT for their contribution to the End Point Review Committee, RD and EJ for radiology training and quality control, EZS for providing an electrocardiogram platform and for his cardiology review, VK for input with the echocardiogram protocol, and TS, JM, KM, MN for their input in the advanced drafts of the manuscript. We also extend our gratitude to the INSIGHT network for sharing their clinical endpoint criteria. LB is supported by an NIHR Clinical Lecturer Fellowship. SP is supported by an MRC (UK) core funding MC_UU_12023/23.

AUTHORS' CONTRIBUTIONS

LB and IP developed the first draft. HM, NN, KJ, CK, LA, JKT, SP, MH, JVO, RH had major input for the revision of the second draft. JH is the project manager for RHICCA with oversight from LB, IP, and HM. MH contributed to the statistical methods. LB, JKT, JVO contributed to the clinical training. SP chaired the End point Review Committee.

FUNDING STATEMENT

Funding for this study was provided by the GlaxoSmithKline Africa Non-Communicable Disease Open Lab Grant (Project Number: 7964)

COMPETING INTERESTS

SLP has academic grants from Sysmex Corporation, Gilead Sciences, and ViiV Healthcare. All other authors have no competing interest.

For peer review only

BMJ OPEN

REFERENCES

1. Roth GA, Johnson C, Abajobir A, et al. Global, Regional, and National Burden of Cardiovascular Diseases for 10 Causes, 1990 to 2015. *J Am Coll Cardiol* 2017;70(1):1-25. doi: 10.1016/j.jacc.2017.04.052
2. Organization WH. Global Update on HIV Treatment 2013: Results, Impact and Opportunities. WHO Report. Kuala Lumpure, Malaysia, 2013.
3. Macro NSONaI. Malawi Demographic and Health Survey 2010. Zomba,Malawi, and Calverton, Maryland, USA: NSO and ICF Macro, 2010.
4. Freiberg MS, Chang CC, Kuller LH, et al. HIV infection and the risk of acute myocardial infarction. *JAMA Intern Med* 2013;173(8):614-22. doi: 10.1001/jamainternmed.2013.3728

1659742 [pii] [published Online First: 2013/03/06]
5. Currier JS, Lundgren JD, Carr A, et al. Epidemiological evidence for cardiovascular disease in HIV-infected patients and relationship to highly active antiretroviral therapy. *Circulation* 2008;118(2):e29-35. doi: 10.1161/CIRCULATIONAHA.107.189624 [published Online First: 2008/06/21]
6. Butt AA, Chang CC, Kuller L, et al. Risk of heart failure with human immunodeficiency virus in the absence of prior diagnosis of coronary heart disease. *Archives of internal medicine* 2011;171(8):737-43. doi: 10.1001/archinternmed.2011.151 [published Online First: 2011/04/27]
7. Benjamin LA, Corbett EL, Connor MD, et al. HIV, antiretroviral treatment, hypertension, and stroke in Malawian adults: A case-control study. *Neurology* 2016;86(4):324-33. doi: 10.1212/WNL.0000000000002278
8. Allain TJ, Kinley L, Tsidya B, et al. The spectrum of heart disease in adults in Malawi: A review of the literature with reference to the importance of echocardiography as a diagnostic modality. *Malawi Med J* 2016;28(2):61-65. [published Online First: 2016/11/30]
9. Soliman EZ, Juma H. Cardiac disease patterns in northern Malawi: epidemiologic transition perspective. *J Epidemiol* 2008;18(5):204-8. [published Online First: 2008/08/30]
10. Syed FF, Sani MU. Recent advances in HIV-associated cardiovascular diseases in Africa. *Heart* 2013;99(16):1146-53. doi: 10.1136/heartjnl-2012-303177 [published Online First: 2013/05/18]
11. Soliman EZ, Sharma S, Arasteh K, et al. Baseline cardiovascular risk in the INSIGHT Strategic Timing of AntiRetroviral Treatment (START) trial. *HIV Med* 2015;16 Suppl 1:46-54. doi: 10.1111/hiv.12233 [published Online First: 2015/02/26]
12. Benjamin LA, Allain TJ, Mzinganjira H, et al. The Role of Human Immunodeficiency Virus-Associated Vasculopathy in the Etiology of Stroke. *J Infect Dis* 2017;216(5):545-53. doi: 10.1093/infdis/jix340 [published Online First: 2017/09/22]
13. Benjamin LA, Bryer A, Lucas S, et al. Arterial ischemic stroke in HIV: Defining and classifying etiology for research studies. *Neurol Neuroimmunol Neuroinflamm* 2016;3(4):e254. doi: 10.1212/NXI.0000000000000254
14. Nakanjako D, Kiragga A, Ibrahim F, et al. Sub-optimal CD4 reconstitution despite viral suppression in an urban cohort on antiretroviral therapy (ART) in sub-Saharan Africa: frequency and clinical significance. *AIDS Res Ther* 2008;5:23. doi: 10.1186/1742-6405-5-23 [published Online First: 2008/10/30]
15. Pawelec G, Derhovanessian E. Role of CMV in immune senescence. *Virus Res* 2011;157(2):175-9. doi: 10.1016/j.virusres.2010.09.010 [published Online First: 2010/09/28]
16. Appay V, Sauce D. Immune activation and inflammation in HIV-1 infection: causes and consequences. *J Pathol* 2008;214(2):231-41. doi: 10.1002/path.2276 [published Online First: 2007/12/29]
17. Shaw AC, Joshi S, Greenwood H, et al. Aging of the innate immune system. *Curr Opin Immunol* 2010;22(4):507-13. doi: 10.1016/j.coi.2010.05.003 [published Online First: 2010/07/30]

For peer review only - http://bmjopen.bmj.com/site/about/guidelines.xhtml

18. Hearps AC, Angelovich TA, Jaworowski A, et al. HIV infection and aging of the innate immune system. *Sex Health* 2011;8(4):453-64. doi: 10.1071/SH11028 [published Online First: 2011/12/01]
19. Kovacs EJ, Palmer JL, Fortin CF, et al. Aging and innate immunity in the mouse: impact of intrinsic and extrinsic factors. *Trends Immunol* 2009;30(7):319-24. doi: 10.1016/j.it.2009.03.012 [published Online First: 2009/06/23]
20. Hansson GK. Inflammation, atherosclerosis, and coronary artery disease. *The New England journal of medicine* 2005;352(16):1685-95. doi: 10.1056/NEJMra043430 [published Online First: 2005/04/22]
21. Boekholdt SM, Stroes ES. The interleukin-6 pathway and atherosclerosis. *Lancet* 2012;379(9822):1176-8. doi: 10.1016/S0140-6736(12)60361-4 [published Online First: 2012/03/17]
22. Duprez DA, Neuhaus J, Kuller LH, et al. Inflammation, coagulation and cardiovascular disease in HIV-infected individuals. *PloS one* 2012;7(9):e44454. doi: 10.1371/journal.pone.0044454 [published Online First: 2012/09/13]
23. Lundgren JD, Babiker AG, Gordin F, et al. Initiation of Antiretroviral Therapy in Early Asymptomatic HIV Infection. *N Engl J Med* 2015;373(9):795-807. doi: 10.1056/NEJMoa1506816 [published Online First: 2015/07/21]
24. Eggena MP, Barugahare B, Okello M, et al. T cell activation in HIV-seropositive Ugandans: differential associations with viral load, CD4+ T cell depletion, and coinfection. *The Journal of infectious diseases* 2005;191(5):694-701. doi: 10.1086/427516 [published Online First: 2005/02/03]
25. Mussini CL, P.; Cozzi-Lepri,A.; Lapadula,G.; Marchetti,G.; Nicastri,E.; Cingolani,A.; Lichtner,M.;Antinori,A.; Gori,A.; Monforte, A. CD4/CD8 ratio normalisation and non-AIDS-related events in individuals with HIV who achieve viral load suppression with antiretroviral therapy: an observational cohort study. *Lancet HIV* 2015;2:e98–e106.
26. Sereti I, Krebs SJ, Phanuphak N, et al. Persistent, Albeit Reduced, Chronic Inflammation in Persons Starting Antiretroviral Therapy in Acute HIV Infection. *Clin Infect Dis* 2017;64(2):124-31. doi: 10.1093/cid/ciw683 [published Online First: 2016/10/16]
27. Brunner S, Herndler-Brandstetter D, Weinberger B, et al. Persistent viral infections and immune aging. *Ageing Res Rev* 2011;10(3):362-9. doi: 10.1016/j.arr.2010.08.003 [published Online First: 2010/08/24]
28. Moss P. The emerging role of cytomegalovirus in driving immune senescence: a novel therapeutic opportunity for improving health in the elderly. *Curr Opin Immunol* 2010;22(4):529-34. doi: 10.1016/j.coi.2010.07.001 [published Online First: 2010/08/06]
29. Appay V, Rowland-Jones SL. Premature ageing of the immune system: the cause of AIDS? *Trends Immunol* 2002;23(12):580-5. [published Online First: 2002/12/05]
30. Schaftenaar E, Verjans GM, Getu S, et al. High seroprevalence of human herpesviruses in HIV-infected individuals attending primary healthcare facilities in rural South Africa. *PloS one* 2014;9(6):e99243. doi: 10.1371/journal.pone.0099243 [published Online First: 2014/06/11]
31. Hunt PW, Martin JN, Sinclair E, et al. Valganciclovir reduces T cell activation in HIV-infected individuals with incomplete CD4+ T cell recovery on antiretroviral therapy. *J Infect Dis* 2011;203(10):1474-83. doi: 10.1093/infdis/jir060 [published Online First: 2011/04/20]
32. Corbett EL. Intensified HIV/TB prevention linking home-based HIV testing, including the option of selftesting, with HIV care. ISRCTN02004005. London: ISRCTN, 2012.
33. Roth GA, Huffman MD, Moran AE, et al. Global and regional patterns in cardiovascular mortality from 1990 to 2013. *Circulation* 2015;132(17):1667-78. doi: 10.1161/CIRCULATIONAHA.114.008720
34. Maple PA, Breuer J, Quinlivan M, et al. Comparison of a commercial Varicella Zoster glycoprotein IgG enzyme immunoassay with a reference time resolved fluorescence immunoassay (VZV TRFIA) for measuring VZV IgG in sera from pregnant women, sera sent for confirmatory

For peer review only - http://bmjopen.bmj.com/site/about/guidelines.xhtml

testing and pre and post vOka vaccination sera from healthcare workers. *J Clin Virol* 2012;53(3):201-7. doi: 10.1016/j.jcv.2011.12.010 [published Online First: 2012/01/21]
35. Carlier P, Harika N, Bailly R, et al. Laboratory evaluation of the new Access (R) cytomegalovirus immunoglobulin IgM and IgG assays. *J Clin Virol* 2010;49(3):192-7. doi: 10.1016/j.jcv.2010.07.024 [published Online First: 2010/08/31]
36. Laurent S, Cockcroft J, Van Bortel L, et al. Expert consensus document on arterial stiffness: methodological issues and clinical applications. *Eur Heart J* 2006;27(21):2588-605. doi: 10.1093/eurheartj/ehl254 [published Online First: 2006/09/27]
37. Touboul PJ, Hennerici MG, Meairs S, et al. Mannheim carotid intima-media thickness and plaque consensus (2004-2006-2011). An update on behalf of the advisory board of the 3rd, 4th and 5th watching the risk symposia, at the 13th, 15th and 20th European Stroke Conferences, Mannheim, Germany, 2004, Brussels, Belgium, 2006, and Hamburg, Germany, 2011. *Cerebrovasc Dis* 2012;34(4):290-6. doi: 10.1159/000343145

000343145 [pii] [published Online First: 2012/11/07]
38. Serina P, Riley I, Stewart A, et al. Improving performance of the Tariff Method for assigning causes of death to verbal autopsies. *BMC Med* 2015;13:291. doi: 10.1186/s12916-015-0527-9 [published Online First: 2015/12/09]
39. Organization WH. Consolidated ARV guidelines 2013 [Available from: http://www.who.int/hiv/pub/guidelines/arv2013/art/artmonitoring/en/index4.html accessed 15 Oct 2015.
40. Misiri HE, Edriss A, Aalen OO, et al. Estimation of HIV incidence in Malawi from cross-sectional population-based sero-prevalence data. *Journal of the International AIDS Society* 2012;15(1):14. doi: 10.1186/1758-2652-15-14 [published Online First: 2012/03/16]
41. MacPherson P, Houben RM, Glynn JR, et al. Pre-treatment loss to follow-up in tuberculosis patients in low- and lower-middle-income countries and high-burden countries: a systematic review and meta-analysis. *Bull World Health Organ* 2014;92(2):126-38. doi: 10.2471/BLT.13.124800 [published Online First: 2014/03/14]
42. Walker R, Whiting D, Unwin N, et al. Stroke incidence in rural and urban Tanzania: a prospective, community-based study. *Lancet Neurol* 2010;9(8):786-92. doi: S1474-4422(10)70144-7 [pii]

10.1016/S1474-4422(10)70144-7 [published Online First: 2010/07/09]
43. von Elm E, Altman DG, Egger M, et al. The Strengthening the Reporting of Observational Studies in Epidemiology (STROBE) Statement: guidelines for reporting observational studies. *International journal of surgery* 2014;12(12):1495-9. doi: 10.1016/j.ijsu.2014.07.013 [published Online First: 2014/07/22]
44. Nagin DS, Jones BL, Passos VL, et al. Group-based multi-trajectory modeling. *Stat Methods Med Res* 2018;27(7):2015-23. doi: 10.1177/0962280216673085 [published Online First: 2018/05/31]
45. Organization WH. Global Update on the Health Sector Response to HIV 2014. Geneva: World Health Organization, 2014.

For peer review only

For peer review only - http://bmjopen.bmj.com/site/about/guidelines.xhtml

BMJ OPEN

Figure 1. Hypothetical pathway of the interplay between chronic viruses, immune activation, systemic inflammation, endothelial activation, and vasculopathy.

Figure 2. Outline of study design for a 36-month cohort study

For peer review only

For peer review only - http://bmjopen.bmj.com/site/about/guidelines.xhtml

BMJ OPEN

Table 1. Laboratory tests and clinical procedures in ART patients and HIV-uninfected adults

	Study Time Points						
	Baseline	6 months	12 months	18 months	24 months	30 months	36 months
**Clinical Procedures**							
PWV	X	X	X	X	X	X	X
CIMT	X				X		
ABPI	X	X	X	X	X	X	X
Cardiac Echo *(participant sub-set)*	X				X		
ECG *(participant sub-set)*	X				X		
**Cardiometabolic markers**							
Creatinine	X				X		
Full Blood Count	X	X					
Cholesterol (LDL, HDL, Triglycerides)	X				X		
Serum glucose/HBA1C	X				X		
**HIV Infection and Progression**							
HIV viral load *(HIV patients)*	X	X	X				
CD4 count *(HIV patients)*	X	X	X				
HIV rapid test *(controls)*	X		X		X		X
**Immune dysregulation**							
Soluble markers of systemic inflammation	X	X	X				
Soluble markers of endothelial activation	X	X	X				
CD8 and CD4 T-cell activation and senescence *(participant subset)*	X	X	X		X		X
Monocyte/ Macrophage activation and senescence *(participant subset)*	X	X	X		X		X
Herpesviruses infection							
CMV IgG	X	X					
VZV IgG	X	X					

For peer review only

Table 2: Case definitions of primary and secondary endpoints for the study

	Type	Definitions
**Primary Endpoint**	Carotid intimal medial thickness (CIMT)	The occurrence of new-onset vasculopathy [CIMT – a measure of atherosclerosis]: CIMT >0.9 mm or >75th percentile of age/sex references values or presence of plaque on the carotid scan<br><br>Progression: total change in CIMT at 24 months from baseline
	Pulse wave velocity (PWV)	Occurrence of new onset vasculopathy [PWV – a measure of arterial stiffness]: PWV >12[m/s]<br><br>Progression: total change in PWV at 24 months from baseline
**Secondary endpoint**	Stroke	**Confirmed (1+2) or 3 or 4 or 5:**<br>1. Acute onset with a clinically compatible course, including unequivocal objective findings of a localizing neurologic deficit<br>2. CT or MRI compatible with a diagnosis of stroke and current neurologic signs and symptoms<br>3. Stroke diagnosed as the cause of death at autopsy<br>4. Compatible clinical history and positive lumbar puncture compatible with subarachnoid hemorrhage<br>5. Death certificate or death note from medical record listing stroke as the cause of death
	Myocardial Infarction [MI]	**Confirmed: One of the following 5 criteria (adapted from 2007 Universal Definition of Myocardial Infarction): [1 + (2 or 3 or 6)] or 4 or 5**<br><br>1. Rise and/or fall of cardiac biomarkers (preferably troponin), with at least one value above the 99th percentile of upper reference limit (URL);<br>2. The occurrence of a compatible clinical syndrome, including symptoms consistent with myocardial ischemia;<br>3. ECG changes indicative of new ischemia (new ST-changes or new left bundle branch block [LBBB]), or development of pathological Q waves on the ECG;<br>4. Sudden unexpected cardiac death involving cardiac arrest before biomarkers are obtained or before a time when biomarkers appear, along with (a) new ST-changes or new LBB, or (b) evidence of fresh thrombus on coronary angiography or at autopsy<br>5. Pathologic findings of acute myocardial infarction (including

For peer review only - http://bmjopen.bmj.com/site/about/guidelines.xhtml

BMJ OPEN

[redacted]		acute MI demonstrated as the cause of death on autopsy)<br>6. Development of a) evolving new Q waves, or b) evolving ST elevation, preferably based on at least 2 ECGs taken during the same hospital admission
	Coronary artery disease requiring drug treatment	**Confirmed (1 or 2) + 3:**<br><br>1. Evidence of myocardial ischemia based on either diagnostic imaging (such as a stress echocardiogram or thallium scan) or diagnostic changes on an electrocardiogram (such as during stress testing or an episode of chest pain)<br>2. Evidence of coronary artery disease based on coronary angiography or other diagnostic imaging<br>3. Use of medication given to treat or prevent angina (e.g., nitrates, beta blockers, calcium channel blockers)
	Peripheral vascular disease [PVD]	**Confirmed (1+2) or (1+3):**<br>1. Compatible clinical signs and symptoms<br>2. Positive results on diagnostic imaging studies (e.g., Doppler ultrasound, contrast arteriography, MRI arteriography); 3. Ankle Brachial Pressure Index < 0.90 in non-diabetics
	Vascular Immune reconstitution syndrome (IRIS)	A new onset vasculopathy within 6 months of starting ART
	All-cause death and vascular-related deaths	Death (of any or vascular cause) that occurs after recruitment into the study

For peer review only - http://bmjopen.bmj.com/site/about/guidelines.xhtml

For peer review only

For peer review only - http://bmjopen.bmj.com/site/about/guidelines.xhtml

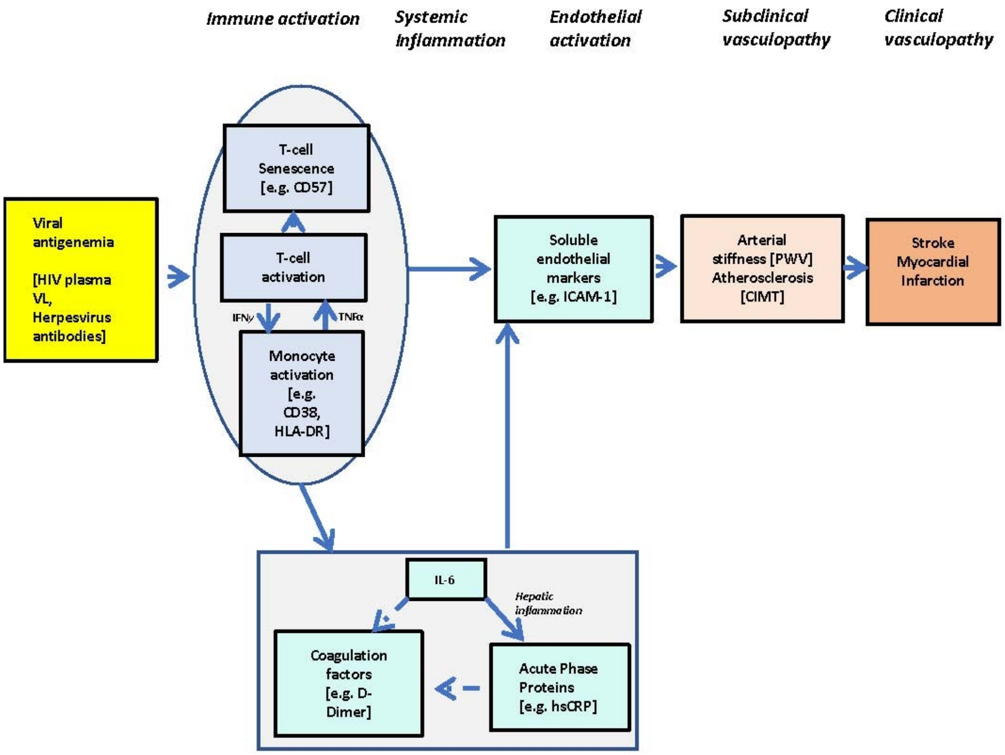

Figure 1

90x90mm (300 x 300 DPI)

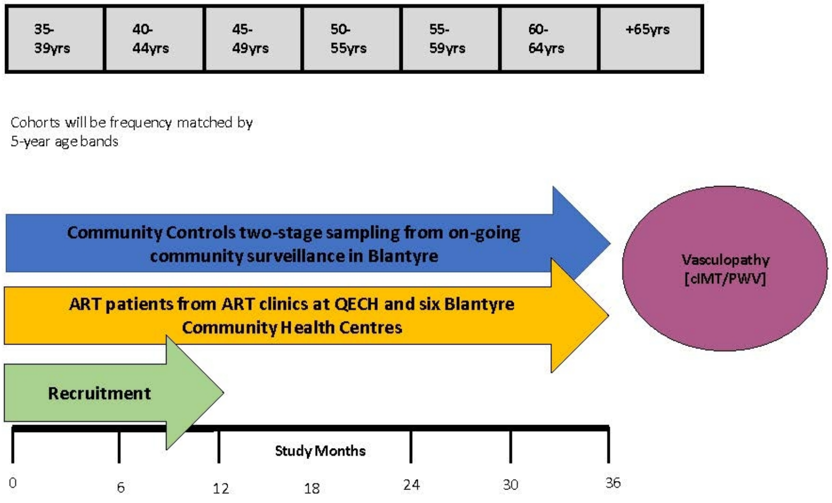

Figure 2

90x90mm (300 x 300 DPI)

RHICCA – Clinical endpoint diagnostic criteria vs 3.5 Apr 2018

RHICCA
Reactivation of herpesviruses and inflammation as cardiovascular and cerebrovascular risk factors in ART initiators

	CONFIRMED	PROBABLE
**INFECTIONS**		
Aspergillosis, invasive pulmonary	**Confirmed: 1 + 2 + 3:**<br>1. Compatible clinical course **(Appendix 13)**,<br>2. CXR abnormality compatible with aspergillosis;<br>3. invasive mycelia consistent with Aspergillus on lung biopsy or positive culture of lung tissue or **positive culture of sputum** collected by any method	**Probable: 1 + 2 + 3:**<br>1. Compatible clinical course **(Appendix 13)**,<br>2. CXR abnormality compatible with aspergillosis;<br>3. invasive mycelia consistent with Aspergillus on tissue biopsy or positive culture at a normally sterile site (e.g., blood) apart from the lungs.
Aspergillosis, other invasive	**Confirmed: 1 + 2 + 3:**<br>1.compatible clinical course **(Appendix 11)**,<br>2. invasive mycelia consistent with Aspergillus on tissue biopsy or clinical evidence of infection,<br>3. positive culture from the affected tissue	**Probable: 1 + 2:**<br>1.clinical evidence of invasive infection **(Appendix 11)**, 2.invasive mycelia consistent with Aspergillus on tissue biopsy or positive culture at a normally sterile site (e.g., blood) apart from the involved tissue
Bartonellosis	**Confirmed 1+ 2:**<br>1.Clinical or histologic evidence of bacillary angiomatosis or bacillary peliosis,<br>2. a positive culture or PCR for *B. quintana* or *B. henselae*	**Probable 1 + 2:**<br>1.Clinical evidence of bacillary angiomatosis or bacillary peliosis **(Appendix 12)**,<br>2. positive silver stain for bacilli from a skin lesion or an affected organ
Candidiasis,oral	**Confirmed:1 + 2 + 3:**<br>1. Macroscopic appearance on examination of the mouth<br>2. microscopic evidence of yeasts or pseudo hyphae<br>3. no evidence of oesophageal involvement -	**Probable: 1 + 2 + 3:**<br>1. a clinical diagnosis of oral candidiasis and/or microscopic evidence of yeasts or pseudo hyphae<br>2. clinical response to treatment<br>3. no evidence of oesophageal involvement
Candidiasis of bronchi, trachea, or lungs	**Confirmed:1 + 2:**<br>Macroscopic appearance at bronchoscopy or autopsy<br>microscopic evidence of yeasts or pseudo hyphae	**None**
Candidiasis, esophageal	**Confirmed: 1 + 2:**<br>1. Macroscopic appearance at esophagoscopy or autopsy.<br>2. microscopic evidence of yeasts or pseudo hyphae	**Probable: 1 + 2 + 3:**<br>1. Recent onset of retrosternal pain or difficulty on swallowing.<br>2. a clinical diagnosis of oral candidiasis, endoscopic visualization of candidiasis and/or microscopic evidence of yeasts or pseudo hyphae from oropharyngeal mucosa<br>3. clinical response to treatment

For peer review only

RHICCA – Clinical endpoint diagnostic criteria vs 3.5 Apr 2018

	CONFIRMED	PROBABLE
**INFECTIONS (CONTINUED)**		
Cryptococcosis, extrapulmonary (not meningitis)	**Confirmed: 1 or 2 or 3:**<br><br>From tissue other than lung or hilum:<br>1. microscopic demonstration of narrow based budding yeast<br>2. positive culture,<br>3. antigen detention	**None**
Cryptococcosis meningitis	**Confirmed: 1 or 2 or 3 or 4:**<br>1. Brain histopathology microscopic demonstration of narrow based budding yeast<br>2. CSF evidence of India ink test<br>3. CSF evidence of positive culture<br>4. CSF evidence of positive antigen detection	**None**
Cryptosporidiosis	**Confirmed: 1 + 2**<br>1. Diarrhea for > 1 month<br>2. positive microscopy	**None**
CMV retinitis	Autopsy demonstration	**Probable 1 + 2:**<br>1. Typical appearance on fundoscopy of discrete patches of retinal whitening, spreading along blood vessels.<br>2. Associated vasculitis, hemorrhage and necrosis, confirmed by ophthalmologist

	CONFIRMED	PROBABLE
**INFECTIONS (CONTINUED)**		
HZV single dermatome	**Confirmed 1+2:**<br>1.multiple ulcerated lesions affecting at least 1 dermatome, and/or 1 or more contiguous dematomes;<br><br>2. positive culture, PCR, or antigen assay from affected tissue	**Probable 1+ 2:**<br>1.multiple typical ulcerated lesions affecting at Least 1 dermatome, and/or 1 or more contiguous dermatomes;<br><br>2. response to an antiviral active against HZV unless resistance is demonstrated
HZV, disseminated	**Confirmed 1+2:**<br>1. multiple ulcerated lesions affecting at least 2 non-contiguous dermatomes, or with generalized cutaneous dissemination HZV involvement of the lung, liver, brain, or other internal organs<br>2. positive culture, PCR, or antigen assay from affected tissue	**Probable 1+2:**<br>1. multiple typical ulcerated lesions affecting at least 2 non-contiguous dermatomes, or with generalized cutaneous dissemination<br>2. response to an antiviral active against HZV unless resistance is demonstrated
HSV mucocutaneous ulceration	**Confirmed 1 +2:**<br>1.Ulceration for > 1 Month<br>2. Histology, culture, PCR, or detection of antigen from affected tissue	**Probable 1 + 2:**<br>1. Typical HSV ulceration for > 1 month,<br>2. response to an antiviral active against HZV unless resistance is demonstrated
Histoplasmosis, disseminated or extrapulmonary	**Confirmed 1+2:**<br>1.Compatible symptoms,<br>2. histology or culture or elevated blood or urine antigen levels	None
Isosporiasis	**Confirmed 1 + 2:**<br>1.Diarrhea for > 1 month<br>2.microscopic identification of *Isospora belli*	None

For peer review only - http://bmjopen.bmj.com/site/about/guidelines.xhtml

RHICCA – Clinical endpoint diagnostic criteria vs 3.5 Apr 2018

RHICCA

Reactivation of herpesviruses and inflammation as cardiovascular and cerebrovascular risk factors in ART initiators

Microsporidiosis	**Confirmed 1 + 2:**<br>1.Diarrhea for > 1 month<br>2.Microscopic identification of Microsporidia	None
MAC and other mycobacterial disseminated diseas	**Confirmed 1 + 2:**<br>1. Fever, fatigue, anemia or diarrhea<br>2.positive culture from blood, body fluids or tissue other than pulmonary, hilar or stool	**Probable 1+2+3:**<br>1. Fever, fatigue, anemia or diarrhea<br>2. **AFB** or positive direct MAC PCR in blood, body fluids or tissue other than pulmonary, hilar or stool<br>3. no concurrent non-pulmonary TB

	**CONFIRMED**	**PROBABLE**	**POSSIBLE**
M. tuberculosis disease, pulmonary	**Confirmed 1+2:**<br>1.Compatible symptoms of fever, dyspnea, cough, weight loss or fatigue<br>2. culture or PCR from **sputum** or bronchial lavage or lung tissue	**Probable 1+2+3+4:**<br>1.Symptoms of fever, dyspnea, cough, weight loss or fatigue<br>2.abnormal chest X-ray,<br>3. AFBs seen in sputum or lavage or lung tissue but not grown in culture,<br>4.responds to treatment	**Possible 1+2+3+4:**<br>1.Symptoms of fever, dyspnea , cough, weight loss or fatigue<br>2.abnormal chest X-ray compatible with pulmonary TB (such as upper lobe cavitation, pleural exudate)<br>3. No other etiology for pulmonary symptoms and signs identified,<br>4.Responds to anti tuberculosis treatment
M. tuberculosis disease, Extrapulmonary (not meningitis)	**Confirmed 1+2:**<br>1. Compatible symptoms<br>2. culture or PCR or MTB Xpert from blood or affected tissue (i.e. pericardial, ascites, and lymph glands)	**Probable 1+2+3**:<br>1.Compatible symptoms<br>2. AFBs seen from affected tissue or blood<br>3.concurrent diagnosis of pulmonary TB or responds to treatment	**Possible 1+2+3:**<br>1.Compatible symptoms<br>2. No other etiology for symptoms and signs identified<br>3.concurrent diagnosis of pulmonary TB or responds to treatment
M. tuberculosis disease, meningitis	**Confirmed 1+2:**<br>**1.** Clinical symptoms of meningism **(Appendix 7)**<br>**2.** Tissue/CSF culture, or PCR, or AFB or MTB Xpert	**Probable 1+ a score ≥12 (Appendix: Table 2):**<br>1. Clinical symptoms of meningism **(Appendix 7)**<br>2. A score ≥12, based on clinical, CSF, cerebral brain imaging criteria or evidence of TB elsewhere	
Nocardiosis	**Confirmed 1+2:**<br>1. Clinical evidence of invasive Infection **(Appendix 8)**<br>2. a positive culture from the affected tissue or blood	**Probable 1+2:**<br>1. Clinical evidence of invasive Infection **(Appendix 8)**<br>2. microscopic evidence of bronchial weakly acid fast organisms from the affected tissue	

For peer review only

For peer review only - http://bmjopen.bmj.com/site/about/guidelines.xhtml

RHICCA – Clinical endpoint diagnostic criteria vs 3.5 Apr 2018

Pneumocystis jirovecii pulmonary	**Confirmed 1+2:**<br>1. compatible clinical syndrome **(Appendix 9)**<br>2. microscopic or histological demonstration of *P. jirovecii* cysts in a pulmonary specimen	Probable 1+2+3+4+5<br>1. dyspnea or cough, or fever progressive over > 1 week<br>2. **diffuse chest x-ray abnormality** or, if on inhalational pentamidine, diffuse upper lung field abnormality<br>3. evidence of hypoxia<br>4. not suggestive of bacterial pneumonia (i.e., not purulent sputum or hemoptysis, no bacterial pathogen identified in blood or bronchial wash)<br>5. response to PcJ treatment
Pneumocystis jirovecii, extrapulmonary	**Confirmed 1+2:**<br>1. compatible clinical syndrome<br>2. microscopic or histological demonstration of *P. jirovecii* cysts in a tissue other than pulmonary specimen	None

For peer review only

RHICCA – Clinical endpoint diagnostic criteria vs 3.5 Apr 2018

RHICCA
Reactivation of herpesviruses and inflammation as cardiovascular and cerebrovascular risk factors in ART initiators

	CONFIRMED	PROBABLE
**INFECTIONS (CONTINUED)**		
Pneumonia, **SINGLE EPISODE (isolated)**<br>bacterial, excludes: (a) post-obstructive pneumonias, including in those with intrapulmonary/bronchial lesions, (b) aspiration pneumonia, including in those with decreased consciousness levels, (c) nosocomial ventilator-associated pneumonias	**Confirmed 1+2+3:**<br>pneumonia episodes must occur after enrollment;<br>1. Signs and symptoms suggestive of bacterial pneumonia (**appendix 10**)<br>2. Focal CXR abnormality compatible with bacterial pneumonia,<br>3. identification of a bacterial pathogen by positive blood culture, positive bronchoscopic sputum culture, detection of Legionella or pneumococcal antigen in urine or blood or diagnostic serologic findings	**Probable 1+2:**<br>pneumonia episodes must occur after enrollment;<br>1.Signs and symptoms suggestive of bacterial pneumonia (**Appendix 10**)<br>2. Focal CXR abnormality compatible with Bacterial pneumonia
Pneumonia, **recurrent**<br>bacterial, excludes: (a) post-obstructive pneumonias, including in those with intrapulmonary/bronchial lesions, (b) aspiration pneumonia, including in those with decreased consciousness levels, (c) nosocomial ventilator-associated pneumonias	**Confirmed 1+2+3+4+5**<br>Second pneumonia episodes must occur after enrollment and after an isolated pneumonia, within 365 days of the first episode and with strong clinical evidence that the first episode was cured;<br>1. Signs and symptoms of second event suggestive of bacterial pneumonia (**Appendix 10**)<br>2. Focal CXR abnormality compatible with bacterial pneumonia,<br>3. identification of a bacterial pathogen by positive blood culture, positive bronchoscopic sputum culture, detection of Legionella or pneumococcal antigen in urine or blood or diagnostic serologic findings<br>4. the second pneumonia had onset of symptoms < 365 days after the first episode<br>5. there is strong evidence that the first episode was cured such as an intervening clear CXR or absence of symptoms after > 1 month off antibacterial effective against pathogens commonly producing pneumonia	**Probable 1+2+3+4:**<br>Second pneumonia episodes must occur after enrollment and after an isolated pneumonia, within 365 days of the first episode and with strong clinical evidence that the first episode was cured;<br>1. Signs and symptoms suggestive of bacterial pneumonia (**Appendix 10**)<br>2. focal CXR abnormality compatible with bacterial pneumonia<br>3. the second pneumonia had onset of symptoms < 365 days after the first episode<br>4. there is strong evidence that the first episode was cured such as an intervening clear CXR or absence of symptoms after > 1 month off antibacterials effective against pathogens commonly producing pneumonia
PML (progressive multifocal leukoencephalopathy)	**Confirmed 1 or 2:**<br>1. positive histology,<br>2. compatible clinical (**Appendix 11**) and radiologic course and positive CSF PCR for JK virus	**Probable 1+2+3:**<br>1. Consistent symptoms (**Appendix 11)**,<br>2. brain image consistent with PML,<br>3. no response to toxo treatment or toxoplasma [illegible]
Salmonella blood stream infection or bacteraemia, isolated	**Confirmed 1:**<br>A septic episode must occur after enrollment;<br>1. Positive blood or tissue culture	None
Salmonella blood stream infection or bacteraemia, recurrent	**Confirmed 1:**<br>A second septic episode must occur after enrollment and after an isolated episode;<br>1. Has met the criteria of isolated Salmonella septicemia<br>2. Positive blood or tissue culture on the second episode<br>3. the second septicemia had onset of symptoms < 365 days after the first episode<br>4. the second septicemia must be due to a different Salmonella serotype or there must be strong evidence that the first episode was cured such as a negative blood culture off effective antibacterials for > 1 week or absence of symptoms off antibacterials for > 1 month	None

For peer review only - http://bmjopen.bmj.com/site/about/guidelines.xhtml

RHICCA – Clinical endpoint diagnostic criteria vs 3.5 Apr 2018

RHICCA
Reactivation of herpesviruses and inflammation as cardiovascular and cerebrovascular risk factors in ART initiators

Toxoplasmosis of brain	**Confirmed 1+2+3:**<br>1. Compatible clinical findings **(Appendix 12)**<br>2. Compatible radiological findings<br>3. Detection of T gondii in the **CSF** or brain tissue (i.e. microscopy or PCR)	**Probable 1+2+3:**<br>1. Symptoms of focal intracranial abnormality or decreased consciousness<br>2. brain image consistent with lesion(s) enhanced by contrast<br>3. positive toxoplasma serology or responds to treatment clinically or by scan

	CONFIRMED	PROBABLE
**NEOPLASMS**		
Cervical carcinoma, invasive	**Confirmed 1:**<br>1. Histology (NOT carcinoma-in-situ)	**None**
Kaposi sarcoma, (mucocutaneous or visceral)	**Confirmed 1:**<br>1. Histology	1. Highly typical appearance<br>2. persistence for > 1 month
Lymphoma, primary, of brain	**Confirmed 1:**<br>1. Histology of brain tissue	**Probable 1+2+3:**<br>1. Symptoms consistent with lymphoma<br>2. at least one CNS lesion with mass effect<br>3. lack of clinical or radiographic response at least 2 weeks of treatment for toxoplasmosis
Lymphoma, Hodgkin's	1. Histology	None
Lymphoma, non-Hodgkin's, all cell types	**Confirmed 1:**<br>1. Histology	None
**NEUROLOGICAL**		
HIV-related encephalopathy (including AIDS Dementia Complex)	None	**Probable 1+2+3+4:**<br>1. Cognitive or motor dysfunction interfering with usual activity, progressive over weeks or months<br>2. no other condition to explain the findings<br>3. brain image obtained and suggests no other causes<br>4. grade 2 or worse impairment in at least 2 domains by NARS (**appendix – table 1**) excluding abnormal domains at trial entry. (For persons with abnormal domains at entry worsening by at least two grades meets criteria.)

For peer review only - http://bmjopen.bmj.com/site/about/guidelines.xhtml

RHICCA – Clinical endpoint diagnostic criteria vs 3.5 Apr 2018

RHICCA
Reactivation of herpesviruses and inflammation as cardiovascular and cerebrovascular risk factors in ART initiators

CARDIOVASCULAR DISEASES		
Acute Myocardial Infarction	**Confirmed: One of the following 5 criteria (adapted from 2007 Universal Definition of Myocardial Infarction): [1 + (2 or 3 or 6)] or 4 or 5**<br>1. Rise and/or fall of cardiac biomarkers (preferably troponin), with at least one value above 99th percentile of upper reference limit (URL);<br>2. Occurrence of a compatible clinical syndrome, including symptoms (such as chest pain **– see Appendix 1**) consistent with myocardial ischemia;<br>3. ECG changes indicative of new ischemia (new ST-changes or new left bundle branch block [LBBB]), or development of pathological Q waves on the ECG;<br>4. Sudden unexpected cardiac death involving cardiac arrest before biomarkers are obtained or before a time when biomarkers appear, along with (a) new ST-changes or new LBB, or (b) evidence of fresh thrombus on coronary angiography or at autopsy<br>5. Pathologic findings of acute myocardial infarction (including acute MI demonstrated as the cause of death on autopsy)<br>6. Development of a) evolving new Q waves, or b) evolving ST elevation, preferably based on at least 2 ECGs taken during the same hospital admission	**Probable 1 and 2:**<br>1. Occurrence of a compatible clinical syndrome (**Appendix 1**), including symptoms (such as chest pain) consistent with myocardial ischemia)<br>2. Development of a) evolving new Q waves, or b) evolving ST elevation, preferably based on at least; ECGs taken during the same hospital admission.
Peripheral vascular disease	**Confirmed (1+2) or (1+3):**<br>1. Compatible clinical signs and symptoms (**see Appendix 3**)<br>2. Positive results on diagnostic imaging studies (e.g., Doppler ultrasound, contrast arteriography, MRI arteriography); 3. Ankle Brachial Pressure Index < 0.90 in non-diabetics	**Probable 1:**<br>1. Compatible clinical signs and symptoms (**see Appendix 3**)
Stroke	**Confirmed (1+2) or 3 or 4 or 5:**<br>1. Acute onset with a clinically compatible course, including unequivocal objective findings of a localizing neurologic deficit **(appendix 4)**;<br>2. CT or MRI compatible with diagnosis of stroke and current neurologic signs and symptoms<br>3. Stroke diagnosed as cause of death at autopsy<br>4. Compatible clinical history and positive lumbar puncture compatible with subarachnoid hemorrhage<br>5. Death certificate or death note from medical record listing stroke as cause of death	**Probable (1+2) or (1+3):**<br>1. Acute onset with a clinically compatible course, including unequivocal objective findings of a localizing neurologic deficit **(appendix 4)**;<br>2. Positive lumbar puncture compatible with subarachnoid hemorrhage<br>3. Death certificate or death note from medical record listing stroke as cause of death
Congestive heart failure	**Confirmed (1+2) or (1+3) or (1+4):**<br>1. Clinical signs and symptoms compatible with left or right sided heart failure [**Appendix 5]** without an alternative explanation<br>2. Hemodynamic measurements, radionucleotide ventriculography, echocardiogram, cardiac catheterization, or multiple gated acquisition scan showing a decreased ejection fraction of < 45%<br>3. Echocardiogram, cardiac catheterization or other studies showing evidence of increased left atrial pressure or right heart failure;<br>4. Elevated levels of Brain Natriuretic Peptide (BNP) or NT-proBNP	**Probable 1+2+3:**<br>1. Clinical signs and symptoms compatible with left or right sided heart failure [**Appendix 5]** without an alternative explanation<br>2. Chest x-ray or other imaging study showing evidence of congestive heart failure, including cardiac enlargement;<br>3. Documentation of treatment for congestive heart failure

For peer review only - http://bmjopen.bmj.com/site/about/guidelines.xhtml

RHICCA – Clinical endpoint diagnostic criteria vs 3.5 Apr 2018

RHICCA
Reactivation of herpesviruses and inflammation as cardiovascular and cerebrovascular risk factors in ART initiators

Coronary artery disease requiring drug treatment	**Confirmed (1 or 2) + 3:**<br><br>1. Evidence of myocardial ischemia based on either diagnostic imaging (such as a stress echocardiogram or thallium scan) or diagnostic changes on an electrocardiogram (such as during stress testing or an episode of chest pain)<br>2. Evidence of coronary artery disease based on coronary angiography or other diagnostic imaging<br>3. Use of medication given to treat or prevent angina (e.g., nitrates, beta blockers, calcium channel blockers)	**Probable 1+2:**<br><br>1. Other evidence of myocardial ischemia and/or coronary artery disease (including that based primarily upon symptoms and clinical presentation, such as chest pain with exertion)<br>2. Use of medication given to treat or prevent angina (e.g., nitrates, beta blockers, calcium channel blockers)
Deep vein thrombosis	**Confirmed 1:**<br>1. Diagnosis of deep vein thrombosis (DVT) by contrast venography, or **ultrasonography** other comparable imaging techniques;	**Probable (1)+2+3:**<br>1. An elevated **D-dimer test**;<br>2. A score on the Wells Clinical Prediction Rule for DVT of ≥ 3 points;<br>3. Absence of alternative diagnosis as likely or greater than that of deep venous thrombosis.<br>**Wells Clinical Prediction Rule for DVT (Appendix 6)**
**SYSTEMIC DISEASES**		
Anaemia<br>NOT FOR ERC<br>TO BE DETERMINED RETROSPECTIVELY	**Confirmed 1**<br>Classified according to both WHO and DAIDS thresholds for severe/grade 3-4 anaemia	
Chronic Kidney disease	**Confirmed: 1 or 2**<br><br>1. Kidney damage for >3 months, as defined by structural or functional abnormalities of the kidney with or without decreased GRF, manifest by either;<br>- Pathological abnormalities; or<br>- Markers of kidney damage, including abnormalities in the composition of the blood or urine or abnormalities in imaging results<br>2. GFR <60mL/min/1.73m2 for >3months, with or without kidney disease (estimated by CKD-EPI)	**Confirmed: 1 or 2**<br><br>1. Isolated Kidney damage, as defined by structural or functional abnormalities of the kidney with or without decreased GRF, manifest by either;<br>- Pathological abnormalities; or<br>- Markers of kidney damage, including abnormalities in the composition of the blood or urine or abnormalities in imaging results<br>2. Isolated GFR <60mL/min/1.73m2, with or without kidney disease (estimated by CKD-EPI)
End-stage renal disease	**Confirmed: 1**<br>1.Hemodialysis or peritoneal dialysis documented in a clinical note for a period of at least three months;	**Probable: 1**<br>1. Hemodialysis or peritoneal dialysis documented in a clinical note for a period of at least one month and up to the time of death in a patient who dies within three months after dialysis begins
Diabetes Mellitus<br><br>NOT FOR ERC<br>TO BE DETERMINED RETROSPECTIVELY	**Confirmed: 1 or 2 or 3 or 4**<br><br>1. Symptoms of diabetes plus casual plasma glucose concentration ≥ 200 mg/dL (11.1 *mmol/L*). (Casual is defined as any time of day without regard to time since last meal. The classic symptoms of diabetes include polyuria and polydipsia.)<br><br>2. Fasting plasma glucose ≥ 126 mg/dL (7.0 *mmol/L*). (Fasting is defined as no caloric intake for at least 8 hours.)<br><br>3. 2-hour post-load glucose ≥ 200 mg/dL (11.1 *mmol/L*) during an oral glucose tolerance test. (The test should be performed as described by WHO, using glucose load containing the equivalent of 75 g anhydrous glucose dissolved in water.)<br><br>4.An **HbA1c** of 48mmol/mol (6.5%) or above.	**None**

For peer review only - http://bmjopen.bmj.com/site/about/guidelines.xhtml

RHICCA – Clinical endpoint diagnostic criteria vs 3.5 Apr 2018

RHICCA
Reactivation of herpesviruses and inflammation as cardiovascular and cerebrovascular risk factors in ART initiators

Decompensate Liver disease	**Confirmed: 1+2**<br>1. Histologic, radiographic, or ultrasound evidence of cirrhosis, as documented by one of the following:<br>a. Histologic evidence of cirrhosis obtained by liver biopsy or autopsy<br>b. MRI or CT consistent with cirrhosis<br>c. A positive result on ultrasound imaging consistent with cirrhosis<br><br>2. Clinical evidence of decompensation, as documented by one of the following, and without an alternative explanation:<br>a. Ascites<br>b. Hepatic encephalopathy<br>c. Bleeding from gastric or esophageal varices<br>d. Spontaneous bacterial peritonitis	**Probable: 1**<br><br>1. Clinical evidence of decompensation, as documented by one of the following, and without an alternative explanation:<br>a. Ascites<br>b. Hepatic encephalopathy<br>c. Bleeding from gastric or esophageal varices<br>d. Spontaneous bacterial peritonitis
Hypertension	**Confirmed:** 1 or 2<br>1. An average of three blood pressure (BP) readings of systolic of 140 mm Hg or above and/or diastolic of 90 mm Hg or above on the same day and persist 5-7 days after the initial reading.<br>2. An isolated reading of 140mg systolic or 90mg diastolic and presence of the following end-organ disease:<br>a. Cardiac (i.e. left ventricular hypertrophy meeting the ECG criteria [**Appendix 2**] on evidence on cardiac echocardiogram)<br>b. Renal (i.e. microalbuminuria [urinary albumin excretion of 30-300mg/dl], elevated creatinine, reduced estimated GFR (60-90ml/min)<br>c. Retinal(i.e. hypertensive retinal changes)<br>d. Vascular disease (i.e. stroke [persisting on day 7], peripheral vascular disease, myocardial infarction, coronary artery disease requiring drug treatment, congestive cardiac failure)	**Probable: 1**<br><br>1. An average of three BP readings of systolic of 140 mm Hg or above and/or diastolic of 90 mm Hg or above on the same day.
Hyperlipidemia<br><br>NOT FOR ERC<br>TO BE DETERMINED<br>RETROSPECTIVELY	**Confirmed: 1 or 2**<br>1. Fasting total cholesterol >200mg/dl (>5.2 mmol/L) or LDL cholesterol >130mg/dl (>3.4mmol/l) or Triglycerides >150 mg/dl (1.7 mmol/L)<br>2. Non-fasting total cholesterol >240mg/dl (>6.2 mmol/L) or LDL cholesterol >160mg/dl (>4.1 mmol/L) or Triglycerides >200 mg/dl (2.3mmol/L)	**None**
HIV wasting syndrome	None	**Probable:1 + 2 + 3**<br>1. unexplained, involuntary weight loss >10% from baseline,<br>2. persistent diarrhea with > 2 liquid stools/d for > 1 month or weakness for > 1 month or fever for > 1 month,<br>3. tests for alternate causes of weight loss, such as cancer, TB, MAC, cryptosporidiosis or other specific causes of weight loss, if obtained, should be negative

For peer review only - http://bmjopen.bmj.com/site/about/guidelines.xhtml

Appendix

1. Clinical syndrome of Myocardial infarction (a+ b +d) or (c+d)
    a. Chest pain (with associated clamminess, pallor)
    b. Radiation to the upper extremity and jaw
    c. Epigastric discomfort with exertion or at rest
    d. Severe discomfort lasting for more than 20 minutes

2. ECG criteria for LVH

Any two of the following 3 criteria's should be met:

Sokolow Lyon Criteria<br><br>• S in V_1 or V_2 + R in V_5 or V_6 (whichever is larger) ≥ 35 mm (≥ 7 large squares)<br>• R in aVL ≥ 11 mm<br><br>**Meets all of Sokolow Lyon criteria to be diagnostic**	
Cornell voltage criteria<br><br>ECG diagnosis of LVH involve measurement of the sum of the R wave in lead aVL and the S wave in lead V_3. The Cornell criteria for LVH are:<br><br>• S in V_3 + R in aVL > 28 mm (men)<br>• S in V_3 + R in aVL > 20 mm (women)<br><br>**Meets all of Cornell voltage criteria to be diagnostic**	
Romhilt-Estes point score system ECG Criteria	Points
Voltage Criteria (any of):<br>1. R or S in limb leads ≥20 mm<br>2. S in V_1 or V_2 ≥30 mm<br>3. R in V_5 or V_6 ≥30 mm	3
--<br>ST-T Abnormalities:	
1. ST-T vector opposite to QRS without digitalis	3
2. ST-T vector opposite to QRS with digitalis	1
3. Negative terminal P mode in V_1 1 mm in depth and 0.04 sec in duration (indicates left atrial enlargement)	3
4. Left axis deviation (QRS of -30° or more)	2
5. QRS duration ≥0.09 sec	1
6. Delayed R wave peak time (intrinsicoid deflection) in V_5 or V_6 (>0.05 sec)	1
**Romhilt-Estes point score >4 is diagnostic**	

3. Clinical syndrome of Peripheral vascular disease (a+ (b or c or d)
    a. Painful cramping in the hip, thigh or calf muscles after certain activities, such as walking or climbing stairs (claudication)
    b. femoral bruit
    c. decreased peripheral pulses
    d. change in color or temperature of limb suggesting peripheral arterial disease

For peer review only

For peer review only - http://bmjopen.bmj.com/site/about/guidelines.xhtml

RHICCA – Clinical endpoint diagnostic criteria vs 3.5 Apr 2018

4. <u>Clinical syndrome of stroke; should meet the 3 criteria's</u>;

1. **Sudden onset**	
2. **Focal deficit** (or global disturbance but not seizures)	**Large artery disease (anterior circulation syndrome)**<br>Hemi-paresis + Hemi-sensory loss + higher cortical dysfunction (gaze paresis, language impairment [expression + comprehension], visual field defect, hemi-neglect)<br><br>**Large artery disease (posterior circulation syndrome)**<br>Vertigo, visual field defect, gaze paresis, double vision, swallowing difficultly, crossed signs [contralateral limb weakness and ipsilateral cranial nerves abnormality], ataxic limb and gait, drowsy/loss of consciousness<br><br>**Small vessel disease (lacunar syndrome)**<br>Pure hemi-sensory loss<br>Pure hemiparesis<br>Pure sensorimotor<br>Pure ataxic hemiparesis (including dysarthria-clumsy hand syndrome)<br><br>Thunderclap headache*
3. **Lasting > 24 hours** (<24 hours is a TIA)	
*seen in those with a suspicion of subarachnoid or venous stroke. In this case criteria 1 and 3 does not necessarily have to be met	

5. <u>Clinical syndrome of congestive heart failure:</u>

Using the Framingham criteria relies on clinical signs and symptoms; 1 or more major <u>and</u> two or more minor criteria are clinically suggestive of heart failure:

Major criteria

A. Acute pulmonary edema
B. Cardiomegaly
C. Hepatojugular reflex
D. Neck vein distention
E. Paroxysmal nocturnal Dyspnea or Orthopnea
F. Pulmonary crackles
G. Third Heart Sound (S3 Gallup Rhythm)

Minor Criteria

A. Ankle edema
B. Dyspnea on exertion
C. Hepatomegaly
D. Nocturnal cough
E. Pleural Effusion
F. Tachycardia (Heart Rate >120 beats per minute)

For peer review only

For peer review only - http://bmjopen.bmj.com/site/about/guidelines.xhtml

RHICCA – Clinical endpoint diagnostic criteria vs 3.5 Apr 2018

RHICCA
Reactivation of herpesviruses and inflammation as cardiovascular and cerebrovascular risk factors in ART initiators

6. Wells Clinical Prediction Rule for DVT (Adapted from: Wells PS et al. Lancet 1997;350:1796).

One point for each of the following:

- Active cancer (treatment ongoing or within previous 6 months, or palliative)
- Paralysis, paresis, or plaster immobilization of lower extremities
- Recently bedridden for more than 3 days, or major surgery, within 4 weeks
- Localized tenderness along distribution of the deep venous system
- Entire leg swollen
- Calf swelling by more than 3 cm when compared with the asymptomatic leg (measured 10 cm below tibial tuberosity)
- Pitting edema (greater in the symptomatic leg)
- Collateral superficial veins (non-varicose)

7. Clinical symptoms of meningism

Meninigism is the triad of nuchal **rigidity** (neck stiffness), **photophobia** (intolerance of bright light) and **headache**.

8. Clinical symptoms of norcardia

Symptoms vary and depend on the organs involved.

If in the lungs, symptoms may include:

- Chest pain when breathing (may occur suddenly or slowly)
- Coughing up blood
- Fevers
- Night sweats
- Weight loss

If in the brain, symptoms may include:

- Fever
- Headache
- Seizures
- If the skin is affected, symptoms may include:
- Skin breakdown
- Skin breakdown and abnormal passage or draining tract (fistula)
- Ulcers or nodules with infection sometimes spreading along lymph nodes

Some people with nocardia infection have no symptoms.

9. Symptoms of Pneumocystis Pneumonia

- Fever.
- Mild and dry cough or wheezing.
- Shortness of breath, especially with activity.
- Rapid breathing.
- Fatigue.
- Major weight loss.
- Chest pain when you breathe.

10. Clinical syndrome of bacterial pneumonia

- cough with thick yellow, green, or blood-tinged mucus.
- chest pain that worsens when coughing or breathing.
- sudden onset of chills.
- fever of 102°F or above (fever lower than 102°F in older persons)
- headache.
- muscle pain.
- breathlessness or rapid breathing.

For peer review only

For peer review only - http://bmjopen.bmj.com/site/about/guidelines.xhtml

RHICCA – Clinical endpoint diagnostic criteria vs 3.5 Apr 2018

11. Clinical finding of Central Nervous System PML
    - Deficits in motor function, especially **weakness** and **clumsiness**, are common
    - associated altered mental state or behaviour and fever

12. Clinical finding of CNS toxoplasmosis
    - Headaches
    - Seizures
    - Focal neurological deficit of a subacute onset
    - confusion and coma
    - A lung infection, causing cough, fever, and shortness of breath may co-exist.
    -

13. Clinical symptoms suggest of Aspergillosis;

- Fever and chills.
- Cough that brings up blood-streaked sputum (hemoptysis)
- Severe bleeding from the lungs.
- Shortness of breath.
- Chest or joint pain.
- Headaches or eye symptoms.
- Nosebleed
- Facial swelling on one side

Table 1: Abbreviated NARS (Neuropsychiatric AIDS Rating Scale) Grading for HIV Encephalopathy
Adapted from: Price RW, Brew BJ. The AIDS dementia complex. J Infect Dis 1988; 158 (5): 1079-83, and Hughes CP, Berg L, Danziger WL. A new clinical scale for the staging of dementia. Brit J Psych 1982; 140: 566-92.

NARS stage	Cognitive-Behavioral Domains					
	Orientation	Memory	Motor	Behavior	Problem solving	Activities of daily living
0.5	fully oriented	complains of memory problems	fully ambulatory slightly slowed movements	normal	has slight mental slowing	slight impairment in business dealings
1	fully oriented, may have brief periods of “spaciness"	mild memory problems	balance, co-ordination and handwriting difficulties	more irritable, labile or apathetic, withdrawn	difficulty planning and completing work	can do simple daily tasks, may need prompting
2	some disorientation	memory moderately impaired, new learning impaired	ambulatory but may require walking aid	some impulsivity or agitated behavior	severe impairment, poor social judgement, gets lost easily	needs assistance with ADLs
3	frequent disorientation	severe memory loss, only fragments of memory remain	ambulatory with assistance	may have organic psychosis	judgement very poor	cannot live independently
4	confused and disoriented	virtually no memory	bedridden	mute and unresponsive	no problem solving ability	nearly vegetative

For peer review only - http://bmjopen.bmj.com/site/about/guidelines.xhtml

RHICCA – Clinical endpoint diagnostic criteria vs 3.5 Apr 2018

For peer review only

For peer review only - http://bmjopen.bmj.com/site/about/guidelines.xhtml

RHICCA – Clinical endpoint diagnostic criteria vs 3.5 Apr 2018

RHICCA
Reactivation of herpesviruses and inflammation as cardiovascular and cerebrovascular risk factors in ART initiators

Table 2: Diagnostic criteria for classification of definite, probable, possible, and not tuberculosis meningitis (Marais S, et al. Lancet Infect Dis 2010)

	Diagnostic score
**Clinical criteria**	(Maximum category score=6)
Symptom duration of more than 5 days	4
Systemic symptoms suggestive of tuberculosis (one or more of the following): weight loss (or poor weight gain in children), night sweats, or persistent cough for more than 2 weeks	2
History of recent (within past year) close contact with an individual with pulmonary tuberculosis or a positive TST or IGRA (only in children <10 years of age)	2
Focal neurological deficit (excluding cranial nerve palsies)	1
Cranial nerve palsy	1
Altered consciousness	1
**CSF criteria**	(Maximum category score=4)
Clear appearance	1
Cells: 10–500 per µl	1
Lymphocytic predominance (>50%)	1
Protein concentration greater than 1 g/L	1
CSF to plasma glucose ratio of less than 50% or an absolute CSF glucose concentration less than 2.2mmol/L	1
**Cerebral imaging criteria**	(Maximum category score=6)
Hydrocephalus	1
Basal meningeal enhancement	2
Tuberculoma	2
Infarct	1
Pre-contrast basal hyperdensity	2
Evidence of tuberculosis elsewhere	(Maximum category score=4)
**Chest radiograph** suggestive of active tuberculosis: signs of tuberculosis=2; miliary tuberculosis=4	2/4
**CT/ MRI/** ultrasound evidence for tuberculosis outside the CNS 2	
**AFB** identifi ed or *Mycobacterium tuberculosis* cultured from another source—ie, sputum, lymph node, gastric	2
washing, urine, blood culture	4
Positive commercial *M tuberculosis* NAAT from extra-neural specimen	
	4

Exclusion of alternative diagnoses

An alternative diagnosis must be confirmed microbiologically (by stain, culture, or NAAT when appropriate), serologically (eg, syphilis), or histopathologically (eg, lymphoma). The list of alternative diagnoses that should be considered, dependent upon age, immune status, and geographical region, include: pyogenic bacterial meningitis, cryptococcal meningitis, syphilitic meningitis, viral meningo-encephalitis, cerebral malaria, parasitic or eosinophilic meningitis (*Angiostrongylus cantonesis*, *Gnathostoma spinigerum*, toxocariasis, cysticercosis), cerebral toxoplasmosis and bacterial brain abscess (space-occupying Lesion on cerebral imaging)and malignancy (eg, lymphoma)

TST=tuberculin skin test. IGRA=interferon-gamma release assay. NAAT=nucleic acid amplification test. AFB=acid-fast bacilli. The individual points for each criterion (one, two, or four points) were determined by consensus and by considering their quantified diagnostic value as defined in studies.

Key:
Bold text: of the options available likely to be the only tool available in a Malawi setting
Greyed out text: ideal investigation but not available in a Malawi setting

For peer review only

For peer review only - http://bmjopen.bmj.com/site/about/guidelines.xhtml

BMJ Open

Protocol for a longitudinal cohort study to evaluate the reactivation of herpesviruses and inflammation as cardiovascular and cerebrovascular risk factors in antiretroviral therapy initiators, in an African HIV-infected population (RHICCA)

Journal:	*BMJ Open*
Manuscript ID	bmjopen-2018-025576.R2
Article Type:	Protocol
Date Submitted by the Author:	06-Jul-2019
Complete List of Authors:	Peterson, Ingrid; Liverpool School of Tropical Medicine; Malawi Liverpool Wellcome Trust Clinical Research Programme<br>Ntsui, Ntobeko; University of Cape Town<br>Jambo, Kondwani; Liverpool School of Tropical Medicine; Malawi Liverpool Wellcome Trust Clinical Research Programme<br>Kelly, Christine; Malawi Liverpool Wellcome Trust Clinical Research Programme; University College Dublin<br>Huwa, Jacqueline; Malawi Liverpool Wellcome Trust Clinical Research Programme<br>Afran, Louise; Malawi Liverpool Wellcome Trust Clinical Research Programme<br>Tatuene, Joseph; University of liverpool, Institute of Infection and Global Health; Malawi-Liverpool-Wellcome Trust Clinical Research Programme,<br>Pett, Sarah; University College London, Institute of Infection and Global Health; University of New South Wales, Kirby Institute<br>Henrion, Marc; Malawi Liverpool Wellcome Trust Clinical Research Programme; Liverpool School of Tropical Medicine<br>Van Oosterhout, Joep; University of Malawi College of Medicine; Dignitas International<br>Heyderman, Robert; University College London, Division of Infection and Immunity; University of Malawi College of Medicine, Malawi Liverpool Wellcome Trust Clinical Research Programme<br>Mwandumba, Henry; Liverpool School of Tropical Medicine; Malawi Liverpool Wellcome Trust Clinical Research Programme<br>Benjamin, Laura; University of Liverpool Institute of Infection and Global Health, ; University College London Institute of Neurology,
**Primary Subject Heading**:	Cardiovascular medicine
Secondary Subject Heading:	HIV/AIDS, Immunology (including allergy), Infectious diseases, Global health
Keywords:	Ischaemic heart disease < CARDIOLOGY, EPIDEMIOLOGY, HIV & AIDS < INFECTIOUS DISEASES, Stroke medicine < INTERNAL MEDICINE, Cardiovascular

For peer review only - http://bmjopen.bmj.com/site/about/guidelines.xhtml

BMJ OPEN

Title: Protocol for a longitudinal cohort study to evaluate the reactivation of herpesviruses and inflammation as cardiovascular and cerebrovascular risk factors in antiretroviral therapy initiators, in an African HIV-infected population (RHICCA)

Authors:

Ingrid Peterson[1,2], Ntobeko Ntsui[3], Kondwani C Jambo[1,2], Christine Kelly[1,4], Jacqueline Huwa[1], Louise Afran[1], Joseph Kamtchum-Tatuene[5], Sarah Pett[6,7,8], Marc Yves Romain Henrion[1,2], Joep Van Oosterhout[9,10], Robert Heyderman[11], Henry C Mwandumba[1,2], Laura A Benjamin[4,12]** on behalf of the Investigators of the RHICCA study*

Affiliations:

1. Malawi-Liverpool-Wellcome Trust Clinical Research Programme, University of Malawi College of Medicine
2. Department of Clinical Sciences, Liverpool School of Tropical Medicine
3. Department of Medicine, University of Cape Town
4. HIV Molecular Research Group, University College Dublin
5. Institute of Infection and Global Health, University of Liverpool
6. Institute of Global Health, University College London
7. MRC CTU at UCL, Institute of Medicine, Clinical Trials and Methodology, University College London
8. Kirby Institute, University of New South Wales, Australia
9. Dignitas International, PO Box 071, Zomba, Malawi
10. College of Medicine, University of Malawi
11. Department of Infection and Immunity, University College London
12. Department of Brain Repair and Rehabilitation, Institute of Neurology, UCL

For peer review only - http://bmjopen.bmj.com/site/about/guidelines.xhtml

BMJ OPEN

*The Investigators of the RHICCA study

Brian Angus - Oxford Centre for Clinical Tropical Medicine, University of Oxford

Myles Connor - University of Edinburgh

Reena Dwivedi - Greater Manchester Neurosciences Centre, Salford Royal Foundation Trust

Lewis Haddow - Institute for Global Health, University College London

Terttu Heikinheimo-Connell - Hyvinkää Hospital, Department of Neurology, University of Helsinki

Elizabeth Joekes - Liverpool School of Tropical Medicine

Vanessa Kandoole - Department of Medicine, University of Malawi College of Medicine, Blantyre

Moffat Nyrienda - MRC Research Unit, Uganda

Kennedy Malisita- Department of Medicine, Queen Elizabeth Central Hospital

Jane Mallewa- Department of Medicine, University of Malawi College of Medicine, Blantyre

Elsayed Z. Soliman - School of Medicine, Wake Forest School of Medicine

Tom Solomon - Institute of Infection and Global Health, University of Liverpool

**Corresponding author

Laura Benjamin

Institute of Infection and Global Health,

Ronald Ross building,

The University of Liverpool,

L69 7BE, Liverpool,

United Kingdom

For peer review only - http://bmjopen.bmj.com/site/about/guidelines.xhtml

BMJ OPEN

l.benjamin@liverpool.ac.uk

Key words:

Cardiovascular, cerebrovascular, HIV, herpesvirus, Immune dysregulation,

Journal Guidance:

Abstract word count: 300/300

Article word count: 4095 /4000

Figure/Table: 5/5

For peer review only

For peer review only - http://bmjopen.bmj.com/site/about/guidelines.xhtml

BMJ OPEN

ABSTRACT

Introduction: In Sub-Saharan Africa, rising rates of cerebrovascular and cardiovascular disease (CBD/CVD) are intersecting with an aging HIV-infected population. The widespread use of antiretroviral therapy (ART) may confer an additive risk and may not completely suppress the risk associated with HIV infection. High-quality prospective studies are needed to determine if HIV-infected patients in Africa are at increased risk of CBD/CVD and to identify factors associated with this risk. This study will test the hypothesis that immune activation and dysfunction, driven by HIV and reactivation of latent herpesvirus infections lead to increased CBD/CVD risk in Malawian adults aged ≥ 35 years.

Methods and Analysis: We will conduct a single-centre 36-month prospective cohort study in 800 HIV-infected patients initiating antiretroviral therapy (ART) and 190 HIV-uninfected controls in Blantyre, Malawi. Patients and controls will be recruited from government ART clinics and the community, respectively and will be frequency-matched by 5-year age band and sex. At baseline and follow-up visits, we will measure carotid intima thickness (CIMT), pulse wave velocity (PWV) as surrogate markers of vasculopathy, and thus CBD/CVD risk. Our primary exposures of interest will be prospectively measured; these include cytomegalovirus and varicella zoster reactivation, changes in HIV plasma viral load, and markers of systemic inflammation and endothelial function. Multivariable regression models will be developed to assess the study's primary hypothesis. The occurrence of clinical CBD/CVD will be assessed as secondary study endpoints. ISRCTN registry https://doi.org/10.1186/ISRCTN42862937.

Ethics and dissemination: This was approved by the University of Malawi College of Medicine and the Liverpool School of Tropical Medicine research ethics committees. Our goal is to gain insight into the pathogenesis of cardiovascular and cerebrovascular disease among HIV cohorts on ART, in sub-Saharan Africa, and provide data to inform future interventional clinical trials. This study started in May 2017 and will continue until August 2020.

For peer review only

For peer review only - http://bmjopen.bmj.com/site/about/guidelines.xhtml

STRENGTHS AND LIMITATIONS

- This is one of the first large-scale studies in Sub-Saharan Africa to explore the relationship between HIV infection, latent herpesviruses, inflammation and cardiovascular and cerebrovascular diseases, immediately after starting antiretroviral therapy (ART).
- Clinical events and death will be comprehensively reviewed through an end-point review committee, using strict diagnostic criteria for events based on those used in the INSIGHT network, or validated verbal autopsy for death with limited data.
- Because of the recent roll-out of ART in asymptomatic patients, there will be an absence of ART-naïve population, limiting our ability to explore the impact of ART.
- Approximately one-third of strokes will be asymptomatic. We anticipate not capturing some of these. However, multiple cerebral infarcts without a focal neurological deficit will manifest as cognitive impairment, which we will screen for, and corroborate with MRI imaging in a small number of symptomatic cases.
- Two-thirds of myocardial infarction will be silent and could potentially be missed. In a nested group, we will use a digital electrocardiogram to evaluate this further.

For peer review only - http://bmjopen.bmj.com/site/about/guidelines.xhtml

INTRODUCTION

The growing epidemic of cerebrovascular disease (CBD e.g. Stroke) and cardiovascular disease (CVD e.g. myocardial infarction) now intersects with the HIV epidemic[1]. Countries like Malawi, have an adult HIV prevalence of approximately 10%[2]. There is an increased life expectancy among people living with HIV, largely because of the successful scale-up of ART[3]. In Europe and the US, HIV is associated with a 50% increased risk of CVD compared to HIV-uninfected populations[4], attributable to long-term antiretroviral therapy (ART) use and HIV *per se*[4 5]. HIV infection is also associated with a 1.8 fold increased risk of all-cause heart failure in US veterans[6]. Our recent case-control study of stroke in Malawian adults is one of several examples that demonstrates a high risk of HIV infection associated with stroke and heart disease, pointing to a considerable and unappreciated CBD/CVD risk among HIV patients, in this setting[7-10].

There are reports of geographical differences in the distribution of CVD risk factors, supporting the argument that evidence derived from high-income countries cannot be applied to Sub-Saharan (SSA)[11]. Addressing this knowledge gap is essential to the development of clinical drug trials for primary prevention of CBD/CVD among individuals living with HIV. Vasculopathy due to accelerated atherosclerosis, arterial stiffening and vasculitis are the major mechanisms believed to underlie the CBD/CVD burden[12 13]. It is hypothesized that despite viral suppression, low-grade HIV virus replication and the associated host systemic inflammation are important drivers of this vasculopathy (Figure 1). In patients receiving ART, HIV antigenemia, partly resulting from HIV persistence in sanctuary sites, incomplete virologic suppression and virologic resurgence, drives the chronic immune activation observed in about 20% of ART patients in SSA[14]. This immune state is characterized by ongoing activation and senescence of cell-mediated immunity[15 16], increased monocyte/macrophage activation, stimulation of the interleukin-6 (IL-6) pathway and production of acute phase proteins[17-19]. Activation of the IL-6 pathway is established with atherosclerosis[20 21], and may also contribute to non-atherosclerotic vasculopathy. Inflammation alone confers a 2-fold increased risk of clinical CBD/CVD events[22]. The current push to introduce more effective ART regimens, and to start treatment soon after HIV diagnosis is made, may reduce inflammation and in turn, CBD/CVD risk[23]. However, there is

For peer review only - http://bmjopen.bmj.com/site/about/guidelines.xhtml

growing evidence of chronic inflammation in HIV despite achieving the goal of therapy, which is long-term suppression (<50 copies/mL) of plasma viral load, suggesting adjunctive therapy may be required.[24-26]

In addition to HIV, there is compelling evidence that reactivation of latent herpesviruses may be an important cause of vasculopathy. In HIV-uninfected elderly populations from high-income settings, latent cytomegalovirus (CMV) infection drives dysregulation of cell-mediated immunity[15 27-29], not dissimilar to what's described in HIV-associated immune activation[29]. CMV and other viral proteins have been found in atherosclerotic plaques[20]. Varicella-zoster virus (VZV) can directly infect the vascular endothelium to cause vasculitis and subsequent stroke and was found to be the commonest opportunistic infection (prevalence 15%) in a study of HIV-infected stroke patients in Malawi [12]. The seroprevalence of herpesviruses is high in SSA[30], particularly in HIV-infected populations[16].

The involvement of herpesviruses in the mechanistic pathway for CBD/CVD is compelling and may offer additional therapeutic avenues, especially for CMV and VZV. However, our understanding is incomplete, and its population impact is yet to be defined. It is important to determine if, in addition to ART, there is a role for other pharmacological interventions targeting latent viral infections or downstream inflammatory pathways to reduce vasculopathy in HIV-infected patients on ART. Previous work from North America supports the potential of treating reactivated herpesviruses[31]. Furthermore, there are opportunities for intervention using the recently licensed Letermovir; a treatment for CMV. By focusing on HIV and Herpes viral antigenemia and immune dysregulation as mechanisms of vasculopathy, this study will identify subgroups of HIV-infected patients on ART at high risk of CBD/CVD, the timing of CBD/CVD risk in such patients, as well as potential targets for intervention.

For peer review only - http://bmjopen.bmj.com/site/about/guidelines.xhtml

BMJ OPEN

STUDY OBJECTIVES

This study will test the hypothesis that immune activation and dysfunction, driven by HIV and reactivation of latent CMV/VZV herpesvirus infections lead to increased CBD/CVD risk in adults aged $\geq$ 35 years in SSA. We will address this through the following objectives;

1) To determine if progression of the surrogate marker of CBD/CVD or occurrence of new-onset vasculopathy is higher in adults aged $\geq$ 35 years with HIV infection on ART compared to those without HIV.
2) To determine if progression of surrogate markers of CBD/CVD or occurrence of new-onset vasculopathy is higher in adults aged $\geq$ 35 years with HIV/herpes viral antigenemia or chronic immune activation compared to those without HIV/herpes viral antigenemia or chronic immune activation. Specifically, we will determine if progression of surrogate markers or new-onset vasculopathy is higher:
    a. in ART patients with reactivated latent herpes viral infection, compared to those without reactivated latent herpes viral infection.
    b. in ART patients with the highest 25% of markers for immune activation, inflammation or endothelial activation compared to the bottom 25%
    c. in ART patients with incomplete virologic suppression or virologic resurgence of HIV, compared to those with suppressed HIV plasma viral load.

The secondary study objectives are to determine if viral antigenemia or chronic immune activation increase occurrence of the following clinical events: 1) stroke, 2) myocardial infarction (MI), 3) angina (excluding MI), 4) peripheral vascular disease (PVD), 5) all-cause death/vascular-related death and 6) immune reconstitution inflammatory vasculopathy.

METHODS AND ANALYSIS

Study design

To address objective 1, we will conduct a single-center 36-month prospective cohort study in 800 HIV-infected patients initiating ART and 190 HIV-uninfected adults aged $\geq$ 35 years. HIV-infected and HIV-uninfected participants will be frequency matched by 5-year age band and sex. On a 6-monthly basis, we will measure markers of viral infection, inflammation and endothelial function along with surrogate markers for CBD/CVD (Table 1).

For peer review only - http://bmjopen.bmj.com/site/about/guidelines.xhtml

Study Setting

This study will recruit consecutive ART patients from the ART clinic of Queen Elizabeth Central Hospital (QECH), and ART clinics in several Blantyre City Community Health Centres (CHCs). These clinics collectively initiate over 100 HIV-infected patients aged ≥ 35 years onto ART each month. HIV-uninfected adults will be selected from pre-ART counseling sessions, and from randomly selected households in the community by two-stage random sampling (of households and individuals within households) from a previously enumerated sampling frame in the CHC catchment areas[32]. All study procedures will be conducted at QECH, which is located adjacent to the Malawi-Liverpool-Wellcome Trust Clinical Research Programme (MLW). QECH also hosts a 0.35T MRI imaging facility, which will contribute to characterizing our secondary endpoints.

Study Participants

Study inclusion criteria will be: a) age ≥ 35 years and b) resident in Blantyre. HIV-infected patients must further be: c) ART-naïve or initiated ART <10 days prior to enrolment and d) initiating standard first-line ART (in Malawi this is: Tenofovir [TDF]/Lamivudine [3TC]/Efavirenz [EFV]). Adult controls must further be: e) HIV-uninfected. Study exclusion criteria are: f) clinical history of CBD/CVD, g) pregnancy, h) critical illness or symptomatic anemia at baseline and i) enrollment in an intervention study. At the analysis stage abnormal PWV at baseline (as defined in Table 2) will be excluded for new-onset vasculopathy analysis but not for progression of vasculopathy. The same approach will be applied for baseline CIMT measurements. If the study participant becomes pregnant after recruitment, they will be withdrawn.

Justification of study inclusion and exclusion criteria is as follows; in many populations, CBD/CVD risk rises sharply from 35-years of age[33], thus individuals aged 35 and older will be eligible (recruitment of participants aged 35 -39 will be limited to 15% of the study sample to avoid overrepresentation). Restricting recruitment by age will enable this study to have greater statistical power. For clarity of etiologic inference, the study will assess the risk of new-onset vasculopathy not associated with pregnancy and thus exclude patients who are pregnant or with a history of CBD/CVD. To eliminate confounding by ART regimen, patients

For peer review only - http://bmjopen.bmj.com/site/about/guidelines.xhtml

must initiate on standard first-line ART (> 90% of ART patients in Blantyre do this). Critically ill patients are excluded primarily for ethical reasons.

Laboratory methods

Surface immunophenotyping of peripheral blood mononuclear cells

Immunophenotyping will be used to characterize peripheral blood mononuclear cells (PBMC) isolated from blood samples of HIV-uninfected and HIV-infected ART initiators. PBMCs will be harvested by density centrifugation using lymphoprep (Axis Shield, UK). PBMCs ($2x10^6$) will be stained with anti-CD45 PerCP CY5.5, anti-CD3 AF700, anti-CD4 BV421, anti-CD8 PE Dazzle, anti-CD38 BV605, anti-HLA-DR APC CY7, anti-CD57 APC, anti-PD1 PE CY7, anti-CTLA4 PE, and anti-CD223 FITC (all from eBiosciences, UK) to determine the expression of these markers on the surface of T-cells. In addition, ($2x10^6$) PBMCs stained with anti-CD16 BV421, anti-CD14 PE, anti-HLA-DR PerCP CY5.5, anti-CD45 AF700, anti-CCR2 BV605, anti-CD11b APC, anti-CX3CR1 PE Dazzle and anti-CD38 FITC (all from eBiosciences, UK) will be used for monocytes. Dead cells, $CD3^+$ T-cells, and $CD56^+$ NK cells will be excluded using: LIVE/DEAD™ Fixable Aqua Dead Cell Stain (Thermofisher, UK), anti-CD3 BV503 and anti-CD56 BV503 (eBiosciences, UK), respectively. Stained cells will be acquired on a BD LSR Fortessa flow cytometer (Becton Dickinson, USA) and data will be analyzed using FlowJo software version 10.0 (Tree Star, San Carlos, CA). For each stained sample analyzed, the median fluorescence intensity (MFI) for each parameter will be normalized to its respective unstained control.

Measurement of soluble markers of immune activation using multiplex bead array

A custom-made multiplex assay will be used to assess soluble markers of monocyte activation (CD163), systemic inflammation (Interleukin-6) and endothelial activation (Intracellular adhesion molecule 1) in plasma, isolated from blood samples of HIV-uninfected and HIV-infected ART initiators. Following isolation, plasma will be aliquoted and stored at -80^oC until further use.

For peer review only - http://bmjopen.bmj.com/site/about/guidelines.xhtml

Assessment of exposure to human cytomegalovirus and varicella zoster virus by ELISA

Quantitative VIDAS CMV IgG and IgM (BioMerieux, USA) and VZV glycoprotein IgG Low-Level Enzyme Immunoassay Kit [VaccZyme™EIA], will be used to determine exposure to these viruses using a commercial enzyme-linked immunosorbent assay (ELISA) platform. These kits will detect VZV antigen to a sensitivity and specificity of 97.8% and 96.8% respectively and for CMV, 97.2% and 100% for IgG and 100% and 97.4% for IgM respectively[34 35]. Plasma samples from HIV-uninfected and HIV-infected ART initiators stored at -80°C following collection will be used for these assessments

HIV

HIV infection will be diagnosed using two rapid tests in parallel, EIA rapid tests (Determine HIV-1/2 [Abbott Laboratories, USA] and Uni-Gold HIV [Trinity Biotech PLC, Ireland]), will be used as a tiebreak). HIV-1 RNA levels in plasma will be measured using the Abbott Real-Time HIV-1 assay with a lower limit of detection of 150 copies/mL (Abbott Molecular, Germany), according to the manufacturer's instructions. CD4+ T -cell count measurements will be performed using BD FACS Count machine (Partec platform).

Procedures

Carotid-femoral pulse wave velocity (PWV)[36] and carotid intima-media thickness (CIMT)[37] measurement will be performed in accordance with expert consensus guidelines, using a standardized study protocol on the Vicorder system (SMART Medical, UK) and Philips CX50 machine (Philips healthcare, UK) respectively. CIMT measurements will be performed by three trained operators. The intra-class correlation coefficient will be used to assess the performance of the operators against that of a certified neurosonologist prior to study commencement.

For peer review only - http://bmjopen.bmj.com/site/about/guidelines.xhtml

Outcomes

Primary outcomes

Primary outcomes are the progression of surrogate markers of CBD/CVD, namely PWV and CIMT as well as the occurrence of new-onset vasculopathy defined by threshold values outlined in Table 2.

Secondary outcomes

Secondary outcomes are the following clinical events: 1) stroke, 2) myocardial infarction (MI), 3) unstable angina, 4) peripheral vascular disease (PVD), 5) all-cause death/ vascular death and 6) immune reconstitution inflammatory syndrome (IRIS) vasculopathy (Table 2). Changes in PWV or endothelial activation at 6 months post ART initiation will be interpreted as a subclinical vascular IRIS event. These outcomes will be assessed through active surveillance in QECH inpatient wards for admissions of study participants. To improve capture of clinical outcomes, we will conduct brief telephone interviews with study participants about CBD/CVD symptoms and hospitalizations between study visits and facilitate unsolicited participant self-report. Clinical events and deaths in study participants will be reviewed by an independent endpoint review committee (ERC), comprising of clinicians experienced in Endpoint review. Each event will be reviewed and adjudicated by the ERC Chair and 2 ERC reviewers, using a standard set of diagnostic criteria (Table 2 and Supplement – S1). The format of reporting will be based on modifications of the INSIGHT network clinical diagnostic criteria. Deaths will be reviewed by the ERC using the CoDe approach[23]. For death with limited clinical data, a validated verbal autopsy will be performed to ascertain the cause[38].

Exposures

The exposure for Primary Objective 1 will be HIV status. Yearly HIV rapid tests in HIV-uninfected adults will be performed to exclude those with new HIV infections (Figure 2).

Potential confounding and mediating factors will be recorded in study participants. This will include demographic factors, lifestyle and behavioral factors (e.g. cigarette smoking and alcohol consumption), chronic co-morbidities (i.e. hypertension, diabetes), cardiometabolic,

For peer review only - http://bmjopen.bmj.com/site/about/guidelines.xhtml

renal and hematological factors (i.e. full blood count, creatinine in urine and serum, body-mass-index, waist-to-hip ratio, random glucose, HbA1c, and lipid profile). Blood pressure will be measured at all study visits. Although vascular immune reconstitution inflammatory syndrome (IRIS) (Table 2) will be considered as a primary endpoint, non-vascular IRIS will be defined as a risk factor. Where feasible, we will conduct PCR tests for common causes of IRIS in blood or cerebrospinal fluid (CSF) samples. Adherence to ART and change of ART regimen will be assessed at all study visits through extraction of data from 'ART master cards'; this is a government-supported monitoring tool used by all patients on ART, in Malawi.

For Objective 2a-2c, markers of herpes and HIV viral antigenemia and immune inflammation will be measured according to the outline in Table 1. For primary objective 2a, reactivated latent herpes viral infections will be assessed by quantification of VZV, and CMV antibodies. We will estimate the risk of atherosclerosis and arterial stiffening associated with current herpesviruses reactivation at baseline, and sustained reactivation (i.e. those that continue to have a high titer from measurement at baseline to 6 months after ART initiation). Hyperactivation of B cells may result in an expansion of polyclonal antibodies and thus an overestimation of virus-specific antibody titers. To address this issue and make appropriate adjustments for hypergammaglobulinemia we will 1) measure more than one herpesviruses and 2) measure total IgG.

For primary objective 2b, markers of immune activation, inflammation, and endothelial activation will be measured (Figures 1 & Table 1). Quantitative cell surface immunophenotyping will be performed for CD4+ and CD8+ T-cell activation (e.g. HLA-DR) and senescence (e.g. CD57) in a subset of participants. In all study participants, at baseline, 6, 12, months, we will measure soluble markers associated with systemic inflammation and endothelial activation.

For primary objective 2c, incomplete viral response and viral rebound of HIV will be measured by quantitative PCR in patients on ART.[39] HIV viral load will be measured in patients on ART at 0, 6 and 12 months.

For peer review only - http://bmjopen.bmj.com/site/about/guidelines.xhtml

BMJ OPEN

Data Collection Between May 2017 and August 2020

The two-stage screening will be conducted to find and recruit potential study participants. A trained field worker will first screen to assess eligibility for criteria (a)-(c) in pre-ART counseling sessions, and in individuals from randomly selected households in the community. Eligible participants will then be referred to QECH to complete screening for criteria (d)-(i) and if eligible, consented to participate in the study. At study visits, a tablet-based, standardized Open Data Kit (ODK) case report form (CRF) will be administered in one-on-one interviews by a study nurse to capture demographic and clinical data. Study data will be collected as outlined in Table 1. Daily upload of electronic data will occur with oversight from the data manager at MLW. We will collect up to 30ml of whole blood. An ACR dipstick test will be used to test for creatinine, proteinuria, and glucosuria. In a subset of participants, an electrocardiogram supported by a digital platform and echocardiogram will be performed at baseline, 6 and 24 months, as well as in any participant experiencing a clinical event suggestive of a cardiac etiology. To facilitate the retention and clinical referrals of participants, contact will be made every 3 months to assess the occurrence of clinical events. Participants who miss a scheduled study visit will be contacted by phone and/or visited at home to assess their willingness to maintain their participation and to record intervening clinical events. Recording and definitions of other clinical events, including HIV associated events will be evaluated by the ERC chair. SMS messages will be used for appointment reminders. Technical appendix, statistical code, and dataset will be made available from a data repository, after publication of our work.

Sample Size and Statistical Analysis

The required sample size for the study's primary objectives is 800 HIV-infected patients and 190 HIV-uninfected adults using standard, normal distribution approximation sample size formulas for comparing proportions in two groups of unequal size and based on the following assumptions: **a)** 18.4% of HIV positive study participants have abnormal PWV at baseline. We will exclude these participants from analysis. The 18.4% figure is informed by our ongoing studies of vasculopathy in HIV-infected patients, where this is the percentage of participants aged $\geq$35 years that have a PWV (>12 m/s). **b)** 20% of both HIV-infected patients and HIV-uninfected adults will be lost to follow-up, including by death and HIV

For peer review only - http://bmjopen.bmj.com/site/about/guidelines.xhtml

sero-conversion[40 41]. **c)** The minimum relative risk (RR) of interest is 2 for Objective 1 and 1.8 for Objective 2. **d)** The 24-month cumulative risk of clinically significant vasculopathy over study follow-up is 18.4% in the HIV positive group. This is based on the same study data cited in (a). **e)** For objectives 2a)-c)**,** the exposure prevalence for each risk factor is 20%. **f)** Statistical tests will have 80% power based on a 2-sided test with; α=0.05. Testing of hypotheses for the secondary outcome will be exploratory. However, we estimate 26 strokes (4 mimics), an unknown number of MIs and 80 deaths occurring during the study[7 42]. Taken together, c), d), e) mean that, for 80% power, we assume a 24-month cumulative vasculopathy risk of 9.2% in HIV negative participants, 18.4% in all HIV infected participants, 15.9% in HIV infected participants not exposed to the risk factors from objectives 2a)-c), 28.6% in the HIV infected participants exposed to these risk factors.

The reporting of this study will be prepared in accordance with the STROBE guidelines[43]. Summary and descriptive statistics will be tabulated for all primary and secondary outcome variables, as well as for exposure variables and potential confounding or mediating factors. Time plots for all outcome variables will be inspected. Quantitative data analysis will be conducted to assess the primary outcomes.

There will be 3 analysis time points: 1) after recruitment has finished and baseline data is available for all participants (baseline analysis), 2) once every participant has completed 6 months in the study (6-month analysis) and 3) at 36 months, when each participant has completed 24 months in the study (final analysis).

The baseline analysis will largely consist of descriptive statistics on participant characteristics and data recorded at baseline. Simple regression models will also be used to investigate relationships between exposure and outcome variables measured at baseline. Unadjusted analyses will consist of paired t-tests or Wilcoxon signed rank tests (depending whether the data are normally distributed or not) for continuously measured variables and Chi-Squared or Fisher's exact tests (depending on contingency table cell counts) for binary and categorical variables. Adjusted analyses will be conducted using generalised linear models (GLMs).

For peer review only - http://bmjopen.bmj.com/site/about/guidelines.xhtml

The 6-month analysis will be limited in score and serves 2 purposes: 1) characterise new onset vasculopathy in HIV-infected participants that have initiated ART treatment at baseline (vascular IRIS) and 2) define vasculopathy outcomes for the final analysis. The main analysis of the study data happens at the final analysis time point.

For objective 1 we will develop three regression models. Two GLMs will be developed to compare mean progression of arterial damage from baseline in HIV-infected ART patients and HIV-uninfected adults. These models will regress change from baseline in PWV, respectively cIMT, on HIV status. We will develop a third model to estimate the RR and population attributable fraction of new-onset arterial damage in HIV-infected patients compared to HIV-uninfected adults.

For objective 2a, a set of GLMs will be developed to compare mean progression of vasculopathy in HIV-infected ART patients with and without reactivated latent herpes viral infection. These models will regress change from baseline in PWV, respectively cIMT, on two log-transformed variables for antibody titres of CMV and VZV, respectively.

For objective 2b, we will again fit a set GLMs, with change from baseline in PWV as response variable, this time to investigate if, in HIV-infected ART patients, there is an association between progression of vasculopathy and immune activation and inflammation biomarkers (IL-6, ICAM, CD163). Specifically, for each marker, we will regress PWV on marker quantiles. After having built models for each marker, we will then develop comprehensive multiple regression models for PWV and cIMT with multiple independent markers as predictor variables.

For objective 2c, we will proceed as for objective 2a, but comparing HIV-infected ART patients with incomplete virological suppression or virological resurgence of HIV to those with suppressed HIV plasma viral load.

For peer review only - http://bmjopen.bmj.com/site/about/guidelines.xhtml

In addition to these analyses, given the repeated measurements for PWV, immune activation, inflammation markers, we will extent the GLMs for PWV to linear mixed models taking full account of the longitudinal nature of the data. Mixed models will also handle deviations from the visit schedule in a principled fashion and use all available data for drop-outs. In the case a log link function is required for PWV in the GLMs, we will fit marginalised models using GEE instead of the LMMs.

For secondary study objectives, we will use univariate methods to assess the frequency of clinical events within exposure strata. If there are sufficient numbers of clinical events we will develop Poisson or negative binomial regression models (depending on model fit) for each clinical event type to compare exposure-defined participants.

We will also use time-to-event models, specifically Cox proportional hazard models, to investigate associations between all-cause mortality and exposures.

As part of exploratory analyses, we will aim to identify risk groups that are potentially incompletely captured with the measured exposure variables. We will perform unsupervised group-based multi-trajectory modeling of multivariate longitudinal patient trajectories to confirm any associations we have found using more traditional approaches[44].

All efforts will be made to collect complete data on all study participants. However, there will inevitably be missing data due to drop-outs and a variety of other reasons. All primary analyses will be performed using multiple imputation. For sensitivity analyses, we will use all-available-cases (AAC), direct likelihood and fully Bayesian models and, for GEE models, weighted GEE. If the number of missingness patterns is sufficiently small, we will also use pattern mixture models which can be used under the general missing-not-at-random setting but make additional identification assumptions.

For peer review only - http://bmjopen.bmj.com/site/about/guidelines.xhtml

PATIENT PUBLIC INVOLVEMENT

The global burden of HIV associated CBD and CVD has tripled over the last two decades with the greatest impact in sub-Saharan Africa. CBD and CVD are a priority for patients in Malawi as HIV infection is endemic and the population are living for longer. Knowledge of this, informed our research question with the aim of understanding the mechanisms and thus direct targeted novel therapies to reduce this burden. Patients will be involved in the recruitment of the study, but not in the design. Patients and their advisors will be thanked for contributing to the study.

ETHICS AND DISSEMINATION

Written informed consent will be obtained from all study participants, either written or witnessed verbal consent with thumbprint if the participant is non-literate. Study data will be maintained in an encrypted and password protected database to which only study staff will have access. Study participants who develop a clinical event will be managed, using the hospital guidelines, by our study clinician alongside the hospital doctor. Clinical data will be anonymized using unique identifying code. Study data will be kept for 10 years and then destroyed with a record, as recommended by good clinical practice guidelines. This protocol was approved by the ethics committees at University of Malawi College of Medicine (Protocol P02/16/1874) and the Liverpool School of Tropical Medicine (Protocol 16-014).

DISCUSSION

African regions continue to bear the brunt of HIV infection, in 2013, an estimated 8.5 million adults were receiving ART[45]. As the landscape evolves, this population will live longer with stable HIV infection but likely remain at an increased risk of CBD/CVD compared to HIV-uninfected individuals of a similar age and sex. This study will be the first to determine the extent to which HIV reactivation of herpesvirus infection and inflammation contribute to CBD/CVD risk in an adult African population starting ART. The results of this work could potentially open avenues for novel anti-inflammatory and anti-viral interventions for the primary prevention of CBD/CVD in HIV populations in Africa.

For peer review only - http://bmjopen.bmj.com/site/about/guidelines.xhtml

ACKNOWLEDGMENTS

The authors would like to thank Agbor Ako and Maria Davy from Research and Development, GlaxoSmithKline and the NCD Africa Open Lab of GlaxoSmithKline review committee for providing valuable advice for this protocol. The authors would like to thank BA, MC, LH, THC, JVO, NT for their contribution to the End Point Review Committee, RD and EJ for radiology training and quality control, EZS for providing an electrocardiogram platform and for his cardiology review, VK for input with the echocardiogram protocol, and TS, JM, KM, MN for their input in the advanced drafts of the manuscript. We also extend our gratitude to the INSIGHT network for sharing their clinical endpoint criteria. LB is supported by an NIHR Clinical Lecturer Fellowship. SP is supported by an MRC (UK) core funding MC_UU_12023/23.

AUTHORS' CONTRIBUTIONS

LB and IP developed the first draft. HM, NN, KJ, CK, LA, JKT, SP, MH, JVO, RH had major input for the revision of the second draft. JH is the project manager for RHICCA with oversight from LB, IP, and HM. MH contributed to the statistical methods. LB, JKT, JVO contributed to the clinical training. SP chaired the End point Review Committee.

FUNDING STATEMENT

Funding for this study was provided by the GlaxoSmithKline Africa Non-Communicable Disease Open Lab Grant (Project Number: 7964)

COMPETING INTERESTS

SLP has academic grants from Sysmex Corporation, Gilead Sciences, and ViiV Healthcare. All other authors have no competing interest.

For peer review only - http://bmjopen.bmj.com/site/about/guidelines.xhtml

BMJ OPEN

For peer review only

For peer review only - http://bmjopen.bmj.com/site/about/guidelines.xhtml

REFERENCES

1. Roth GA, Johnson C, Abajobir A, et al. Global, Regional, and National Burden of Cardiovascular Diseases for 10 Causes, 1990 to 2015. *J Am Coll Cardiol* 2017;70(1):1-25. doi: 10.1016/j.jacc.2017.04.052
2. Organization WH. Global Update on HIV Treatment 2013: Results, Impact and Opportunities. WHO Report. Kuala Lumpure, Malaysia, 2013.
3. Macro NSONaI. Malawi Demographic and Health Survey 2010. Zomba,Malawi, and Calverton, Maryland, USA: NSO and ICF Macro, 2010.
4. Freiberg MS, Chang CC, Kuller LH, et al. HIV infection and the risk of acute myocardial infarction. *JAMA Intern Med* 2013;173(8):614-22. doi: 10.1001/jamainternmed.2013.3728

1659742 [pii] [published Online First: 2013/03/06]
5. Currier JS, Lundgren JD, Carr A, et al. Epidemiological evidence for cardiovascular disease in HIV-infected patients and relationship to highly active antiretroviral therapy. *Circulation* 2008;118(2):e29-35. doi: 10.1161/CIRCULATIONAHA.107.189624 [published Online First: 2008/06/21]
6. Butt AA, Chang CC, Kuller L, et al. Risk of heart failure with human immunodeficiency virus in the absence of prior diagnosis of coronary heart disease. *Archives of internal medicine* 2011;171(8):737-43. doi: 10.1001/archinternmed.2011.151 [published Online First: 2011/04/27]
7. Benjamin LA, Corbett EL, Connor MD, et al. HIV, antiretroviral treatment, hypertension, and stroke in Malawian adults: A case-control study. *Neurology* 2016;86(4):324-33. doi: 10.1212/WNL.0000000000002278
8. Allain TJ, Kinley L, Tsidya B, et al. The spectrum of heart disease in adults in Malawi: A review of the literature with reference to the importance of echocardiography as a diagnostic modality. *Malawi Med J* 2016;28(2):61-65. [published Online First: 2016/11/30]
9. Soliman EZ, Juma H. Cardiac disease patterns in northern Malawi: epidemiologic transition perspective. *J Epidemiol* 2008;18(5):204-8. [published Online First: 2008/08/30]
10. Syed FF, Sani MU. Recent advances in HIV-associated cardiovascular diseases in Africa. *Heart* 2013;99(16):1146-53. doi: 10.1136/heartjnl-2012-303177 [published Online First: 2013/05/18]
11. Soliman EZ, Sharma S, Arasteh K, et al. Baseline cardiovascular risk in the INSIGHT Strategic Timing of AntiRetroviral Treatment (START) trial. *HIV Med* 2015;16 Suppl 1:46-54. doi: 10.1111/hiv.12233 [published Online First: 2015/02/26]
12. Benjamin LA, Allain TJ, Mzinganjira H, et al. The Role of Human Immunodeficiency Virus-Associated Vasculopathy in the Etiology of Stroke. *J Infect Dis* 2017;216(5):545-53. doi: 10.1093/infdis/jix340 [published Online First: 2017/09/22]
13. Benjamin LA, Bryer A, Lucas S, et al. Arterial ischemic stroke in HIV: Defining and classifying etiology for research studies. *Neurol Neuroimmunol Neuroinflamm* 2016;3(4):e254. doi: 10.1212/NXI.0000000000000254
14. Nakanjako D, Kiragga A, Ibrahim F, et al. Sub-optimal CD4 reconstitution despite viral suppression in an urban cohort on antiretroviral therapy (ART) in sub-Saharan Africa: frequency and clinical significance. *AIDS Res Ther* 2008;5:23. doi: 10.1186/1742-6405-5-23 [published Online First: 2008/10/30]
15. Pawelec G, Derhovanessian E. Role of CMV in immune senescence. *Virus Res* 2011;157(2):175-9. doi: 10.1016/j.virusres.2010.09.010 [published Online First: 2010/09/28]
16. Appay V, Sauce D. Immune activation and inflammation in HIV-1 infection: causes and consequences. *J Pathol* 2008;214(2):231-41. doi: 10.1002/path.2276 [published Online First: 2007/12/29]
17. Shaw AC, Joshi S, Greenwood H, et al. Aging of the innate immune system. *Curr Opin Immunol* 2010;22(4):507-13. doi: 10.1016/j.coi.2010.05.003 [published Online First: 2010/07/30]

For peer review only - http://bmjopen.bmj.com/site/about/guidelines.xhtml

18. Hearps AC, Angelovich TA, Jaworowski A, et al. HIV infection and aging of the innate immune system. *Sex Health* 2011;8(4):453-64. doi: 10.1071/SH11028 [published Online First: 2011/12/01]
19. Kovacs EJ, Palmer JL, Fortin CF, et al. Aging and innate immunity in the mouse: impact of intrinsic and extrinsic factors. *Trends Immunol* 2009;30(7):319-24. doi: 10.1016/j.it.2009.03.012 [published Online First: 2009/06/23]
20. Hansson GK. Inflammation, atherosclerosis, and coronary artery disease. *The New England journal of medicine* 2005;352(16):1685-95. doi: 10.1056/NEJMra043430 [published Online First: 2005/04/22]
21. Boekholdt SM, Stroes ES. The interleukin-6 pathway and atherosclerosis. *Lancet* 2012;379(9822):1176-8. doi: 10.1016/S0140-6736(12)60361-4 [published Online First: 2012/03/17]
22. Duprez DA, Neuhaus J, Kuller LH, et al. Inflammation, coagulation and cardiovascular disease in HIV-infected individuals. *PloS one* 2012;7(9):e44454. doi: 10.1371/journal.pone.0044454 [published Online First: 2012/09/13]
23. Lundgren JD, Babiker AG, Gordin F, et al. Initiation of Antiretroviral Therapy in Early Asymptomatic HIV Infection. *N Engl J Med* 2015;373(9):795-807. doi: 10.1056/NEJMoa1506816 [published Online First: 2015/07/21]
24. Eggena MP, Barugahare B, Okello M, et al. T cell activation in HIV-seropositive Ugandans: differential associations with viral load, CD4+ T cell depletion, and coinfection. *The Journal of infectious diseases* 2005;191(5):694-701. doi: 10.1086/427516 [published Online First: 2005/02/03]
25. Mussini CL, P.; Cozzi-Lepri,A.; Lapadula,G.; Marchetti,G.; Nicastri,E.; Cingolani,A.; Lichtner,M.;Antinori,A.; Gori,A.; Monforte, A. CD4/CD8 ratio normalisation and non-AIDS-related events in individuals with HIV who achieve viral load suppression with antiretroviral therapy: an observational cohort study. *Lancet HIV* 2015;2:e98–e106.
26. Sereti I, Krebs SJ, Phanuphak N, et al. Persistent, Albeit Reduced, Chronic Inflammation in Persons Starting Antiretroviral Therapy in Acute HIV Infection. *Clin Infect Dis* 2017;64(2):124-31. doi: 10.1093/cid/ciw683 [published Online First: 2016/10/16]
27. Brunner S, Herndler-Brandstetter D, Weinberger B, et al. Persistent viral infections and immune aging. *Ageing Res Rev* 2011;10(3):362-9. doi: 10.1016/j.arr.2010.08.003 [published Online First: 2010/08/24]
28. Moss P. The emerging role of cytomegalovirus in driving immune senescence: a novel therapeutic opportunity for improving health in the elderly. *Curr Opin Immunol* 2010;22(4):529-34. doi: 10.1016/j.coi.2010.07.001 [published Online First: 2010/08/06]
29. Appay V, Rowland-Jones SL. Premature ageing of the immune system: the cause of AIDS? *Trends Immunol* 2002;23(12):580-5. [published Online First: 2002/12/05]
30. Schaftenaar E, Verjans GM, Getu S, et al. High seroprevalence of human herpesviruses in HIV-infected individuals attending primary healthcare facilities in rural South Africa. *PloS one* 2014;9(6):e99243. doi: 10.1371/journal.pone.0099243 [published Online First: 2014/06/11]
31. Hunt PW, Martin JN, Sinclair E, et al. Valganciclovir reduces T cell activation in HIV-infected individuals with incomplete CD4+ T cell recovery on antiretroviral therapy. *J Infect Dis* 2011;203(10):1474-83. doi: 10.1093/infdis/jir060 [published Online First: 2011/04/20]
32. Corbett EL. Intensified HIV/TB prevention linking home-based HIV testing, including the option of selftesting, with HIV care. ISRCTN02004005. London: ISRCTN, 2012.
33. Roth GA, Huffman MD, Moran AE, et al. Global and regional patterns in cardiovascular mortality from 1990 to 2013. *Circulation* 2015;132(17):1667-78. doi: 10.1161/CIRCULATIONAHA.114.008720
34. Maple PA, Breuer J, Quinlivan M, et al. Comparison of a commercial Varicella Zoster glycoprotein IgG enzyme immunoassay with a reference time resolved fluorescence immunoassay (VZV TRFIA) for measuring VZV IgG in sera from pregnant women, sera sent for confirmatory

For peer review only - http://bmjopen.bmj.com/site/about/guidelines.xhtml

testing and pre and post vOka vaccination sera from healthcare workers. *J Clin Virol* 2012;53(3):201-7. doi: 10.1016/j.jcv.2011.12.010 [published Online First: 2012/01/21]
35. Carlier P, Harika N, Bailly R, et al. Laboratory evaluation of the new Access (R) cytomegalovirus immunoglobulin IgM and IgG assays. *J Clin Virol* 2010;49(3):192-7. doi: 10.1016/j.jcv.2010.07.024 [published Online First: 2010/08/31]
36. Laurent S, Cockcroft J, Van Bortel L, et al. Expert consensus document on arterial stiffness: methodological issues and clinical applications. *Eur Heart J* 2006;27(21):2588-605. doi: 10.1093/eurheartj/ehl254 [published Online First: 2006/09/27]
37. Touboul PJ, Hennerici MG, Meairs S, et al. Mannheim carotid intima-media thickness and plaque consensus (2004-2006-2011). An update on behalf of the advisory board of the 3rd, 4th and 5th watching the risk symposia, at the 13th, 15th and 20th European Stroke Conferences, Mannheim, Germany, 2004, Brussels, Belgium, 2006, and Hamburg, Germany, 2011. *Cerebrovasc Dis* 2012;34(4):290-6. doi: 10.1159/000343145

000343145 [pii] [published Online First: 2012/11/07]
38. Serina P, Riley I, Stewart A, et al. Improving performance of the Tariff Method for assigning causes of death to verbal autopsies. *BMC Med* 2015;13:291. doi: 10.1186/s12916-015-0527-9 [published Online First: 2015/12/09]
39. Organization WH. Consolidated ARV guidelines 2013 [Available from: http://www.who.int/hiv/pub/guidelines/arv2013/art/artmonitoring/en/index4.html accessed 15 Oct 2015.
40. Misiri HE, Edriss A, Aalen OO, et al. Estimation of HIV incidence in Malawi from cross-sectional population-based sero-prevalence data. *Journal of the International AIDS Society* 2012;15(1):14. doi: 10.1186/1758-2652-15-14 [published Online First: 2012/03/16]
41. MacPherson P, Houben RM, Glynn JR, et al. Pre-treatment loss to follow-up in tuberculosis patients in low- and lower-middle-income countries and high-burden countries: a systematic review and meta-analysis. *Bull World Health Organ* 2014;92(2):126-38. doi: 10.2471/BLT.13.124800 [published Online First: 2014/03/14]
42. Walker R, Whiting D, Unwin N, et al. Stroke incidence in rural and urban Tanzania: a prospective, community-based study. *Lancet Neurol* 2010;9(8):786-92. doi: S1474-4422(10)70144-7 [pii]

10.1016/S1474-4422(10)70144-7 [published Online First: 2010/07/09]
43. von Elm E, Altman DG, Egger M, et al. The Strengthening the Reporting of Observational Studies in Epidemiology (STROBE) Statement: guidelines for reporting observational studies. *International journal of surgery* 2014;12(12):1495-9. doi: 10.1016/j.ijsu.2014.07.013 [published Online First: 2014/07/22]
44. Nagin DS, Jones BL, Passos VL, et al. Group-based multi-trajectory modeling. *Stat Methods Med Res* 2018;27(7):2015-23. doi: 10.1177/0962280216673085 [published Online First: 2018/05/31]
45. Organization WH. Global Update on the Health Sector Response to HIV 2014. Geneva: World Health Organization, 2014.

For peer review only - http://bmjopen.bmj.com/site/about/guidelines.xhtml

Figure 1. Hypothetical pathway of the interplay between chronic viruses, immune activation, systemic inflammation, endothelial activation, and vasculopathy.

Figure 2. Outline of study design for a 36-month cohort study

For peer review only

For peer review only - http://bmjopen.bmj.com/site/about/guidelines.xhtml

Table 1. Laboratory tests and clinical procedures in ART patients and HIV-uninfected adults

	Study Time Points						
	Baseline	6 months	12 months	18 months	24 months	30 months	36 months
**Clinical Procedures**							
PWV	X	X	X	X	X	X	X
CIMT	X				X		
ABPI	X	X	X	X	X	X	X
Cardiac Echo *(participant sub-set)*	X				X		
ECG *(participant sub-set)*	X				X		
**Cardiometabolic markers**							
Creatinine	X				X		
Full Blood Count	X	X					
Cholesterol (LDL, HDL, Triglycerides)	X				X		
Serum glucose/HBA1C	X				X		
**HIV Infection and Progression**							
HIV viral load *(HIV patients)*	X	X	X				
CD4 count *(HIV patients)*	X	X	X				
HIV rapid test *(controls)*	X		X		X		X
**Immune dysregulation**							
Soluble markers of systemic inflammation	X	X	X				
Soluble markers of endothelial activation	X	X	X				
CD8 and CD4 T-cell activation and senescence *(participant subset)*	X	X	X		X		X
Monocyte/ Macrophage activation and senescence *(participant subset)*	X	X	X		X		X
Herpesviruses infection							
CMV IgG	X	X					
VZV IgG	X	X					

For peer review only

For peer review only - http://bmjopen.bmj.com/site/about/guidelines.xhtml

Table 2: Case definitions of primary and secondary endpoints for the study

	Type	Definitions
**Primary Endpoint**	Carotid intimal medial thickness (CIMT)	The occurrence of new-onset vasculopathy [CIMT – a measure of atherosclerosis]: CIMT >0.9 mm or >75th percentile of age/sex references values or presence of plaque on the carotid scan<br><br>Progression: total change in CIMT at 24 months from baseline
	Pulse wave velocity (PWV)	Occurrence of new onset vasculopathy [PWV – a measure of arterial stiffness]: PWV >12[m/s]<br><br>Progression: total change in PWV at 24 months from baseline
**Secondary endpoint**	Stroke	**Confirmed (1+2) or 3 or 4 or 5:**<br>1. Acute onset with a clinically compatible course, including unequivocal objective findings of a localizing neurologic deficit<br>2. CT or MRI compatible with a diagnosis of stroke and current neurologic signs and symptoms<br>3. Stroke diagnosed as the cause of death at autopsy<br>4. Compatible clinical history and positive lumbar puncture compatible with subarachnoid hemorrhage<br>5. Death certificate or death note from medical record listing stroke as the cause of death
	Myocardial Infarction [MI]	**Confirmed: One of the following 5 criteria (adapted from 2007 Universal Definition of Myocardial Infarction): [1 + (2 or 3 or 6)] or 4 or 5**<br><br>1. Rise and/or fall of cardiac biomarkers (preferably troponin), with at least one value above the 99th percentile of upper reference limit (URL);<br>2. The occurrence of a compatible clinical syndrome, including symptoms consistent with myocardial ischemia;<br>3. ECG changes indicative of new ischemia (new ST-changes or new left bundle branch block [LBBB]), or development of pathological Q waves on the ECG;<br>4. Sudden unexpected cardiac death involving cardiac arrest before biomarkers are obtained or before a time when biomarkers appear, along with (a) new ST-changes or new LBB, or (b) evidence of fresh thrombus on coronary angiography or at autopsy<br>5. Pathologic findings of acute myocardial infarction (including

For peer review only - http://bmjopen.bmj.com/site/about/guidelines.xhtml

[redacted]		acute MI demonstrated as the cause of death on autopsy)<br>6. Development of a) evolving new Q waves, or b) evolving ST elevation, preferably based on at least 2 ECGs taken during the same hospital admission
	Coronary artery disease requiring drug treatment	**Confirmed (1 or 2) + 3:**<br><br>1. Evidence of myocardial ischemia based on either diagnostic imaging (such as a stress echocardiogram or thallium scan) or diagnostic changes on an electrocardiogram (such as during stress testing or an episode of chest pain)<br>2. Evidence of coronary artery disease based on coronary angiography or other diagnostic imaging<br>3. Use of medication given to treat or prevent angina (e.g., nitrates, beta blockers, calcium channel blockers)
	Peripheral vascular disease [PVD]	**Confirmed (1+2) or (1+3):**<br>1. Compatible clinical signs and symptoms<br>2. Positive results on diagnostic imaging studies (e.g., Doppler ultrasound, contrast arteriography, MRI arteriography); 3. Ankle Brachial Pressure Index < 0.90 in non-diabetics
	Vascular Immune reconstitution syndrome (IRIS)	A new onset vasculopathy within 6 months of starting ART
	All-cause death and vascular-related deaths	Death (of any or vascular cause) that occurs after recruitment into the study

For peer review only - http://bmjopen.bmj.com/site/about/guidelines.xhtml

BMJ OPEN

For peer review only

For peer review only - http://bmjopen.bmj.com/site/about/guidelines.xhtml

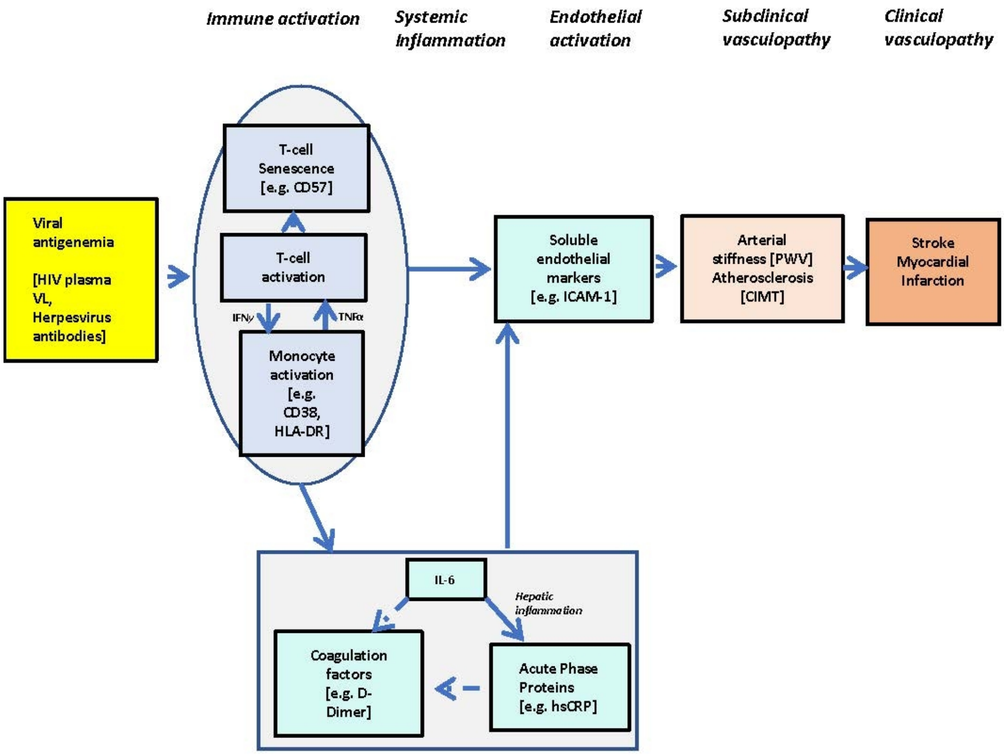

Figure 1

90x90mm (300 x 300 DPI)

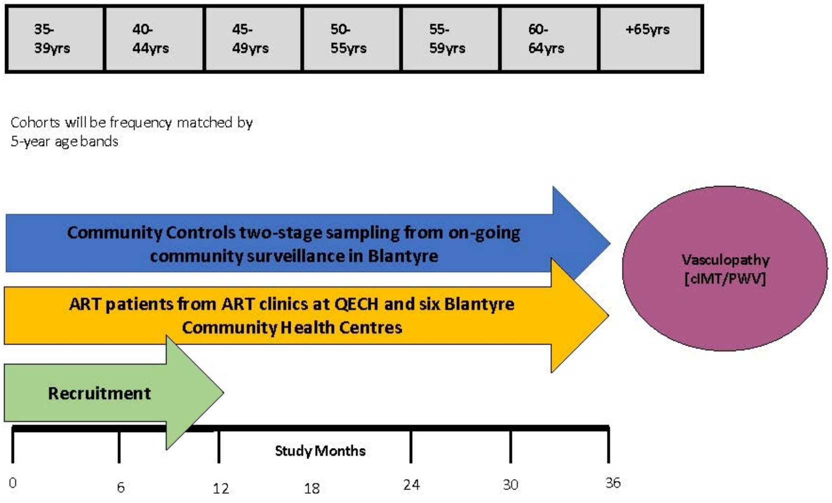

Figure 2

90x90mm (300 x 300 DPI)

RHICCA – Clinical endpoint diagnostic criteria vs 3.5 Apr 2018

RHICCA
Reactivation of herpesviruses and inflammation as cardiovascular and cerebrovascular risk factors in ART initiators

	CONFIRMED	PROBABLE
**INFECTIONS**		
Aspergillosis, invasive pulmonary	**Confirmed: 1 + 2 + 3:**<br>1. Compatible clinical course **(Appendix 13)**,<br>2. CXR abnormality compatible with aspergillosis;<br>3. invasive mycelia consistent with Aspergillus on lung biopsy or positive culture of lung tissue or **positive culture of sputum** collected by any method	**Probable: 1 + 2 + 3:**<br>1. Compatible clinical course **(Appendix 13)**,<br>2. CXR abnormality compatible with aspergillosis;<br>3. invasive mycelia consistent with Aspergillus on tissue biopsy or positive culture at a normally sterile site (e.g., blood) apart from the lungs.
Aspergillosis, other invasive	**Confirmed: 1 + 2 + 3:**<br>1.compatible clinical course **(Appendix 11)**,<br>2. invasive mycelia consistent with Aspergillus on tissue biopsy or clinical evidence of infection,<br>3. positive culture from the affected tissue	**Probable: 1 + 2:**<br>1.clinical evidence of invasive infection **(Appendix 11)**, 2.invasive mycelia consistent with Aspergillus on tissue biopsy or positive culture at a normally sterile site (e.g., blood) apart from the involved tissue
Bartonellosis	**Confirmed 1+ 2:**<br>1.Clinical or histologic evidence of bacillary angiomatosis or bacillary peliosis,<br>2. a positive culture or PCR for *B. quintana* or *B. henselae*	**Probable 1 + 2:**<br>1.Clinical evidence of bacillary angiomatosis or bacillary peliosis **(Appendix 12**),<br>2. positive silver stain for bacilli from a skin lesion or an affected organ
Candidiasis,oral	**Confirmed:1 + 2 + 3:**<br>1. Macroscopic appearance on examination of the mouth<br>2. microscopic evidence of yeasts or pseudo hyphae<br>3. no evidence of oesophageal involvement -	**Probable: 1 + 2 + 3:**<br>1. a clinical diagnosis of oral candidiasis and/or microscopic evidence of yeasts or pseudo hyphae<br>2. clinical response to treatment<br>3. no evidence of oesophageal involvement
Candidiasis of bronchi, trachea, or lungs	**Confirmed:1 + 2:**<br><br>Macroscopic appearance at bronchoscopy or autopsy<br>microscopic evidence of yeasts or pseudo hyphae	**None**
Candidiasis, esophageal	**Confirmed: 1 + 2:**<br>1. Macroscopic appearance at esophagoscopy or autopsy.<br>2. microscopic evidence of yeasts or pseudo hyphae	**Probable: 1 + 2 + 3:**<br>1. Recent onset of retrosternal pain or difficulty on swallowing.<br>2. a clinical diagnosis of oral candidiasis, endoscopic visualization of candidiasis and/or microscopic evidence of yeasts or pseudo hyphae from oropharyngeal mucosa<br>3. clinical response to treatment

For peer review only

For peer review only - http://bmjopen.bmj.com/site/about/guidelines.xhtml

RHICCA – Clinical endpoint diagnostic criteria vs 3.5 Apr 2018

RHICCA
Reactivation of herpesviruses and inflammation as cardiovascular and cerebrovascular risk factors in ART initiators

	CONFIRMED	PROBABLE
**INFECTIONS (CONTINUED)**		
Cryptococcosis, extrapulmonary (not meningitis)	**Confirmed: 1 or 2 or 3:**<br><br>From tissue other than lung or hilum:<br>1. microscopic demonstration of narrow based budding yeast<br>2. positive culture,<br>3. antigen detention	**None**
Cryptococcosis meningitis	**Confirmed: 1 or 2 or 3 or 4:**<br>1. Brain histopathology microscopic demonstration of narrow based budding yeast<br>2. CSF evidence of India ink test<br>3. CSF evidence of positive culture<br>4. CSF evidence of positive antigen detection	**None**
Cryptosporidiosis	**Confirmed: 1 + 2**<br>1. Diarrhea for > 1 month<br>2. positive microscopy	**None**
CMV retinitis	Autopsy demonstration	**Probable 1 + 2:**<br>1. Typical appearance on fundoscopy of discrete patches of retinal whitening, spreading along blood vessels.<br>2. Associated vasculitis, hemorrhage and necrosis, confirmed by ophthalmologist

	CONFIRMED	PROBABLE
**INFECTIONS (CONTINUED)**		
HZV single dermatome	**Confirmed 1+2:**<br>1.multiple ulcerated lesions affecting at least 1 dermatome, and/or 1 or more contiguous dematomes;<br><br>2. positive culture, PCR, or antigen assay from affected tissue	**Probable 1+ 2:**<br>1.multiple typical ulcerated lesions affecting at Least 1 dermatome, and/or 1 or more contiguous dermatomes;<br><br>2. response to an antiviral active against HZV unless resistance is demonstrated
HZV, disseminated	**Confirmed 1+2:**<br>1. multiple ulcerated lesions affecting at least 2 non-contiguous dermatomes, or with generalized cutaneous dissemination HZV involvement of the lung, liver, brain, or other internal organs<br>2. positive culture, PCR, or antigen assay from affected tissue	**Probable 1+2:**<br>1. multiple typical ulcerated lesions affecting at least 2 non-contiguous dermatomes, or with generalized cutaneous dissemination<br>2. response to an antiviral active against HZV unless resistance is demonstrated
HSV mucocutaneous ulceration	**Confirmed 1 +2:**<br>1.Ulceration for > 1 Month<br>2. Histology, culture, PCR, or detection of antigen from affected tissue	**Probable 1 + 2:**<br>1. Typical HSV ulceration for > 1 month,<br>2. response to an antiviral active against HZV unless resistance is demonstrated
Histoplasmosis, disseminated or extrapulmonary	**Confirmed 1+2:**<br>1.Compatible symptoms,<br>2. histology or culture or elevated blood or urine antigen levels	None
Isosporiasis	**Confirmed 1 + 2:**<br>1.Diarrhea for > 1 month<br>2.microscopic identification of *Isospora belli*	None

For peer review only

RHICCA – Clinical endpoint diagnostic criteria vs 3.5 Apr 2018

Microsporidiosis	**Confirmed 1 + 2:**<br>1.Diarrhea for > 1 month<br>2.Microscopic identification of Microsporidia	None
MAC and other mycobacterial disseminated diseas	**Confirmed 1 + 2:**<br>1. Fever, fatigue, anemia or diarrhea<br>2.positive culture from blood, body fluids or tissue other than pulmonary, hilar or stool	**Probable 1+2+3:**<br>1. Fever, fatigue, anemia or diarrhea<br>2. **AFB** or positive direct MAC PCR in blood, body fluids or tissue other than pulmonary, hilar or stool<br>3. no concurrent non-pulmonary TB

-

	CONFIRMED	PROBABLE	POSSIBLE
M. tuberculosis disease, pulmonary	**Confirmed 1+2:**<br>1.Compatible symptoms of fever, dyspnea, cough, weight loss or fatigue<br>2. culture or PCR from **sputum** or bronchial lavage or lung tissue	**Probable 1+2+3+4:**<br>1.Symptoms of fever, dyspnea, cough, weight loss or fatigue<br>2.abnormal chest X-ray,<br>3. AFBs seen in sputum or lavage or lung tissue but not grown in culture,<br>4.responds to treatment	**Possible 1+2+3+4:**<br>1.Symptoms of fever, dyspnea , cough, weight loss or fatigue<br>2.abnormal chest X-ray compatible with pulmonary TB (such as upper lobe cavitation, pleural exudate)<br>3. No other etiology for pulmonary symptoms and signs identified,<br>4.Responds to anti tuberculosis treatment
M. tuberculosis disease, Extrapulmonary (not meningitis)	**Confirmed 1+2:**<br>1. Compatible symptoms<br>2. culture or PCR or MTB Xpert from blood or affected tissue (i.e. pericardial, ascites, and lymph glands)	**Probable 1+2+3**:<br>1.Compatible symptoms<br>2. AFBs seen from affected tissue or blood<br>3.concurrent diagnosis of pulmonary TB or responds to treatment	**Possible 1+2+3:**<br>1.Compatible symptoms<br>2. No other etiology for symptoms and signs identified<br>3.concurrent diagnosis of pulmonary TB or responds to treatment
M. tuberculosis disease, meningitis	**Confirmed 1+2:**<br>**1.** Clinical symptoms of meningism **(Appendix 7)**<br>**2.** Tissue/CSF culture, or PCR, or AFB or MTB Xpert	**Probable 1+ a score ≥12 (Appendix: Table 2):**<br>1. Clinical symptoms of meningism **(Appendix 7)**<br>2. A score ≥12, based on clinical, CSF, cerebral brain imaging criteria or evidence of TB elsewhere	
Nocardiosis	**Confirmed 1+2:**<br>1. Clinical evidence of invasive Infection **(Appendix 8)**<br>2. a positive culture from the affected tissue or blood	**Probable 1+2:**<br>1. Clinical evidence of invasive Infection **(Appendix 8)**<br>2. microscopic evidence of bronchial weakly acid fast organisms from the affected tissue	

For peer review only

RHICCA – Clinical endpoint diagnostic criteria vs 3.5 Apr 2018

RHICCA
Reactivation of herpesviruses and inflammation as cardiovascular and cerebrovascular risk factors in ART initiators

Pneumocystis jirovecii pulmonary	**Confirmed 1+2:**<br>1. compatible clinical syndrome **(Appendix 9)**<br>2. microscopic or histological demonstration of *P. jirovecii* cysts in a pulmonary specimen	Probable 1+2+3+4+5<br>1. dyspnea or cough, or fever progressive over > 1 week<br>2. **diffuse chest x-ray abnormality** or, if on inhalational pentamidine, diffuse upper lung field abnormality<br>3. evidence of hypoxia<br>4. not suggestive of bacterial pneumonia (i.e., not purulent sputum or hemoptysis, no bacterial pathogen identified in blood or bronchial wash)<br>5. response to PcJ treatment
Pneumocystis jirovecii, extrapulmonary	**Confirmed 1+2:**<br>1. compatible clinical syndrome<br>2. microscopic or histological demonstration of *P. jirovecii* cysts in a tissue other than pulmonary specimen	None

For peer review only

RHICCA – Clinical endpoint diagnostic criteria vs 3.5 Apr 2018

RHICCA
Reactivation of herpesviruses and inflammation as cardiovascular and cerebrovascular risk factors in ART initiators

	CONFIRMED	PROBABLE
**INFECTIONS (CONTINUED)**		
Pneumonia, **SINGLE EPISODE (isolated)**<br>bacterial, excludes: (a) post-obstructive pneumonias, including in those with intrapulmonary/bronchial lesions, (b) aspiration pneumonia, including in those with decreased consciousness levels, (c) nosocomial ventilator-associated pneumonias	**Confirmed 1+2+3:**<br>pneumonia episodes must occur after enrollment;<br>1. Signs and symptoms suggestive of bacterial pneumonia (**appendix 10**)<br>2. Focal CXR abnormality compatible with bacterial pneumonia,<br>3. identification of a bacterial pathogen by positive blood culture, positive bronchoscopic sputum culture, detection of Legionella or pneumococcal antigen in urine or blood or diagnostic serologic findings	**Probable 1+2:**<br>pneumonia episodes must occur after enrollment;<br>1.Signs and symptoms suggestive of bacterial pneumonia (**Appendix 10**)<br>2. Focal CXR abnormality compatible with Bacterial pneumonia
Pneumonia, **recurrent**<br>bacterial, excludes: (a) post-obstructive pneumonias, including in those with intrapulmonary/bronchial lesions, (b) aspiration pneumonia, including in those with decreased consciousness levels, (c) nosocomial ventilator-associated pneumonias	**Confirmed 1+2+3+4+5**<br>Second pneumonia episodes must occur after enrollment and after an isolated pneumonia, within 365 days of the first episode and with strong clinical evidence that the first episode was cured;<br>1. Signs and symptoms of second event suggestive of bacterial pneumonia (**Appendix 10**)<br>2. Focal CXR abnormality compatible with bacterial pneumonia,<br>3. identification of a bacterial pathogen by positive blood culture, positive bronchoscopic sputum culture, detection of Legionella or pneumococcal antigen in urine or blood or diagnostic serologic findings<br>4. the second pneumonia had onset of symptoms < 365 days after the first episode<br>5. there is strong evidence that the first episode was cured such as an intervening clear CXR or absence of symptoms after > 1 month off antibacterial effective against pathogens commonly producing pneumonia	**Probable 1+2+3+4:**<br>Second pneumonia episodes must occur after enrollment and after an isolated pneumonia, within 365 days of the first episode and with strong clinical evidence that the first episode was cured;<br>1. Signs and symptoms suggestive of bacterial pneumonia (**Appendix 10**)<br>2. focal CXR abnormality compatible with bacterial pneumonia<br>3. the second pneumonia had onset of symptoms < 365 days after the first episode<br>4. there is strong evidence that the first episode was cured such as an intervening clear CXR or absence of symptoms after > 1 month off antibacterials effective against pathogens commonly producing pneumonia
PML (progressive multifocal leukoencephalopathy)	**Confirmed 1 or 2:**<br>1. positive histology,<br>2. compatible clinical (**Appendix 11**) and radiologic course and positive CSF PCR for JK virus	**Probable 1+2+3:**<br>1. Consistent symptoms (**Appendix 11)**,<br>2. brain image consistent with PML,<br>3. no response to toxo treatment or toxoplasma [illegible]
Salmonella blood stream infection or bacteraemia, isolated	**Confirmed 1:**<br>A septic episode must occur after enrollment;<br>1. Positive blood or tissue culture	None
Salmonella blood stream infection or bacteraemia, recurrent	**Confirmed 1:**<br>A second septic episode must occur after enrollment and after an isolated episode;<br>1. Has met the criteria of isolated Salmonella septicemia<br>2. Positive blood or tissue culture on the second episode<br>3. the second septicemia had onset of symptoms < 365 days after the first episode<br>4. the second septicemia must be due to a different Salmonella serotype or there must be strong evidence that the first episode was cured such as a negative blood culture off effective antibacterials for > 1 week or absence of symptoms off antibacterials for > 1 month	None

For peer review only

RHICCA – Clinical endpoint diagnostic criteria vs 3.5 Apr 2018

RHICCA
Reactivation of herpesviruses and inflammation as cardiovascular and cerebrovascular risk factors in ART initiators

Toxoplasmosis of brain	**Confirmed 1+2+3:**<br>1. Compatible clinical findings **(Appendix 12)**<br>2. Compatible radiological findings<br>3. Detection of T gondii in the **CSF** or brain tissue (i.e. microscopy or PCR)	**Probable 1+2+3:**<br>1. Symptoms of focal intracranial abnormality or decreased consciousness<br>2. brain image consistent with lesion(s) enhanced by contrast<br>3. positive toxoplasma serology or responds to treatment clinically or by scan

	CONFIRMED	PROBABLE
**NEOPLASMS**		
Cervical carcinoma, invasive	**Confirmed 1:**<br>1. Histology (NOT carcinoma-in-situ)	**None**
Kaposi sarcoma, (mucocutaneous or visceral)	**Confirmed 1:**<br>1. Histology	1. Highly typical appearance<br>2. persistence for > 1 month
Lymphoma, primary, of brain	**Confirmed 1:**<br>1. Histology of brain tissue	**Probable 1+2+3:**<br>1. Symptoms consistent with lymphoma<br>2. at least one CNS lesion with mass effect<br>3. lack of clinical or radiographic response at least 2 weeks of treatment for toxoplasmosis
Lymphoma, Hodgkin's	1. Histology	None
Lymphoma, non-Hodgkin's, all cell types	**Confirmed 1:**<br>1. Histology	None
**NEUROLOGICAL**		
HIV-related encephalopathy (including AIDS Dementia Complex)	None	**Probable 1+2+3+4:**<br>1. Cognitive or motor dysfunction interfering with usual activity, progressive over weeks or months<br>2. no other condition to explain the findings<br>3. brain image obtained and suggests no other causes<br>4. grade 2 or worse impairment in at least 2 domains by NARS (**appendix – table 1**) excluding abnormal domains at trial entry. (For persons with abnormal domains at entry worsening by at least two grades meets criteria.)

For peer review only - http://bmjopen.bmj.com/site/about/guidelines.xhtml

RHICCA – Clinical endpoint diagnostic criteria vs 3.5 Apr 2018

RHICCA
Reactivation of herpesviruses and inflammation as cardiovascular and cerebrovascular risk factors in ART initiators

CARDIOVASCULAR DISEASES		
Acute Myocardial Infarction	**Confirmed: One of the following 5 criteria (adapted from 2007 Universal Definition of Myocardial Infarction): [1 + (2 or 3 or 6)] or 4 or 5**<br>1. Rise and/or fall of cardiac biomarkers (preferably troponin), with at least one value above 99th percentile of upper reference limit (URL);<br>2. Occurrence of a compatible clinical syndrome, including symptoms (such as chest pain **– see Appendix 1**) consistent with myocardial ischemia;<br>3. ECG changes indicative of new ischemia (new ST-changes or new left bundle branch block [LBBB]), or development of pathological Q waves on the ECG;<br>4. Sudden unexpected cardiac death involving cardiac arrest before biomarkers are obtained or before a time when biomarkers appear, along with (a) new ST-changes or new LBB, or (b) evidence of fresh thrombus on coronary angiography or at autopsy<br>5. Pathologic findings of acute myocardial infarction (including acute MI demonstrated as the cause of death on autopsy)<br>6. Development of a) evolving new Q waves, or b) evolving ST elevation, preferably based on at least 2 ECGs taken during the same hospital admission	**Probable 1 and 2:**<br>1. Occurrence of a compatible clinical syndrome (**Appendix 1**), including symptoms (such as chest pain) consistent with myocardial ischemia)<br>2. Development of a) evolving new Q waves, or b) evolving ST elevation, preferably based on at least; ECGs taken during the same hospital admission.
Peripheral vascular disease	**Confirmed (1+2) or (1+3):**<br>1. Compatible clinical signs and symptoms (**see Appendix 3**)<br>2. Positive results on diagnostic imaging studies (e.g., Doppler ultrasound, contrast arteriography, MRI arteriography); 3. Ankle Brachial Pressure Index < 0.90 in non-diabetics	**Probable 1:**<br>1. Compatible clinical signs and symptoms (**see Appendix 3**)
Stroke	**Confirmed (1+2) or 3 or 4 or 5:**<br>1. Acute onset with a clinically compatible course, including unequivocal objective findings of a localizing neurologic deficit **(appendix 4)**;<br>2. CT or MRI compatible with diagnosis of stroke and current neurologic signs and symptoms<br>3. Stroke diagnosed as cause of death at autopsy<br>4. Compatible clinical history and positive lumbar puncture compatible with subarachnoid hemorrhage<br>5. Death certificate or death note from medical record listing stroke as cause of death	**Probable (1+2) or (1+3):**<br>1. Acute onset with a clinically compatible course, including unequivocal objective findings of a localizing neurologic deficit **(appendix 4)**;<br>2. Positive lumbar puncture compatible with subarachnoid hemorrhage<br>3. Death certificate or death note from medical record listing stroke as cause of death
Congestive heart failure	**Confirmed (1+2) or (1+3) or (1+4):**<br>1. Clinical signs and symptoms compatible with left or right sided heart failure [**Appendix 5]** without an alternative explanation<br>2. Hemodynamic measurements, radionucleotide ventriculography, echocardiogram, cardiac catheterization, or multiple gated acquisition scan showing a decreased ejection fraction of < 45%<br>3. Echocardiogram, cardiac catheterization or other studies showing evidence of increased left atrial pressure or right heart failure;<br>4. Elevated levels of Brain Natriuretic Peptide (BNP) or NT-proBNP	**Probable 1+2+3:**<br>1. Clinical signs and symptoms compatible with left or right sided heart failure [**Appendix 5]** without an alternative explanation<br>2. Chest x-ray or other imaging study showing evidence of congestive heart failure, including cardiac enlargement;<br>3. Documentation of treatment for congestive heart failure

For peer review only - http://bmjopen.bmj.com/site/about/guidelines.xhtml

RHICCA – Clinical endpoint diagnostic criteria vs 3.5 Apr 2018

RHICCA
Reactivation of herpesviruses and inflammation as cardiovascular and cerebrovascular risk factors in ART initiators

Coronary artery disease requiring drug treatment	**Confirmed (1 or 2) + 3:**<br><br>1. Evidence of myocardial ischemia based on either diagnostic imaging (such as a stress echocardiogram or thallium scan) or diagnostic changes on an electrocardiogram (such as during stress testing or an episode of chest pain)<br>2. Evidence of coronary artery disease based on coronary angiography or other diagnostic imaging<br>3. Use of medication given to treat or prevent angina (e.g., nitrates, beta blockers, calcium channel blockers)	**Probable 1+2:**<br><br>1. Other evidence of myocardial ischemia and/or coronary artery disease (including that based primarily upon symptoms and clinical presentation, such as chest pain with exertion)<br>2. Use of medication given to treat or prevent angina (e.g., nitrates, beta blockers, calcium channel blockers)
Deep vein thrombosis	**Confirmed 1:**<br>1. Diagnosis of deep vein thrombosis (DVT) by contrast venography, or **ultrasonography** other comparable imaging techniques;	**Probable (1)+2+3:**<br>1. An elevated **D-dimer test**;<br>2. A score on the Wells Clinical Prediction Rule for DVT of ≥ 3 points;<br>3. Absence of alternative diagnosis as likely or greater than that of deep venous thrombosis.<br>**Wells Clinical Prediction Rule for DVT (Appendix 6)**
**SYSTEMIC DISEASES**		
Anaemia<br>NOT FOR ERC<br>TO BE DETERMINED RETROSPECTIVELY	**Confirmed 1**<br>Classified according to both WHO and DAIDS thresholds for severe/grade 3-4 anaemia	
Chronic Kidney disease	**Confirmed: 1 or 2**<br><br>1. Kidney damage for >3 months, as defined by structural or functional abnormalities of the kidney with or without decreased GRF, manifest by either;<br>- Pathological abnormalities; or<br>- Markers of kidney damage, including abnormalities in the composition of the blood or urine or abnormalities in imaging results<br>2. GFR <60mL/min/1.73m2 for >3months, with or without kidney disease (estimated by CKD-EPI)	**Confirmed: 1 or 2**<br><br>1. Isolated Kidney damage, as defined by structural or functional abnormalities of the kidney with or without decreased GRF, manifest by either;<br>- Pathological abnormalities; or<br>- Markers of kidney damage, including abnormalities in the composition of the blood or urine or abnormalities in imaging results<br>2. Isolated GFR <60mL/min/1.73m2, with or without kidney disease (estimated by CKD-EPI)
End-stage renal disease	**Confirmed: 1**<br>1.Hemodialysis or peritoneal dialysis documented in a clinical note for a period of at least three months;	**Probable: 1**<br>1. Hemodialysis or peritoneal dialysis documented in a clinical note for a period of at least one month and up to the time of death in a patient who dies within three months after dialysis begins
Diabetes Mellitus<br><br>NOT FOR ERC<br>TO BE DETERMINED RETROSPECTIVELY	**Confirmed: 1 or 2 or 3 or 4**<br><br>1. Symptoms of diabetes plus casual plasma glucose concentration ≥ 200 mg/dL (11.1 *mmol/L*). (Casual is defined as any time of day without regard to time since last meal. The classic symptoms of diabetes include polyuria and polydipsia.)<br><br>2. Fasting plasma glucose ≥ 126 mg/dL (7.0 *mmol/L*). (Fasting is defined as no caloric intake for at least 8 hours.)<br><br>3. 2-hour post-load glucose ≥ 200 mg/dL (11.1 *mmol/L*) during an oral glucose tolerance test. (The test should be performed as described by WHO, using glucose load containing the equivalent of 75 g anhydrous glucose dissolved in water.)<br><br>4.An **HbA1c** of 48mmol/mol (6.5%) or above.	**None**

For peer review only

For peer review only - http://bmjopen.bmj.com/site/about/guidelines.xhtml

RHICCA – Clinical endpoint diagnostic criteria vs 3.5 Apr 2018

Decompensate Liver disease	**Confirmed: 1+2**<br>1. Histologic, radiographic, or ultrasound evidence of cirrhosis, as documented by one of the following:<br>a. Histologic evidence of cirrhosis obtained by liver biopsy or autopsy<br>b. MRI or CT consistent with cirrhosis<br>c. A positive result on ultrasound imaging consistent with cirrhosis<br><br>2. Clinical evidence of decompensation, as documented by one of the following, and without an alternative explanation:<br>a. Ascites<br>b. Hepatic encephalopathy<br>c. Bleeding from gastric or esophageal varices<br>d. Spontaneous bacterial peritonitis	**Probable: 1**<br><br>1. Clinical evidence of decompensation, as documented by one of the following, and without an alternative explanation:<br>a. Ascites<br>b. Hepatic encephalopathy<br>c. Bleeding from gastric or esophageal varices<br>d. Spontaneous bacterial peritonitis
Hypertension	**Confirmed:** 1 or 2<br>1. An average of three blood pressure (BP) readings of systolic of 140 mm Hg or above and/or diastolic of 90 mm Hg or above on the same day and persist 5-7 days after the initial reading.<br>2. An isolated reading of 140mg systolic or 90mg diastolic and presence of the following end-organ disease:<br>a. Cardiac (i.e. left ventricular hypertrophy meeting the ECG criteria [**Appendix 2**] on evidence on cardiac echocardiogram)<br>b. Renal (i.e. microalbuminuria [urinary albumin excretion of 30-300mg/dl], elevated creatinine, reduced estimated GFR (60-90ml/min)<br>c. Retinal(i.e. hypertensive retinal changes)<br>d. Vascular disease (i.e. stroke [persisting on day 7], peripheral vascular disease, myocardial infarction, coronary artery disease requiring drug treatment, congestive cardiac failure)	**Probable: 1**<br><br>1. An average of three BP readings of systolic of 140 mm Hg or above and/or diastolic of 90 mm Hg or above on the same day.
Hyperlipidemia<br><br>NOT FOR ERC<br>TO BE DETERMINED RETROSPECTIVELY	**Confirmed: 1 or 2**<br>1. Fasting total cholesterol >200mg/dl (>5.2 mmol/L) or LDL cholesterol >130mg/dl (>3.4mmol/l) or Triglycerides >150 mg/dl (1.7 mmol/L)<br>2. Non-fasting total cholesterol >240mg/dl (>6.2 mmol/L) or LDL cholesterol >160mg/dl (>4.1 mmol/L) or Triglycerides >200 mg/dl (2.3mmol/L)	**None**
HIV wasting syndrome	None	**Probable:1 + 2 + 3**<br>1. unexplained, involuntary weight loss >10% from baseline,<br>2. persistent diarrhea with > 2 liquid stools/d for > 1 month or weakness for > 1 month or fever for > 1 month,<br>3. tests for alternate causes of weight loss, such as cancer, TB, MAC, cryptosporidiosis or other specific causes of weight loss, if obtained, should be negative

For peer review only - http://bmjopen.bmj.com/site/about/guidelines.xhtml

RHICCA – Clinical endpoint diagnostic criteria vs 3.5 Apr 2018

Appendix

1. Clinical syndrome of Myocardial infarction (a+ b +d) or (c+d)
    a. Chest pain (with associated clamminess, pallor)
    b. Radiation to the upper extremity and jaw
    c. Epigastric discomfort with exertion or at rest
    d. Severe discomfort lasting for more than 20 minutes

2. ECG criteria for LVH

Any two of the following 3 criteria's should be met:

Sokolow Lyon Criteria<br><br>• S in V_1 or V_2 + R in V_5 or V_6 (whichever is larger) ≥ 35 mm (≥ 7 large squares)<br>• R in aVL ≥ 11 mm<br><br>**Meets all of Sokolow Lyon criteria to be diagnostic**	
Cornell voltage criteria<br><br>ECG diagnosis of LVH involve measurement of the sum of the R wave in lead aVL and the S wave in lead V_3. The Cornell criteria for LVH are:<br><br>• S in V_3 + R in aVL > 28 mm (men)<br>• S in V_3 + R in aVL > 20 mm (women)<br><br>**Meets all of Cornell voltage criteria to be diagnostic**	
Romhilt-Estes point score system ECG Criteria<br>Voltage Criteria (any of):<br>1. R or S in limb leads ≥20 mm<br>2. S in V_1 or V_2 ≥30 mm<br>3. R in V_5 or V_6 ≥30 mm<br>---<br>ST-T Abnormalities:<br><br>1. ST-T vector opposite to QRS without digitalis<br>2. ST-T vector opposite to QRS with digitalis<br>3. Negative terminal P mode in V_1 1 mm in depth and 0.04 sec in duration (indicates left atrial enlargement)<br>4. Left axis deviation (QRS of -30° or more)<br>5. QRS duration ≥0.09 sec<br>6. Delayed R wave peak time (intrinsicoid deflection) in V_5 or V_6 (>0.05 sec)<br><br>**Romhilt-Estes point score >4 is diagnostic**	Points<br><br>3<br><br><br><br><br><br>3<br>1<br>3<br><br>2<br>1<br>1

3. Clinical syndrome of Peripheral vascular disease (a+ (b or c or d)
    a. Painful cramping in the hip, thigh or calf muscles after certain activities, such as walking or climbing stairs (claudication)
    b. femoral bruit
    c. decreased peripheral pulses
    d. change in color or temperature of limb suggesting peripheral arterial disease

For peer review only

For peer review only - http://bmjopen.bmj.com/site/about/guidelines.xhtml

4. Clinical syndrome of stroke; should meet the 3 criteria's;

1. **Sudden onset**	
2. **Focal deficit** (or global disturbance but not seizures)	**Large artery disease (anterior circulation syndrome)**<br>Hemi-paresis + Hemi-sensory loss + higher cortical dysfunction (gaze paresis, language impairment [expression + comprehension], visual field defect, hemi-neglect)<br><br>**Large artery disease (posterior circulation syndrome)**<br>Vertigo, visual field defect, gaze paresis, double vision, swallowing difficultly, crossed signs [contralateral limb weakness and ipsilateral cranial nerves abnormality], ataxic limb and gait, drowsy/loss of consciousness<br><br>**Small vessel disease (lacunar syndrome)**<br>Pure hemi-sensory loss<br>Pure hemiparesis<br>Pure sensorimotor<br>Pure ataxic hemiparesis (including dysarthria-clumsy hand syndrome)<br><br>Thunderclap headache*
3. **Lasting > 24 hours** (<24 hours is a TIA)	
*seen in those with a suspicion of subarachnoid or venous stroke. In this case criteria 1 and 3 does not necessarily have to be met	

5. Clinical syndrome of congestive heart failure:

Using the Framingham criteria relies on clinical signs and symptoms; 1 or more major and two or more minor criteria are clinically suggestive of heart failure:

Major criteria

A. Acute pulmonary edema
B. Cardiomegaly
C. Hepatojugular reflex
D. Neck vein distention
E. Paroxysmal nocturnal Dyspnea or Orthopnea
F. Pulmonary crackles
G. Third Heart Sound (S3 Gallup Rhythm)

Minor Criteria

A. Ankle edema
B. Dyspnea on exertion
C. Hepatomegaly
D. Nocturnal cough
E. Pleural Effusion
F. Tachycardia (Heart Rate >120 beats per minute)

For peer review only

For peer review only - http://bmjopen.bmj.com/site/about/guidelines.xhtml

RHICCA – Clinical endpoint diagnostic criteria vs 3.5 Apr 2018

RHICCA
Reactivation of herpesviruses and inflammation as cardiovascular and cerebrovascular risk factors in ART initiators

6. Wells Clinical Prediction Rule for DVT (Adapted from: Wells PS et al. Lancet 1997;350:1796).

One point for each of the following:

- Active cancer (treatment ongoing or within previous 6 months, or palliative)
- Paralysis, paresis, or plaster immobilization of lower extremities
- Recently bedridden for more than 3 days, or major surgery, within 4 weeks
- Localized tenderness along distribution of the deep venous system
- Entire leg swollen
- Calf swelling by more than 3 cm when compared with the asymptomatic leg (measured 10 cm below tibial tuberosity)
- Pitting edema (greater in the symptomatic leg)
- Collateral superficial veins (non-varicose)

7. Clinical symptoms of meningism
   Meninigism is the triad of nuchal **rigidity** (neck stiffness), **photophobia** (intolerance of bright light) and **headache**.

8. Clinical symptoms of norcardia

   Symptoms vary and depend on the organs involved.

   If in the lungs, symptoms may include:
   - Chest pain when breathing (may occur suddenly or slowly)
   - Coughing up blood
   - Fevers
   - Night sweats
   - Weight loss

   If in the brain, symptoms may include:
   - Fever
   - Headache
   - Seizures
   - If the skin is affected, symptoms may include:
   - Skin breakdown
   - Skin breakdown and abnormal passage or draining tract (fistula)
   - Ulcers or nodules with infection sometimes spreading along lymph nodes

   Some people with nocardia infection have no symptoms.

9. Symptoms of Pneumocystis Pneumonia
   - Fever.
   - Mild and dry cough or wheezing.
   - Shortness of breath, especially with activity.
   - Rapid breathing.
   - Fatigue.
   - Major weight loss.
   - Chest pain when you breathe.

10. Clinical syndrome of bacterial pneumonia
   - cough with thick yellow, green, or blood-tinged mucus.
   - chest pain that worsens when coughing or breathing.
   - sudden onset of chills.
   - fever of 102°F or above (fever lower than 102°F in older persons)
   - headache.
   - muscle pain.
   - breathlessness or rapid breathing.

For peer review only

For peer review only - http://bmjopen.bmj.com/site/about/guidelines.xhtml

RHICCA – Clinical endpoint diagnostic criteria vs 3.5 Apr 2018

11. Clinical finding of Central Nervous System PML
    - Deficits in motor function, especially **weakness** and **clumsiness**, are common
    - associated altered mental state or behaviour and fever

12. Clinical finding of CNS toxoplasmosis
    - Headaches
    - Seizures
    - Focal neurological deficit of a subacute onset
    - confusion and coma
    - A lung infection, causing cough, fever, and shortness of breath may co-exist.
    -

13. Clinical symptoms suggest of Aspergillosis;

- Fever and chills.
- Cough that brings up blood-streaked sputum (hemoptysis)
- Severe bleeding from the lungs.
- Shortness of breath.
- Chest or joint pain.
- Headaches or eye symptoms.
- Nosebleed
- Facial swelling on one side

Table 1: Abbreviated NARS (Neuropsychiatric AIDS Rating Scale) Grading for HIV Encephalopathy
Adapted from: Price RW, Brew BJ. The AIDS dementia complex. J Infect Dis 1988; 158 (5): 1079-83, and Hughes CP, Berg L, Danziger WL. A new clinical scale for the staging of dementia. Brit J Psych 1982; 140: 566-92.

NARS stage	Cognitive-Behavioral Domains					
	**Orientation**	**Memory**	**Motor**	**Behavior**	**Problem solving**	**Activities of daily living**
**0.5**	fully oriented	complains of memory problems	fully ambulatory slightly slowed movements	normal	has slight mental slowing	slight impairment in business dealings
**1**	fully oriented, may have brief periods of “spaciness"	mild memory problems	balance, co-ordination and handwriting difficulties	more irritable, labile or apathetic, withdrawn	difficulty planning and completing work	can do simple daily tasks, may need prompting
**2**	some disorientation	memory moderately impaired, new learning impaired	ambulatory but may require walking aid	some impulsivity or agitated behavior	severe impairment, poor social judgement, gets lost easily	needs assistance with ADLs
**3**	frequent disorientation	severe memory loss, only fragments of memory remain	ambulatory with assistance	may have organic psychosis	judgement very poor	cannot live independently
**4**	confused and disoriented	virtually no memory	bedridden	mute and unresponsive	no problem solving ability	nearly vegetative

For peer review only
For peer review only - http://bmjopen.bmj.com/site/about/guidelines.xhtml

RHICCA – Clinical endpoint diagnostic criteria vs 3.5 Apr 2018

RHICCA

Reactivation of herpesviruses and inflammation as cardiovascular and cerebrovascular risk factors in ART initiators

For peer review only

RHICCA – Clinical endpoint diagnostic criteria vs 3.5 Apr 2018

RHICCA
Reactivation of herpesviruses and inflammation as cardiovascular and cerebrovascular risk factors in ART initiators

Table 2: Diagnostic criteria for classification of definite, probable, possible, and not tuberculosis meningitis (Marais S, et al. Lancet Infect Dis 2010)

	Diagnostic score
**Clinical criteria**	(Maximum category score=6)
Symptom duration of more than 5 days	4
Systemic symptoms suggestive of tuberculosis (one or more of the following): weight loss (or poor weight gain in children), night sweats, or persistent cough for more than 2 weeks	2
History of recent (within past year) close contact with an individual with pulmonary tuberculosis or a positive TST or IGRA (only in children <10 years of age)	2
Focal neurological deficit (excluding cranial nerve palsies)	1
Cranial nerve palsy	1
Altered consciousness	1
**CSF criteria**	(Maximum category score=4)
Clear appearance	1
Cells: 10–500 per µl	1
Lymphocytic predominance (>50%)	1
Protein concentration greater than 1 g/L	1
CSF to plasma glucose ratio of less than 50% or an absolute CSF glucose concentration less than 2.2mmol/L	1
**Cerebral imaging criteria**	(Maximum category score=6)
Hydrocephalus	1
Basal meningeal enhancement	2
Tuberculoma	2
Infarct	1
Pre-contrast basal hyperdensity	2
Evidence of tuberculosis elsewhere	(Maximum category score=4)
**Chest radiograph** suggestive of active tuberculosis: signs of tuberculosis=2; miliary tuberculosis=4	2/4
**CT/ MRI/** ultrasound evidence for tuberculosis outside the CNS 2	2
**AFB** identifi ed or *Mycobacterium tuberculosis* cultured from another source—ie, sputum, lymph node, gastric washing, urine, blood culture	4
Positive commercial *M tuberculosis* NAAT from extra-neural specimen	4

Exclusion of alternative diagnoses

An alternative diagnosis must be confirmed microbiologically (by stain, culture, or NAAT when appropriate), serologically (eg, syphilis), or histopathologically (eg, lymphoma). The list of alternative diagnoses that should be considered, dependent upon age, immune status, and geographical region, include: pyogenic bacterial meningitis, cryptococcal meningitis, syphilitic meningitis, viral meningo-encephalitis, cerebral malaria, parasitic or eosinophilic meningitis (*Angiostrongylus cantonesis*, *Gnathostoma spinigerum*, toxocariasis, cysticercosis), cerebral toxoplasmosis and bacterial brain abscess (space-occupying Lesion on cerebral imaging)and malignancy (eg, lymphoma)

TST=tuberculin skin test. IGRA=interferon-gamma release assay. NAAT=nucleic acid amplification test. AFB=acid-fast bacilli. The individual points for each criterion (one, two, or four points) were determined by consensus and by considering their quantified diagnostic value as defined in studies.

Key:
Bold text: of the options available likely to be the only tool available in a Malawi setting
Greyed out text: ideal investigation but not available in a Malawi setting

For peer review only - http://bmjopen.bmj.com/site/about/guidelines.xhtml

For peer review only

BMJ Open

Protocol for a longitudinal cohort study to evaluate the reactivation of herpesviruses and inflammation as cardiovascular and cerebrovascular risk factors in antiretroviral therapy initiators, in an African HIV-infected population (RHICCA)

Journal:	*BMJ Open*
Manuscript ID	bmjopen-2018-025576.R3
Article Type:	Protocol
Date Submitted by the Author:	26-Jul-2019

Complete List of Authors:	Peterson, Ingrid; Liverpool School of Tropical Medicine; Malawi Liverpool Wellcome Trust Clinical Research Programme<br>Ntsui, Ntobeko; University of Cape Town<br>Jambo, Kondwani; Liverpool School of Tropical Medicine; Malawi Liverpool Wellcome Trust Clinical Research Programme<br>Kelly, Christine; Malawi Liverpool Wellcome Trust Clinical Research Programme; University College Dublin<br>Huwa, Jacqueline; Malawi Liverpool Wellcome Trust Clinical Research Programme<br>Afran, Louise; Malawi Liverpool Wellcome Trust Clinical Research Programme<br>Tatuene, Joseph; University of liverpool, Institute of Infection and Global Health; Malawi-Liverpool-Wellcome Trust Clinical Research Programme,<br>Pett, Sarah; University College London, Institute of Infection and Global Health; University of New South Wales, Kirby Institute<br>Henrion, Marc; Malawi Liverpool Wellcome Trust Clinical Research Programme; Liverpool School of Tropical Medicine<br>Van Oosterhout, Joep; University of Malawi College of Medicine; Dignitas International<br>Heyderman, Robert; University College London, Division of Infection and Immunity; University of Malawi College of Medicine, Malawi Liverpool Wellcome Trust Clinical Research Programme<br>Mwandumba, Henry; Liverpool School of Tropical Medicine; Malawi Liverpool Wellcome Trust Clinical Research Programme<br>Benjamin, Laura; University of Liverpool Institute of Infection and Global Health, ; University College London Institute of Neurology,

**Primary Subject Heading**:	Cardiovascular medicine
Secondary Subject Heading:	HIV/AIDS, Immunology (including allergy), Infectious diseases, Global health
Keywords:	Ischaemic heart disease < CARDIOLOGY, EPIDEMIOLOGY, HIV & AIDS < INFECTIOUS DISEASES, Stroke medicine < INTERNAL MEDICINE, Cardiovascular

BMJ OPEN

Title: Protocol for a longitudinal cohort study to evaluate the reactivation of herpesviruses and inflammation as cardiovascular and cerebrovascular risk factors in antiretroviral therapy initiators, in an African HIV-infected population (RHICCA)

Authors:

Ingrid Peterson[1,2], Ntobeko Ntsui[3], Kondwani C Jambo[1,2], Christine Kelly[1,4], Jacqueline Huwa[1], Louise Afran[1], Joseph Kamtchum-Tatuene[5], Sarah Pett[6,7,8], Marc Yves Romain Henrion[1,2], Joep Van Oosterhout[9,10], Robert Heyderman[11], Henry C Mwandumba[1,2], Laura A Benjamin[4,12**] on behalf of the Investigators of the RHICCA study*

Affiliations:

1. Malawi-Liverpool-Wellcome Trust Clinical Research Programme, University of Malawi College of Medicine
2. Department of Clinical Sciences, Liverpool School of Tropical Medicine
3. Department of Medicine, University of Cape Town
4. HIV Molecular Research Group, University College Dublin
5. Institute of Infection and Global Health, University of Liverpool
6. Institute of Global Health, University College London
7. MRC CTU at UCL, Institute of Medicine, Clinical Trials and Methodology, University College London
8. Kirby Institute, University of New South Wales, Australia
9. Dignitas International, PO Box 071, Zomba, Malawi
10. College of Medicine, University of Malawi
11. Department of Infection and Immunity, University College London
12. Department of Brain Repair and Rehabilitation, Institute of Neurology, UCL

For peer review only - http://bmjopen.bmj.com/site/about/guidelines.xhtml

BMJ OPEN

*The Investigators of the RHICCA study

Brian Angus - Oxford Centre for Clinical Tropical Medicine, University of Oxford

Myles Connor - University of Edinburgh

Reena Dwivedi - Greater Manchester Neurosciences Centre, Salford Royal Foundation Trust

Lewis Haddow - Institute for Global Health, University College London

Terttu Heikinheimo-Connell - Hyvinkää Hospital, Department of Neurology, University of Helsinki

Elizabeth Joekes - Liverpool School of Tropical Medicine

Vanessa Kandoole - Department of Medicine, University of Malawi College of Medicine, Blantyre

Moffat Nyrienda - MRC Research Unit, Uganda

Kennedy Malisita- Department of Medicine, Queen Elizabeth Central Hospital

Jane Mallewa- Department of Medicine, University of Malawi College of Medicine, Blantyre

Elsayed Z. Soliman - School of Medicine, Wake Forest School of Medicine

Tom Solomon - Institute of Infection and Global Health, University of Liverpool

**Corresponding author

Laura Benjamin

Institute of Infection and Global Health,

Ronald Ross building,

The University of Liverpool,

L69 7BE, Liverpool,

United Kingdom

For peer review only - http://bmjopen.bmj.com/site/about/guidelines.xhtml

BMJ OPEN

l.benjamin@liverpool.ac.uk

Key words:

Cardiovascular, cerebrovascular, HIV, herpesvirus, Immune dysregulation,

Journal Guidance:

Abstract word count: 299/300

Article word count: 4095 /4000

Figure/Table: 5/5

For peer review only

BMJ OPEN

ABSTRACT

Introduction: In Sub-Saharan Africa, rising rates of cerebrovascular and cardiovascular disease (CBD/CVD) are intersecting with an aging HIV-infected population. The widespread use of antiretroviral therapy (ART) may confer an additive risk and may not completely suppress the risk associated with HIV infection. High-quality prospective studies are needed to determine if HIV-infected patients in Africa are at increased risk of CBD/CVD and to identify factors associated with this risk. This study will test the hypothesis that immune activation and dysfunction, driven by HIV and reactivation of latent herpesvirus infections lead to increased CBD/CVD risk in Malawian adults aged $\geq$ 35 years.

Methods and Analysis: We will conduct a single-centre 36-month prospective cohort study in 800 HIV-infected patients initiating antiretroviral therapy (ART) and 190 HIV-uninfected controls in Blantyre, Malawi. Patients and controls will be recruited from government ART clinics and the community, respectively and will be frequency-matched by 5-year age band and sex. At baseline and follow-up visits, we will measure carotid intima thickness (CIMT), pulse wave velocity (PWV) as surrogate markers of vasculopathy, and thus CBD include /CVD risk. Our primary exposures of interest cytomegalovirus and varicella zoster reactivation, changes in HIV plasma viral load, and markers of systemic inflammation and endothelial function. Multivariable regression models will be developed to assess the study's primary hypothesis. The occurrence of clinical CBD/CVD will be assessed as secondary study endpoints. ISRCTN registry https://doi.org/10.1186/ISRCTN42862937.

Ethics and dissemination: University of Malawi College of Medicine and Liverpool School of Tropical Medicine research ethics committees approved this work. Our goal is to understand the pathogenesis of CBD/CVD among HIV cohorts on ART, in sub-Saharan Africa, and provide data to inform future interventional clinical trials. This study runs between May 2017 and August 2020. Results of the main trial will be submitted for publication in a peer-reviewed journal.

For peer review only - http://bmjopen.bmj.com/site/about/guidelines.xhtml

STRENGTHS AND LIMITATIONS

- This is one of the first large-scale studies in Sub-Saharan Africa to explore the relationship between HIV infection, latent herpesviruses, inflammation and cardiovascular and cerebrovascular diseases, immediately after starting antiretroviral therapy (ART).
- Clinical events and death will be comprehensively reviewed through an end-point review committee, using strict diagnostic criteria for events based on those used in the INSIGHT network, or validated verbal autopsy for death with limited data.
- Because of the recent roll-out of ART in asymptomatic patients, there will be an absence of ART-naïve population, limiting our ability to explore the impact of ART.
- Approximately one-third of strokes will be asymptomatic. We anticipate not capturing some of these. However, multiple cerebral infarcts without a focal neurological deficit will manifest as cognitive impairment, which we will screen for, and corroborate with MRI imaging in a small number of symptomatic cases.
- Two-thirds of myocardial infarction will be silent and could potentially be missed. In a nested group, we will use a digital electrocardiogram to evaluate this further.

For peer review only - http://bmjopen.bmj.com/site/about/guidelines.xhtml

INTRODUCTION

The growing epidemic of cerebrovascular disease (CBD e.g. Stroke) and cardiovascular disease (CVD e.g. myocardial infarction) now intersects with the HIV epidemic[1]. Countries like Malawi, have an adult HIV prevalence of approximately 10%[2]. There is an increased life expectancy among people living with HIV, largely because of the successful scale-up of ART[3]. In Europe and the US, HIV is associated with a 50% increased risk of CVD compared to HIV-uninfected populations[4], attributable to long-term antiretroviral therapy (ART) use and HIV *per se*[4 5]. HIV infection is also associated with a 1.8 fold increased risk of all-cause heart failure in US veterans[6]. Our recent case-control study of stroke in Malawian adults is one of several examples that demonstrates a high risk of HIV infection associated with stroke and heart disease, pointing to a considerable and unappreciated CBD/CVD risk among HIV patients, in this setting[7-10].

There are reports of geographical differences in the distribution of CVD risk factors, supporting the argument that evidence derived from high-income countries cannot be applied to Sub-Saharan (SSA)[11]. Addressing this knowledge gap is essential to the development of clinical drug trials for primary prevention of CBD/CVD among individuals living with HIV. Vasculopathy due to accelerated atherosclerosis, arterial stiffening and vasculitis are the major mechanisms believed to underlie the CBD/CVD burden[12 13]. It is hypothesized that despite viral suppression, low-grade HIV virus replication and the associated host systemic inflammation are important drivers of this vasculopathy (Figure 1). In patients receiving ART, HIV antigenemia, partly resulting from HIV persistence in sanctuary sites, incomplete virologic suppression and virologic resurgence, drives the chronic immune activation observed in about 20% of ART patients in SSA[14]. This immune state is characterized by ongoing activation and senescence of cell-mediated immunity[15 16], increased monocyte/macrophage activation, stimulation of the interleukin-6 (IL-6) pathway and production of acute phase proteins[17-19]. Activation of the IL-6 pathway is established with atherosclerosis[20 21], and may also contribute to non-atherosclerotic vasculopathy. Inflammation alone confers a 2-fold increased risk of clinical CBD/CVD events[22]. The current push to introduce more effective ART regimens, and to start treatment soon after HIV diagnosis is made, may reduce inflammation and in turn, CBD/CVD risk[23]. However, there is

For peer review only - http://bmjopen.bmj.com/site/about/guidelines.xhtml

growing evidence of chronic inflammation in HIV despite achieving the goal of therapy, which is long-term suppression (<50 copies/mL) of plasma viral load, suggesting adjunctive therapy may be required.[24-26]

In addition to HIV, there is compelling evidence that reactivation of latent herpesviruses may be an important cause of vasculopathy. In HIV-uninfected elderly populations from high-income settings, latent cytomegalovirus (CMV) infection drives dysregulation of cell-mediated immunity[15 27-29], not dissimilar to what's described in HIV-associated immune activation[29]. CMV and other viral proteins have been found in atherosclerotic plaques[20]. Varicella-zoster virus (VZV) can directly infect the vascular endothelium to cause vasculitis and subsequent stroke and was found to be the commonest opportunistic infection (prevalence 15%) in a study of HIV-infected stroke patients in Malawi [12]. The seroprevalence of herpesviruses is high in SSA[30], particularly in HIV-infected populations[16].

The involvement of herpesviruses in the mechanistic pathway for CBD/CVD is compelling and may offer additional therapeutic avenues, especially for CMV and VZV. However, our understanding is incomplete, and its population impact is yet to be defined. It is important to determine if, in addition to ART, there is a role for other pharmacological interventions targeting latent viral infections or downstream inflammatory pathways to reduce vasculopathy in HIV-infected patients on ART. Previous work from North America supports the potential of treating reactivated herpesviruses[31]. Furthermore, there are opportunities for intervention using the recently licensed Letermovir; a treatment for CMV. By focusing on HIV and Herpes viral antigenemia and immune dysregulation as mechanisms of vasculopathy, this study will identify subgroups of HIV-infected patients on ART at high risk of CBD/CVD, the timing of CBD/CVD risk in such patients, as well as potential targets for intervention.

For peer review only - http://bmjopen.bmj.com/site/about/guidelines.xhtml

STUDY OBJECTIVES

This study will test the hypothesis that immune activation and dysfunction, driven by HIV and reactivation of latent CMV/VZV herpesvirus infections lead to increased CBD/CVD risk in adults aged $\geq$ 35 years in SSA. We will address this through the following objectives;

1) To determine if progression of the surrogate marker of CBD/CVD or occurrence of new-onset vasculopathy is higher in adults aged $\geq$ 35 years with HIV infection on ART compared to those without HIV.
2) To determine if progression of surrogate markers of CBD/CVD or occurrence of new-onset vasculopathy is higher in adults aged $\geq$ 35 years with HIV/herpes viral antigenemia or chronic immune activation compared to those without HIV/herpes viral antigenemia or chronic immune activation. Specifically, we will determine if progression of surrogate markers or new-onset vasculopathy is higher:
   a. in ART patients with reactivated latent herpes viral infection, compared to those without reactivated latent herpes viral infection.
   b. in ART patients with the highest 25% of markers for immune activation, inflammation or endothelial activation compared to the bottom 25%
   c. in ART patients with incomplete virologic suppression or virologic resurgence of HIV, compared to those with suppressed HIV plasma viral load.

The secondary study objectives are to determine if viral antigenemia or chronic immune activation increase occurrence of the following clinical events: 1) stroke, 2) myocardial infarction (MI), 3) angina (excluding MI), 4) peripheral vascular disease (PVD), 5) all-cause death/vascular-related death and 6) immune reconstitution inflammatory vasculopathy.

METHODS AND ANALYSIS

Study design

To address objective 1, we will conduct a single-center 36-month prospective cohort study in 800 HIV-infected patients initiating ART and 190 HIV-uninfected adults aged $\geq$ 35 years. HIV-infected and HIV-uninfected participants will be frequency matched by 5-year age band and sex. On a 6-monthly basis, we will measure markers of viral infection, inflammation and endothelial function along with surrogate markers for CBD/CVD (Table 1).

For peer review only - http://bmjopen.bmj.com/site/about/guidelines.xhtml

Study Setting

This study will recruit consecutive ART patients from the ART clinic of Queen Elizabeth Central Hospital (QECH), and ART clinics in several Blantyre City Community Health Centres (CHCs). These clinics collectively initiate over 100 HIV-infected patients aged $\geq$ 35 years onto ART each month. HIV-uninfected adults will be selected from pre-ART counseling sessions, and from randomly selected households in the community by two-stage random sampling (of households and individuals within households) from a previously enumerated sampling frame in the CHC catchment areas[32]. All study procedures will be conducted at QECH, which is located adjacent to the Malawi-Liverpool-Wellcome Trust Clinical Research Programme (MLW). QECH also hosts a 0.35T MRI imaging facility, which will contribute to characterizing our secondary endpoints.

Study Participants

Study inclusion criteria will be: a) age $\geq$ 35 years and b) resident in Blantyre. HIV-infected patients must further be: c) ART-naïve or initiated ART <10 days prior to enrolment and d) initiating standard first-line ART (in Malawi this is: Tenofovir [TDF]/Lamivudine [3TC]/Efavirenz [EFV]). Adult controls must further be: e) HIV-uninfected. Study exclusion criteria are: f) clinical history of CBD/CVD, g) pregnancy, h) critical illness or symptomatic anemia at baseline and i) enrollment in an intervention study. At the analysis stage abnormal PWV at baseline (as defined in Table 2) will be excluded for new-onset vasculopathy analysis but not for progression of vasculopathy. The same approach will be applied for baseline CIMT measurements. If the study participant becomes pregnant after recruitment, they will be withdrawn.

Justification of study inclusion and exclusion criteria is as follows; in many populations, CBD/CVD risk rises sharply from 35-years of age[33], thus individuals aged 35 and older will be eligible (recruitment of participants aged 35 -39 will be limited to 15% of the study sample to avoid overrepresentation). Restricting recruitment by age will enable this study to have greater statistical power. For clarity of etiologic inference, the study will assess the risk of new-onset vasculopathy not associated with pregnancy and thus exclude patients who are pregnant or with a history of CBD/CVD. To eliminate confounding by ART regimen, patients

For peer review only - http://bmjopen.bmj.com/site/about/guidelines.xhtml

must initiate on standard first-line ART (> 90% of ART patients in Blantyre do this). Critically ill patients are excluded primarily for ethical reasons.

Laboratory methods

Surface immunophenotyping of peripheral blood mononuclear cells

Immunophenotyping will be used to characterize peripheral blood mononuclear cells (PBMC) isolated from blood samples of HIV-uninfected and HIV-infected ART initiators. PBMCs will be harvested by density centrifugation using lymphoprep (Axis Shield, UK). PBMCs ($2x10^6$) will be stained with anti-CD45 PerCP CY5.5, anti-CD3 AF700, anti-CD4 BV421, anti-CD8 PE Dazzle, anti-CD38 BV605, anti-HLA-DR APC CY7, anti-CD57 APC, anti-PD1 PE CY7, anti-CTLA4 PE, and anti-CD223 FITC (all from eBiosciences, UK) to determine the expression of these markers on the surface of T-cells. In addition, ($2x10^6$) PBMCs stained with anti-CD16 BV421, anti-CD14 PE, anti-HLA-DR PerCP CY5.5, anti-CD45 AF700, anti-CCR2 BV605, anti-CD11b APC, anti-CX3CR1 PE Dazzle and anti-CD38 FITC (all from eBiosciences, UK) will be used for monocytes. Dead cells, $CD3^+$ T-cells, and $CD56^+$ NK cells will be excluded using: LIVE/DEAD™ Fixable Aqua Dead Cell Stain (Thermofisher, UK), anti-CD3 BV503 and anti-CD56 BV503 (eBiosciences, UK), respectively. Stained cells will be acquired on a BD LSR Fortessa flow cytometer (Becton Dickinson, USA) and data will be analyzed using FlowJo software version 10.0 (Tree Star, San Carlos, CA). For each stained sample analyzed, the median fluorescence intensity (MFI) for each parameter will be normalized to its respective unstained control.

For peer review only

Measurement of soluble markers of immune activation using multiplex bead array

A custom-made multiplex assay will be used to assess soluble markers of monocyte activation (CD163), systemic inflammation (Interleukin-6) and endothelial activation (Intracellular adhesion molecule 1) in plasma, isolated from blood samples of HIV-uninfected and HIV-infected ART initiators. Following isolation, plasma will be aliquoted and stored at -80°C until further use.

For peer review only - http://bmjopen.bmj.com/site/about/guidelines.xhtml

Assessment of exposure to human cytomegalovirus and varicella zoster virus by ELISA

Quantitative VIDAS CMV IgG and IgM (BioMerieux, USA) and VZV glycoprotein IgG Low-Level Enzyme Immunoassay Kit [VaccZyme™EIA], will be used to determine exposure to these viruses using a commercial enzyme-linked immunosorbent assay (ELISA) platform. These kits will detect VZV antigen to a sensitivity and specificity of 97.8% and 96.8% respectively and for CMV, 97.2% and 100% for IgG and 100% and 97.4% for IgM respectively[34 35]. Plasma samples from HIV-uninfected and HIV-infected ART initiators stored at -80°C following collection will be used for these assessments

HIV

HIV infection will be diagnosed using two rapid tests in parallel, EIA rapid tests (Determine HIV-1/2 [Abbott Laboratories, USA] and Uni-Gold HIV [Trinity Biotech PLC, Ireland]), will be used as a tiebreak). HIV-1 RNA levels in plasma will be measured using the Abbott Real-Time HIV-1 assay with a lower limit of detection of 150 copies/mL (Abbott Molecular, Germany), according to the manufacturer's instructions. CD4+ T -cell count measurements will be performed using BD FACS Count machine (Partec platform).

<u>Procedures</u>

Carotid-femoral pulse wave velocity (PWV)[36] and carotid intima-media thickness (CIMT)[37] measurement will be performed in accordance with expert consensus guidelines, using a standardized study protocol on the Vicorder system (SMART Medical, UK) and Philips CX50 machine (Philips healthcare, UK) respectively. CIMT measurements will be performed by three trained operators. The intra-class correlation coefficient will be used to assess the performance of the operators against that of a certified neurosonologist prior to study commencement.

For peer review only - http://bmjopen.bmj.com/site/about/guidelines.xhtml

Outcomes

Primary outcomes

Primary outcomes are the progression of surrogate markers of CBD/CVD, namely PWV and CIMT as well as the occurrence of new-onset vasculopathy defined by threshold values outlined in Table 2.

Secondary outcomes

Secondary outcomes are the following clinical events: 1) stroke, 2) myocardial infarction (MI), 3) unstable angina, 4) peripheral vascular disease (PVD), 5) all-cause death/ vascular death and 6) immune reconstitution inflammatory syndrome (IRIS) vasculopathy (Table 2). Changes in PWV or endothelial activation at 6 months post ART initiation will be interpreted as a subclinical vascular IRIS event. These outcomes will be assessed through active surveillance in QECH inpatient wards for admissions of study participants. To improve capture of clinical outcomes, we will conduct brief telephone interviews with study participants about CBD/CVD symptoms and hospitalizations between study visits and facilitate unsolicited participant self-report. Clinical events and deaths in study participants will be reviewed by an independent endpoint review committee (ERC), comprising of clinicians experienced in Endpoint review. Each event will be reviewed and adjudicated by the ERC Chair and 2 ERC reviewers, using a standard set of diagnostic criteria (Table 2 and Supplement – S1). The format of reporting will be based on modifications of the INSIGHT network clinical diagnostic criteria. Deaths will be reviewed by the ERC using the CoDe approach[23]. For death with limited clinical data, a validated verbal autopsy will be performed to ascertain the cause[38].

Exposures

The exposure for Primary Objective 1 will be HIV status. Yearly HIV rapid tests in HIV-uninfected adults will be performed to exclude those with new HIV infections (Figure 2).

Potential confounding and mediating factors will be recorded in study participants. This will include demographic factors, lifestyle and behavioral factors (e.g. cigarette smoking and alcohol consumption), chronic co-morbidities (i.e. hypertension, diabetes), cardiometabolic,

For peer review only - http://bmjopen.bmj.com/site/about/guidelines.xhtml

renal and hematological factors (i.e. full blood count, creatinine in urine and serum, body-mass-index, waist-to-hip ratio, random glucose, HbA1c, and lipid profile). Blood pressure will be measured at all study visits. Although vascular immune reconstitution inflammatory syndrome (IRIS) (Table 2) will be considered as a primary endpoint, non-vascular IRIS will be defined as a risk factor. Where feasible, we will conduct PCR tests for common causes of IRIS in blood or cerebrospinal fluid (CSF) samples. Adherence to ART and change of ART regimen will be assessed at all study visits through extraction of data from 'ART master cards'; this is a government-supported monitoring tool used by all patients on ART, in Malawi.

For Objective 2a-2c, markers of herpes and HIV viral antigenemia and immune inflammation will be measured according to the outline in Table 1. For primary objective 2a, reactivated latent herpes viral infections will be assessed by quantification of VZV, and CMV antibodies. We will estimate the risk of atherosclerosis and arterial stiffening associated with current herpesviruses reactivation at baseline, and sustained reactivation (i.e. those that continue to have a high titer from measurement at baseline to 6 months after ART initiation). Hyperactivation of B cells may result in an expansion of polyclonal antibodies and thus an overestimation of virus-specific antibody titers. To address this issue and make appropriate adjustments for hypergammaglobulinemia we will 1) measure more than one herpesviruses and 2) measure total IgG.

For primary objective 2b, markers of immune activation, inflammation, and endothelial activation will be measured (Figures 1 & Table 1). Quantitative cell surface immunophenotyping will be performed for CD4+ and CD8+ T-cell activation (e.g. HLA-DR) and senescence (e.g. CD57) in a subset of participants. In all study participants, at baseline, 6, 12, months, we will measure soluble markers associated with systemic inflammation and endothelial activation.

For primary objective 2c, incomplete viral response and viral rebound of HIV will be measured by quantitative PCR in patients on ART.[39] HIV viral load will be measured in patients on ART at 0, 6 and 12 months.

For peer review only - http://bmjopen.bmj.com/site/about/guidelines.xhtml

Data Collection Between May 2017 and August 2020

The two-stage screening will be conducted to find and recruit potential study participants. A trained field worker will first screen to assess eligibility for criteria (a)-(c) in pre-ART counseling sessions, and in individuals from randomly selected households in the community. Eligible participants will then be referred to QECH to complete screening for criteria (d)-(i) and if eligible, consented to participate in the study. At study visits, a tablet-based, standardized Open Data Kit (ODK) case report form (CRF) will be administered in one-on-one interviews by a study nurse to capture demographic and clinical data. Study data will be collected as outlined in Table 1. Daily upload of electronic data will occur with oversight from the data manager at MLW. We will collect up to 30ml of whole blood. An ACR dipstick test will be used to test for creatinine, proteinuria, and glucosuria. In a subset of participants, an electrocardiogram supported by a digital platform and echocardiogram will be performed at baseline, 6 and 24 months, as well as in any participant experiencing a clinical event suggestive of a cardiac etiology. To facilitate the retention and clinical referrals of participants, contact will be made every 3 months to assess the occurrence of clinical events. Participants who miss a scheduled study visit will be contacted by phone and/or visited at home to assess their willingness to maintain their participation and to record intervening clinical events. Recording and definitions of other clinical events, including HIV associated events will be evaluated by the ERC chair. SMS messages will be used for appointment reminders. Technical appendix, statistical code, and dataset will be made available from a data repository, after publication of our work.

Sample Size and Statistical Analysis

The required sample size for the study's primary objectives is 800 HIV-infected patients and 190 HIV-uninfected adults using standard, normal distribution approximation sample size formulas for comparing proportions in two groups of unequal size and based on the following assumptions: **a)** 18.4% of HIV positive study participants have abnormal PWV at baseline. We will exclude these participants from analysis. The 18.4% figure is informed by our ongoing studies of vasculopathy in HIV-infected patients, where this is the percentage of participants aged $\geq$35 years that have a PWV (>12 m/s). **b)** 20% of both HIV-infected patients and HIV-uninfected adults will be lost to follow-up, including by death and HIV

For peer review only

For peer review only - http://bmjopen.bmj.com/site/about/guidelines.xhtml

sero-conversion[40 41]. **c)** The minimum relative risk (RR) of interest is 2 for Objective 1 and 1.8 for Objective 2. **d)** The 24-month cumulative risk of clinically significant vasculopathy over study follow-up is 18.4% in the HIV positive group. This is based on the same study data cited in (a). **e)** For objectives 2a)-c)**,** the exposure prevalence for each risk factor is 20%. **f)** Statistical tests will have 80% power based on a 2-sided test with; α=0.05. Testing of hypotheses for the secondary outcome will be exploratory. However, we estimate 26 strokes (4 mimics), an unknown number of MIs and 80 deaths occurring during the study[7 42]. Taken together, c), d), e) mean that, for 80% power, we assume a 24-month cumulative vasculopathy risk of 9.2% in HIV negative participants, 18.4% in all HIV infected participants, 15.9% in HIV infected participants not exposed to the risk factors from objectives 2a)-c), 28.6% in the HIV infected participants exposed to these risk factors.

The reporting of this study will be prepared in accordance with the STROBE guidelines[43]. Summary and descriptive statistics will be tabulated for all primary and secondary outcome variables, as well as for exposure variables and potential confounding or mediating factors. Time plots for all outcome variables will be inspected. Quantitative data analysis will be conducted to assess the primary outcomes.

There will be 3 analysis time points: 1) after recruitment has finished and baseline data is available for all participants (baseline analysis), 2) once every participant has completed 6 months in the study (6-month analysis) and 3) at 36 months, when each participant has completed 24 months in the study (final analysis).

The baseline analysis will largely consist of descriptive statistics on participant characteristics and data recorded at baseline. Simple regression models will also be used to investigate relationships between exposure and outcome variables measured at baseline. Unadjusted analyses will consist of paired t-tests or Wilcoxon signed rank tests (depending whether the data are normally distributed or not) for continuously measured variables and Chi-Squared or Fisher's exact tests (depending on contingency table cell counts) for binary and categorical variables. Adjusted analyses will be conducted using generalised linear models (GLMs).

For peer review only - http://bmjopen.bmj.com/site/about/guidelines.xhtml

The 6-month analysis will be limited in score and serves 2 purposes: 1) characterise new onset vasculopathy in HIV-infected participants that have initiated ART treatment at baseline (vascular IRIS) and 2) define vasculopathy outcomes for the final analysis. The main analysis of the study data happens at the final analysis time point.

For objective 1 we will develop three regression models. Two GLMs will be developed to compare mean progression of arterial damage from baseline in HIV-infected ART patients and HIV-uninfected adults. These models will regress change from baseline in PWV, respectively cIMT, on HIV status. We will develop a third model to estimate the RR and population attributable fraction of new-onset arterial damage in HIV-infected patients compared to HIV-uninfected adults.

For objective 2a, a set of GLMs will be developed to compare mean progression of vasculopathy in HIV-infected ART patients with and without reactivated latent herpes viral infection. These models will regress change from baseline in PWV, respectively cIMT, on two log-transformed variables for antibody titres of CMV and VZV, respectively.

For objective 2b, we will again fit a set GLMs, with change from baseline in PWV as response variable, this time to investigate if, in HIV-infected ART patients, there is an association between progression of vasculopathy and immune activation and inflammation biomarkers (IL-6, ICAM, CD163). Specifically, for each marker, we will regress PWV on marker quantiles. After having built models for each marker, we will then develop comprehensive multiple regression models for PWV and cIMT with multiple independent markers as predictor variables.

For objective 2c, we will proceed as for objective 2a, but comparing HIV-infected ART patients with incomplete virological suppression or virological resurgence of HIV to those with suppressed HIV plasma viral load.

For peer review only - http://bmjopen.bmj.com/site/about/guidelines.xhtml

In addition to these analyses, given the repeated measurements for PWV, immune activation, inflammation markers, we will extent the GLMs for PWV to linear mixed models taking full account of the longitudinal nature of the data. Mixed models will also handle deviations from the visit schedule in a principled fashion and use all available data for drop-outs. In the case a log link function is required for PWV in the GLMs, we will fit marginalised models using GEE instead of the LMMs.

For secondary study objectives, we will use univariate methods to assess the frequency of clinical events within exposure strata. If there are sufficient numbers of clinical events we will develop Poisson or negative binomial regression models (depending on model fit) for each clinical event type to compare exposure-defined participants.

We will also use time-to-event models, specifically Cox proportional hazard models, to investigate associations between all-cause mortality and exposures.

As part of exploratory analyses, we will aim to identify risk groups that are potentially incompletely captured with the measured exposure variables. We will perform unsupervised group-based multi-trajectory modeling of multivariate longitudinal patient trajectories to confirm any associations we have found using more traditional approaches[44].

All efforts will be made to collect complete data on all study participants. However, there will inevitably be missing data due to drop-outs and a variety of other reasons. All primary analyses will be performed using multiple imputation. For sensitivity analyses, we will use all-available-cases (AAC), direct likelihood and fully Bayesian models and, for GEE models, weighted GEE. If the number of missingness patterns is sufficiently small, we will also use pattern mixture models which can be used under the general missing-not-at-random setting but make additional identification assumptions.

For peer review only - http://bmjopen.bmj.com/site/about/guidelines.xhtml

PATIENT PUBLIC INVOLVEMENT

The global burden of HIV associated CBD and CVD has tripled over the last two decades with the greatest impact in sub-Saharan Africa. CBD and CVD are a priority for patients in Malawi as HIV infection is endemic and the population are living for longer. Knowledge of this, informed our research question with the aim of understanding the mechanisms and thus direct targeted novel therapies to reduce this burden. Patients will be involved in the recruitment of the study, but not in the design. Patients and their advisors will be thanked for contributing to the study.

ETHICS AND DISSEMINATION

Written informed consent will be obtained from all study participants, either written or witnessed verbal consent with thumbprint if the participant is non-literate. Study data will be maintained in an encrypted and password protected database to which only study staff will have access. Study participants who develop a clinical event will be managed, using the hospital guidelines, by our study clinician alongside the hospital doctor. Clinical data will be anonymized using unique identifying code. Study data will be kept for 10 years and then destroyed with a record, as recommended by good clinical practice guidelines. This protocol was approved by the ethics committees at University of Malawi College of Medicine (Protocol P02/16/1874) and the Liverpool School of Tropical Medicine (Protocol 16-014). Results of the main trial and each of the secondary endpoints will be submitted for publication in a peer-reviewed journal.

DISCUSSION

African regions continue to bear the brunt of HIV infection, in 2013, an estimated 8.5 million adults were receiving ART[45]. As the landscape evolves, this population will live longer with stable HIV infection but likely remain at an increased risk of CBD/CVD compared to HIV-uninfected individuals of a similar age and sex. This study will be the first to determine the extent to which HIV reactivation of herpesvirus infection and inflammation contribute to CBD/CVD risk in an adult African population starting ART. The results of this work could

For peer review only - http://bmjopen.bmj.com/site/about/guidelines.xhtml

potentially open avenues for novel anti-inflammatory and anti-viral interventions for the primary prevention of CBD/CVD in HIV populations in Africa.

ACKNOWLEDGMENTS

The authors would like to thank Agbor Ako and Maria Davy from Research and Development, GlaxoSmithKline and the NCD Africa Open Lab of GlaxoSmithKline review committee for providing valuable advice for this protocol. The authors would like to thank BA, MC, LH, THC, JVO, NT for their contribution to the End Point Review Committee, RD and EJ for radiology training and quality control, EZS for providing an electrocardiogram platform and for his cardiology review, VK for input with the echocardiogram protocol, and TS, JM, KM, MN for their input in the advanced drafts of the manuscript. We also extend our gratitude to the INSIGHT network for sharing their clinical endpoint criteria. LB is supported by an NIHR Clinical Lecturer Fellowship. SP is supported by an MRC (UK) core funding MC_UU_12023/23.

AUTHORS' CONTRIBUTIONS

LB and IP developed the first draft. HM, NN, KJ, CK, LA, JKT, SP, MH, JVO, RH had major input for the revision of the second draft. JH is the project manager for RHICCA with oversight from LB, IP, and HM. MH contributed to the statistical methods. LB, JKT, JVO contributed to the clinical training. SP chaired the End point Review Committee.

FUNDING STATEMENT

Funding for this study was provided by the GlaxoSmithKline Africa Non-Communicable Disease Open Lab Grant (Project Number: 7964)

COMPETING INTERESTS

For peer review only - http://bmjopen.bmj.com/site/about/guidelines.xhtml

BMJ OPEN

SLP has academic grants from Sysmex Corporation, Gilead Sciences, and ViiV Healthcare. All other authors have no competing interest.

For peer review only

For peer review only - http://bmjopen.bmj.com/site/about/guidelines.xhtml

BMJ OPEN

REFERENCES

1. Roth GA, Johnson C, Abajobir A, et al. Global, Regional, and National Burden of Cardiovascular Diseases for 10 Causes, 1990 to 2015. *J Am Coll Cardiol* 2017;70(1):1-25. doi: 10.1016/j.jacc.2017.04.052
2. Organization WH. Global Update on HIV Treatment 2013: Results, Impact and Opportunities. WHO Report. Kuala Lumpure, Malaysia, 2013.
3. Macro NSONaI. Malawi Demographic and Health Survey 2010. Zomba,Malawi, and Calverton, Maryland, USA: NSO and ICF Macro, 2010.
4. Freiberg MS, Chang CC, Kuller LH, et al. HIV infection and the risk of acute myocardial infarction. *JAMA Intern Med* 2013;173(8):614-22. doi: 10.1001/jamainternmed.2013.3728

1659742 [pii] [published Online First: 2013/03/06]
5. Currier JS, Lundgren JD, Carr A, et al. Epidemiological evidence for cardiovascular disease in HIV-infected patients and relationship to highly active antiretroviral therapy. *Circulation* 2008;118(2):e29-35. doi: 10.1161/CIRCULATIONAHA.107.189624 [published Online First: 2008/06/21]
6. Butt AA, Chang CC, Kuller L, et al. Risk of heart failure with human immunodeficiency virus in the absence of prior diagnosis of coronary heart disease. *Archives of internal medicine* 2011;171(8):737-43. doi: 10.1001/archinternmed.2011.151 [published Online First: 2011/04/27]
7. Benjamin LA, Corbett EL, Connor MD, et al. HIV, antiretroviral treatment, hypertension, and stroke in Malawian adults: A case-control study. *Neurology* 2016;86(4):324-33. doi: 10.1212/WNL.0000000000002278
8. Allain TJ, Kinley L, Tsidya B, et al. The spectrum of heart disease in adults in Malawi: A review of the literature with reference to the importance of echocardiography as a diagnostic modality. *Malawi Med J* 2016;28(2):61-65. [published Online First: 2016/11/30]
9. Soliman EZ, Juma H. Cardiac disease patterns in northern Malawi: epidemiologic transition perspective. *J Epidemiol* 2008;18(5):204-8. [published Online First: 2008/08/30]
10. Syed FF, Sani MU. Recent advances in HIV-associated cardiovascular diseases in Africa. *Heart* 2013;99(16):1146-53. doi: 10.1136/heartjnl-2012-303177 [published Online First: 2013/05/18]
11. Soliman EZ, Sharma S, Arasteh K, et al. Baseline cardiovascular risk in the INSIGHT Strategic Timing of AntiRetroviral Treatment (START) trial. *HIV Med* 2015;16 Suppl 1:46-54. doi: 10.1111/hiv.12233 [published Online First: 2015/02/26]
12. Benjamin LA, Allain TJ, Mzinganjira H, et al. The Role of Human Immunodeficiency Virus-Associated Vasculopathy in the Etiology of Stroke. *J Infect Dis* 2017;216(5):545-53. doi: 10.1093/infdis/jix340 [published Online First: 2017/09/22]
13. Benjamin LA, Bryer A, Lucas S, et al. Arterial ischemic stroke in HIV: Defining and classifying etiology for research studies. *Neurol Neuroimmunol Neuroinflamm* 2016;3(4):e254. doi: 10.1212/NXI.0000000000000254
14. Nakanjako D, Kiragga A, Ibrahim F, et al. Sub-optimal CD4 reconstitution despite viral suppression in an urban cohort on antiretroviral therapy (ART) in sub-Saharan Africa: frequency and clinical significance. *AIDS Res Ther* 2008;5:23. doi: 10.1186/1742-6405-5-23 [published Online First: 2008/10/30]
15. Pawelec G, Derhovanessian E. Role of CMV in immune senescence. *Virus Res* 2011;157(2):175-9. doi: 10.1016/j.virusres.2010.09.010 [published Online First: 2010/09/28]
16. Appay V, Sauce D. Immune activation and inflammation in HIV-1 infection: causes and consequences. *J Pathol* 2008;214(2):231-41. doi: 10.1002/path.2276 [published Online First: 2007/12/29]
17. Shaw AC, Joshi S, Greenwood H, et al. Aging of the innate immune system. *Curr Opin Immunol* 2010;22(4):507-13. doi: 10.1016/j.coi.2010.05.003 [published Online First: 2010/07/30]

For peer review only - http://bmjopen.bmj.com/site/about/guidelines.xhtml

18. Hearps AC, Angelovich TA, Jaworowski A, et al. HIV infection and aging of the innate immune system. *Sex Health* 2011;8(4):453-64. doi: 10.1071/SH11028 [published Online First: 2011/12/01]
19. Kovacs EJ, Palmer JL, Fortin CF, et al. Aging and innate immunity in the mouse: impact of intrinsic and extrinsic factors. *Trends Immunol* 2009;30(7):319-24. doi: 10.1016/j.it.2009.03.012 [published Online First: 2009/06/23]
20. Hansson GK. Inflammation, atherosclerosis, and coronary artery disease. *The New England journal of medicine* 2005;352(16):1685-95. doi: 10.1056/NEJMra043430 [published Online First: 2005/04/22]
21. Boekholdt SM, Stroes ES. The interleukin-6 pathway and atherosclerosis. *Lancet* 2012;379(9822):1176-8. doi: 10.1016/S0140-6736(12)60361-4 [published Online First: 2012/03/17]
22. Duprez DA, Neuhaus J, Kuller LH, et al. Inflammation, coagulation and cardiovascular disease in HIV-infected individuals. *PloS one* 2012;7(9):e44454. doi: 10.1371/journal.pone.0044454 [published Online First: 2012/09/13]
23. Lundgren JD, Babiker AG, Gordin F, et al. Initiation of Antiretroviral Therapy in Early Asymptomatic HIV Infection. *N Engl J Med* 2015;373(9):795-807. doi: 10.1056/NEJMoa1506816 [published Online First: 2015/07/21]
24. Eggena MP, Barugahare B, Okello M, et al. T cell activation in HIV-seropositive Ugandans: differential associations with viral load, CD4+ T cell depletion, and coinfection. *The Journal of infectious diseases* 2005;191(5):694-701. doi: 10.1086/427516 [published Online First: 2005/02/03]
25. Mussini CL, P.; Cozzi-Lepri,A.; Lapadula,G.; Marchetti,G.; Nicastri,E.; Cingolani,A.; Lichtner,M.;Antinori,A.; Gori,A.; Monforte, A. CD4/CD8 ratio normalisation and non-AIDS-related events in individuals with HIV who achieve viral load suppression with antiretroviral therapy: an observational cohort study. *Lancet HIV* 2015;2:e98–e106.
26. Sereti I, Krebs SJ, Phanuphak N, et al. Persistent, Albeit Reduced, Chronic Inflammation in Persons Starting Antiretroviral Therapy in Acute HIV Infection. *Clin Infect Dis* 2017;64(2):124-31. doi: 10.1093/cid/ciw683 [published Online First: 2016/10/16]
27. Brunner S, Herndler-Brandstetter D, Weinberger B, et al. Persistent viral infections and immune aging. *Ageing Res Rev* 2011;10(3):362-9. doi: 10.1016/j.arr.2010.08.003 [published Online First: 2010/08/24]
28. Moss P. The emerging role of cytomegalovirus in driving immune senescence: a novel therapeutic opportunity for improving health in the elderly. *Curr Opin Immunol* 2010;22(4):529-34. doi: 10.1016/j.coi.2010.07.001 [published Online First: 2010/08/06]
29. Appay V, Rowland-Jones SL. Premature ageing of the immune system: the cause of AIDS? *Trends Immunol* 2002;23(12):580-5. [published Online First: 2002/12/05]
30. Schaftenaar E, Verjans GM, Getu S, et al. High seroprevalence of human herpesviruses in HIV-infected individuals attending primary healthcare facilities in rural South Africa. *PloS one* 2014;9(6):e99243. doi: 10.1371/journal.pone.0099243 [published Online First: 2014/06/11]
31. Hunt PW, Martin JN, Sinclair E, et al. Valganciclovir reduces T cell activation in HIV-infected individuals with incomplete CD4+ T cell recovery on antiretroviral therapy. *J Infect Dis* 2011;203(10):1474-83. doi: 10.1093/infdis/jir060 [published Online First: 2011/04/20]
32. Corbett EL. Intensified HIV/TB prevention linking home-based HIV testing, including the option of selftesting, with HIV care. ISRCTN02004005. London: ISRCTN, 2012.
33. Roth GA, Huffman MD, Moran AE, et al. Global and regional patterns in cardiovascular mortality from 1990 to 2013. *Circulation* 2015;132(17):1667-78. doi: 10.1161/CIRCULATIONAHA.114.008720
34. Maple PA, Breuer J, Quinlivan M, et al. Comparison of a commercial Varicella Zoster glycoprotein IgG enzyme immunoassay with a reference time resolved fluorescence immunoassay (VZV TRFIA) for measuring VZV IgG in sera from pregnant women, sera sent for confirmatory

For peer review only - http://bmjopen.bmj.com/site/about/guidelines.xhtml

testing and pre and post vOka vaccination sera from healthcare workers. *J Clin Virol* 2012;53(3):201-7. doi: 10.1016/j.jcv.2011.12.010 [published Online First: 2012/01/21]
35. Carlier P, Harika N, Bailly R, et al. Laboratory evaluation of the new Access (R) cytomegalovirus immunoglobulin IgM and IgG assays. *J Clin Virol* 2010;49(3):192-7. doi: 10.1016/j.jcv.2010.07.024 [published Online First: 2010/08/31]
36. Laurent S, Cockcroft J, Van Bortel L, et al. Expert consensus document on arterial stiffness: methodological issues and clinical applications. *Eur Heart J* 2006;27(21):2588-605. doi: 10.1093/eurheartj/ehl254 [published Online First: 2006/09/27]
37. Touboul PJ, Hennerici MG, Meairs S, et al. Mannheim carotid intima-media thickness and plaque consensus (2004-2006-2011). An update on behalf of the advisory board of the 3rd, 4th and 5th watching the risk symposia, at the 13th, 15th and 20th European Stroke Conferences, Mannheim, Germany, 2004, Brussels, Belgium, 2006, and Hamburg, Germany, 2011. *Cerebrovasc Dis* 2012;34(4):290-6. doi: 10.1159/000343145

000343145 [pii] [published Online First: 2012/11/07]
38. Serina P, Riley I, Stewart A, et al. Improving performance of the Tariff Method for assigning causes of death to verbal autopsies. *BMC Med* 2015;13:291. doi: 10.1186/s12916-015-0527-9 [published Online First: 2015/12/09]
39. Organization WH. Consolidated ARV guidelines 2013 [Available from: http://www.who.int/hiv/pub/guidelines/arv2013/art/artmonitoring/en/index4.html accessed 15 Oct 2015.
40. Misiri HE, Edriss A, Aalen OO, et al. Estimation of HIV incidence in Malawi from cross-sectional population-based sero-prevalence data. *Journal of the International AIDS Society* 2012;15(1):14. doi: 10.1186/1758-2652-15-14 [published Online First: 2012/03/16]
41. MacPherson P, Houben RM, Glynn JR, et al. Pre-treatment loss to follow-up in tuberculosis patients in low- and lower-middle-income countries and high-burden countries: a systematic review and meta-analysis. *Bull World Health Organ* 2014;92(2):126-38. doi: 10.2471/BLT.13.124800 [published Online First: 2014/03/14]
42. Walker R, Whiting D, Unwin N, et al. Stroke incidence in rural and urban Tanzania: a prospective, community-based study. *Lancet Neurol* 2010;9(8):786-92. doi: S1474-4422(10)70144-7 [pii]

10.1016/S1474-4422(10)70144-7 [published Online First: 2010/07/09]
43. von Elm E, Altman DG, Egger M, et al. The Strengthening the Reporting of Observational Studies in Epidemiology (STROBE) Statement: guidelines for reporting observational studies. *International journal of surgery* 2014;12(12):1495-9. doi: 10.1016/j.ijsu.2014.07.013 [published Online First: 2014/07/22]
44. Nagin DS, Jones BL, Passos VL, et al. Group-based multi-trajectory modeling. *Stat Methods Med Res* 2018;27(7):2015-23. doi: 10.1177/0962280216673085 [published Online First: 2018/05/31]
45. Organization WH. Global Update on the Health Sector Response to HIV 2014. Geneva: World Health Organization, 2014.

For peer review only

For peer review only - http://bmjopen.bmj.com/site/about/guidelines.xhtml

BMJ OPEN

Figure 1. Hypothetical pathway of the interplay between chronic viruses, immune activation, systemic inflammation, endothelial activation, and vasculopathy.

Figure 2. Outline of study design for a 36-month cohort study

For peer review only

Table 1. Laboratory tests and clinical procedures in ART patients and HIV-uninfected adults

	Study Time Points						
	Baseline	6 months	12 months	18 months	24 months	30 months	36 months
**Clinical Procedures**							
PWV	X	X	X	X	X	X	X
CIMT	X				X		
ABPI	X	X	X	X	X	X	X
Cardiac Echo *(participant sub-set)*	X				X		
ECG *(participant sub-set)*	X				X		
**Cardiometabolic markers**							
Creatinine	X				X		
Full Blood Count	X	X					
Cholesterol (LDL, HDL, Triglycerides)	X				X		
Serum glucose/HBA1C	X				X		
**HIV Infection and Progression**							
HIV viral load *(HIV patients)*	X	X	X				
CD4 count *(HIV patients)*	X	X	X				
HIV rapid test *(controls)*	X		X		X		X
**Immune dysregulation**							
Soluble markers of systemic inflammation	X	X	X				
Soluble markers of endothelial activation	X	X	X				
CD8 and CD4 T-cell activation and senescence *(participant subset)*	X	X	X		X		X
Monocyte/ Macrophage activation and senescence *(participant subset)*	X	X	X		X		X
Herpesviruses infection							
CMV IgG	X	X					
VZV IgG	X	X					

For peer review only

Table 2: Case definitions of primary and secondary endpoints for the study

	Type	Definitions
**Primary Endpoint**	Carotid intimal medial thickness (CIMT)	The occurrence of new-onset vasculopathy [CIMT – a measure of atherosclerosis]: CIMT >0.9 mm or >75th percentile of age/sex references values or presence of plaque on the carotid scan<br><br>Progression: total change in CIMT at 24 months from baseline
	Pulse wave velocity (PWV)	Occurrence of new onset vasculopathy [PWV – a measure of arterial stiffness]: PWV >12[m/s]<br><br>Progression: total change in PWV at 24 months from baseline
**Secondary endpoint**	Stroke	**Confirmed (1+2) or 3 or 4 or 5:**<br>1. Acute onset with a clinically compatible course, including unequivocal objective findings of a localizing neurologic deficit<br>2. CT or MRI compatible with a diagnosis of stroke and current neurologic signs and symptoms<br>3. Stroke diagnosed as the cause of death at autopsy<br>4. Compatible clinical history and positive lumbar puncture compatible with subarachnoid hemorrhage<br>5. Death certificate or death note from medical record listing stroke as the cause of death
	Myocardial Infarction [MI]	**Confirmed: One of the following 5 criteria (adapted from 2007 Universal Definition of Myocardial Infarction): [1 + (2 or 3 or 6)] or 4 or 5**<br><br>1. Rise and/or fall of cardiac biomarkers (preferably troponin), with at least one value above the 99th percentile of upper reference limit (URL);<br>2. The occurrence of a compatible clinical syndrome, including symptoms consistent with myocardial ischemia;<br>3. ECG changes indicative of new ischemia (new ST-changes or new left bundle branch block [LBBB]), or development of pathological Q waves on the ECG;<br>4. Sudden unexpected cardiac death involving cardiac arrest before biomarkers are obtained or before a time when biomarkers appear, along with (a) new ST-changes or new LBB, or (b) evidence of fresh thrombus on coronary angiography or at autopsy<br>5. Pathologic findings of acute myocardial infarction (including

For peer review only - http://bmjopen.bmj.com/site/about/guidelines.xhtml

[redacted]		acute MI demonstrated as the cause of death on autopsy)<br>6. Development of a) evolving new Q waves, or b) evolving ST elevation, preferably based on at least 2 ECGs taken during the same hospital admission
	Coronary artery disease requiring drug treatment	**Confirmed (1 or 2) + 3:**<br><br>1. Evidence of myocardial ischemia based on either diagnostic imaging (such as a stress echocardiogram or thallium scan) or diagnostic changes on an electrocardiogram (such as during stress testing or an episode of chest pain)<br>2. Evidence of coronary artery disease based on coronary angiography or other diagnostic imaging<br>3. Use of medication given to treat or prevent angina (e.g., nitrates, beta blockers, calcium channel blockers)
	Peripheral vascular disease [PVD]	**Confirmed (1+2) or (1+3):**<br>1. Compatible clinical signs and symptoms<br>2. Positive results on diagnostic imaging studies (e.g., Doppler ultrasound, contrast arteriography, MRI arteriography); 3. Ankle Brachial Pressure Index < 0.90 in non-diabetics
	Vascular Immune reconstitution syndrome (IRIS)	A new onset vasculopathy within 6 months of starting ART
	All-cause death and vascular-related deaths	Death (of any or vascular cause) that occurs after recruitment into the study

For peer review only

BMJ OPEN

For peer review only

For peer review only - http://bmjopen.bmj.com/site/about/guidelines.xhtml

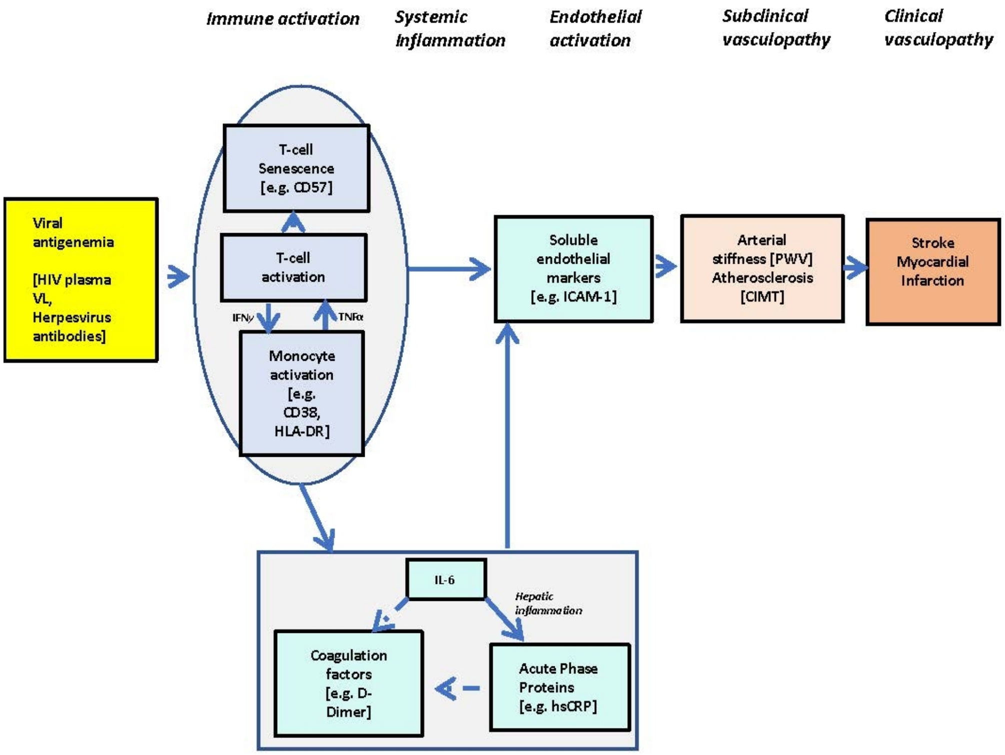

Figure 1

90x90mm (300 x 300 DPI)

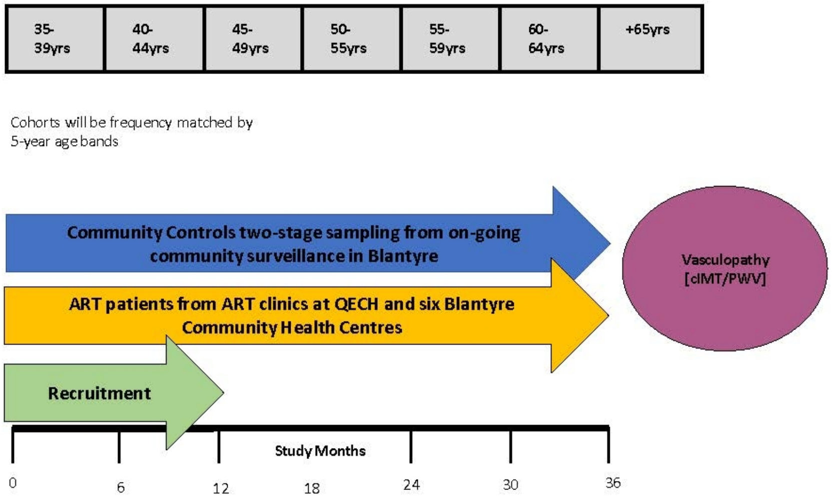

Figure 2

90x90mm (300 x 300 DPI)

RHICCA – Clinical endpoint diagnostic criteria vs 3.5 Apr 2018

RHICCA
Reactivation of herpesviruses and inflammation as cardiovascular and cerebrovascular risk factors in ART initiators

	CONFIRMED	PROBABLE
**INFECTIONS**		
Aspergillosis, invasive pulmonary	**Confirmed: 1 + 2 + 3:**<br>1. Compatible clinical course **(Appendix 13)**,<br>2. CXR abnormality compatible with aspergillosis;<br>3. invasive mycelia consistent with Aspergillus on lung biopsy or positive culture of lung tissue or **positive culture of sputum** collected by any method	**Probable: 1 + 2 + 3:**<br>1. Compatible clinical course **(Appendix 13)**,<br>2. CXR abnormality compatible with aspergillosis;<br>3. invasive mycelia consistent with Aspergillus on tissue biopsy or positive culture at a normally sterile site (e.g., blood) apart from the lungs.
Aspergillosis, other invasive	**Confirmed: 1 + 2 + 3:**<br>1.compatible clinical course **(Appendix 11)**,<br>2. invasive mycelia consistent with Aspergillus on tissue biopsy or clinical evidence of infection,<br>3. positive culture from the affected tissue	**Probable: 1 + 2:**<br>1.clinical evidence of invasive infection **(Appendix 11)**, 2.invasive mycelia consistent with Aspergillus on tissue biopsy or positive culture at a normally sterile site (e.g., blood) apart from the involved tissue
Bartonellosis	**Confirmed 1+ 2:**<br>1.Clinical or histologic evidence of bacillary angiomatosis or bacillary peliosis,<br>2. a positive culture or PCR for *B. quintana* or *B. henselae*	**Probable 1 + 2:**<br>1.Clinical evidence of bacillary angiomatosis or bacillary peliosis **(Appendix 12**),<br>2. positive silver stain for bacilli from a skin lesion or an affected organ
Candidiasis,oral	**Confirmed:1 + 2 + 3:**<br>1. Macroscopic appearance on examination of the mouth<br>2. microscopic evidence of yeasts or pseudo hyphae<br>3. no evidence of oesophageal involvement -	**Probable: 1 + 2 + 3:**<br>1. a clinical diagnosis of oral candidiasis and/or microscopic evidence of yeasts or pseudo hyphae<br>2. clinical response to treatment<br>3. no evidence of oesophageal involvement
Candidiasis of bronchi, trachea, or lungs	**Confirmed:1 + 2:**<br><br>Macroscopic appearance at bronchoscopy or autopsy<br>microscopic evidence of yeasts or pseudo hyphae	**None**
Candidiasis, esophageal	**Confirmed: 1 + 2:**<br>1. Macroscopic appearance at esophagoscopy or autopsy.<br>2. microscopic evidence of yeasts or pseudo hyphae	**Probable: 1 + 2 + 3:**<br>1. Recent onset of retrosternal pain or difficulty on swallowing.<br>2. a clinical diagnosis of oral candidiasis, endoscopic visualization of candidiasis and/or microscopic evidence of yeasts or pseudo hyphae from oropharyngeal mucosa<br>3. clinical response to treatment

For peer review only

RHICCA – Clinical endpoint diagnostic criteria vs 3.5 Apr 2018

RHICCA
Reactivation of herpesviruses and inflammation as cardiovascular and cerebrovascular risk factors in ART initiators

	CONFIRMED	PROBABLE
**INFECTIONS (CONTINUED)**		
Cryptococcosis, extrapulmonary (not meningitis)	**Confirmed: 1 or 2 or 3:**<br><br>From tissue other than lung or hilum:<br>1. microscopic demonstration of narrow based budding yeast<br>2. positive culture,<br>3. antigen detention	**None**
Cryptococcosis meningitis	**Confirmed: 1 or 2 or 3 or 4:**<br>1. Brain histopathology microscopic demonstration of narrow based budding yeast<br>2. CSF evidence of India ink test<br>3. CSF evidence of positive culture<br>4. CSF evidence of positive antigen detection	**None**
Cryptosporidiosis	**Confirmed: 1 + 2**<br>1. Diarrhea for > 1 month<br>2. positive microscopy	**None**
CMV retinitis	Autopsy demonstration	**Probable 1 + 2:**<br>1. Typical appearance on fundoscopy of discrete patches of retinal whitening, spreading along blood vessels.<br>2. Associated vasculitis, hemorrhage and necrosis, confirmed by ophthalmologist

	CONFIRMED	PROBABLE
**INFECTIONS (CONTINUED)**		
HZV single dermatome	**Confirmed 1+2:**<br>1.multiple ulcerated lesions affecting at least 1 dermatome, and/or 1 or more contiguous dematomes;<br><br>2. positive culture, PCR, or antigen assay from affected tissue	**Probable 1+ 2:**<br>1.multiple typical ulcerated lesions affecting at Least 1 dermatome, and/or 1 or more contiguous dermatomes;<br><br>2. response to an antiviral active against HZV unless resistance is demonstrated
HZV, disseminated	**Confirmed 1+2:**<br>1. multiple ulcerated lesions affecting at least 2 non-contiguous dermatomes, or with generalized cutaneous dissemination HZV involvement of the lung, liver, brain, or other internal organs<br>2. positive culture, PCR, or antigen assay from affected tissue	**Probable 1+2:**<br>1. multiple typical ulcerated lesions affecting at least 2 non-contiguous dermatomes, or with generalized cutaneous dissemination<br>2. response to an antiviral active against HZV unless resistance is demonstrated
HSV mucocutaneous ulceration	**Confirmed 1 +2:**<br>1.Ulceration for > 1 Month<br>2. Histology, culture, PCR, or detection of antigen from affected tissue	**Probable 1 + 2:**<br>1. Typical HSV ulceration for > 1 month,<br>2. response to an antiviral active against HZV unless resistance is demonstrated
Histoplasmosis, disseminated or extrapulmonary	**Confirmed 1+2:**<br>1.Compatible symptoms,<br>2. histology or culture or elevated blood or urine antigen levels	None
Isosporiasis	**Confirmed 1 + 2:**<br>1.Diarrhea for > 1 month<br>2.microscopic identification of *Isospora belli*	None

For peer review only

RHICCA – Clinical endpoint diagnostic criteria vs 3.5 Apr 2018

RHICCA

Reactivation of herpesviruses and inflammation as cardiovascular and cerebrovascular risk factors in ART initiators

Microsporidiosis	**Confirmed 1 + 2:**<br>1.Diarrhea for > 1 month<br>2.Microscopic identification of Microsporidia	None
MAC and other mycobacterial disseminated diseas	**Confirmed 1 + 2:**<br>1. Fever, fatigue, anemia or diarrhea<br>2.positive culture from blood, body fluids or tissue other than pulmonary, hilar or stool	**Probable 1+2+3:**<br>1. Fever, fatigue, anemia or diarrhea<br>2. **AFB** or positive direct MAC PCR in blood, body fluids or tissue other than pulmonary, hilar or stool<br>3. no concurrent non-pulmonary TB

-

	CONFIRMED	PROBABLE	POSSIBLE
M. tuberculosis disease, pulmonary	**Confirmed 1+2:**<br>1.Compatible symptoms of fever, dyspnea, cough, weight loss or fatigue<br>2. culture or PCR from **sputum** or bronchial lavage or lung tissue	**Probable 1+2+3+4:**<br>1.Symptoms of fever, dyspnea, cough, weight loss or fatigue<br>2.abnormal chest X-ray,<br>3. AFBs seen in sputum or lavage or lung tissue but not grown in culture,<br>4.responds to treatment	**Possible 1+2+3+4:**<br>1.Symptoms of fever, dyspnea , cough, weight loss or fatigue<br>2.abnormal chest X-ray compatible with pulmonary TB (such as upper lobe cavitation, pleural exudate)<br>3. No other etiology for pulmonary symptoms and signs identified,<br>4.Responds to anti tuberculosis treatment
M. tuberculosis disease, Extrapulmonary (not meningitis)	**Confirmed 1+2:**<br>1. Compatible symptoms<br>2. culture or PCR or MTB Xpert from blood or affected tissue (i.e. pericardial, ascites, and lymph glands)	**Probable 1+2+3**:<br>1.Compatible symptoms<br>2. AFBs seen from affected tissue or blood<br>3.concurrent diagnosis of pulmonary TB or responds to treatment	**Possible 1+2+3:**<br>1.Compatible symptoms<br>2. No other etiology for symptoms and signs identified<br>3.concurrent diagnosis of pulmonary TB or responds to treatment
M. tuberculosis disease, meningitis	**Confirmed 1+2:**<br>**1.** Clinical symptoms of meningism **(Appendix 7)**<br>**2.** Tissue/CSF culture, or PCR, or AFB or MTB Xpert	**Probable 1+ a score ≥12 (Appendix: Table 2):**<br>1. Clinical symptoms of meningism **(Appendix 7)**<br>2. A score ≥12, based on clinical, CSF, cerebral brain imaging criteria or evidence of TB elsewhere	
Nocardiosis	**Confirmed 1+2:**<br>1. Clinical evidence of invasive Infection **(Appendix 8)**<br>2. a positive culture from the affected tissue or blood	**Probable 1+2:**<br>1. Clinical evidence of invasive Infection **(Appendix 8)**<br>2. microscopic evidence of bronchial weakly acid fast organisms from the affected tissue	

For peer review only

For peer review only - http://bmjopen.bmj.com/site/about/guidelines.xhtml

RHICCA – Clinical endpoint diagnostic criteria vs 3.5 Apr 2018

RHICCA
Reactivation of herpesviruses and inflammation as cardiovascular and cerebrovascular risk factors in ART initiators

Pneumocystis jirovecii pulmonary	**Confirmed 1+2:**<br>1. compatible clinical syndrome **(Appendix 9)**<br>2. microscopic or histological demonstration of *P. jirovecii* cysts in a pulmonary specimen	Probable 1+2+3+4+5<br>1. dyspnea or cough, or fever progressive over > 1 week<br>2. **diffuse chest x-ray abnormality** or, if on inhalational pentamidine, diffuse upper lung field abnormality<br>3. evidence of hypoxia<br>4. not suggestive of bacterial pneumonia (i.e., not purulent sputum or hemoptysis, no bacterial pathogen identified in blood or bronchial wash)<br>5. response to PcJ treatment
Pneumocystis jirovecii, extrapulmonary	**Confirmed 1+2:**<br>1. compatible clinical syndrome<br>2. microscopic or histological demonstration of *P. jirovecii* cysts in a tissue other than pulmonary specimen	None

For peer review only

RHICCA – Clinical endpoint diagnostic criteria vs 3.5 Apr 2018

RHICCA

Reactivation of herpesviruses and inflammation as cardiovascular and cerebrovascular risk factors in ART initiators

	CONFIRMED	PROBABLE
**INFECTIONS (CONTINUED)**		
Pneumonia, **SINGLE EPISODE (isolated)**<br>bacterial, excludes: (a) post-obstructive pneumonias, including in those with intrapulmonary/bronchial lesions, (b) aspiration pneumonia, including in those with decreased consciousness levels, (c) nosocomial ventilator-associated pneumonias	**Confirmed 1+2+3:**<br>pneumonia episodes must occur after enrollment;<br>1. Signs and symptoms suggestive of bacterial pneumonia (**appendix 10**)<br>2. Focal CXR abnormality compatible with bacterial pneumonia,<br>3. identification of a bacterial pathogen by positive blood culture, positive bronchoscopic sputum culture, detection of Legionella or pneumococcal antigen in urine or blood or diagnostic serologic findings	**Probable 1+2:**<br>pneumonia episodes must occur after enrollment;<br>1.Signs and symptoms suggestive of bacterial pneumonia (**Appendix 10**)<br>2. Focal CXR abnormality compatible with Bacterial pneumonia
Pneumonia, **recurrent**<br>bacterial, excludes: (a) post-obstructive pneumonias, including in those with intrapulmonary/bronchial lesions, (b) aspiration pneumonia, including in those with decreased consciousness levels, (c) nosocomial ventilator-associated pneumonias	**Confirmed 1+2+3+4+5**<br>Second pneumonia episodes must occur after enrollment and after an isolated pneumonia, within 365 days of the first episode and with strong clinical evidence that the first episode was cured;<br>1. Signs and symptoms of second event suggestive of bacterial pneumonia (**Appendix 10**)<br>2. Focal CXR abnormality compatible with bacterial pneumonia,<br>3. identification of a bacterial pathogen by positive blood culture, positive bronchoscopic sputum culture, detection of Legionella or pneumococcal antigen in urine or blood or diagnostic serologic findings<br>4. the second pneumonia had onset of symptoms < 365 days after the first episode<br>5. there is strong evidence that the first episode was cured such as an intervening clear CXR or absence of symptoms after > 1 month off antibacterial effective against pathogens commonly producing pneumonia	**Probable 1+2+3+4:**<br>Second pneumonia episodes must occur after enrollment and after an isolated pneumonia, within 365 days of the first episode and with strong clinical evidence that the first episode was cured;<br>1. Signs and symptoms suggestive of bacterial pneumonia (**Appendix 10**)<br>2. focal CXR abnormality compatible with bacterial pneumonia<br>3. the second pneumonia had onset of symptoms < 365 days after the first episode<br>4. there is strong evidence that the first episode was cured such as an intervening clear CXR or absence of symptoms after > 1 month off antibacterials effective against pathogens commonly producing pneumonia
PML (progressive multifocal leukoencephalopathy)	**Confirmed 1 or 2:**<br>1. positive histology,<br>2. compatible clinical (**Appendix 11**) and radiologic course and positive CSF PCR for JK virus	**Probable 1+2+3:**<br>1. Consistent symptoms (**Appendix 11)**,<br>2. brain image consistent with PML,<br>3. no response to toxo treatment or toxoplasma [illegible]
Salmonella blood stream infection or bacteraemia, isolated	**Confirmed 1:**<br>A septic episode must occur after enrollment;<br>1. Positive blood or tissue culture	None
Salmonella blood stream infection or bacteraemia, recurrent	**Confirmed 1:**<br>A second septic episode must occur after enrollment and after an isolated episode;<br>1. Has met the criteria of isolated Salmonella septicemia<br>2. Positive blood or tissue culture on the second episode<br>3. the second septicemia had onset of symptoms < 365 days after the first episode<br>4. the second septicemia must be due to a different Salmonella serotype or there must be strong evidence that the first episode was cured such as a negative blood culture off effective antibacterials for > 1 week or absence of symptoms off antibacterials for > 1 month	None

For peer review only

RHICCA – Clinical endpoint diagnostic criteria vs 3.5 Apr 2018

RHICCA
Reactivation of herpesviruses and inflammation as cardiovascular and cerebrovascular risk factors in ART initiators

Toxoplasmosis of brain	**Confirmed 1+2+3:**<br>1. Compatible clinical findings **(Appendix 12)**<br>2. Compatible radiological findings<br>3. Detection of T gondii in the **CSF** or brain tissue (i.e. microscopy or PCR)	**Probable 1+2+3:**<br>1. Symptoms of focal intracranial abnormality or decreased consciousness<br>2. brain image consistent with lesion(s) enhanced by contrast<br>3. positive toxoplasma serology or responds to treatment clinically or by scan

	CONFIRMED	PROBABLE
**NEOPLASMS**		
Cervical carcinoma, invasive	**Confirmed 1:**<br>1. Histology (NOT carcinoma-in-situ)	**None**
Kaposi sarcoma, (mucocutaneous or visceral)	**Confirmed 1:**<br>1. Histology	1. Highly typical appearance<br>2. persistence for > 1 month
Lymphoma, primary, of brain	**Confirmed 1:**<br>1. Histology of brain tissue	**Probable 1+2+3:**<br>1. Symptoms consistent with lymphoma<br>2. at least one CNS lesion with mass effect<br>3. lack of clinical or radiographic response at least 2 weeks of treatment for toxoplasmosis
Lymphoma, Hodgkin's	1. Histology	None
Lymphoma, non-Hodgkin's, all cell types	**Confirmed 1:**<br>1. Histology	None
**NEUROLOGICAL**		
HIV-related encephalopathy (including AIDS Dementia Complex)	None	**Probable 1+2+3+4:**<br>1. Cognitive or motor dysfunction interfering with usual activity, progressive over weeks or months<br>2. no other condition to explain the findings<br>3. brain image obtained and suggests no other causes<br>4. grade 2 or worse impairment in at least 2 domains by NARS (**appendix – table 1**) excluding abnormal domains at trial entry. (For persons with abnormal domains at entry worsening by at least two grades meets criteria.)

For peer review only - http://bmjopen.bmj.com/site/about/guidelines.xhtml

RHICCA – Clinical endpoint diagnostic criteria vs 3.5 Apr 2018

RHICCA
Reactivation of herpesviruses and inflammation as cardiovascular and cerebrovascular risk factors in ART initiators

CARDIOVASCULAR DISEASES		
Acute Myocardial Infarction	**Confirmed: One of the following 5 criteria (adapted from 2007 Universal Definition of Myocardial Infarction): [1 + (2 or 3 or 6)] or 4 or 5**<br>1. Rise and/or fall of cardiac biomarkers (preferably troponin), with at least one value above 99th percentile of upper reference limit (URL);<br>2. Occurrence of a compatible clinical syndrome, including symptoms (such as chest pain **– see Appendix 1**) consistent with myocardial ischemia;<br>3. ECG changes indicative of new ischemia (new ST-changes or new left bundle branch block [LBBB]), or development of pathological Q waves on the ECG;<br>4. Sudden unexpected cardiac death involving cardiac arrest before biomarkers are obtained or before a time when biomarkers appear, along with (a) new ST-changes or new LBB, or (b) evidence of fresh thrombus on coronary angiography or at autopsy<br>5. Pathologic findings of acute myocardial infarction (including acute MI demonstrated as the cause of death on autopsy)<br>6. Development of a) evolving new Q waves, or b) evolving ST elevation, preferably based on at least 2 ECGs taken during the same hospital admission	**Probable 1 and 2:**<br>1. Occurrence of a compatible clinical syndrome (**Appendix 1**), including symptoms (such as chest pain) consistent with myocardial ischemia)<br>2. Development of a) evolving new Q waves, or b) evolving ST elevation, preferably based on at least; ECGs taken during the same hospital admission.
Peripheral vascular disease	**Confirmed (1+2) or (1+3):**<br>1. Compatible clinical signs and symptoms (**see Appendix 3**)<br>2. Positive results on diagnostic imaging studies (e.g., Doppler ultrasound, contrast arteriography, MRI arteriography); 3. Ankle Brachial Pressure Index < 0.90 in non-diabetics	**Probable 1:**<br>1. Compatible clinical signs and symptoms (**see Appendix 3**)
Stroke	**Confirmed (1+2) or 3 or 4 or 5:**<br>1. Acute onset with a clinically compatible course, including unequivocal objective findings of a localizing neurologic deficit **(appendix 4)**;<br>2. CT or MRI compatible with diagnosis of stroke and current neurologic signs and symptoms<br>3. Stroke diagnosed as cause of death at autopsy<br>4. Compatible clinical history and positive lumbar puncture compatible with subarachnoid hemorrhage<br>5. Death certificate or death note from medical record listing stroke as cause of death	**Probable (1+2) or (1+3):**<br>1. Acute onset with a clinically compatible course, including unequivocal objective findings of a localizing neurologic deficit **(appendix 4)**;<br>2. Positive lumbar puncture compatible with subarachnoid hemorrhage<br>3. Death certificate or death note from medical record listing stroke as cause of death
Congestive heart failure	**Confirmed (1+2) or (1+3) or (1+4):**<br>1. Clinical signs and symptoms compatible with left or right sided heart failure [**Appendix 5]** without an alternative explanation<br>2. Hemodynamic measurements, radionucleotide ventriculography, echocardiogram, cardiac catheterization, or multiple gated acquisition scan showing a decreased ejection fraction of < 45%<br>3. Echocardiogram, cardiac catheterization or other studies showing evidence of increased left atrial pressure or right heart failure;<br>4. Elevated levels of Brain Natriuretic Peptide (BNP) or NT-proBNP	**Probable 1+2+3:**<br>1. Clinical signs and symptoms compatible with left or right sided heart failure [**Appendix 5]** without an alternative explanation<br>2. Chest x-ray or other imaging study showing evidence of congestive heart failure, including cardiac enlargement;<br>3. Documentation of treatment for congestive heart failure

For peer review only - http://bmjopen.bmj.com/site/about/guidelines.xhtml

RHICCA – Clinical endpoint diagnostic criteria vs 3.5 Apr 2018

RHICCA
Reactivation of herpesviruses and inflammation as cardiovascular and cerebrovascular risk factors in ART initiators

Coronary artery disease requiring drug treatment	**Confirmed (1 or 2) + 3:**<br><br>1. Evidence of myocardial ischemia based on either diagnostic imaging (such as a stress echocardiogram or thallium scan) or diagnostic changes on an electrocardiogram (such as during stress testing or an episode of chest pain)<br>2. Evidence of coronary artery disease based on coronary angiography or other diagnostic imaging<br>3. Use of medication given to treat or prevent angina (e.g., nitrates, beta blockers, calcium channel blockers)	**Probable 1+2:**<br><br>1. Other evidence of myocardial ischemia and/or coronary artery disease (including that based primarily upon symptoms and clinical presentation, such as chest pain with exertion)<br>2. Use of medication given to treat or prevent angina (e.g., nitrates, beta blockers, calcium channel blockers)
Deep vein thrombosis	**Confirmed 1:**<br>1. Diagnosis of deep vein thrombosis (DVT) by contrast venography, or **ultrasonography** other comparable imaging techniques;	**Probable (1)+2+3:**<br>1. An elevated **D-dimer test**;<br>2. A score on the Wells Clinical Prediction Rule for DVT of ≥ 3 points;<br>3. Absence of alternative diagnosis as likely or greater than that of deep venous thrombosis.<br>**Wells Clinical Prediction Rule for DVT (Appendix 6)**
**SYSTEMIC DISEASES**		
Anaemia<br>NOT FOR ERC<br>TO BE DETERMINED RETROSPECTIVELY	**Confirmed 1**<br>Classified according to both WHO and DAIDS thresholds for severe/grade 3-4 anaemia	
Chronic Kidney disease	**Confirmed: 1 or 2**<br><br>1. Kidney damage for >3 months, as defined by structural or functional abnormalities of the kidney with or without decreased GRF, manifest by either;<br>- Pathological abnormalities; or<br>- Markers of kidney damage, including abnormalities in the composition of the blood or urine or abnormalities in imaging results<br>2. GFR <60mL/min/1.73m2 for >3months, with or without kidney disease (estimated by CKD-EPI)	**Confirmed: 1 or 2**<br><br>1. Isolated Kidney damage, as defined by structural or functional abnormalities of the kidney with or without decreased GRF, manifest by either;<br>- Pathological abnormalities; or<br>- Markers of kidney damage, including abnormalities in the composition of the blood or urine or abnormalities in imaging results<br>2. Isolated GFR <60mL/min/1.73m2, with or without kidney disease (estimated by CKD-EPI)
End-stage renal disease	**Confirmed: 1**<br>1.Hemodialysis or peritoneal dialysis documented in a clinical note for a period of at least three months;	**Probable: 1**<br>1. Hemodialysis or peritoneal dialysis documented in a clinical note for a period of at least one month and up to the time of death in a patient who dies within three months after dialysis begins
Diabetes Mellitus<br><br>NOT FOR ERC<br>TO BE DETERMINED RETROSPECTIVELY	**Confirmed: 1 or 2 or 3 or 4**<br><br>1. Symptoms of diabetes plus casual plasma glucose concentration ≥ 200 mg/dL (11.1 *mmol/L*). (Casual is defined as any time of day without regard to time since last meal. The classic symptoms of diabetes include polyuria and polydipsia.)<br><br>2. Fasting plasma glucose ≥ 126 mg/dL (7.0 *mmol/L*). (Fasting is defined as no caloric intake for at least 8 hours.)<br><br>3. 2-hour post-load glucose ≥ 200 mg/dL (11.1 *mmol/L*) during an oral glucose tolerance test. (The test should be performed as described by WHO, using glucose load containing the equivalent of 75 g anhydrous glucose dissolved in water.)<br><br>4.An **HbA1c** of 48mmol/mol (6.5%) or above.	**None**

For peer review only

RHICCA – Clinical endpoint diagnostic criteria vs 3.5 Apr 2018

RHICCA
Reactivation of herpesviruses and inflammation as cardiovascular and cerebrovascular risk factors in ART initiators

Decompensate Liver disease	**Confirmed: 1+2**<br>1. Histologic, radiographic, or ultrasound evidence of cirrhosis, as documented by one of the following:<br>a. Histologic evidence of cirrhosis obtained by liver biopsy or autopsy<br>b. MRI or CT consistent with cirrhosis<br>c. A positive result on ultrasound imaging consistent with cirrhosis<br><br>2. Clinical evidence of decompensation, as documented by one of the following, and without an alternative explanation:<br>a. Ascites<br>b. Hepatic encephalopathy<br>c. Bleeding from gastric or esophageal varices<br>d. Spontaneous bacterial peritonitis	**Probable: 1**<br><br>1. Clinical evidence of decompensation, as documented by one of the following, and without an alternative explanation:<br>a. Ascites<br>b. Hepatic encephalopathy<br>c. Bleeding from gastric or esophageal varices<br>d. Spontaneous bacterial peritonitis
Hypertension	**Confirmed:** 1 or 2<br>1. An average of three blood pressure (BP) readings of systolic of 140 mm Hg or above and/or diastolic of 90 mm Hg or above on the same day and persist 5-7 days after the initial reading.<br>2. An isolated reading of 140mg systolic or 90mg diastolic and presence of the following end-organ disease:<br>a. Cardiac (i.e. left ventricular hypertrophy meeting the ECG criteria [**Appendix 2**] on evidence on cardiac echocardiogram)<br>b. Renal (i.e. microalbuminuria [urinary albumin excretion of 30-300mg/dl], elevated creatinine, reduced estimated GFR (60-90ml/min)<br>c. Retinal(i.e. hypertensive retinal changes)<br>d. Vascular disease (i.e. stroke [persisting on day 7], peripheral vascular disease, myocardial infarction, coronary artery disease requiring drug treatment, congestive cardiac failure)	**Probable: 1**<br><br>1. An average of three BP readings of systolic of 140 mm Hg or above and/or diastolic of 90 mm Hg or above on the same day.
Hyperlipidemia<br><br>NOT FOR ERC<br>TO BE DETERMINED RETROSPECTIVELY	**Confirmed: 1 or 2**<br>1. Fasting total cholesterol >200mg/dl (>5.2 mmol/L) or LDL cholesterol >130mg/dl (>3.4mmol/l) or Triglycerides >150 mg/dl (1.7 mmol/L)<br>2. Non-fasting total cholesterol >240mg/dl (>6.2 mmol/L) or LDL cholesterol >160mg/dl (>4.1 mmol/L) or Triglycerides >200 mg/dl (2.3mmol/L)	**None**
HIV wasting syndrome	None	**Probable:1 + 2 + 3**<br>1. unexplained, involuntary weight loss >10% from baseline,<br>2. persistent diarrhea with > 2 liquid stools/d for > 1 month or weakness for > 1 month or fever for > 1 month,<br>3. tests for alternate causes of weight loss, such as cancer, TB, MAC, cryptosporidiosis or other specific causes of weight loss, if obtained, should be negative

For peer review only

RHICCA – Clinical endpoint diagnostic criteria vs 3.5 Apr 2018

Appendix

1. Clinical syndrome of Myocardial infarction (a+ b +d) or (c+d)
    a. Chest pain (with associated clamminess, pallor)
    b. Radiation to the upper extremity and jaw
    c. Epigastric discomfort with exertion or at rest
    d. Severe discomfort lasting for more than 20 minutes

2. ECG criteria for LVH

Any two of the following 3 criteria's should be met:

For peer review only

Sokolow Lyon Criteria<br><br>• S in V_1 or V_2 + R in V_5 or V_6 (whichever is larger) ≥ 35 mm (≥ 7 large squares)<br>• R in aVL ≥ 11 mm<br><br>**Meets all of Sokolow Lyon criteria to be diagnostic**	
Cornell voltage criteria<br><br>ECG diagnosis of LVH involve measurement of the sum of the R wave in lead aVL and the S wave in lead V_3. The Cornell criteria for LVH are:<br><br>• S in V_3 + R in aVL > 28 mm (men)<br>• S in V_3 + R in aVL > 20 mm (women)<br><br>**Meets all of Cornell voltage criteria to be diagnostic**	
Romhilt-Estes point score system ECG Criteria<br>Voltage Criteria (any of):<br>1. R or S in limb leads ≥20 mm<br>2. S in V_1 or V_2 ≥30 mm<br>3. R in V_5 or V_6 ≥30 mm<br>---<br>ST-T Abnormalities:<br><br>1. ST-T vector opposite to QRS without digitalis<br>2. ST-T vector opposite to QRS with digitalis<br>3. Negative terminal P mode in V_1 1 mm in depth and 0.04 sec in duration (indicates left atrial enlargement)<br>4. Left axis deviation (QRS of -30° or more)<br>5. QRS duration ≥0.09 sec<br>6. Delayed R wave peak time (intrinsicoid deflection) in V_5 or V_6 (>0.05 sec)<br><br>**Romhilt-Estes point score >4 is diagnostic**	Points<br><br>3<br><br><br><br><br><br><br>3<br>1<br>3<br><br>2<br>1<br>1

3. Clinical syndrome of Peripheral vascular disease (a+ (b or c or d)
    a. Painful cramping in the hip, thigh or calf muscles after certain activities, such as walking or climbing stairs (claudication)
    b. femoral bruit
    c. decreased peripheral pulses
    d. change in color or temperature of limb suggesting peripheral arterial disease

For peer review only - http://bmjopen.bmj.com/site/about/guidelines.xhtml

RHICCA – Clinical endpoint diagnostic criteria vs 3.5 Apr 2018

RHICCA
Reactivation of herpesviruses and inflammation as cardiovascular and cerebrovascular risk factors in ART initiators

4. Clinical syndrome of stroke; should meet the 3 criteria's;

1. **Sudden onset**	
2. **Focal deficit** (or global disturbance but not seizures)	**Large artery disease (anterior circulation syndrome)**<br>Hemi-paresis + Hemi-sensory loss + higher cortical dysfunction (gaze paresis, language impairment [expression + comprehension], visual field defect, hemi-neglect)<br><br>**Large artery disease (posterior circulation syndrome)**<br>Vertigo, visual field defect, gaze paresis, double vision, swallowing difficultly, crossed signs [contralateral limb weakness and ipsilateral cranial nerves abnormality], ataxic limb and gait, drowsy/loss of consciousness<br><br>**Small vessel disease (lacunar syndrome)**<br>Pure hemi-sensory loss<br>Pure hemiparesis<br>Pure sensorimotor<br>Pure ataxic hemiparesis (including dysarthria-clumsy hand syndrome)<br><br>Thunderclap headache*
3. **Lasting > 24 hours** (<24 hours is a TIA)	
*seen in those with a suspicion of subarachnoid or venous stroke. In this case criteria 1 and 3 does not necessarily have to be met	

5. Clinical syndrome of congestive heart failure:

Using the Framingham criteria relies on clinical signs and symptoms; 1 or more major and two or more minor criteria are clinically suggestive of heart failure:

Major criteria

A. Acute pulmonary edema
B. Cardiomegaly
C. Hepatojugular reflex
D. Neck vein distention
E. Paroxysmal nocturnal Dyspnea or Orthopnea
F. Pulmonary crackles
G. Third Heart Sound (S3 Gallup Rhythm)

Minor Criteria

A. Ankle edema
B. Dyspnea on exertion
C. Hepatomegaly
D. Nocturnal cough
E. Pleural Effusion
F. Tachycardia (Heart Rate >120 beats per minute)

For peer review only

RHICCA – Clinical endpoint diagnostic criteria vs 3.5 Apr 2018

RHICCA
Reactivation of herpesviruses and inflammation as cardiovascular and cerebrovascular risk factors in ART initiators

6. Wells Clinical Prediction Rule for DVT (Adapted from: Wells PS et al. Lancet 1997;350:1796).

One point for each of the following:

- Active cancer (treatment ongoing or within previous 6 months, or palliative)
- Paralysis, paresis, or plaster immobilization of lower extremities
- Recently bedridden for more than 3 days, or major surgery, within 4 weeks
- Localized tenderness along distribution of the deep venous system
- Entire leg swollen
- Calf swelling by more than 3 cm when compared with the asymptomatic leg (measured 10 cm below tibial tuberosity)
- Pitting edema (greater in the symptomatic leg)
- Collateral superficial veins (non-varicose)

7. Clinical symptoms of meningism
   Meninigism is the triad of nuchal **rigidity** (neck stiffness), **photophobia** (intolerance of bright light) and **headache**.

8. Clinical symptoms of norcardia

   Symptoms vary and depend on the organs involved.

   If in the lungs, symptoms may include:
   - Chest pain when breathing (may occur suddenly or slowly)
   - Coughing up blood
   - Fevers
   - Night sweats
   - Weight loss

   If in the brain, symptoms may include:
   - Fever
   - Headache
   - Seizures
   - If the skin is affected, symptoms may include:
   - Skin breakdown
   - Skin breakdown and abnormal passage or draining tract (fistula)
   - Ulcers or nodules with infection sometimes spreading along lymph nodes

   Some people with nocardia infection have no symptoms.

9. Symptoms of Pneumocystis Pneumonia
   - Fever.
   - Mild and dry cough or wheezing.
   - Shortness of breath, especially with activity.
   - Rapid breathing.
   - Fatigue.
   - Major weight loss.
   - Chest pain when you breathe.

10. Clinical syndrome of bacterial pneumonia
    - cough with thick yellow, green, or blood-tinged mucus.
    - chest pain that worsens when coughing or breathing.
    - sudden onset of chills.
    - fever of 102°F or above (fever lower than 102°F in older persons)
    - headache.
    - muscle pain.
    - breathlessness or rapid breathing.

For peer review only
For peer review only - http://bmjopen.bmj.com/site/about/guidelines.xhtml

11. Clinical finding of Central Nervous System PML
    - Deficits in motor function, especially **weakness** and **clumsiness**, are common
    - associated altered mental state or behaviour and fever

12. Clinical finding of CNS toxoplasmosis
    - Headaches
    - Seizures
    - Focal neurological deficit of a subacute onset
    - confusion and coma
    - A lung infection, causing cough, fever, and shortness of breath may co-exist.
    -

13. Clinical symptoms suggest of Aspergillosis;

- Fever and chills.
- Cough that brings up blood-streaked sputum (hemoptysis)
- Severe bleeding from the lungs.
- Shortness of breath.
- Chest or joint pain.
- Headaches or eye symptoms.
- Nosebleed
- Facial swelling on one side

For peer review only

Table 1: Abbreviated NARS (Neuropsychiatric AIDS Rating Scale) Grading for HIV Encephalopathy
Adapted from: Price RW, Brew BJ. The AIDS dementia complex. J Infect Dis 1988; 158 (5): 1079-83, and Hughes CP, Berg L, Danziger WL. A new clinical scale for the staging of dementia. Brit J Psych 1982; 140: 566-92.

NARS stage	Cognitive-Behavioral Domains					
	**Orientation**	**Memory**	**Motor**	**Behavior**	**Problem solving**	**Activities of daily living**
**0.5**	fully oriented	complains of memory problems	fully ambulatory slightly slowed movements	normal	has slight mental slowing	slight impairment in business dealings
**1**	fully oriented, may have brief periods of “spaciness"	mild memory problems	balance, co-ordination and handwriting difficulties	more irritable, labile or apathetic, withdrawn	difficulty planning and completing work	can do simple daily tasks, may need prompting
**2**	some disorientation	memory moderately impaired, new learning impaired	ambulatory but may require walking aid	some impulsivity or agitated behavior	severe impairment, poor social judgement, gets lost easily	needs assistance with ADLs
**3**	frequent disorientation	severe memory loss, only fragments of memory remain	ambulatory with assistance	may have organic psychosis	judgement very poor	cannot live independently
**4**	confused and disoriented	virtually no memory	bedridden	mute and unresponsive	no problem solving ability	nearly vegetative

For peer review only - http://bmjopen.bmj.com/site/about/guidelines.xhtml

RHICCA – Clinical endpoint diagnostic criteria vs 3.5 Apr 2018

RHICCA
Reactivation of herpesviruses and inflammation as cardiovascular and cerebrovascular risk factors in ART initiators

For peer review only

For peer review only - http://bmjopen.bmj.com/site/about/guidelines.xhtml

RHICCA – Clinical endpoint diagnostic criteria vs 3.5 Apr 2018

RHICCA
Reactivation of herpesviruses and inflammation as cardiovascular and cerebrovascular risk factors in ART initiators

Table 2: Diagnostic criteria for classification of definite, probable, possible, and not tuberculosis meningitis (Marais S, et al. Lancet Infect Dis 2010)

	Diagnostic score
**Clinical criteria**	(Maximum category score=6)
Symptom duration of more than 5 days	4
Systemic symptoms suggestive of tuberculosis (one or more of the following): weight loss (or poor weight gain in children), night sweats, or persistent cough for more than 2 weeks	2
History of recent (within past year) close contact with an individual with pulmonary tuberculosis or a positive TST or IGRA (only in children <10 years of age)	2
Focal neurological deficit (excluding cranial nerve palsies)	1
Cranial nerve palsy	1
Altered consciousness	1
**CSF criteria**	(Maximum category score=4)
Clear appearance	1
Cells: 10–500 per µl	1
Lymphocytic predominance (>50%)	1
Protein concentration greater than 1 g/L	1
CSF to plasma glucose ratio of less than 50% or an absolute CSF glucose concentration less than 2.2mmol/L	1
**Cerebral imaging criteria**	(Maximum category score=6)
Hydrocephalus	1
Basal meningeal enhancement	2
Tuberculoma	2
Infarct	1
Pre-contrast basal hyperdensity	2
Evidence of tuberculosis elsewhere	(Maximum category score=4)
**Chest radiograph** suggestive of active tuberculosis: signs of tuberculosis=2; miliary tuberculosis=4	2/4
**CT/ MRI/** ultrasound evidence for tuberculosis outside the CNS 2	2
**AFB** identifi ed or *Mycobacterium tuberculosis* cultured from another source—ie, sputum, lymph node, gastric washing, urine, blood culture	4
Positive commercial *M tuberculosis* NAAT from extra-neural specimen	4

Exclusion of alternative diagnoses

An alternative diagnosis must be confirmed microbiologically (by stain, culture, or NAAT when appropriate), serologically (eg, syphilis), or histopathologically (eg, lymphoma). The list of alternative diagnoses that should be considered, dependent upon age, immune status, and geographical region, include: pyogenic bacterial meningitis, cryptococcal meningitis, syphilitic meningitis, viral meningo-encephalitis, cerebral malaria, parasitic or eosinophilic meningitis (*Angiostrongylus cantonesis*, *Gnathostoma spinigerum*, toxocariasis, cysticercosis), cerebral toxoplasmosis and bacterial brain abscess (space-occupying Lesion on cerebral imaging)and malignancy (eg, lymphoma)

TST=tuberculin skin test. IGRA=interferon-gamma release assay. NAAT=nucleic acid amplification test. AFB=acid-fast bacilli. The individual points for each criterion (one, two, or four points) were determined by consensus and by considering their quantified diagnostic value as defined in studies.

Key:
Bold text: of the options available likely to be the only tool available in a Malawi setting
Greyed out text: ideal investigation but not available in a Malawi setting

For peer review only - http://bmjopen.bmj.com/site/about/guidelines.xhtml

For peer review only
